# Haplotype-based inference of the distribution of fitness effects

**DOI:** 10.1093/genetics/iyac002

**Published:** 2022-01-09

**Authors:** Diego Ortega-Del Vecchyo, Kirk E Lohmueller, John Novembre

**Affiliations:** 1 Laboratorio Internacional de Investigación sobre el Genoma Humano, Universidad Nacional Autónoma de México, Juriquilla, Querétaro 76230, México; 2 Interdepartmental Program in Bioinformatics, University of California, Los Angeles, Los Angeles, CA 90095, USA; 3 Department of Ecology and Evolutionary Biology, University of California, Los Angeles, Los Angeles, CA 90095, USA; 4 Department of Human Genetics, David Geffen School of Medicine, University of California, Los Angeles, Los Angeles, CA 90095, USA; 5 Department of Human Genetics, University of Chicago, Chicago, IL 60637, USA; 6 Department of Ecology and Evolution, University of Chicago, Chicago, IL 60637, USA

**Keywords:** haplotype, selection, inference, DFE

## Abstract

Recent genome sequencing studies with large sample sizes in humans have discovered a vast quantity of low-frequency variants, providing an important source of information to analyze how selection is acting on human genetic variation. In order to estimate the strength of natural selection acting on low-frequency variants, we have developed a likelihood-based method that uses the lengths of pairwise identity-by-state between haplotypes carrying low-frequency variants. We show that in some nonequilibrium populations (such as those that have had recent population expansions) it is possible to distinguish between positive or negative selection acting on a set of variants. With our new framework, one can infer a fixed selection intensity acting on a set of variants at a particular frequency, or a distribution of selection coefficients for standing variants and new mutations. We show an application of our method to the *UK10K* phased haplotype dataset of individuals.

## Teaser

Ortega-Del Vecchyo *et al*. describe a new method to infer the distribution of fitness effects (DFE). The majority of previous methods use data from the site frequency spectrum and ignore information from linked genetic variation. This new method uses pairwise haplotype identity-by-state lengths to infer the DFE acting on a set of variants with a particular frequency in the population and shows that the DFE inferred from variants at a particular frequency can be used to infer the DFE of new mutations.

## Introduction

The distribution of fitness effects of new mutations, DFE, is a probability distribution that quantifies the proportion of new mutations having a certain selection coefficient s, where s can take positive or negative values depending on whether the new allele is under positive or negative selection. The DFE has a direct impact on current levels of genetic variation, since the frequencies of the alleles depend on their selection coefficient (Sawyer and Hartl 1992; Hartl *et al.* 1994; Bustamante *et al.* 2001), and alleles under selection change the genetic variation at linked sites due to the effects of linked selection (Smith and Haigh 1974; Charlesworth *et al.* 1993). Moreover, the DFE is a key feature in the evolution of complex phenotypic traits (Simons *et al.* 2014; Lohmueller 2014a; Mancuso *et al.* 2016), since the association between the selection coefficients and the effect of mutations on a complex trait is an important determinant of the genetic architecture of a trait (Eyre-Walker 2010). Due to the impact of the DFE on levels of genetic and phenotypic variation, properly inferring the DFE is essential to many fundamental problems such as validating predictions of the nearly neutral theory (Kimura and Crow 1964; Crow 1972; Ohta 1992), understanding changes in the deleterious segregating variation observed in different populations (Gazave *et al.* 2013; Lohmueller 2014b; Henn *et al.* 2015; Brandvain and Wright 2016; Gravel 2016; Simons and Sella 2016; Koch and Novembre 2017), elucidating the factors that influence changes on the DFE between species (Martin and Lenormand 2006; Charlesworth and Eyre-Walker 2007; Serohijos and Shakhnovich 2014; Tenaillon 2014; Rice *et al.* 2015; Huber *et al.* 2017), and inferring the amount of adaptive evolution between species (Gossmann *et al.* 2012; Galtier 2016; Zhen *et al.* 2018).

Broadly, 2 lines of research have been developed to infer a DFE. One is based on experimental approaches and the other one is based on the analysis of population genetic variation at putatively neutral and deleterious sites. The main experimental approaches taken with viruses, bacteria, and yeast are site-directed mutagenesis experiments in target regions (Bataillon and Bailey 2014) and mutation–accumulation experiments (Halligan and Keightley 2009). Advantageous mutations tend to be rare or not found in results from experimental approaches (Halligan and Keightley 2009; Lind *et al.* 2010; Jacquier *et al.* 2013; Bataillon and Bailey 2014) with some exceptions (Sanjuán *et al.* 2004; Dickinson 2008; Böndel *et al.* 2019). Due to this, some studies have focused on inferring the distributional form for the DFE taking neutral and deleterious mutations. The types of probability distributions that have provided a good fit to the DFE of neutral and deleterious mutations in site-directed mutagenesis experiments are a gamma distribution (Domingo-Calap *et al.* 2009; Lind *et al.* 2010; Jacquier *et al.* 2013), a unimodal distribution with a similar shape to a gamma distribution (Sanjuán *et al.* 2004; Domingo-Calap *et al.* 2009; Peris *et al.* 2010), and a bimodal distribution with one part of the probability mass on nearly neutral mutations and the other one on the highly deleterious mutations (Hietpas *et al.* 2011).

The other main approach is to use population genetic variation data to estimate the DFE with information from the site frequency spectrum (SFS) on putatively neutral and deleterious sites (Sawyer and Hartl 1992; Williamson *et al.* 2005; Keightley and Eyre-Walker 2007; Boyko *et al.* 2008; Gutenkunst *et al.* 2009; Kim *et al.* 2017). The first step in these approaches is to infer the demographic scenario that fits the SFS at putatively neutral sites, which typically are chosen to be variants at synonymous sites. The DFE is then inferred from putatively deleterious sites of interest, typically nonsynonymous sites, while taking the demographic scenario into account. An interesting extension has recently been developed to take SFS information and divergence data from an outgroup to infer the DFE from the population where the SFS data were taken along with the rate of adaptive molecular evolution based on the divergence data (Tataru *et al.* 2017). Two other extensions have been taken to model the correlation between the fitness effects of multiple nonsynonymous alleles at a particular position (Ragsdale *et al.* 2016) and to calculate the joint DFE between pairs of populations (Fortier *et al.* 2019). Some species where these approaches have been applied to infer the DFE include humans (Eyre-Walker *et al.* 2006; Boyko *et al.* 2008; Li *et al.* 2010; Huber *et al.* 2017; Kim *et al.* 2017), mice (Halligan *et al.* 2013; Kousathanas and Keightley 2013), and *Drosophila melanogaster* (Kousathanas and Keightley 2013; Huber *et al.* 2017). Studies that compare the fit of different probability distributions argue in favor of a DFE of deleterious nonsynonymous mutations in humans that follows either (1) a gamma distribution (Boyko *et al.* 2008; Kim *et al.* 2017) or (2) a combination of a point mass at neutrality plus a gamma distribution (Kim *et al.* 2017). Those 2 studies infer a leptokurtic DFE with a proportion of nearly neutral mutations (*s* < 10^−5^) of 18.3–26.3%, and moderate to strong deleterious mutations (*s* > 10^−3^) of 46.6–57.4%.

One drawback of the majority of current methods that estimate the DFE using population genetic variation is that they ignore all linkage information. To our knowledge, the only exception is a recent study using an approximate Bayesian computation approach that includes linkage disequilibrium statistics in the analysis (Johri *et al.* 2020). The lack of studies exploiting the information from linked genetic variation to estimate the DFE is surprising given the fact that many studies have analyzed how both deleterious (Charlesworth *et al.* 1993, 1995; Hudson and Kaplan 1995; Nordborg *et al.* 1996; Nicolaisen and Desai 2013; Cvijović *et al.* 2018) and advantageous variants (Smith and Haigh 1974; Kaplan *et al.* 1989; Braverman *et al.* 1995; Nielsen 2005) decrease linked genetic variation. Further, linked genetic variation has been effectively used to infer the age of particular variants (Slatkin and Rannala 1997; Tishkoff *et al.* 2007; Chen and Slatkin 2013; Mathieson and McVean 2014; Chen *et al.* 2015; Nakagome *et al.* 2016; Ormond *et al.* 2016; Albers and McVean 2018), the coalescent time between rare-variant carrying chromosomes with chromosomes not carrying the rare-variant (Platt *et al.* 2019), the time to the common ancestor of a positively selected allele (Smith *et al.* 2018), the time since fixation of an advantageous allele (Przeworski 2003), the selection coefficient of an allele (Slatkin 2001, 2008; Coop and Griffiths 2004; Tishkoff *et al.* 2007; Chen and Slatkin 2013; Chen *et al.* 2015; Ormond *et al.* 2016), and to detect loci under positive selection (Kim and Stephan 2002; Sabeti *et al.* 2002, 2007; Voight *et al.* 2006; Wang *et al.* 2006; Tang *et al.* 2007; Williamson *et al.* 2007; Pavlidis *et al.* 2010; Li 2011; Ferrer-Admetlla *et al.* 2014; Garud *et al.* 2015; Field *et al.* 2016; Huber *et al.* 2016). Since there has been so much progress in understanding how selection changes the linked variation around individual variants, it should be feasible to pool the haplotype information from many variants putatively under selection at a certain frequency f to infer the DFE and the distribution of fitness effects of variants at a frequency, which we will call $DFE_{f}$.

Here, we propose a new approach to infer $DFE_{f}$. We note that $DFE_{f}$ is different from the distribution of fitness effects of new mutations entering the population, which we call the DFE. For instance, natural selection can act to increase the frequency of advantageous variants and to decrease the frequency of deleterious variants, causing a difference between DFE and $DFE_{f}$. The relationship between $DFE_{f}$ and DFE is one of the topics we will address in this study.

Recent large population genomic datasets such as the *UK10K* (Walter *et al.* 2015), the Netherlands Genome Project (Francioli *et al.* 2014), the Haplotype Reference Consortium (McCarthy *et al.* 2016), and the NHLBI TOPMed Program (Taliun *et al.* 2019) provide an unprecedented source of haplotype information to quantify both the $DFE_{f}$ and the DFE. These datasets have started to be exploited to understand the impact of selection on variants at a certain frequency. For example, Kiezun *et al.* (2013) found that, conditioning on the variants having a certain frequency f in the population, nonsynonymous variants have more extended linkage disequilibrium with neighboring neutral variation compared with synonymous variants in data from the Netherlands Genome Project. This is in line with Takeo Maruyama’s results showing that deleterious variants at a certain frequency have a younger age compared with neutral variants (Maruyama 1974), implying that there is less variation on haplotypes carrying deleterious variants.

Building on previous work to estimate the strength of selection acting on advantageous variants (Slatkin 2001; Chen and Slatkin 2013), we propose an approach to provide a point estimate of the population-scaled selection coefficient or a distribution of fitness effects acting on a set of variants at a particular frequency f ($DFE_{f}$). We infer the strength of natural selection using pairwise haplotypic identity-by-state lengths (the length in one direction along a pair of haplotypes carrying a focal allele to the first difference between the pair of haplotypes). For each pair j of haplotypes we define the observed length as $L_{j}$. The length can be measured in both directions along the chromosome extending outward from the focal allele. We show that these lengths can be used to distinguish between alleles under positive and negative selection in some nonequilibrium demographic scenarios. Further, we show how the $DFE_{f}$ can be used to infer the DFE. The resulting method can help improve the understanding of how selection is influencing, for instance, the low-frequency variants present in a population. Finally, we show an application of our method to the *UK10K* dataset.

## Materials and methods

### A method for inference of the population-scaled selection coefficient based on haplotype variation

Our analysis is based on a set of ℓ haplotype pairs carrying a derived focal allele at a sample allele frequency f in a focal site (see Supplementary Table 1 for the notation used in this study). The information from haplotypes carrying the ancestral allele is ignored in our method following methods that infer the impact of natural selection based on the genealogical patterns of haplotypes carrying a derived allele (Slatkin and Rannala 1997; Slatkin 2001). Haplotypes with the ancestral allele contain information that could be useful to infer the impact of natural selection because their diversity patterns are dependent on the strength of natural selection acting on a derived allele. However, information provided by haplotypes carrying the ancestral allele would greatly increase the computation time of our method since it would require the analysis of the haplotypes containing the ancestral allele. That information is also partly redundant since the frequency changes of the derived allele directly change the frequency of the ancestral allele.

**Fig. 1.**
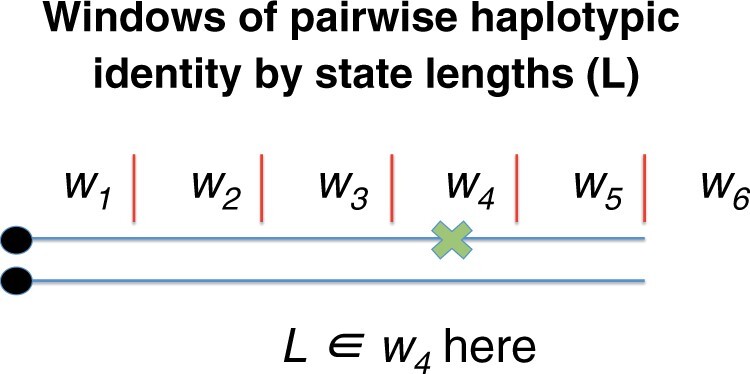
Two haplotypes containing a derived allele, here represented as a black dot, that has a frequency *f*. The physical distance near the allele at a focal site is divided into 5 nonoverlapping equidistant windows of a certain length, with an extra window *w_6_* indicating that there are no differences in any of the windows *w_1_* to *w_5_*. The first difference between the pairs of haplotypes is denoted by the green “x.”

We compute the pairwise identity by state length *L_j_* for every haplotype pair, which is defined as the distance from the derived allele at a focal site to the first difference between a pair of haplotypes. For computational simplicity, we bin the chromosome under analysis into a set of M discrete nonoverlapping windows $W=\{w_{1},w_{2},\ldots ,\;w_{M}\}$ that extend to the side of the derived allele at a focal site. Thus, for a set of ℓ haplotype pairs carrying an allele, our analysis is based on which window the first difference appears in for each haplotype pair ($L=\{L_{1}\;\in w_{m_{1}},\;L_{2}\in w_{m_{2}},\;L_{3}\in w_{m_{3}},\;\ldots ,\;L_{\ell }\in w_{m_{\ell }}\}$). We define $m_{1},\ldots ,m_{\ell }$ as integers between 1 and M indicating the windows in which each length falls. The majority of the analysis in this paper use *M* = 6 discrete nonoverlapping windows as seen in Fig. 1. When *M* is different, we note it in that particular analysis. We can calculate a length $L_{j}$ for all possible pairwise comparisons of n haplotypes containing the derived allele to obtain $\ell =\left(\begin{array}{c} n\\ 2 \end{array}\right)$ values of L. This procedure can be done going upstream and downstream to obtain $\ell =2\times \left(\begin{array}{c} n\\ 2 \end{array}\right)$ values of L. Further, if we take a number A of loci where the derived focal allele has a frequency f, we will observe a total number $\ell =2\times \;A\times \left(\begin{array}{c} n\\ 2 \end{array}\right)$ of L length values.

For our inference procedure, we will consider each $L_{j}$ independently and so we momentarily refer generically to a single observed length as L. The parameter we wish to infer is the population scaled selection coefficient 4Ns. For the moment, we assume that all the derived alleles in the A loci share the same selection coefficient 4Ns. That parameter is defined in terms of the effective population size N from the most ancient epoch in the demographic scenario D. It is also possible to define the population scaled selection coefficient in terms of the most recent epoch. If the population size of the most recent epoch is $N_{R}$ and we continue with our definition of the effective population size in the most ancient epoch as N, then the population scaled selection coefficient in the most recent time is equal to $4N_{R}s$.

The likelihood of a particular population scaled selection coefficient, 4Ns, conditioned on the frequency f of a derived allele at a focal site and a demographic scenario D with a single observed length L that falls in a window $w_{j}$ can be expressed as:

(1) $$L(4Ns,f,D|L\in w_{j})=P(L\in w_{j}|4Ns,f,D)=\int P\left(L\in w_{j}|4Ns,\;f,\;D,H_{k}\right)P\left(H_{k}|4Ns,f,\;D\right)dH_{k}=\int P\left(L\in w_{j}|\;D,H_{k}\right)P\left(H_{k}|4Ns,f,\;D\right)dH_{k}$$

where $H_{k}$ is a particular allele frequency trajectory of the set of all possible allele frequency trajectories H. $H_{k}$ is a vector listing the number of derived alleles at a focal site per generation since the emergence of the allele until the present. A sample of chromosomes is taken from the present and the allele frequency of the derived allele in that sample is equal to f while the number of chromosomes containing the derived allele will be equal to n. Note that $P\left(L\in w_{j}|4Ns,\;f,\;D,{\;H}_{k}\right)=P\left(L\in w_{j}|\;{D,\;H}_{k}\right)$ in this equation. The interpretation of this equality is that the distribution of L values is just dependent on $H_{k}$ and D even when we have additional information on 4Ns and f. In the right hand of the equation, we are performing an integration over the space of allele frequency trajectories $H_{k}$. We can compute $P\left(L\in w_{j}|{D,H}_{k}\right)$ via Monte–Carlo simulations done using *mssel* (Kindly provided by Richard Hudson), which simulates haplotypes containing a derived allele whose frequency trajectory is determined by $H_{k}$ under the demographic scenario D (Hudson and Kaplan 1988; Kaplan *et al.* 1988). To do this, we used *mssel* to simulate 100 sets of n haplotypes for each realization of $H_{k}$ (see below for sampling of $H_{k}$) under the demographic scenario D with the derived allele loci located at the left end of the simulated region as shown in Fig. 1. We computed $\ell =\left(\begin{array}{c} n\\ 2 \end{array}\right)$ values of L for each set of n simulated haplotypes by estimating L in all the possible comparisons of haplotype pairs containing the derived allele. We used n = 40 haplotypes with the derived allele for all scenarios but the *UK10K* scenario, where we used n = 72 haplotypes to mimic the number of haplotypes sampled when the derived allele has a 1% allele frequency in the *UK10K* dataset. We pooled the collection of L values across the 100 sets of haplotypes to estimate the probability $P\left(L\in w_{j}|{D,\;H}_{k}\right)$. It is important to appreciate that these Monte–Carlo simulations of haplotypes must include information about the recombination rate per base *r* and mutation rate per base *u* present in the simulated region of length *l*. Using the appropriate *r* and *u* values is important because those parameters determine the values of L.

The integration over the space of allele frequency trajectories $H_{k}$ is challenging since we specifically need to sample trajectories $H_{k}$ where the derived allele has a frequency f in the present. Here, we performed the integration over the space of allele frequency trajectories using an importance sampling approach where we only sample $H_{k}$ that have a present-day frequency f (see Appendix). The effectiveness of our importance sampling approach to evaluate $L(4Ns,f,D|L\in w_{j})$ is given, in part, by the effective sample size (ESS) which is an estimate of the sample size used in a Monte–Carlo evaluation of $L(4Ns,f,D|L\in w_{j})$ that is equivalent to the estimate we obtain using our importance sampling approach. Higher values of the ESS are necessary, but not sufficient to obtain more accurate estimates of $L(4Ns,f,D|L\in w_{j})$. More information on how to calculate the ESS is given in Appendix.

Finally, given a set of values of $L=\left\{L_{1}\;\in w_{m_{1}},\;L_{2}\in w_{m_{2}},\;L_{3}\in w_{m_{3}},\;\ldots ,\;L_{\ell }\in w_{m_{\ell }}\right\},$ we can estimate the composite likelihood of having that set of L values as:

(2) $$L(4Ns,f,D|L)=\prod_{j=1}^{\ell }L(4Ns,f,D|L_{j}\in w_{m_{j}}).$$

An estimator of 4Ns can be obtained by maximizing this composite likelihood function, which here we do simply by using a grid search over a range of candidate 4Ns values going from −200 to 200. Note that in Equations (1) and (2) we have 3 parameters: f, D, and 4Ns. When we perform our grid approach to find a 4Ns estimate we set f and D as fixed parameters and we only try to estimate the value of 4Ns.

We defined 6 nonoverlapping windows $W=\{w_{1},\;w_{2},\;w_{3},\;w_{4},\;w_{5},\;w_{6}\}$*= {(0, 50*,*000], (50*,*000, 100*,*000], (100*,*000, 150*,*000], (150*,*000, 200*,*000], (200*,*000, 250*,*000], (250*,*000*, ∞*)}* in the majority of our analysis. We chose this particular window length after inspecting the L distribution in the *UK10K* 1% ±0.05% derived allele frequency nonsynonymous variants. Choosing this particular window length gave us more than 5% of the window lengths falling into each window $w_{j}$. The probabilities of L falling on the windows $w_{1},\;w_{2},\;w_{3},\;w_{4},\;w_{5}\;$and $w_{6}$ are equal to 39.23%, 21.39%, 13.99%, 8.18%, 5.19%, and 12.03%, respectively. Each window was potentially informative of the strength of selection because they contained at least more than 5% of the haplotype lengths L falling on them. The analysis where we used a different number of windows or window length are noted in the caption of the figure related to each analysis.

### Forward-in-time simulations to assess the impact of selection on the allele frequency trajectories, allele ages, pairwise coalescent times $T_{2}$, and and L values

We performed forward-in-time simulations using *PReFerSim* (Ortega-Del Vecchyo *et al.* 2016) to build an understanding of the inference problem and the method’s performance by assessing the impact of selection on allele frequency trajectories, pairwise coalescent times $T_{2}$, and haplotype identity-by-state-lengths L. *PReFerSim* performs simulations under the Poisson Random Field model (Sawyer and Hartl 1992), where the number of new unlinked independent mutations that enter the population each generation follows a Poisson distribution with a mean equal to $\theta /2=(4N_{j}ul/2)$ and their changes in allele frequency per generation are determined by a Wright–Fisher model with selection. $N_{j}$ is the population size in generation j, u is the mutation rate per base, and l is the number of bases. All of our simulations were done using a θ value equal to 1,000 for the most ancestral epoch. The allele frequency trajectory $H_{k}$ of each allele can be obtained in simulations done with *PReFerSim*.

We used *PReFerSim* to obtain 10,000 independent allele frequency trajectories $H_{k}$ of a derived allele with a 1% frequency f in the present in a sample of 4,000 chromosomes for each value of 4Ns explored in the demographic scenarios analyzed but the *UK10K* demographic scenario. The derived allele appears in the focal site (see Fig. 1). Each $H_{k}$ represents the frequency change of an independent and different focal derived allele at a focal site. In the case of the *UK10K* demographic scenario, we sampled 7,242 chromosomes and retained those trajectories where f= 1% ± 0.05% to mimic the number of chromosomes and the allele frequency of the sites we retained to perform inferences of selection. The allele ages associated to each $H_{k}$ were also recorded. We also estimated the distribution of pairwise coalescent times $T_{2}$ associated with each $H_{k}$ using an analytical formula (Appendix).

We used the 10,000 $H_{k}$ generated for each 4Ns value in each demographic scenario to test our inferences of 4Ns. To do this, we performed simulations where each simulation replicate has $\ell =2\times A\times \left(\begin{array}{c} n\\ 2 \end{array}\right)$ values of L. A is equal to 300 and n=40 to obtain $\ell =2\times 300\times \left(\begin{array}{c} 40\\ 2 \end{array}\right)=468,000$ values of L in each simulation replicate but the ones performed under the *UK10K* demographic scenario (Appendix). The value of A=300 was chosen to have a similar A number to the f=1% ± 0.05% nonsynonymous variants in the *UK10K* dataset A=275. The value of n (40) was chosen to have a n value of the same order of magnitude to what is observed in the *UK10K* dataset (69–76). We tested 100 simulation replicates in each examined value of 4Ns per demographic scenario. We used the following algorithm to obtain a simulation replicate:

1. Sample a random trajectory $H_{k}$ from the available allele frequency trajectories (10,000 in the case of the estimation of point population-scaled values of selection 4Ns).
2. Simulate n haplotypes with *mssel* that contain a derived allele with a trajectory determined by $H_{k}$. The derived allele is always set in the midpoint of the simulated haplotypic region. These simulations must be performed with a defined average per base mutation rate u, an average per base recombination rate r and a specified length l for the simulated region. We used $u=1.2\;X\;10^{-8}$, $r=1.0\;X\;10^{-8}$ and l=500 kp for almost all the simulations done under the constant population size model and the population expansion model. The analysis where we used a different value of u or r are explicitly mentioned in the figure caption accompanying each analysis.
3. Calculate $\ell =2\times \left(\begin{array}{c} n\\ 2 \end{array}\right)\;$values of L by performing all pairwise comparisons of haplotypes containing the derived allele. Since the derived allele is located in the midpoint of the simulated region, we can calculate the L values going upstream and downstream of the loci containing the derived allele.
4. Go back to 1) until you have simulated $\ell =2\times A\times \left(\begin{array}{c} n\\ 2 \end{array}\right)$ values of L, where A is the number of independent loci that contain a derived allele at a frequency *f.*

Our analyses were focused mainly in 2 demographic models: (1) a constant population size model with 10,000 individuals and (2) a population expansion model where 100 generations ago the population grew from 5,000 to 50,000 individuals. We chose to analyze the constant population size model to investigate what happens under a very simple demographic model where we have analytical theory to explain the age of a particular allele as a function of its frequency (Maruyama 1974). The population expansion model has a recent 10-fold population size growth, similar to documented population size changes on human populations (Schiffels and Durbin 2014). We also include an analysis under a population bottleneck where the population is temporarily reduced from 5,000 to 1,000 individuals between 5,000 and 5,200 generations ago; 3 population expansion models where the number of individuals grew from 5,000 to 50,000 individuals at a different number of generations ago (1,000, 10,000, and 100,000); and 2 realistic demographic models (Schiffels and Durbin 2014; Tennessen *et al*. 2012) from the Yoruba and African population. Those analyses were added to test the performance of our method to infer 4Ns under more demographic scenarios. The values of 4Ns analyzed in those scenarios were equal to 0, 50, 100, −50, and −100 for the constant population size and population expansion scenario. The 4Ns values used for other scenarios are noted in the caption of the figure associated to each analysis.

### A method for inference of the distribution of fitness effects for variants found at a particular frequency (“$DFE_{f}$”)

Our composite likelihood framework is extendible to find the distribution of fitness effects $DFE_{f}$ for a set of variants at a particular frequency f. This distribution, which we denote as $DFE_{f}$, is different from the canonical DFE, which represents the distribution of fitness effects of new mutations. To parameterize the $DFE_{f}$ we use a discretized gamma distribution following studies that use a gamma distribution (Boyko *et al.* 2008; Kim *et al.* 2017) under the assumption that the $DFE_{f}$ is only composed of neutral or deleterious mutations. The values of 4Ns presented here will refer to the effect of deleterious or neutral mutations. We parameterize the gamma component with 2 parameters that represent the shape α and scale β. We discretize the distribution to intervals centered on the integer values of 4Ns, and then collapse the tail probability of all values greater than a threshold fixed 4Ns value (which we denote as τ) to a single point mass. We denote the resulting distribution as $DFE_{f}(\alpha ,\beta )$.

The likelihood of having a certain distribution of identity by state lengths L given a demographic scenario D, a variant at a frequency f and 2 parameters α and β is equal to:

(3) $$L(\alpha ,\beta ,D,f|L\in w_{m_{j}})=\sum_{4Ns=0}^{\tau }P\left(L\in w_{m_{j}}|4Ns,f,D\right)P(4Ns|\alpha ,\beta )\;d4Ns$$

where $P\left(L\in w_{m_{j}}|4Ns,f,D\right)=$$L(4Ns,f,D|L\in w_{m_{j}})$ and was introduced in Equation (1). P(4Ns|α,β) is the probability of having that discrete value of 4Ns given a discretized gamma distribution with parameters α and β. That probability is equal to $F\left(4Ns+0.5|\alpha ,\beta \right)-F\left(\max(4Ns-0.5,\;0)|\alpha ,\beta \right)\;$for 4Ns values smaller than τ. $F\left(x|\alpha ,\beta \right)$ is the cumulative distribution function of a having a value x given a gamma distribution with parameters α and β. When 4Ns=τ, we use $1-F\left(\tau -0.5|\alpha ,\beta \right).$

We obtain an estimate of the α and β parameters by doing a grid search over a set of α and β candidate values. We find the combination of α and β parameters that maximize the composite likelihood function:

(4) $$L\left(\alpha ,\beta ,D,f|L\right)=\prod_{j=1}^{\ell }L(\alpha ,\beta ,f,D|L_{j}\in w_{m_{j}})$$

We tested the performance of our method using forward-in-time simulations. To do this, we generated 10,000 allele frequency trajectories $H_{k}$ using *PReFerSim* of an allele with a 1% frequency f in the present in a sample of 4,000 chromosomes. The simulations of $H_{k}$ were done under a combination of the 2 demographic models previously defined (constant population size model and a population expansion model) with 2 DFEs estimated in different species: 1 from humans (shape = 0.184; scale = 319.8626; *N* = 1,000) (Boyko *et al.* 2008) and another one from mice (shape = 0.11; scale = 8,636,364; *N* = 1,000,000) (Halligan *et al.* 2013). Then, we use 10,000 $H_{k}$ generated for each demographic scenario and DFE to obtain 100 simulation replicates with $\ell =2\times A\times \left(\begin{array}{c} n\\ 2 \end{array}\right)=2\times 300\times \left(\begin{array}{c} 40\\ 2 \end{array}\right)$ values of L using the algorithm shown in the section *Forward-in-time simulations to assess the impact of selection on the allele frequency trajectories, allele ages, pairwise coalescent times*$T_{2}$, *and*L*values*.

### Connecting the distribution of fitness effects of variants at a particular frequency (*DFE_f_*) with the distribution of fitness effects of new mutations (*DFE*)

The distribution of fitness effects of new mutations DFE can be broadly defined as a probability distribution that is a function f(ψ) dependent on κ parameters whose values are equal to $\psi =\{\psi_{1},\psi_{2},\psi_{3},\ldots ,\psi_{\kappa }\}$. The distribution of fitness effects of variants at a particular frequency $DFE_{f}$ in the population is related to the DFE determined by κ parameters $\psi =\{\psi_{1},\psi_{2},\psi_{3},\ldots ,\psi_{\kappa }\}$ by the following equation based on the Bayes’ theorem:

(5) $$P_{\psi }\left(f|s_{j},D\right)=\frac{P_{\psi }(s_{j}|f,D)\;P_{\psi }(f|D)}{P_{\psi }\left(s_{j}|D\right)}$$

where we can rearrange the above equation to obtain:

(6) $$P_{\psi }\left(s_{j}|D\right)=\frac{P_{\psi }(s_{j}|f,D)\;P_{\psi }(f|D)}{P_{\psi }(f|s_{j},D)}$$

where $s_{j}$ represents a continuous interval of 4Ns values $\left[4Ns_{j-1},4Ns_{j}\right)$ containing 4Ns values greater or equal than some value $“4Ns_{j-1}”$ and 4Ns values smaller than some value “$4Ns_{j}.$”

$P_{\psi }\left(s_{j}|D\right)$ defines the distribution of fitness effects of new mutations over a set of discrete bins when using the information contained across all nonoverlapping intervals **σ**=*{[*4Ns*_0_*, 4Ns*_1_), [*4Ns*_1_*, 4Ns*_2_), [*4Ns*_2_*, 4Ns*_3_)…, [*4Ns_*b*__−_*_1_*, 4Ns*_b_)} = {*$s_{1},s_{2},\;s_{3},\;\ldots ,s_{b}$*}* covering all 4Ns values from 0 to infinite. We assume that the values of 4Ns presented here represent deleterious or neutral mutations. The selection coefficients $s_{0},s_{1},\ldots s_{b}$ are ordered in an ascending order starting from $s_{0}=0$. We defined the endpoints of the first b-1 intervals to be equal to 5(i-1) and 5i, where i takes values from 1 to b-1. The last interval was set to be equal to [5(b-1),∞). Since $P_{\psi }\left(s_{j}|D\right)$ is independent of the demographic scenario D, then $P_{\psi }\left(s_{j}|D\right)=P_{\psi }\left(s_{j}\right)$ because D does not impact the proportion of new variants in a selection interval $s_{j}$. $P_{\psi }\left(s_{j}\right)$ defines the proportion of new mutations inside a $s_{j}$ interval (i.e. it is the DFE over a set of discrete intervals). With this equality, we rearrange Equation (6) to obtain:

(7) $$P_{\psi }\left(s_{j}|D\right)=P_{\psi }\left(s_{j}\right)=\frac{P_{\psi }(s_{j}|f,D)\;P_{\psi }(f|D)}{P_{\psi }(f|s_{j},D)}$$

We explain how to compute each of the probabilities $P_{\psi }(s_{j}|f,D)$, $P_{\psi }(f|D)$ and $P_{\psi }(f|s_{j},D)$ to estimate $P_{\psi }\left(s_{j}\right)$ in Appendix. $P_{\psi }\left(s_{j}|f,D\right)$ is the probability of having variants with a value of selection inside the interval $s_{j}$ given that the variants were sampled at a frequency f under the demographic scenario D and that the DFE follows a function f(ψ). $P_{\psi }(f|D)$ is the probability of having variants sampled at a frequency f given the demographic scenario D and that the DFE follows a function f(ψ). Finally, $P_{\psi }(f|s_{j},D)$ is the probability of having variants sampled at a frequency f given that the variants have a value of selection inside the interval $s_{j}$, we have a demographic scenario D and that the DFE follows a function f(ψ). $P_{\psi }(f|D)$ and $P_{\psi }(f|s_{j},D)$ can be computed via simulations (Appendix). We also tested Equation (7) using forward-in-time simulations with *PReFerSim* to calculate the probabilities $P_{\psi }(s_{j}|f,D)$, $P_{\psi }(f|D)$, and $P_{\psi }(f|s_{j},D)$ as explained in Appendix.

### ABC-based inference of the demographic scenario

All of the analysis described so far assume that the demographic scenario is known. There are different methods to perform demographic inferences based on different sources of data (Beichman *et al.* 2018) such as the SFS (Gutenkunst *et al.* 2009; Excoffier *et al.* 2013; Kamm *et al.* 2018) or the linked patterns of heterozygous and homozygous genotypes across the genome (Li and Durbin 2011; Schiffels and Durbin 2014). The demographic inferences performed using different sources of information are not concordant (Beichman *et al.* 2017) and this has motivated a discussion among the scientific community to generate datasets evolving under different demographic scenarios to test the inference performance of different available methods (Adrion *et al.* 2020). Therefore, the discussion on what summary statistics or data should be used for demographic inferences is still open. Given the discordances between the demographic inferences based on the data used, we decided to estimate the demographic scenario using the L values in a set of putatively neutral variants using an ABC approach (see Appendix). Our approach is to infer the demographic scenario on a set of putatively neutral variants using the L values to then evaluate the impact of selection on a set of variants where natural selection could be acting also employing the L values. We fix the demographic scenario and estimate the impact of natural selection in a set of putatively functional sites [as in Boyko *et al.* (2008) and Kim *et al.* (2017) where the same summary statistic is used to infer the demographic scenario and the DFE].

### Assessing the robustness of the method

We assessed the impact of multiple factors on the estimates of selection using our method. First, we analyzed the effect of ancestral state misidentification on our estimates of selection. We also tested the robustness of our method to biases in SNP and genotype calling in low frequency variants, haplotype phasing errors, mutation rate misspecification and recombination rate misspecification (see Appendix). We also tested the accuracy of our inference method to the use of a different number (4, 6, 11, 51, and 101) of nonoverlapping equidistant windows $W=\{w_{1},w_{2},\ldots ,\;w_{M}\}$ that extend to the side of the derived allele at a focal site. We also explored if our inferences could be improved using 2 different Monte–Carlo strategies to compute $P\left(L\in w_{j}|\;D,H_{k}\right)$ from Equation (1) that require (1) Simulating 200 sets of n haplotypes for each realization of $H_{k}$ (instead of 100 as we have done previously) to obtain $\ell =200\times \left(\begin{array}{c} n\\ 2 \end{array}\right)$ values of L for each $H_{k}$; (2) performing simulations where the focal allele is located on the center of the simulated haplotypes and we estimate $P\left(L\in w_{j}|\;D,H_{k}\right)$ by taking the distances going upstream and downstream of the focal allele site (instead of calculating the values of L only going downstream of the focal allele). We also explored if the use of a different identity by state statistic L′ that uses information from the upstream and downstream region of the focal allele could improve our inferences (see Appendix). Finally, we analyzed the performance of our method when each simulation set had 150 variants with a recombination rate equal to 0 and 150 variants had a recombination rate equal to $1\times 10^{-8}$. We also test a modification of our methodology to perform inferences when the 300 variants had variable recombination rates per base (see Appendix).

We also performed forward-in-time simulations using SLiM (Haller and Messer 2019) to analyze the impact of linked selection on our estimates of selection. We performed simulations that mimic the arrangement of exonic elements (Harrow *et al.* 2012), conserved noncoding elements (Siepel *et al.* 2005; Huber *et al.* 2017) and recombination rates (Kong *et al.* 2010) in the human genome. Our simulations were performed under a scaled population expansion demographic model (see Appendix).

### Application to the *UK10K* dataset

We inferred the distribution of fitness effects of the 1% ± 0.05% frequency variants at non-CpG nonsynonymous sites that are more than 5 Mb away from the centromere or telomeres in the phased *UK10K* haplotype reference panel. The panel was statistically phased with *Shapeit2* (Delaneau *et al.* 2013). We discarded a set of related individuals along with other individuals with no clear European ancestry from the haplotype panel, as previously defined (Walter *et al.* 2015). In the end, we obtained a sample size of 3,621 individuals (7,242 haplotypes) from the *UK10K* haplotype panel.

We estimated the proportion of exonic sites, *PhastCons* element sites (Siepel *et al.* 2005) and the average strength of background selection based on the *B* values (McVicker *et al.* 2009) in the 250-kb regions upstream and downstream of the focal non-CpG synonymous variants and nonsynonymous variants to assess if there were differences on the proportion of those functional elements that might suggest a differing effect of background selection surrounding those 2 categories of variants.

We used an *ABC* algorithm to infer the demographic scenario that explains the distribution of L for the 142 non-CpG synonymous variants at a 1% ± 0.05% frequency that are more than 5 Mb away from the centromere or telomeres (see Appendix). CpG sites were removed before estimating *L* around the non-CpG synonymous sites following (McVicker *et al.* 2009). For computational efficiency, in the ABC method we scaled the population size down by a factor of 5 while increasing the mutation rate μ, selection coefficient *s* and recombination rate *r* by the same factor of 5 to keep 4Ns, θ=4Nu and ρ=4Nr constant. That same scaling was used in all the simulations described in this section and in our inference of selection in the *UK10K* data. We will refer to the inferred scaled model as the “scaled *UK10K* model” and we will refer to the model without the scaling as the “*UK10K* model.” We performed forward-in-time simulations under the “scaled *UK10K* model” to understand how changes in the 4Ns values impact the allele frequency trajectories, allele ages, pairwise coalescent times $T_{2}$ and *L* values. The inferred demographic model was used to perform the inferences of selection in the nonsynonymous sites.

We performed simulations to analyze if the amount of information present in the *UK10K* dataset was sufficient to infer selection coefficients in 1%±0.05% frequency variants under the “scaled *UK10K* model.” Our approach takes into account the differences in recombination rates on the regions surrounding each variant on the genome in the *UK10K* data (see Appendix). We performed 100 simulation replicates, where each replicate mimics the amount of information present in the *UK10K* dataset. We assessed the impact of ancestry misspecification, phasing, mutation rate misidentification, and recombination rate misspecification. We also tested how well our method could infer the DFE with simulations performed under the Boyko distribution of fitness effects with the “*UK10K* model” and the “scaled *UK10K* model.”

Finally, we used the L values in the *UK10K* dataset to infer a point 4Ns value for the nonsynonymous and synonymous 1%±0.05% frequency variants. CpG sites were removed before estimating *L* around the non-CpG nonsynonymous sites (McVicker *et al.* 2009). Then we applied our method to infer the DFE of the nonsynonymous variants and in 100 bootstrap replicates (Appendix). We also provide information on how we estimated $P_{\psi }(f|D)$ in the Appendix.

## Results

### Evaluation of population-scaled selection coefficient inference for constant population sizes

We investigated the performance of our method to estimate 4Ns values using forward-in-time simulations. Specifically, we used *PReFerSim* (Ortega-Del Vecchyo *et al.* 2016) to obtain 10,000 allele frequency trajectories for an allele with a present-day sample allele frequency of f = 1% (n = 40 chromosomes with the derived allele in a sample of 4,000 chromosomes) for 5 different values of selection (4Ns = 0, −50, −100, 50, 100).

**Fig. 2.**
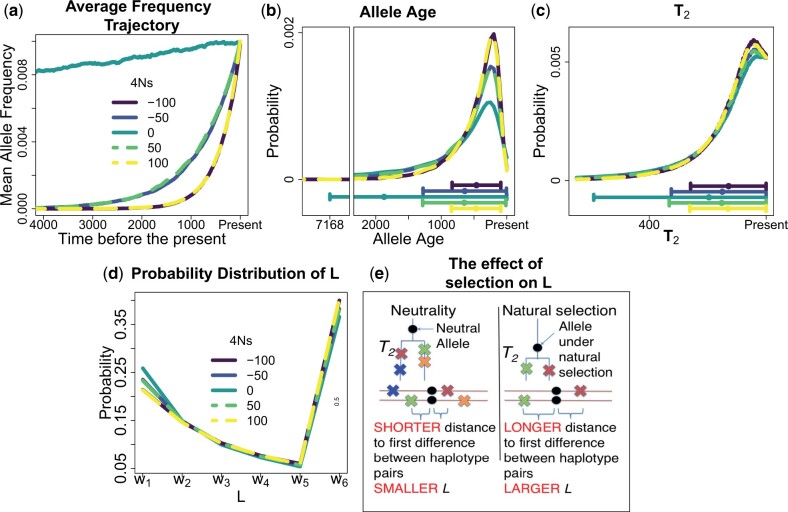
Properties of alleles sampled at a present-day frequency f=1% under different strengths of natural selection in a constant size population (*N* = 10,000). We obtained 10,000 frequency trajectories for f = 1% frequency alleles under different strengths of selection using forward-in-time simulations under the *PRF* model. We used those frequency trajectories to calculate: a) the mean allele frequency at different times in the past, in units of generations, to obtain an average frequency trajectory; b) the probability distribution of allele ages; c) the probability distribution of pairwise coalescent times $T_{2}$. Below b) and c), we show a dot with 2 whiskers extending at both sides of the dot. The dot represents the mean value of the distribution and the 2 whiskers extend 1 SD below or above the mean. The whisker that extends 1 SD below the mean is constrained to extend until max(mean—SD, 0). d) Probability distribution of $P(L\in w_{i}|4Ns,f,D)$. We define L by taking the physical distance in basepairs next to the focal allele across 5 nonoverlapping equidistant windows of 50 kb, with an extra window $w_{6}$ indicating that there are no differences in the 250-kb next to the allele. L is calculated both upstream and downstream of the focal allele and uses A = 30,000 independent sites with 40 haplotypes containing the derived allele in each site to get $l=2\;\times \;30,000\;\times \left(\begin{array}{c} 40\\ 2 \end{array}\right)=46,800,000$ values of L. In this demographic scenario, the alleles under a higher absolute strength of selection 4Ns have younger ages and younger $T_{2}$ on average. The fact that alleles under higher absolute strengths of selection have younger average $T_{2}$ values implies that those alleles tend to have larger L values as shown in d) and e). e) Impact of natural selection on the values of L due to the effect of natural selection on the values of $T_{2}$.

Using the 10,000 recorded allele frequency trajectories for each selection value 4Ns, we calculated the mean allele frequency across many generations going backwards into the past to obtain an average frequency trajectory for 1% frequency alleles (Fig. 2a). As expected (Maruyama 1974), the average allele frequency for neutral alleles (4Ns = 0) is higher for a longer duration going backwards in time compared with alleles under natural selection. Furthermore, alleles under the same absolute strength of selection have the same average allele frequency trajectory, regardless of whether the allele is under positive or negative selection. The distribution of ages is shifted toward younger values for higher absolute values of 4Ns and with increasingly smaller standard deviation (Fig. 2b), and Maruyama’s theoretical results accurately predict the mean age estimates observed in the simulations (Supplementary Table 2).

We computed the distribution of pairwise coalescent times $T_{2}\;$analytically across different values of 4Ns using the 10,000 allele frequency trajectories. We found that alleles under higher absolute values of 4Ns have a more recent average value of $T_{2}$, and their distribution of $T_{2}$ has a smaller standard deviation (Fig. 2c). We calculated the distribution of L for each 4Ns value using simulations assuming a constant population-scaled recombination rate ρ=4Nrl=200 and a constant population-scaled mutation rate θ=4Nul = 240 for a region of *l* = 500 kb with a per-generation mutation rate $u=1.2\;\times \;10^{-8}$ and a per-generation recombination rate $=1\;\times \;10^{-8}$. The focal site with the inspected derived allele is located in the center of the simulated haplotypes. We found that alleles under the same absolute strength of selection have almost identical distributions of L (Fig. 2d). The results in Fig. 2c-d are in line with the fact that $T_{2}$ is younger in alleles under stronger selection coefficients, implying that there will be fewer mutations between haplotypes sharing the allele and, therefore, higher average values of L (Fig. 2e).

**Fig. 3.**
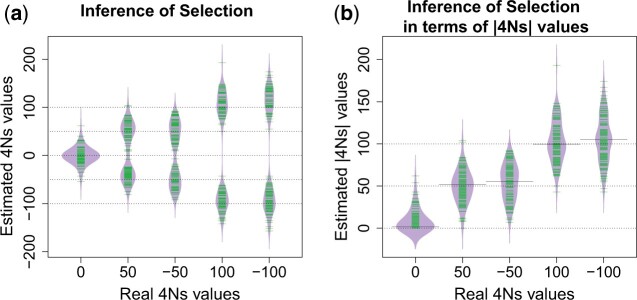
Estimation of the strength of natural selection in a constant population size model using $\ell =2\times \;300\times \left(\begin{array}{c} 40\\ 2 \end{array}\right)=468,000$ realized values of L for each simulation replicate. Each simulation replicate contained 300 independent 1% frequency variants, where each variant had 40 haplotypes with the derived allele. a) Estimated selection values. b) Estimated selection magnitudes (absolute values of s). “Real 4Ns values” refers to the 4Ns values used in the simulations, while “Estimated 4Ns values” refers to the values estimated by our method. The dashed lines are placed on values that match 4Ns values used in the simulations. The median value of the estimates of 4Ns is shown with a solid line. The green lines in a) and b) indicate estimated values of 4Ns, where there are 100 estimated values in each of the for the 5 4Ns values inspected. Each estimated 4Ns value uses $l=2\;\times \;300\;\times \left(\begin{array}{c} 40\\ 2 \end{array}\right)=468,000$ values of L.

We used the simulations to test our method’s ability to estimate the strength of selection in this constant-size population history. For each simulation replicate we used the values of L obtained by simulating A = 300 independent loci with a 1% frequency variant. We sample n = 40 chromosomes with the derived allele in a sample of 4,000 chromosomes in each variant to get a total number $\ell =2\times \;A\times \left(\begin{array}{c} n\\ 2 \end{array}\right)=2\times \;300\times \left(\begin{array}{c} 40\\ 2 \end{array}\right)=468,000$ of values of L. This number of 1% frequency variants is similar to the number of nonsynonymous variants found in the *UK10K* dataset, which is 275. We found that for alleles where, for instance 4Ns is −50, the estimated values of selection tend to be equally distributed around values of −50 or 50 (Fig. 3a). A similar result is seen for the 4Ns values equal to 100 (Fig. 3a). When we display the estimated absolute value of the strength of selection, we see that our method produces nearly unbiased estimates (Fig. 3b). These results show that in a constant size population our method provides accurate estimates of the absolute strength of natural selection, but cannot infer the sign of the selection coefficient.

Additionally, we decided to analyze 1% frequency variants throughout all the manuscript based on the results from Fig. 3. We took this decision based on the accuracy of our results to infer the strength of natural selection given our simulations where we have a similar number of independent 1% frequency variants A to what is observed in the *UK10K* dataset. Maruyama (1974) observed an approximate 8-fold difference in the allele age between neutral alleles and alleles with a 4Ns=100. We hypothesized that this difference is sufficient to drive changes on the values of L (Fig. 2) that are informative of the strength of natural selection as shown in Fig. 3.

### Evaluation of inference performance for nonequilibrium demographic scenarios

**Fig. 4.**
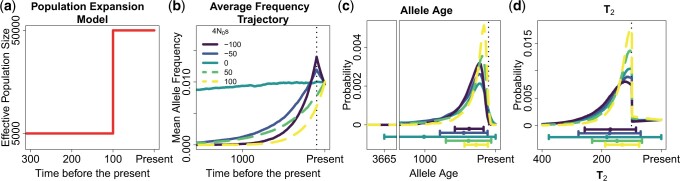
Properties of alleles sampled at a 1% frequency under different strengths of selection in a population expansion scenario. a) Population expansion model analyzed. b) Mean allele frequency at different times in the past, in units of generations, using 10,000 allele frequency trajectories. Note that alleles under the same absolute strength of selection (4Ns) have very different average allele frequency trajectories, in contrast to the constant population size scenario (Fig 2); c) probability distribution of allele ages and d) probability distribution of pairwise coalescent times $T_{2}$. The dot and whiskers below c) and d) represent the mean value of the distribution and the 2 whiskers extend at both sides of the mean until max(mean ± SD, 0).

Following our analysis for constant-size populations, we next analyzed the shape of the average allele frequency trajectory in a population expansion scenario (Fig. 4a) for 1% frequency alleles with different 4Ns values. The 4Ns values are calculated with respect to the population before the expansion. All the 4Ns values have a 10-fold increase after the population expansion taking place 100 generations before the present. Unlike in the constant population size scenario, we found distinct average allele frequency trajectories for alleles under positive or negative selection (Fig. 4b): alleles under positive selection on average had increased in frequency moving forward in time, while alleles under negative selection on average had increased in frequency before the expansion and then decreased after the expansion due to the increased selection efficacy in the large population. The values of 4Ns increase 10-fold after the population expansion leading to a higher efficacy of natural selection compared with drift driving frequency differences in alleles under natural selection. The ages of alleles under the strongest absolute values of selection tend to be younger, and alleles with the same |4Ns| value but different 4Ns value differ in the mean and standard deviation of their allele ages (Fig. 4c). The distributions of pairwise coalescent times for allele carriers show concordant patterns (Fig. 4d) and alleles under the stronger positive selection had, on average, younger $T_{2}$ values than negatively selected alleles of the same magnitude. Further, when we contrasted the $T_{2}$ distribution of the negatively selected alleles inspected (4Ns = −50, −100), we saw that their mean $T_{2}$ value did not differ much, and their biggest difference was due to a slightly smaller standard deviation in the most deleterious allele (Fig. 4d).

**Fig. 5.**
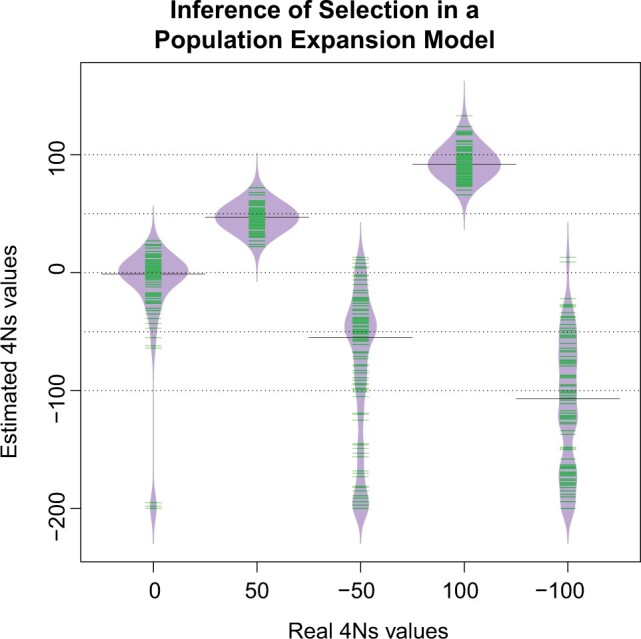
Estimation of the strength of natural selection in a population expansion model for 1% frequency alleles. Each simulation replicate contained $2\;\times \;A\;\times \left(\begin{array}{c} n\\ 2 \end{array}\right)=2\;\times \;300\;\times \left(\begin{array}{c} 40\\ 2 \end{array}\right)=468,000\;$realized values of L. The green lines indicate 1 estimated value of 4Ns. “Real 4Ns values” indicate the 4Ns values used in the simulations and “Estimated 4Ns values” refers to the values estimated by our method. The median value of the estimates of 4Ns is shown with a solid line.

We next used our method to infer the strength of selection for this expansion scenario and found that it can provide approximately unbiased estimates of the sign and strength of selection (Fig. 5). We saw a wider distribution of the 4Ns estimates for deleterious variants compared with advantageous variants. This can be explained by comparing the $P(L\in w_{j}|4Ns,f,D)$ distribution for a set of focal 4Ns values with the distribution $P(L\in w_{j}|4Ns,f,D)$ of other 4Ns values. We see that the distribution $P(L\in w_{j}|4Ns,f,D)$ does not vary much between deleterious variants compared with advantageous variants (Supplementary Fig. 1). The highest similarity of the distribution $P(L\in w_{j}|4Ns,f,D)$ for the deleterious variants produces 4Ns estimates with a higher variance than the advantageous variants since the data used by our inference method relies in the differences on the $P(L\in w_{j}|4Ns,f,D)$ distribution. More differences on the $P(L\in w_{j}|4Ns,f,D)$ distribution for different 4Ns lead to estimates with a smaller variance. We got a few very deleterious 4Ns estimates for the neutral variants, which is consistent with the highest similarity of $P(L\in w_{j}|4Ns=0,f,D)$ and the distribution $P(L\in w_{j}|4Ns,f,D)$ of very deleterious variants in contrast with the distribution $P(L\in w_{j}|4Ns,f,D)$ of very advantageous variants (Supplementary Fig. 1). Despite these differences on the distribution of the 4Ns estimates for deleterious and advantageous variants, our 4Ns estimates appear to be approximately unbiased based on the median of the 4Ns estimates for neutral, advantageous and deleterious variants (Fig. 5).

The inference results under a population expansion model do not imply that we can differentiate between positive and negative selection in all nonequilibrium models. The power to do so will be dependent on the parameters of the nonequilibrium demography being studied. As an example, in an ancient bottleneck scenario we find there are no significant differences in the distribution of $T_{2}$ between alleles that have the same absolute strength of selection, indicating that we would not be able to differentiate between alleles under positive or negative selection under this demographic model (Supplementary Fig. 2).

We also evaluated the performance of our method when the population expansion time took place at more ancient times of 1,000, 10,000, and 100,000 generations ago. Interestingly, we found that our method provided 4Ns estimates that appear unbiased under a population expansion that took place 1,000 generations ago. When the population expansion takes place 10,000 and 100,000 generations ago our method only provides nearly unbiased estimates of |4Ns| but not 4Ns as in the estimates for the constant population size scenario shown in Fig. 3 (see Supplementary Fig. 3 for a more detailed explanation).

We also tested our method under 2 complex human demographic models that show the history of Africans (Tennessen *et al*. 2012) and the YRI population (Schiffels and Durbin 2014). We saw that our method produced nearly unbiased estimates of selection for neutral and advantageous variants (Supplementary Fig. 4). On the other hand, we had a slight overestimation for the 4Ns values in the case of deleterious alleles. However, the real estimate was always contained inside the 25th and 75th percentile of the distribution of estimated values (Supplementary Fig. 4).

### Testing the inference of the distribution of fitness effects for variants found at a particular frequency (“$DFE_{f}$”)

We tested if the distribution of haplotype lengths L can be used to estimate the parameters that define the distribution of fitness effects of variants at a particular frequency using Equation (4). We used distributions of $\ell =2\times \;A\times \left(\begin{array}{c} n\\ 2 \end{array}\right)=2\;\times \;300\;\times \left(\begin{array}{c} 40\\ 2 \end{array}\right)=468,000$L values from 1% frequency alleles in a sample of 4,000 chromosomes obtained via simulations under the constant population size and population expansion demographic model from the past sections under 2 distributions of fitness effect of new mutations estimated in different species: one from humans (shape = 0.184; scale = 319.8626; *N* = 1,000) (Boyko *et al.* 2008) and another one from mice (shape = 0.11; scale = 8,636,364; *N* = 1,000,000) (Halligan *et al.* 2013).

**Fig. 6.**
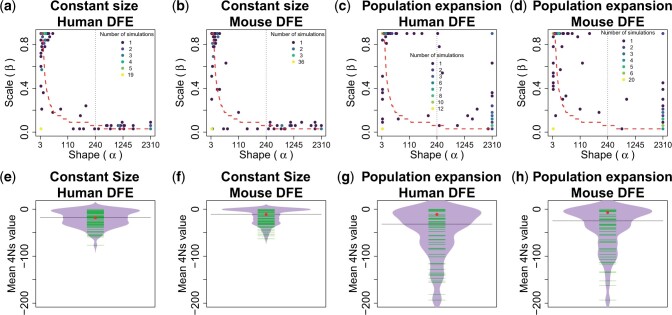
MLEs of the parameters that define the distribution of fitness effect for variants at a 1% frequency. a–d) We tested if our method was capable of estimating the parameters of the $DFE_{f}$ of variants at a particular frequency in 2 demographic models and 2 DFEs. The shape (α) and scale (β) parameters define the compound $DFE_{f}$ distribution using τ=200 in Equation (3). Each simulation replicate contained $2\;\times \;A\;\times \left(\begin{array}{c} n\\ 2 \end{array}\right)=2\;\times \;300\;\times \left(\begin{array}{c} 40\\ 2 \end{array}\right)=468,000\;$realized values of L. The number of simulation replicates estimated to have a particular combination of α and β parameters is shown with a different color in each plot. The dotted red line represents a combination of shape and scale parameters from the partially collapsed gamma distribution that gives a similar mean 4Ns value to the mean 4Ns value of the underlying $DFE_{f}$. The grid of scale parameters explored goes from (0.03, 0.06, …, 0.9) and the grid of shape parameters explored goes from (3, 6, …, 210) and then there is a change in the grid of shape parameters explored, specified by the dotted line, and the grid takes values from (240, 270, …, 2,310). e–h) The beanplots show the distribution of the estimated mean 4Ns values based on the $DFE_{f}$ estimated on the 100 simulation replicates. The red dots show the actual mean 4Ns value in 50,000 1% frequency variants simulated using each particular DFE and demographic model D. The green lines indicate estimated values of 4Ns across simulation replicates based on the $DFE_{f}$ estimates. The median value of the estimates of 4Ns is shown with a solid line.

We found that the estimated parameters of the shape (α) and scale (β) on single replicates of the ${\mathrm{DFE}}_{f}$ have considerable variation (Fig. 6, a and b). However, the estimated shape and scale of the ${\mathrm{DFE}}_{f}$ tend to imply the correct mean value of the ${\mathrm{DFE}}_{f}$ on average, showing that the shape (α) and scale (β) are correctly estimated as a product of those 2 parameters (estimates lie on average approximately close to the red-dashed lines in Fig. 6). This can be better seen in Fig. 6, e–h. We found that the estimated ${\mathrm{DFE}}_{f}$ parameters on constant population sizes define a ${\mathrm{DFE}}_{f}$ with a mean 4Ns value that, on average, is almost equal to the mean 4Ns value found across 50,000 simulated 1% frequency variants. In a population expansion scenario (Fig. 6, c and d), the estimated ${\mathrm{DFE}}_{f}$ parameters imply a ${\mathrm{DFE}}_{f}$ with a mean 4Ns value that is slightly lower than the actual mean 4Ns value, and with considerably higher variance in the estimated mean (Fig. 6, e–h).

### Testing the inference of the distribution of fitness effects of new mutations *DFE from the distribution of fitness effects of variants at a particular frequency (DFE_f_)*

**Fig. 7.**
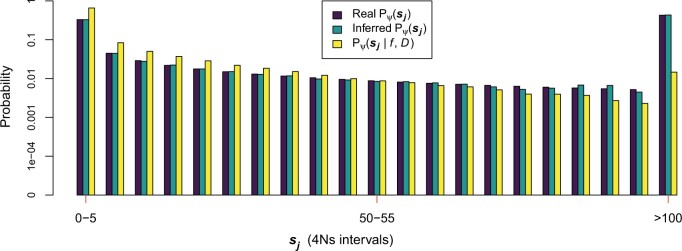
Inference of the distribution of fitness effects of new mutations from the distribution of fitness effects of variants at a certain frequency in deleterious variants. The DFE follows a gamma distribution with shape and scale parameters equal to 0.184 and 1599.313, respectively. This is equal to the gamma distribution inferred by Boyko *et al.* (2008) after adjusting the population sizes to the population expansion model used (Fig. 4a). “Real $P_{\psi_{}}\left(s_{j}\right)$” refers to the probability of having a 4Ns value in a certain interval $s_{j}$ given the distribution of fitness effects of new mutations with parameters ${}_{\psi }$. “$P_{\psi_{}}(s_{j}|f,D)$” is the probability of having an 4Ns value in an interval $s_{j}$ given the distribution of fitness effects DFE with parameters ${}_{\psi }$ and the demographic scenario D in f = 1% frequency variants. We calculated $P_{\psi_{}}(s_{j}|f,D)$ from a set of 62,412 4Ns 1% frequency variants obtained via forward-in-time *PReFerSim* simulations under the Boyko *et al.* (2008)DFE and the population expansion scenario. “Inferred $P_{\psi }\left(s_{j}\right)$” is an estimate of the probability of having a 4Ns value in a certain interval $s_{j}$ given the distribution of fitness effects of new mutations with parameters ${}_{\psi }$. This estimate is calculated using $P_{\psi_{}}(s_{j}|f,D)$, $P_{\psi_{}}(f|D)$, $P_{\psi_{}}(f|s_{j},D)$ and Equation (7) (see Appendix). The selection coefficient s refers exclusively to the action of deleterious variants in this plot.

We estimated the distribution of fitness effects of new mutations, i.e. the DFE, in a population expansion scenario given the distribution of fitness effects $DFE_{f}$ from a set of simulated variants at a 1% frequency (Fig. 7—Boyko Human DFE;Supplementary Fig. 5—Human DFE with a scale value that is 20 times smaller). We see that the inferred and real $P_{\psi }\left(s_{j}\right)$ values match using Equation (7), with some slight discrepancies that could be due to either using a $s_{j}$ bin that is not small enough or small inaccuracies in the estimated probabilities of $P_{\psi }(s_{j}|f,D),\;P_{\psi }(f|D)$, or $P_{\psi }(f|s_{j},D)$. We also note that variants at a 1% frequency tend to be less deleterious compared with new variants based on the comparison of the distributions $P_{\psi }(s_{j}|f,D)$ against $P_{\psi }\left(s_{j}\right)$. We also find that the estimates of $P_{\psi }\left(s_{j}\right)$ under the constant population size and the population expansion model do not depend on the number of generations simulated for the most ancient epoch of both models, as long as the simulated generation number is large enough (>10*N* generations) for the most ancient epoch to achieve mutation–selection balance (Supplementary Figs. 6, 7 and Tables 3 and 4).

We used our $DFE_{f}$ estimates from Fig. 6 to estimate $P_{\psi }\left(s_{j}\right)$. The $P_{\psi }\left(s_{j}\right)$ estimates appear to be unbiased, but display a larger variance under the population expansion scenario compared with the constant size scenario (Supplementary Fig. 8). We also compared our estimates of the DFE with the inferences obtained using *fitDadi* (Kim *et al.* 2017). We generated data from the SFS using *PReFerSim* that contained 300–500 1% variants in our comparisons and we found that *fitDadi* gave very accurate estimates of the DFE under a constant population size, and had very slight biases under the population expansion model. On the other hand, our method always contained the correct proportion of $P_{\psi }(s_{j})$ inside the inferred 5% and 95% quantile of the estimated $P_{\psi }\left(s_{j}\right)$ values but had a larger variance on the estimates compared with the estimates obtained with *fitDadi* (Supplementary Fig. 8). Interestingly, only using 300–500 SNPs to perform inferences with *fitDadi* gave biased results compared with the more accurate results from our method using data from 300 1% frequency variants (Supplementary Fig. 8).

### ABC-based inference of the demographic scenario

All of our past analyses assume that the demographic scenario is known. To address the more challenging scenario where demography is unknown, we developed an *ABC* approach to infer the demographic scenario using a set of L values that have the same frequency f as the putative alleles under natural selection analyzed (see Appendix). We found that this approach gave reliable estimates of the effective population size in the constant population size scenario (Supplementary Fig. 9). The approach also provided accurate estimates of all the parameters in a population expansion scenario, with just a 9.5% overestimation of the population expansion time (Supplementary Fig. 10). Our *ABC* approach provided similarly accurate estimates of the demographic parameters when the recombination rates on the haplotypes surrounding the variants were different under a population expansion scenario (Supplementary Fig. 11) compared with a scenario where all the variants had the same recombination rate (Supplementary Fig. 10).

### Assessing the robustness of the method

We assessed the impact of multiple factors on the estimates of selection using our method (see Appendix for a more complete description of all the described tests). First, we analyzed the effect of ancestral state misidentification on our estimates of selection, since some of the 1% frequency focal alleles we inspected in the analyses of the previous sections could be ancestral alleles instead of derived alleles. We found that the ancestral state of deleterious alleles was not misspecified under the constant population size model and the population expansion model in simulations including more than 5,000 alleles with a 1% minor allele frequency. Neutral and advantageous alleles with a 1% minor allele frequency could be wrongly assigned a derived status with probabilities ranging from 0.014% to 1.18% (Supplementary Table 5, also see Appendix). However, we found that this level of ancestral state misspecification did not cause biases on the estimation of 4Ns for neutral alleles and the strength of selection for advantageous alleles was slightly underestimated (Supplementary Fig. 12).

Biases in SNP and genotype calling are another source of concern for the application of our method. Those biases can cause a decrease or an increase on the number of called rare variants depending on the pipeline used to do the SNP and genotype calling. The impact of these biases is more dramatic for singleton variants than other higher frequency variants (Han *et al.* 2014). One way to mitigate these effects if to perform the calculations of L and the estimation of selection without taking into account very low frequency variants. We tested this idea by estimating L in simulations after masking variants that only appear once in each set of haplotypes with the derived variant. Those variants were also masked before we performed the calculation of L(4Ns,f,D|L). We obtained accurate estimates of selection after performing this procedure (Supplementary Fig. 13) indicating that the masking of low-frequency alleles that could be incorrectly called does not bias the estimates of selection.

We next analyzed the impact of haplotype phasing errors due to the use of statistical phasing software in estimates of selection using our method. We statistically phased simulated haplotypes using *ShapeIt2*, which was also used to phase the *UK10K* dataset, and we estimated L from those phased haplotypes. We found that the estimates of L are not greatly biased by the use of the statistical phasing software (Supplementary Fig. 14). We also found that the haplotype phasing errors affect the variance of the estimates of selection but overall do not cause biased estimates of selection in any particular direction (Supplementary Fig. 15).

We also explored the impact of recombination rate misspecification and mutation rate mis-specification in our estimates of selection. To do this, we performed simulations where the values of ρ or θ were higher or smaller than the values used to calculate $L(4Ns,f,D|L\in w_{m_{j}})$ and then perform the inferences using Equation (2). We found an inverse relationship between the estimated |4Ns| values and the ρ or θ values used in the simulations under a constant population size demographic model (Supplementary Fig. 16). We saw broadly the same trend when analyzing variants under a population expansion model (Supplementary Fig. 17). We only obtained accurate estimates of the |4Ns| values when the values of ρ or θ used in the simulations were similar to the values used in the estimation of $L(4Ns,f,D|L\in w_{m_{j}})$ under the 2 demographic models analyzed.

Our previous analysis used 5 nonoverlapping equidistant windows at the left side of the inspected allele plus an extra window denoting that there are no differences in the 5 defined windows. We analyzed the impact of using a different number of windows (4, 6, 11, 51, and 101) given we analyze the same number of base pairs surrounding the focal allele (Supplementary Fig. 18). Compared with using 6 windows, the median estimated value of selection across the 100 simulations did not improve by more than 7 units in the analyzed demographic models and values of selection (Supplementary Fig. 18). The RMSE did not improve by more than 25 in the constant population size and population expansion model for 5 different values of selection compared with the analysis done with 6 windows (Supplementary Fig. 18). Using a larger number of windows increases the memory required to compute the likelihood equation shown in Equation (1). The number of values that need to be stored to compute the likelihood equation is equal to the number of allele frequency trajectories $H_{k}$, which we set equal to 100,000 throughout all our analysis, times the number of windows. We opted to use a modest number of windows equal to 6 to avoid storing a very large set of numbers to compute Equation (1).

Our estimates using Equation (1) use a Monte–Carlo strategy to compute $P\left(L\in w_{j}|\;D,H_{k}\right)$ where we simulate 100 sets of haplotypes for each $H_{k}$ that contain the focal allele in the left end of the simulated haplotyped. Then we compute $\left(\begin{array}{c} n\\ 2 \end{array}\right)$ values of L for each simulated set of haplotypes to obtain $\ell =100\times \left(\begin{array}{c} n\\ 2 \end{array}\right)$ values of L for each $H_{k}$. We explored if our inferences could be improved by using 2 alternative Monte–Carlo strategies: (1) 1 where we simulate 200 sets of haplotypes to obtain $\ell =200\times \left(\begin{array}{c} n\\ 2 \end{array}\right)$ values of L for each $H_{k}$ and (2) another 1 where the focal allele is located on the center of the simulated haplotypes and we estimate $P\left(L\in w_{j}|\;D,H_{k}\right)$ by taking the distances going upstream and downstream of the focal allele site. We saw no significant improvement in our estimates by using the 2 alternative Monte–Carlo strategies based on not having a decrease bigger than 5 on the root mean square error of our 4*Ns* estimates and that the median of our 4*Ns* estimates across 100 simulation replicates did not improve by a value bigger than 3 (Supplementary Fig. 19). Additionally, the 2 alternative strategies require us to double the computing time required to perform the Monte–Carlo simulations since we are simulating either twice the set of simulated haplotypes or a haplotype region that is 2 times bigger. In the case of the constant population size this increases the fully parallelizable computing time to obtain Equation (1) from approximately 100 to 200 h using an Intel E5-2680v4 @ 2.4 GHz CPU. Therefore, we decided to maintain the Monte–Carlo strategy where we simulate $\ell =100\;\times \left(\begin{array}{c} n\\ 2 \end{array}\right)$ values of L for each $H_{k}$ with the focal allele located in the left end for all the analysis presented in the manuscript.

We also added one additional analysis where we compare our estimates of 4*Ns* taking: (1) the $\ell =2\times \;A\times \left(\begin{array}{c} n\\ 2 \end{array}\right)\;$values of L as detailed in Fig. 1; and (2) taking the information of the upstream and downstream distances to construct a single statistic *L*′ that measures pairwise identity by state lengths to obtain $\ell =A\times \left(\begin{array}{c} n\\ 2 \end{array}\right)$ values of *L*′ (Supplementary Fig. 20). We also did not observe a significant difference by building a pairwise identity by state statistic that jointly takes information from the upstream and downstream region of the focal allele. The root mean square error did not improve by more than 15 units and the median of our 4*Ns* estimates across 100 simulation replicates was not improved by a value bigger than 6 over 5 different values of selection in the constant population size and population expansion scenario (Supplementary Fig. 20). The computational times to compute Equation (1) doubles when using a pairwise identity by state statistic that takes information from the upstream and downstream region of the focal allele and does not lead to great increases in accuracy. Due to those 2 reasons, we decided to stay with the simple statistic depicted in Fig. 1.

We saw that our method provided approximately unbiased estimates of selection when r=0 in 150 variants and $r=1\;\times \;10^{-8}$ in the other 150 variants in each simulation replicate of 300 variants under a population expansion scenario (Supplementary Fig. 21). We also obtained approximately unbiased results using a methodology to estimate either a fixed selection coefficient (Supplementary Fig. 22) or the Boyko distribution of fitness effects (Supplementary Fig. 23) under a population expansion model when each simulation replicate had 300 variants where each variant had a recombination rate sampled from the distribution of recombination rates seen on the 275 1% ±0.05% frequency nonsynonymous variants of the *UK10K* dataset. The estimates obtained when the variants had variable recombination rates had a similar accuracy to those seen when the variants had the same recombination rate (Figs. 5 and 6; Supplementary Fig. 8).

Finally, we evaluated the impact of linked selection in our estimates of selection. To do this, we performed forward-in-time simulations of 20 Mb regions under the recombination rate and arrangement of functional elements seen in the human genome. We performed simulations where the nonsynonymous mutations had a 4Ns value equal to 0, −50, or −100, or the DFE of those variants was distributed as the human DFE. First we inferred the demographic scenario using the L values from 1% frequency synonymous variants present in our data. Then, we inferred the value of selection using the L values from 1% nonsynonymous variants. We found that neutral alleles were accurately estimated as neutral. The median 4Ns estimate for simulations performed with a 4Ns value equal to −50 and −100 was equal to −25 and −200, respectively (Supplementary Fig. 24; also see Appendix). The true estimate of 4Ns was always contained inside the 25th and 75th percentile of the distribution of estimated values for the 3-point 4Ns values inspected. However, we caution that, based on the inferred demographic scenario, sometimes the inferred estimates of selection displayed a large variance and could show a bimodal distribution of estimated selection coefficients (see Supplementary Fig. 24 for a more detailed explanation) as shown in other demographic scenarios (Supplementary Fig. 3). Additionally, depending on the inferred demographic scenario sometimes the neutral variants were not accurately predicted as neutral and for the deleterious variants we observed that the median estimate of 4Ns span the edges of the grid of inspected 4Ns values taking a value of −200 or 0 depending on the demographic scenario. Therefore, the inferences of selection on these simulations display a large variance and are dependent on the demographic scenario inferred.

We also report the estimates of the scale and shape parameters of the $DFE_{f}$ in 10 simulation replicates (Supplementary Fig. 25). Based on these estimates, we performed estimates of the DFE (Supplementary Fig. 26) based on the inferred scale and shape parameters. The estimated proportion of 4Ns values between 0–5 and 5–100 was underestimated. On the other hand, the proportion of 4Ns values bigger than 100 was overestimated. However, the true proportion of 4Ns values in the intervals 0–5, 5–100, and bigger than 100 was always contained inside the 10th and 90th percentile of the distribution of estimated values (Supplementary Fig. 27).

### Application: inference of the distribution of fitness effects of 1% frequency variants in the UK10K dataset

For this analysis, we used variants that were at 1% frequency (±0.05%) and excluded CpG sites, and sites within 5 Mb from the centromeres or telomeres. We refer to these as the “focal” variants. First, we estimated the demographic scenario that best explains the distribution of L for the 142 focal synonymous variants using an *ABC* algorithm (see Appendix; Supplementary Fig. 28).

Before interpreting the results, we investigated whether linked selection may be of similar strength for the 2 variant sets. We find that in the upstream and downstream 250 kb regions surrounding the 142 synonymous 1% frequency variants and the 275 nonsynonymous 1% frequency sites there is a similar proportion of exonic sites (Mann–Whitney *U* test *P*-value = 0.7677), PhastCons element sites (Mann–Whitney *U* test *P*-value = 0.601), and the average strength of background selection (Mann–Whitney *U* test *P*-value = 0.9116) based on the *B* values (McVicker *et al.* 2009). This result suggests that the demographic model we inferred for the synonymous variants can be used to model the evolution of the nonsynonymous variants since the reduction in genetic variation due to background selection is similar on the haplotypes surrounding both types of variants (Supplementary Fig. 29). The approach of inferring the demographic model using synonymous sites is also used in analyses that infer the DFE using the SFS to help control the effects of background selection (Boyko *et al.* 2008; Huber *et al.* 2017; Kim *et al.* 2017; Tataru *et al.* 2017).

**Fig. 8.**
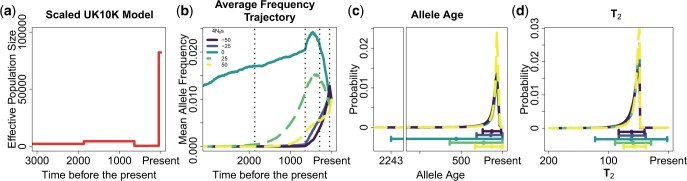
Properties of alleles sampled at a 1% frequency under different strengths of natural selection in the scaled *UK10K* model inferred in the *UK10K* data. a) Population model inferred in the *UK10K* dataset. b) Mean allele frequency at different times in the past, in units of generations. c) Probability distribution of allele ages and d) probability distribution of pairwise coalescent times $T_{2}$. The dot and whiskers below c) and d) represent the mean value of the distribution and the 2 whiskers extend at both sides of the mean until max (mean ± SD, 0).

To assess power and robustness, we performed simulations under the scaled *UK10K* demographic model inferred from the *ABC* algorithm. We found that the frequency trajectories and allele ages are significantly different between alleles under different strengths of selection (Fig. 8). However, the distribution of $T_{2}$ values is very similar for deleterious alleles that experience up to a 2-fold difference in the amount of selection acting upon them. This is important to note since the distribution of $T_{2}$ values is one of the most important factors, along with the mutation and recombination rate, determining the resolution of our approach to infer selection.

We also performed simulations to analyze if the amount of information present in the *UK10K* dataset was sufficient to infer selection coefficients in 1% frequency variants. Our approach takes into account the differences in recombination rates on the regions surrounding each variant on the genome in the *UK10K* data (Appendix). We performed 100 simulation replicates, where each replicate mimics the amount of information present in the *UK10K* dataset. Each replicate contains 275 independent loci with 69–76 haplotypes containing the derived allele (where the sample derived allele frequency f=1%± 0.05%, see Appendix for details on how each simulation replicate is constructed). The recombination rates, both to the upstream and downstream of the loci, were assigned based on the average per base recombination rate in the 250-kb region surrounding each variant (see Supplementary Fig. 30). We calculated L moving upstream and downstream of the focal loci, obtaining approximately $\left(\begin{array}{c} 72\\ 2 \end{array}\right)\times \;2\times \;275\;L$ values for each simulation replicate. Using data simulated under 5 different selection coefficients, we found that we were able to obtain estimates of selection that appear unbiased (Supplementary Fig. 31). We obtained similar results when the simulated 275 loci shared the same recombination rate, although with a more notable slight bias for variants simulated with 4*Ns* = 0 and 4*Ns* = −25 (Supplementary Fig. 32). We compared these results with those obtained when the number of haplotypes containing the derived allele in the present is exactly equal to 72, since the likelihood function $L(4Ns,f,D|L\in w_{j})$ used for all the calculations across this section was built under the assumption that there are exactly 72 haplotypes with the derived allele in the present. We found few differences, in terms of the RMSE, on simulations performed with exactly 72 haplotypes with the derived allele in the present against simulations where the sample present-day derived allele frequency f takes values from f=1%± 0.05% using a likelihood function $L(4Ns,f,D|L\in w_{j})$ built under the assumption of having 72 haplotypes with the derived allele in the present (Supplementary Fig. 32). This analysis shows that our inference strategy does not lead to major biases due to small changes in the derived allele frequency, where the variants included in the analysis can take frequencies from 0.0095 to 0.0105, in this demographic scenario based on the simulation results under different recombination rates (Supplementary Fig. 32).

We must note that there are fewer differences in the distribution of $P(L\in w_{i}|f,D,4Ns)$ between variants under different strengths of selection in this demographic scenario compared with 2 other demographic scenarios we analyzed (Supplementary Fig. 33). This smaller amount of differences in $P(L\in w_{i}|f,D,4Ns)$ makes the inferences of selection much more challenging in this scenario. Additionally, the ESS are lower overall compared with other demographic scenarios, making the estimates of $L(4Ns,f,D|L\in w_{j})$ less accurate in this scenario compared with other scenarios (see Appendix for an explanation of the ESS; also see Supplementary Fig. 34). The ESS is an estimate of the sample size used in a Monte–Carlo evaluation of $L(4Ns,f,D|L\in w_{j})$ that is equivalent to the estimate we obtain using our importance sampling approach. Increasing the ESS is a topic that deserves further studies, since an improvement in our estimates of $L(4Ns,f,D|L\in w_{j})$ will increase the accuracy in our estimates of 4Ns (Supplementary Fig. 35).

We also calculated the probability of ancestral misspecification in the *UK10K* demographic scenario (Supplementary Table 6) and the results suggest that ancestry misspecification should not bias our estimates of selection (Supplementary Fig. 36). Phasing errors are not expected cause a bias in any direction in the estimates of 4Ns (Supplementary Fig. 37). Mutation rate and recombination rate misspecification can bias our estimates of selection, as seen in other demographic models (Supplementary Fig. 38). We performed simulations using the Boyko distribution of fitness effects under the scaled *UK10K* demographic model and the *UK10K* demographic model and found that we obtained estimates of $P_{\psi }\left(s_{j}\right)$ that appear unbiased on the $s_{j}$ intervals (Supplementary Figs. 39, 40 and Tables 7, 8).

We performed bootstrap replicates of the L values from the 275 1% frequency nonsynonymous variants of the *UK10K* dataset and the 142 1% frequency synonymous variants to evaluate the variation in our estimates of 4Ns. The variation around the estimates using bootstrap replicates is shown in Supplementary Fig. 41. The point estimates of 4Ns are equal to 3 for synonymous variants, and −50 for the nonsynonymous variants.

**Fig. 9.**
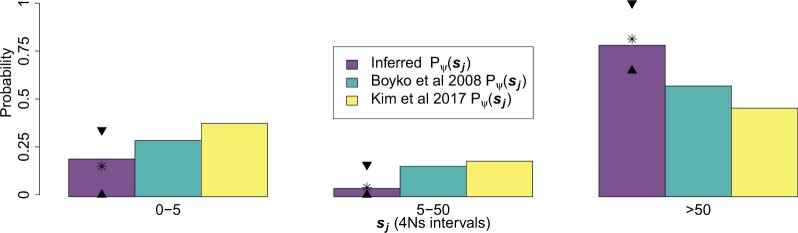
Inferred distribution of fitness effects of new mutations and 1% frequency deleterious variants in the *UK10K* dataset. “Inferred $P_{\psi }\left(s_{j}\right)$” refers to the probability of having a 4Ns value in a particular interval $s_{j}$ given the distribution of fitness effects of new mutations DFE. We estimated $P_{\psi }\left(s_{j}\right)$ for the $s_{j}$ interval = [5, 50) by summing up the $P_{\psi }\left(s_{j}\right)$ probabilities over the intervals [5, 10), [10, 15), [15, 20), [20, 25), [25, 30), [30, 35), [35, 40), [40, 45), and [45, 50). The selection coefficient *s* refers exclusively to the action of deleterious variants in this plot. We compared our inferences with those of Boyko *et al.* (2008) and Kim *et al.* (2017). The 2 triangles shown in each $s_{j}$ interval denote the upper and lower limit of the 90% bootstrap percentile interval across 100 bootstrap replicates. The asterisk signs are the mean values for the inferred probabilities $P_{\psi }\left(s_{j}\right)$ calculated from 100 bootstrap replicates.

We used the L values for the 275 nonsynonymous variants at a 1% frequency to infer the parameters of the distribution of fitness effects $DFE_{f}$. We assume that no derived variants we observe are under positive selection and that the $DFE_{f}$ follows a discretized gamma distribution, as explained in *A method for inference of the distribution of fitness effects for variants found at a particular frequency (“*${\mathrm{DFE}}_{f}$*”).* When we solved the integral from Equation (3), we used a value of τ=75. We only explored 4Ns values from 0 to 75 because we had high resolution for those 4Ns values (as indicated by ESS values bigger than 100), and values lower than −78 had low ESS values. We inferred a scale value of 0.06 and a shape value of 75,000. Based on a set of bootstrap replicates, we found that our estimates tend to cluster on the edges of the shape parameter values explored (Supplementary Fig. 42), indicating a high variance in our estimates of the $DFE_{f}$. This effect is specific to the inferred demographic scenario for the *UK10K* dataset, since we did not observe the same phenomenon in the simulations done under the constant population size and population expansion demographic scenarios we explored previously (Fig. 6). Based on our estimates of the $DFE_{f}$, we estimated $P_{\psi }\left(s_{j}\right)$ by employing Equation (7) and using our estimate of $P_{\psi }(f|D)$ [see Appendix for an explanation of our calculation of $P_{\psi }(f|D)$]. We compared those values with previously obtained estimates (Boyko *et al.* 2008; Kim *et al.* 2017). The point estimates of $P_{\psi }\left(s_{j}\right)$ along with the 90% bootstrap percentile intervals for other $s_{j}$ intervals are shown in Fig. 9 and Supplementary Fig. 43. We also show information for other bootstrap percentile intervals in Supplementary Table 9. We find that the upper limit of our 90% bootstrap percentile interval of $P_{\psi }\left(s_{j}\in [0,5)\right)$ and $P_{\psi }\left(s_{j}\in [5,50)\right)$ is smaller than the estimates computed by Kim *et al.* (2017) and bigger than the estimates computed by Boyko *et al.* (2008). On the other hand, the lower limit of our 90% bootstrap percentile interval of $P_{\psi }\left(s_{j}\in [50,\infty )\right)$ is bigger than the estimates of Boyko *et al.* (2008) and Kim *et al.* (2017). The probabilities of having a value of selection *s* over different orders of magnitude are shown in Supplementary Table 10 and are compared with the probabilities obtained by Boyko *et al.* (2008) and Kim *et al.* (2017). We also computed *P*-values under the null hypothesis that there is no difference between the estimated $P_{\psi }\left(s_{j}\right)$ values from the data and the $P_{\psi }\;\left(s_{j}\right)$ from the Boyko distribution of fitness effects (see Supplementary Fig. 44) and we cannot reject the hypothesis that the distribution of fitness effects inferred using the L values is different to the distribution of fitness effects estimated by Boyko *et al.* (2008) over the $s_{j}$ intervals [0,5), [5, 50) and [50, ∞) inspected.

## Discussion

We have developed a composite likelihood method to estimate the strength of natural selection acting on alleles at a certain frequency in the population. Our method builds upon previous work showing signatures of higher linkage disequilibrium for putatively deleterious alleles in comparison with neutral alleles (Kiezun *et al.* 2013). This result was shown to be in line with Takeo Maruyama’s work showing that deleterious alleles at a certain frequency tended to be younger than neutral alleles in constant population sizes (Maruyama 1974). Here, we introduce a method to estimate the strength of natural selection based on linkage disequilibrium using the pairwise identity by state lengths L.

In a constant population size scenario, we found that the distribution of L captures differences in the absolute strength of the selection coefficient 4Ns in a constant population size scenario. On the other hand, under some nonequilibrium demographic scenarios we found that the distribution of L is sufficient to differentiate between advantageous and deleterious alleles. This is encouraging, since most natural populations are very likely to have evolved under a nonequilibrium demographic scenario and precisely in some of those scenarios we will be capable to differentiate between deleterious and advantageous alleles.

Our simulations of allele frequency trajectories under several demographic scenarios are useful to understand past fluctuations in frequency and haplotypic patterns of selected alleles. The mean allele frequency trajectories of deleterious alleles segregating at a 1% frequency when the population is expanding are particularly noteworthy. These alleles tend to have increased in frequency when the population size is low. Then, they decrease in frequency when the population expands due to a higher efficacy of selection. This suggest that it is likely that, on average, deleterious alleles would tend to come from higher frequencies in the recent past in expanding populations. Recent work has analyzed how different summaries of genetic variation change over time in nonequilibrium scenarios (Peischl *et al.* 2013; Simons *et al.* 2014; Lohmueller 2014a; Balick *et al.* 2015; Do *et al.* 2015; Henn *et al.* 2015; Brandvain and Wright 2016; Marsden *et al.* 2016; Koch and Novembre 2017), and our work analyzing the behavior of frequency trajectories is helpful to understand those changes. Future work could also expand on the impact of selection in dominant and recessive alleles in nonequilibrium scenarios since the frequency trajectory of dominant or recessive alleles are different to what is observed in codominant alleles even on scenarios with constant population sizes (Mafessoni and Lachmann 2015).

In simulations, we find that our method can estimate parameters of the $DFE_{f}$ such that the mean of the $DFE_{f}$ is recovered in several scenarios. Under a constant population size, the scale estimates of the $DFE_{f}$ are inversely correlated with the shape parameters. Note that this curve decay causes the product of the scale and shape parameters to have relatively similar values. Under a population expansion model, the estimates of the shape and scale show a wider variation around the curve than the constant population size scenario (Fig. 6). Similarly, the pairwise coalescent time $T_{2}$ distribution between variants with different negative selection coefficients appear more similar to each other in a population expansion scenario when compared with a constant population size scenario (Figs. 4d and 2c). Due to the greater variation in the estimates of the parameters that define the $DFE_{f}$ of variants at a 1% frequency, we also see a larger variation in the mean 4Ns values estimated in a population expansion as compared with a constant population size demographic scenario based on the $DFE_{f}$ estimates (Fig. 6). Estimates of the mean 4Ns values are more precise under a constant population size compared with the population expansion scenario according to the estimates obtained from the $DFE_{f}$.

We developed an approach [via Equation (7)] to infer the DFE from the $DFE_{f}$ (assuming stationarity of the DFE). We tested our equation in simulations that include different demographic scenarios and DFE’s. We found that it provides accurate estimates of the DFE given the $DFE_{f}$ in a set $s_{j}$ of discrete bins of 4Ns (Fig. 7; Supplementary Figs. 6 and 7). The implication of this result is that an accurate estimate of the DFE can be obtained if we have an accurate estimate of the demographic scenario and the $DFE_{f}$. The $DFE_{f}$ is different from the DFE because the joint action of past demographic events and natural selection will not allow a frequency increase in deleterious variants where the effect of natural selection is stronger than that of genetic drift. Characterizing the $DFE_{f}$ of variants that have a particular functional category is of interest to understand how natural selection is acting to keep deleterious variants at low frequencies in the population. This information is of particular interest to debates on how natural selection and past demographic history influences changes in the genetic load between populations via the frequency decrease of deleterious alleles (Lohmueller 2014b; Brandvain and Wright 2016).

We tested different potential sources of errors in our estimates of selection. We found that ancestral state misspecification does not bias the point estimates of selection 4Ns for neutral and deleterious variants in 3 different demographic models (Supplementary Tables 5 and 6 and Figs. 12, 36). In the case of advantageous variants, we found that ancestral state misspecification does not bias the estimates of 4Ns under a constant population size demographic model but it causes an underestimation of the 4Ns estimate under a population expansion model (Supplementary Fig. 12). We caution that the fact that ancestral misidentification is not a problem for the demographic scenarios explored does not imply that it will never be problematic. A higher mutation rate will increase the amount of homoplasies and will increase the probability of ancestral state misidentification (Baudry and Depaulis 2003). If the ancestral state is defined using an outgroup species, a higher divergence from that species will also increase the probability of ancestral state misidentification (Hernandez *et al.* 2007). Some strategies have been proposed to estimate that particular probability (Baudry and Depaulis 2003; Ragsdale *et al.* 2016). An increase on the probability of ancestral state misidentification will create 2 different problems for the low frequency derived variants we use in our analysis: (1) low frequency ancestral variants miscalled as low frequency derived variants and (2) low frequency derived variants miscalled as low frequency ancestral variants. The first problem will bias the estimates of 4Ns because it will use L values from low frequency ancestral variants. The second problem will decrease the value of A and, in turn, reduce the number of L values to perform our inferences. This will decrease our precision of our 4Ns estimates, since we will have a smaller amount of data, L, to perform our inferences.

Biases in SNP and genotype calling can increase the apparent number of low-frequency variants appearing in genomic datasets. One solution to mitigate the effect of such errors is to remove low-frequency variants from our data before performing the inferences of selection. We tested that solution and found that we could obtain accurate estimates of selection (Supplementary Fig. 13). We also found that the statistical phasing of haplotypes does not bias our point estimates of selection of 4Ns, although it increases the variance on our 4Ns estimates (Supplementary Figs. 15 and 36). Our results also show that using an accurate mutation rate and recombination rate is critical to obtain unbiased estimates of selection (Supplementary Figs. 16 and 17). Finally, we perform forward-in-time simulations with SLiM to evaluate the impact of linked selection in our estimates of selection. We found that linked selection can bias the estimates of 4Ns for nonsynonymous variants and that the estimates possess a large variance, though the true values of 4Ns were contained inside the 25th and 75th percentile of the estimated 4Ns values although the intervals can be large (Supplementary Fig. 27). The impact of linked selection on estimates of 4Ns and the DFE is one topic that deserves further scrutiny for any proposed method to infer the impact of selection. Current community work in progress is standardizing forward-in-time simulations to simulate whole genomes under different demographic scenarios with the impact of selection incorporated on sites with a different functional category annotation (Adrion *et al.* 2020). We hope that all the developed methods to infer the impact of selection across the genome are tested under that standardized framework.

All of the analysis presented in this paper use 1% frequency variants. We decided to use variants at this particular frequency based on 2 observations: (1) the allele age of 1% frequency variants is approximately 8 times older for neutral variants compared with variants under a strength of natural selection equal to 4Ns=100 under a constant demographic scenario (Supplementary Table 1). The difference in the allele ages implies that there should be a significant difference in the values of $T_{2}$ between neutral and strongly selected alleles, as was observed in Fig. 2. This should also translate in significant differences in L values for 1% frequency variants, which is the statistic that we use for our inference. We showed that this was indeed the case for a constant population size scenario and all the other demographic scenarios we explored. (2) The number of nonsynonymous 1% frequency variants in the *UK10K* dataset was in the order of hundreds. We found that this number of variants was sufficient to provide reasonable estimates of $P_{\psi }\left(s_{j}\right)$ in the demographic scenarios we explored. There is a tradeoff with the analysis of variants at different frequencies. Variants at higher frequencies than 1% should display higher differences in L values when there are changes on the strength of natural selection acting on those variants. However, there are fewer variants at those higher frequencies. On the other hand, there are more variants at frequencies lower than 1% but the differences in L values on those variants should be lower. In the end, the best scenario would be to combine the information from variants at different frequencies. This is an avenue of research that should be fruitful to explore in the future.

Changes on the DFE over time could lead to differences in the inferred DFE from the SFS and the haplotypic data. DFE estimates from the SFS data use information from variants that have appeared across a broad range of time. On the other hand, the haplotype data we used comes from 1% frequency variants that have appeared recently.

The *UK10K* analysis we performed in this paper assume that the synonymous mutations are neutral. We used this assumption to be able to compare our DFE estimates for nonsynonymous mutations to previous results (Boyko *et al.* 2008; Kim *et al.* 2017), which also performed their analysis under the assumption that synonymous mutations are neutral. However, synonymous sites are under stronger natural selection than intergenic sites based on SFS data from 797 French Canadians showing a higher proportion of synonymous sites at lower frequencies compared with intergenic variants (Ragsdale *et al.* 2018). Related to this point, analysis based on the SFS have also shown that natural selection is acting on codon usage bias in the human genome giving further support to how natural selection acts on synonymous mutations (Dhindsa *et al.* 2020). The availability of large-scale whole genome sequencing data and ongoing efforts to perform whole-genome simulations including annotations for different functional genomic elements (Adrion *et al.* 2020) will allow us to have a better definition of which mutations from different functional elements will be a better proxy for neutral mutations depending on the functional category of mutations where we wish to infer the DFE. Apart from the strength of natural selection acting on the putatively neutral sites, it would be important to determine the appropriate genetic distance in bp or cM from the putatively neutral sites to the mutations where we wish to infer the strength of natural selection to avoid biases in the estimation of the DFE (Andolfatto 2008; Huang and Siepel 2019; Dhindsa *et al.* 2020).

Another biological phenomenon that could impact our DFE estimates is the incompleteness of the demographic model fitted to the data (Harris and Nielsen 2013; Garud *et al.* 2015; Beichman *et al.* 2017). We are fitting a demographic model with one deme to the *UK10K* dataset, and it is possible that fitting a model with population structure could give a better fit to the haplotypic data and to the SFS data (Harris and Nielsen 2013). We also are not modeling noncrossover gene conversion (Andolfatto and Nordborg 1998; Korunes and Noor 2017). Noncrossover gene conversion events involve haplotype tracts of approximately 100–1,000 bp and the probability that any site in the genome is involved in a noncrossover gene conversion event is $u=5.9\;\times \;10^{-6}$/bp/generation (Williams *et al.* 2015). Their impact is to break down linkage disequilibrium, which in our model, for a single variant would result in inferences that are biased towards neutrality; however, in aggregate if it impacts linkage disequilibrium around synonymous and nonsynonymous variants equally, the effect on inferences may be minor. Nonetheless, modeling noncrossover gene conversion could improve models of the haplotype signatures of selection.

One technical aspect from our methodology that could be subject to future improvement is to increase the ESS values. We only calculated the likelihood function of point estimates of 4Ns shown in Equation (2) in 4Ns values where we had high ESS, bigger than 100. In the case of the estimation of the $DFE_{f}$, the value of τ from equation was chosen to cover a set of values where we had ESS values bigger than 100. In the case of the *UK10K* dataset, the ESS values are smaller than 100 in 4Ns values smaller than −78 (Supplementary Fig. 34). To increase the values of the ESS, 1 possible improvement of our method is to make better proposals for the allele frequency trajectories going backwards in time. That is, to improve our choice of the importance sampling distribution. Future work will be devoted to make improvements in this issue, particularly in populations undergoing recent large expansions. One possibility is to expand the theory of Wright–Fisher bridges to select trajectories that end at a certain frequency f in the present under nonequilibrium scenarios (Schraiber *et al.* 2013). We did not find the same pattern of low ESS values in the other 2 demographic scenarios we analyzed (Fig. 2 and 4), where the population sizes did not experience changes in population size of the same magnitude as in the demographic model inferred in the *UK10K* data (Supplementary Fig. 34). Also, the values of L show fewer differences between different values of selection under the *UK10K* model compared with the 2 other demographic models analyzed (Supplementary Fig. 33) leading to estimates of selection with a larger variance under the *UK10K* model compared with the 2 other models (Figs. 3 and 5; Supplementary Figs. 31 and 32).

The accuracy of our inference method in dependent on the demographic model analyzed, along with the recombination rate and mutation rate on the regions surrounding the focal variants used in our analysis. We strongly recommend to perform simulations (as seen in the section *Forward-in-time simulations to assess the impact of selection on the allele frequency trajectories, allele ages, pairwise coalescent times*$T_{2}$, *and*L*values*) to explore if there is sufficient information to detect differences in L values given the particular demographic scenario, mutation rate and recombination rate used. Then, we recommend testing our inference method to see if the ESS are sufficiently high (at least bigger than 100) to perform inferences under that demographic model. The recombination rates have large variations across the genome (Kong *et al.* 2010) and this must be taken into account when performing inferences. We propose a strategy to take into account local variation in recombination rates in the *UK10K* dataset (Appendix) that gave DFE inferences that appear unbiased (Supplementary Figs. 39 and 40). We also suggest to test the $DFE_{f}$ and DFE inferences using this strategy via simulations under the particular inferred demography, mutation rates and recombination rates of the studied population.

Here we analyzed the distribution of fitness effects of nonsynonymous variants at a certain frequency. However, it is possible to determine the distribution of fitness effects of variants from other specific functional categories, such as variants that are predicted to be more deleterious based on the Fitcons (Gulko *et al.* 2015), SIFT (Sim *et al.* 2012), Polyphen (Adzhubei *et al.* 2010), or C-scores (Kircher *et al.* 2014; Racimo and Schraiber 2014). It is also be possible to estimate the strength of selection in a set of alleles that have a particular collection of genomic features (Huang and Siepel 2019). This can help us to obtain genome-wide estimates of the selection coefficient of variants based on their predicted functional category. This is of particular interest to genome-wide association studies, due to the interest in understanding the association between associated variants and their selection coefficients on different complex traits. Additionally, the use of the newly developed tree-sequence framework (Kelleher *et al.* 2018; Haller *et al.* 2019) for simulations should also help to speed up the calculation of the likelihood of different values of selection in the part of our method that depends on Monte–Carlo simulations. Another future avenue of research is to infer the distribution of selection coefficients of mutations acting at different frequencies in the population. Additionally, the differences in pairwise identity by state lengths between alleles under positive and negative selection under some demographic scenarios indicate that it could be possible to use the haplotypic information to infer the distribution of fitness effects including both advantageous and negative selection. The method presented here could be extended to infer the distribution of fitness effects including mutations under negative and positive selection that better explains the distribution of pairwise identity by state lengths. Broadly, we hope that the haplotype patterns are more exploited in future studies to infer the distribution of fitness effects of new mutations.

## Data availability

The programs and data to reproduce every figure of the paper can be found in https://github.com/dortegadelv/HaplotypeDFEStandingVariation. A pipeline with details on how to run the methods presented here is available here: https://github.com/dortegadelv/HaplotypeDFEStandingVariation/tree/master/Programs/ExamplePipeline. The version of the code used in this manuscript can be found in https://zenodo.org/record/5782755.

Supplemental material is available at *GENETICS* online.

## Supplementary Material

iyac002_Supplementary_Data

## Appendix

### Integration over the space of allele frequency trajectories using importance sampling

Performing an integration over the space of allele frequency trajectories $H_{k}$ where the allele has a frequency f in the present is challenging. One possible way to perform that integration step is to perform many simulations under the assumptions of the Poisson Random Field (PRF) framework (Sawyer and Hartl 1992; Hartl *et al.* 1994) and utilize rejection sampling to only keep those trajectories that end at a frequency f in the present. The number of mutations that enter the population each generation j have a Poisson distribution with mean $2N_{j}uK=\Theta /2$ under the *PRF* model. $N_{j}$ is the population size in generation j, u is the mutation rate per base and *K* is the number of sites being simulated. The sites are independent and the frequency of each mutation changes each generation following a Wright–Fisher model with selection. We could generate many allele frequency trajectories under this framework given a particular value of 4Ns and just keep those trajectories that end at a frequency f. However, this is inefficient and computationally demanding since the vast majority of allele frequency trajectories will not end at a frequency f in the present. And it is particularly more challenging if we wish to calculate L(4Ns,f,D|L) for a grid of 4Ns values.

Here, we used an importance sampling approach to integrate over the space of allele frequency trajectories and calculate the likelihood L(4Ns,f,D|L) over many different values of 4Ns. The efficient integration over the space of allele frequency trajectories is done using the importance sampling approach developed by Slatkin (2001) with a modification regarding the importance sampling distribution we use. Here the “target” distribution $f\left(x\right)=P(H_{k}|4Ns,f,D)$ are samples of allele frequency trajectories that end at a frequency f and have a selection coefficient 4Ns.

Following Slatkin (2001), we can define the trajectory $H_{k}$ of a derived allele *a* as the number of copies $i_{g}$ of the allele *a* each generation g since the allele appeared in the population. Therefore, $H_{k}=\{i_{T},i_{T-1},i_{T-2},\ldots ,i_{2},i_{1},i_{0}\}$, where $i_{T}=0$ and $i_{T-1}=1$. The effective population sizes at those times are $N=\{N_{T},N_{T-1},N_{T-2},\ldots ,N_{2},N_{1},N_{0}\}$. The allele appears in generation T-1, where it has 1 copy in the population.

We define the fitness of the genotypes *AA*, *Aa*, and *aa* as 1, 1+*s*, and 1 + 2s, respectively. Under a Wright–Fisher model with selection, the probability of moving from $i_{t}$ to $i_{t-1}$ copies of the allele going forward in time is equal to:

(A1) $$P\left(i_{t-1}|i_{t}\right)=p_{i_{t},i_{t-1}}=\left(\begin{array}{c} 2N_{t-1}\\ i_{t-1} \end{array}\right)x_{t}^{'i_{t-1}}{\left(1-x_{t}^{'}\right)}^{2N_{t-1}-i_{t-1}},$$

where

(A2) $$x_{t}^{\prime }=x_{t}\frac{1+2sx_{t}+s(1-x_{t})}{1+2sx_{t}^{2}+2sx_{t}(1-x_{t})}.$$

The frequency of the allele at generation t is $x_{t}=i_{t}/\;2N_{t}$.

As a “importance sampling” distribution $g\left(x\right)$, we use a very similar process to a Wright–Fisher neutral model. We start with the count *y* of the number of derived alleles *a* in the present based on a sample of n alleles. Estimating the frequency in generation 0 based on that sample of alleles is equal to the problem of estimating a probability based on binomial data. Therefore, we can follow Gelman *et al.* (2013) to state that the posterior density of the distribution of allele frequencies $\hat{f}$ in generation 0 is distributed as: $\hat{f}|(y,n)\;∼\mathrm{Beta}\left(y+1,n-y+1\right)$. Based on the distribution of $\hat{f},$ we can obtain the distribution of the number of alleles in generation 0, $i_{0}$, just by multiplying $i_{0}=\hat{f}n\;$and rounding $i_{0}$ to a discrete value. Then we can define the probability of having $i_{0}$ alleles in generation 0 given that we sampled y derived alleles in a sample of n alleles as

(A3) $$P\left(i_{0}|n,y\right)=P(X<\frac{i_{0}+0.5}{2N_{0}}|\mathrm{Beta}(y+1,n-y+1))-P(X<\frac{i_{0}-0.5}{2N_{0}}|\mathrm{Beta}(y+1,n-y+1)).$$

On the other hand, the probability that we obtain y derived alleles in a sample of n alleles given $i_{0}$ is

(A4) $$P\left(n,y|i_{0}\right)=\left(\begin{array}{c} n\\ y \end{array}\right){\left(\frac{i_{0}}{2N_{0}}\right)}^{y}{\left(1-\frac{i_{0}}{2N_{0}}\right)}^{n-y}.$$

To sample from $g\left(x\right)$, first we obtain a random value of $i_{0}$ using the probability distribution defined in Equation (A3). Then, we move backwards in time assuming that the allele is neutral. Under this proposal distribution, if $i_{t-1}=1$, then $i_{t}$ can take any value from 0 to $2N_{t}$. If $i_{t-1}=0\;$or $2N_{t}$ then we stop the allele frequency trajectory. If $i_{t-1}$ is bigger than 1 and smaller than $2N_{t}$, then $i_{t}$ can take any value from 1 to $2N_{t}$. These 3 rules are used together to make sure that each trajectory going forward in time always goes from 0 to 1 copy of the allele.

The transition probabilities under $g\left(x\right)$ of going from $i_{t-1}$ alleles in generation t-1 to $i_{t}$ alleles in generation $i_{t}$ are

(A5) $$P\left(i_{t}|i_{t-1}\right)=q_{i_{t-1}\;,i_{t}}=\left\{\begin{array}{cc} \frac{\left(\begin{array}{c} 2N_{t}\\ i_{t} \end{array}\right){\left(x_{t-1}\right)}^{i_{t}}{\left(1-x_{t-1}\right)}^{2N_{t}-i_{t}}}{1-\left(\begin{array}{c} 2N_{t}\\ i_{t} \end{array}\right){\left(x_{t-1}\right)}^{0}{\left(1-x_{t-1}\right)}^{2N_{t}}} & if\;i_{t-1}=\left(2,\;2N_{t}\right)\;\mathrm{and}\;i_{t}>0\\ \left(\begin{array}{c} 2N_{t}\\ i_{t} \end{array}\right){\left(x_{t-1}\right)}^{i_{t}}{\left(1-x_{t-1}\right)}^{2N_{t}-i_{t}} & if\;i_{t-1}=1\\ 0 & if\;1)\;i_{t-1}=0\;\mathrm{or}\;2N_{t}\;\mathrm{or}\;2)\;i_{t-1}=\left(2,\;2N_{t}\right)\;\mathrm{and}\;i_{t}=0\; \end{array}\right.$$

where $x_{t-1}=i_{t-1}/2N_{t-1}$. By generating an allele frequency trajectory with this importance sampling distribution, we can calculate the probability of any sample from this importance sampling distribution $g\left(x\right)$:

(A6) $$g\left(x\right)=P\left(i_{0}|n,y\right)\prod_{t=1}^{T}q_{i_{t-1},i_{t}}$$

Finally, the probability of the whole allele frequency trajectory $H_{k}$ going forward in time is then equal to

(A7) $$P\left(H_{k}|4Ns,f,D\right)=f\left(x\right)=P(n,y|i_{0})\prod_{t=T-1}^{1}p_{i_{t},i_{t-1}}$$

Now that we have defined how to sample allele frequency trajectories using our proposal distribution, we can compute the weight for every simulated allele frequency trajectory $H_{k}$ from $g\left(x\right)$ as $\omega_{k}=(f(x_{k})/g(x_{k}))$. For some of the proposed trajectories sampled under $g\left(x\right)$, the trajectory will end up at a frequency of 1 going backwards into the past, instead of 0. The value of $\omega_{k}$ for those trajectories is defined to be equal to 0.

The expected value that we wish to obtain with this problem is $L(4Ns,f,D|L\in w_{j})$. After generating R replicates using $g\left(x\right)$, we can compute that expected value under the importance sampling framework as

(A8) $$L(4Ns,f,D|L\in w_{j})=\frac{\sum_{k=1}^{R}\omega_{k}P\left(L\in w_{j}|H_{k}\right)}{\sum_{k=1}^{R}\omega_{k}}$$

Using this approach, we can estimate $L(4Ns,f,D|L\in w_{j})$ for different values of 4Ns using the same set of allele frequency trajectories generated from our importance sampling distribution. To do this, we reestimate $f(x_{k})$ using (A7) with a desired value of the selection coefficient *s* in (A2) using the set of generated allele frequency trajectories. Then, we calculate $\omega_{k}=(f(x_{k})/g(x_{k}))$, and we calculate $L(4Ns,f,D|L\in w_{j})$ with (A8). We can calculate the values of $L(4Ns,f,D|L\in w_{j})$ for other selection coefficients *s* by recalculating $f(x_{k})$, $\omega_{k}$, and then $L(4Ns,f,D|L\in w_{j})$ using the same set of generated allele frequency trajectories. This alleviates the need to simulate a different set of allele frequency trajectories for each value of the selection coefficient *s* that we want to evaluate and follows the idea of a driving value (Fearnhead and Donnelly 2001). The proposal distribution $g\left(x\right)$ is not necessarily optimal for every *s* value, but it is possible to verify if the distribution is reasonable based on the ESS values:

(A9) $$\mathrm{ESS}=\frac{1}{\sum_{i=1}^{R}\underline{{\omega_{i}}^{2}}}$$

where

(A10) $$\underline{\omega_{i}}=\frac{\omega_{i}}{\sum_{j=1}^{R}\omega_{j}}$$

The ESS indicates the sample size used in a Monte–Carlo evaluation of $f\left(x\right)$ that is equivalent to the importance sampling approach estimate. The ESS takes values between 1 and *R*, where a higher value of the ESS indicates that more samples from $g\left(x\right)$ are contributing to the estimate of the expected value of $L(4Ns,f,D|L\in w_{j})$. This is a necessary, but not sufficient, condition to obtain an accurate estimate of the expected value of $L(4Ns,f,D|L\in w_{j})$ when using an importance sampling approach. Values of ESS close to 1 indicate that few replicates of $g\left(x\right)$ are making a contribution of the expected value of $L(4Ns,f,D|L\in w_{j})$ and, therefore, the estimated expected value of $L(4Ns,f,D|L\in w_{j})$ is likely to not be accurate.

In every demographic scenario explored, we simulated *R* = 100,000 allele frequency trajectories to evaluate a set of discrete 4Ns values.

### Pairwise coalescent times $T_{2}$ given different values of selection

We investigated properties of the distribution of $T_{2}$ given different strengths of selection. To do this, we discretized and compressed each of the allele frequency trajectories we obtained using *PReFerSim* according to a set of allele frequency boundaries (0.0, 0.001, 0.002, 0.003, 0.004, 0.005, 0.006, 0.007, 0.008, 0.009, 0.01, 0.0125, 0.015, 0.02, 0.03, 0.04, 0.05, 0.06, 0.07, 0.08, 0.09, 0.1, 0.15, 0.2, 0.3, 0.4, 0.5, 0.6, 0.7, 0.8, 0.9, 0.95, 0.96, 0.97, 0.98, 0.99, 0.995, and 1.0) to reduce the computational time needed to simulate haplotypes under the structured coalescent model with *mssel*. When the allele frequency trajectory crosses a boundary, the allele frequency will be equal to the average of the new upper and lower boundaries when applying this compression. We then used each compressed allele frequency trajectory to estimate the distribution of pairwise coalescent times $T_{2}$ between a pair of haplotypes containing the allele changing in frequency. For each compressed allele frequency trajectory, we estimated the probability of coalescing at a time t as

(A11) $$P\left(T_{2}=t\right)=\left[\prod_{x=1}^{t-1}\left(1-\frac{1}{N_{a_{x}}}\right)\right]\frac{1}{N_{a_{t}}}$$

where $N_{a_{x}}$ denotes the number of chromosomes that have the derived allele at generation *x*. Additionally, due to the way we compressed the allele frequency trajectories, where the allele frequency at the time that the allele emerges is not equal to $1/N_{a_{x}}$, the probability of $T_{2}$ in the generation j where the allele appears is equal to $1-\sum_{t=1}^{j-1}P(T_{2}=t)$. We averaged the probabilities of $T_{2}$ over all the simulated allele frequency trajectories given a particular value of 4Ns to obtain the distribution of $T_{2}$ given a value of 4Ns.

### Connecting the distribution of fitness effects of variants at a particular frequency (*DFE_f_*) with the distribution of fitness effects of new mutations (*DFE*)

The distribution of fitness effects of variants at a particular frequency $DFE_{f}$ in the population is related to the distribution of fitness effects of new mutations DFE defined by a set of *κ* parameters $\psi =\{\psi_{1},\psi_{2},\psi_{3},\ldots ,\psi_{\kappa }\}$ using the following equation based on Bayes’ theorem:

(A12) $$P_{\psi }\left(f|s_{j},D\right)=\frac{P_{\psi }(s_{j}|f,D)\;P_{\psi }(f|D)}{P_{\psi }\left(s_{j}|D\right)}$$

where we can rearrange the above equation to obtain:

(A13) $$P_{\psi }\left(s_{j}|D\right)=P_{\psi }\left(s_{j}\right)=\frac{P_{\psi }(s_{j}|f,D)\;P_{\psi }(f|D)}{P_{\psi }(f|s_{j},D)}$$

If we look at the information of all nonoverlapping intervals **σ**=*{[*4Ns*_0_*, 4Ns*_1_), [*4Ns*_1_*, 4Ns*_2_), [*4Ns*_2_*, 4Ns*_3_)…, [*4Ns_*b*__−_*_1_*, 4Ns*_b_)} = {*$s_{1},s_{2},\;s_{3},\;\ldots ,s_{b}$*}* covering all 4Ns values from 0 to infinite, $P_{\psi }(s_{j}|f,D)$ defines the $DFE_{f}$ over a set of discrete bins. As seen in the section *A method for inference of the distribution of fitness effects for variants found at a particular frequency (“*${\mathrm{DFE}}_{f}$*”)*, we can use the L values to infer ${\mathrm{DFE}}_{f}(\alpha ,\beta )$. Then, we can estimate that $P_{\psi }\left(s_{j}|f,D\right)=P_{\alpha ,\beta }\left(s_{j}|f,D\right).$ In other words, $P_{\psi }\left(s_{j}|f,D\right)$ is equal to the probability of having a 4Ns value in the interval $s_{j}$ in variants at a frequency f given the discretized gamma distribution $DFE_{f}\left(\alpha ,\beta \right)$.

$P_{\psi }(f|D)$ can be computed by measuring the proportion of variants at a certain frequency f. That proportion must take into account all variants that have emerged during the demographic history D, including variants that have become fixed or have been lost. We can calculate $P_{\psi }(f|D)$ as:

(A14) $$P_{\psi }\left(f|D\right)=\frac{v_{f}}{\sum_{g=1}^{G}m_{g}}=\frac{v_{f}}{m}.$$

We define the number of variants at a frequency f in the present as $v_{f}$. The demographic history D encompasses G generations, where the number of mutations that appear every generation g is equal to $m_{g}$, Based on the Poisson Random Field, the number of mutations $m_{g}$ appearing every generation follows a Poisson distribution with mean $2N_{g}ul$, where $N_{g}$ is the number of individuals in generation g, u is the mutation rate per base and l is the number of bases where the mutation can take place. Therefore, we can estimate the expected value of m to use in the denominator in A14 as

(A15) $$E\left[m\right]=\sum_{g=1}^{G}E\left[m_{g}\right]=\sum_{g=1}^{G}2N_{g}ul$$

To calculate $P_{\psi }(f|s_{j},D)$, we can make the assumption that all the mutations in the interval $s_{j}$ have very similar selection coefficients, which is more likely to be true when the interval is not very big. This probability can be found via forward-in-time simulations using *PReFerSim*, where we simulate variants that have a selection coefficient contained in a certain interval $s_{j}$ in a particular demographic scenario D. That probability includes variants that have become fixed or have been lost during the demographic history D. We define the number of variants at a frequency f with a selection coefficient 4Ns contained in a certain interval $s_{j}$ in the present as $v_{f,s_{j}}$. The number of mutations that appear every generation g with a selection coefficient 4Ns contained in a certain interval $s_{j}$ is equal to $m_{g,s_{j}}$. In this case, the number of mutations $m_{g,s_{j}}$ appearing every generation follows a Poisson distribution with mean $2N_{g}ul_{s_{j}}$, where $N_{g}$ is the number of individuals in generation g, u is the mutation rate per base and $l_{s_{j}}$ is the number of bases where a mutation leading to a derived allele with a selection coefficient 4Ns contained in a certain interval $s_{j}$ can take place. Therefore, we can estimate the expected value of $m_{s_{j}}$ as:

(A16) $$E\left[m_{s_{j}}\right]=\sum_{g=1}^{G}E\left[m_{g,s_{j}}\right]=\sum_{g=1}^{G}2N_{g}ul_{s_{j}}$$

And we use the expected value of $m_{s_{j}}$ in the denominator of the following equation:

(A17) $$P_{\psi }\left(f|s_{j},D\right)=\frac{v_{f,s_{j}}}{\sum_{g=1}^{G}m_{{g,s}_{j}}}=\frac{v_{f,s_{j}}}{m_{s_{j}}}.$$

To accelerate the calculation of $P_{\psi }(f|s_{j},D)$ we can perform the strategy of doing forward-in-time simulations under an arbitrary DFE that encompasses all the intervals **σ**$=\{s_{1},s_{2},\;s_{3},\;\ldots ,s_{b}\}$ analyzed where we know the proportion of new mutations $p_{s_{j}}$ that will be contained inside an interval $s_{j}$ when the number of bases where the mutation can take place is equal to l. In this case, we can estimate the expected value of $m_{s_{j}}$ to use as the denominator in Equation (A17) as:

(A18) $$E\left[m_{s_{j}}\right]=\sum_{g=1}^{G}E\left[m_{g,s_{j}}\right]=\sum_{g=1}^{G}2N_{g}ulp_{s_{j}}$$

We used that strategy in Supplementary Figs. 8, 23, 26, 39, 40, 43, and Fig. 9.

We calculate $P_{\psi }\left(s_{j}\right)$ for the first b-1 intervals using Equation (7). Then, for the last interval ***s_b_*** we use $P_{\psi }\left(s_{b}\right)=1-\sum_{i}^{b-1}P_{\psi }(s_{i})$. If $\sum_{i}^{b-1}P_{\psi }(s_{i})>1.0$, we set the probabilities $P_{\psi }(s_{j})=P_{\psi }(s_{j})/\sum_{i}^{b-1}P_{\psi }(s_{i})$ for the first b-1 intervals and $P_{\psi }(s_{j})=0$ for the last interval b.

We tested if Equation (7) could provide accurate estimates of $P_{\psi }\left(s_{j}\right)$ using forward-in-time simulations with *PReFerSim*. We performed simulations under the human DFE (shape = 0.184; scale = 319.8626; *N* = 1,000) (Boyko *et al.* 2008), the mice DFE (shape = 0.11; scale = 8,636,364; *N* = 1,000,000) (Halligan *et al.* 2013), and a version of the human DFE with a scale value that is 20 times smaller. These simulations were performed under the constant population size demographic model and the population expansion model. We calculated $P_{\psi }(s_{j}|f,D)$ and $P_{\psi }(f|D)$ from a set of 1% frequency variants obtained in simulations under a particular demographic model and DFE. We calculated $P_{\psi }(f|D)$ from Equation (A14) employing Equation (A15) to estimate $E\left[m\right]$. $P_{\psi }\left(f|s_{j},\;D\right)$ was estimated in all cases by performing simulations under an arbitrary DFE that is different from the DFE under study. The results from these analyses are shown in Fig. 7 and Supplementary Figs. 5–7, where we used the mice DFE to estimate $P_{\psi }\left(f|s_{j},\;D\right)$. The number of simulations and the Poisson mean of the number of mutations per epoch performed to calculate $P_{\psi }(f|s_{j},D)$ and $P_{\psi }\left(f|D\right)$ is equivalent to having a ul value equal to 250 in the results of Fig. 7 and Supplementary Fig. 6 when using Equations (A14), (A15), (A17), and (A18). On the case of Supplementary Fig. 7 the ul value was equal to 125. ul is equal to 100 to calculate $P_{\psi }\left(f|D\right)$ while ul is equal to 500 to calculate $P_{\psi }(f|s_{j},D)$ in Supplementary Fig. 5.

### ABC-based inference of the demographic scenario

We inferred demographic models using variants at a f = 1% frequency in the population. To do this, we first stablish a demographic model we will investigate with a set of parameters we wish to infer. We define a set of prior distributions for each parameter and use an *ABC* approach that follows this procedure:

1. Take a dataset of A loci with a derived allele at a frequency  f = 1%, where the number of derived alleles in each of the A loci is equal to n. Obtain a distribution $\ell =2\times A\times \left(\begin{array}{c} n\\ 2 \end{array}\right)$ of L values. That distribution is calculated by taking all possible pairs of haplotypes with the derived allele, going upstream and downstream in a region of l kb with the focal loci in the middle of the region for the A loci. Then, use that distribution of L values to calculate $D_{w_{i}}=P(L\in w_{i})$ for each of the 6 windows $w_{i}$.
2. Draw a random value for each parameter from each prior distribution of demographic parameters.
3. Simulate A random allele frequency trajectories $H_{k}$ where the allele ends at a frequency f = 1*%* in the present using the demography based on the parameter values sampled from the prior distribution. We do this step using *PReFerSim.*
4. Use *mssel* to simulate n haplotypes containing the derived allele in the middle of the simulated region for each simulated allele frequency trajectory $H_{k}$ obtained in the past step. After this step, A datasets with n derived haplotypes were simulated. The average per base mutation rate u and the average per base recombination rate r in these simulations of haplotypes should be defined by the user.
5. Use the A datasets to calculate $\ell =2\times A\times \left(\begin{array}{c} n\\ 2 \end{array}\right)$ values of L by taking all possible pairs of haplotypes with the derived allele, going upstream and downstream in the A alleles. Use that distribution of L values to calculate ${D^{\prime }}_{w_{i}}=P(L\in w_{i})$ for each of the 6 windows $w_{i}$.
6. Calculate $\alpha =\sum_{i=1}^{6}\left|{D^{\prime }}_{w_{i}}-D_{w_{i}}\right|$
7. Go back to 2) until 10,000 values have been sampled for each demographic parameter from the prior distributions.
8. Retain the 100 simulations where the value of α is smaller. The values obtained for the parameters in those 100 simulations define the posterior distributions of those parameters. The point estimates of each parameter were defined as the median of the posterior distribution.

The values of A, n, and l were set to 150, 40 and 500,000 in these tests, respectively. The 6 windows $\left\{w_{1},\;w_{2},\;w_{3},\;w_{4},\;w_{5},\;w_{6}\right\}$ were set equal to *{(0, 50*,*000], (50*,*000, 100*,*000], (100*,*000, 150*,*000], (150*,*000, 200*,*000], (200*,*000, 250*,*000], (250*,*000*, ∞*)}.* We tested our *ABC* approach in the constant population size scenario and the population expansion scenario in 100 simulated datasets for each demographic scenario using values of $u=1.2\;\times \;10^{-8}$ and $r=1\;\times \;10^{-8}$. The results from these tests can be seen in Supplementary Figs. 9 and 10. We did an additional analysis under the population expansion scenario where each of the A=150 variants had a different recombination rate sampled with replacement from the distribution of the 142 average recombination rates per base using the 250 kb upstream and downstream region surrounding each of the 142 *1%* synonymous variants of the *UK10K* dataset. The results from this analysis are shown in Supplementary Fig. 11. We tested our *ABC* approach in the case of the constant population size scenario setting the effective population size *N* a single parameter that we wish to infer. The uniform prior distribution for this parameter is a N ∼ uniform(1,000, 20,000). On the other hand, 3 parameters to infer in the population expansion scenario are the effective population size in the ancient epoch, the effective population size in the present epoch and the time when the population size changes. The uniform prior distribution for those parameters are uniform(1,000, 10,000), uniform(1,000, 100,000), and uniform(0, 500), respectively.

### Assessing the impact of ancestral state misidentification on our estimates of selection

We assessed the impact of ancestral state misidentification on our method. We use the information from L in a set of haplotypes carrying a derived allele at a frequency f. If the allele at a frequency f is actually an ancestral allele, this could lead to potential biases in our inferences of selection. The probability of miscalling an ancestral allele at a frequency f as a derived allele is dependent on the number of ancestral alleles and derived alleles at a frequency f, and the probability of ancestral state misidentification $p_{\mathrm{misid}}$. Following (Ragsdale *et al.* 2016), we define that the observed number of sites containing a set of alleles called to be derived, either correctly or incorrectly, at a frequency fF(f) will be equal to $F(f)=F_{\mathrm{True}}(f)\;(1-p_{\mathrm{misid}})+F_{\mathrm{True}}\left(\;1\;–\;f\;\right)p_{\mathrm{misid}}$. Here, $F_{\mathrm{True}}(f)$ and $F_{\mathrm{True}}(1-f)$ are the number of sites containing a derived allele and an ancestral allele at a frequency f, respectively. The count of those number of sites will be dependent on the action of natural selection and the demographic scenario under study (Supplementary Table 5). The proportion of ancestral alleles at a frequency f incorrectly called as derived alleles correctly will be equal to:

(A19) $$P_{\mathrm{incorrect}}(f)=(F_{\mathrm{True}}\left(\;1\;–\;f\;\right)p_{\mathrm{misid}})/(F_{\mathrm{True}}(f)\;(1-p_{\mathrm{misid}})+F_{\mathrm{True}}\left(\;1\;–\;f\;\right)p_{\mathrm{misid}}).$$

A recent study reported values of $p_{\mathrm{misid}}$ between 0.001 and 0.015 for different categories of sites in humans (Fortier *et al.* 2019). In our analysis, we use a value of $p_{\mathrm{misid}}$ equal to 0.015. Based on that value of $p_{\mathrm{misid}}$, we found that $P_{\mathrm{incorrect}}(f)\;$was equal to 0 for deleterious alleles, <0.00016 for neutral alleles and <0.012 for advantageous alleles in the demographic scenarios analyzed (Supplementary Table 5). We performed simulations where an ancestral allele could be incorrectly called as a derived allele at a frequency f based on $P_{\mathrm{incorrect}}(f)\;$and found that the ancestral state misspecification did not cause biases on the estimation of 4Ns for neutral and deleterious alleles. We also found that the strength of selection for advantageous alleles was slightly underestimated (Supplementary Fig. 12).

### Assessing the robustness of our method to biases in SNP and genotype calling in low frequency variants, statistical haplotype phasing errors, recombination rate misspecification, mutation rate misspecification, and Monte–Carlo strategy used to estimate the pairwise identity by state lengths

Biases in SNP and genotype calling are another source of concern for the application of our method. Those biases cause a decrease or an increase on the number of called rare variants depending on the pipeline used to do the SNP and genotype calling. The impact of these biases is more dramatic for singleton variants compared with other higher frequency variants (Han *et al.* 2014). One way to mitigate these effects if to mask very low frequency variants that are prone to sequencing errors. We tested this idea in a set of 4Ns values in each inspected demographic scenario by examining 100 simulation replicates (see *Forward-in-time simulations to assess the impact of selection on the allele frequency trajectories, allele ages, pairwise coalescent times*$T_{2}$, *and*L*values*) where we mask variants that appear only once in each set of haplotypes with the derived variant before estimating L. Those variants were also masked when we performed the Monte–Carlo estimations of $P\left(L\in w_{j}|H_{k}\right)$ before we estimated $L(4Ns,f,D|L\in w_{j})$ in Equation (1). The results of these analysis are shown in Supplementary Fig. 13.

The haplotype phase of the majority of the large-scale whole-genome datasets available is resolved utilizing statistical phasing software (Francioli *et al.* 2014; Walter *et al.* 2015; Wall *et al.* 2019), such as *ShapeIT* (Delaneau *et al.* 2019) and *Eagle* (Loh *et al.* 2016). We analyzed the impact of haplotype phasing errors due to the use of statistical phasing software in estimates of selection using our method. We analyzed our inferences of 4Ns in simulations performed with 5 different values of 4Ns in the constant population size model and the population expansion model. We used our simulation pipeline to obtain 100 simulations replicates with 1 loci A=1 simulated in each simulation replicate. We obtained n=40 haplotypes with the derived allele and 3,960 haplotypes with the ancestral allele for each simulation replicate. Those haplotypes were randomly sampled without replacement to obtain 2,000 individuals where the phase of the haplotypes was unknown. We statistically phased the haplotypes using *ShapeIt2*, which was also used to phase the *UK10K* dataset, using the following command:
shapeit.v2.904.3.10.0-693.11.6.el7.x86_64/bin/shapeit --input-vcf <PhasedDataPrefix> -M <GeneticMap> -O <PhasedDataOutput> --output-log <LogOutput> where PhasedDataPrefix is a vcf file with the unphased data of 2,000 individuals; GeneticMap contains the recombination rate of the region; and PhasedDataOutput is the output statistically phased vcf file of the region. We estimated the L values in the statistically phased and compared them with the L values obtained if the haplotype phase was perfectly known (Supplementary Fig. 14). We used the L values of the statistically phased haplotypes to estimate 4Ns. We compared those estimates of selection with the estimates we obtain when we use the L values in the same set of haplotypes if we knew the haplotype phase perfectly (Supplementary Fig. 15).

We also explored the impact of recombination rate misspecification and mutation rate misspecification in our estimates of selection. To do this, we performed simulations where the values of ρ or θ were higher or smaller than the values used to calculate $L(4Ns,f,D|L\in w_{m_{j}})$ and then perform the inferences using Equation (2) (Supplementary Figs. 16 and 17).

The accuracy of the estimates of $P\left(L\in w_{j}|\;D,H_{k}\right)$ based on the Monte–Carlo strategy will impact the likelihood estimates of 4Ns based on Equation (2). We compared our estimates based on the Monte–Carlo strategy previously described where we simulate 100 sets of n haplotypes to compute $\left(\begin{array}{c} n\\ 2 \end{array}\right)$ values of L for each set to obtain $100\times \left(\begin{array}{c} n\\ 2 \end{array}\right)$ values of L for each $H_{k}$. We compared this strategy with 3 alternative methodologies to compute $P\left(L\in w_{j}|\;D,H_{k}\right)$: (1) simulate twice the number of sets of n haplotypes, 200, for each $H_{k}$ to obtain $200\times \left(\begin{array}{c} n\\ 2 \end{array}\right)$ values of L for each $H_{k}$; (2) simulate sets of n haplotypes with the derived allele loci located at the center end of a simulated 500 kb region and then calculate $\ell =2\times \left(\begin{array}{c} n\\ 2 \end{array}\right)$ values of L for each set of n simulated haplotypes by estimating L in all the possible comparisons of haplotype pairs containing the derived allele at both sides of the focal site to obtain $2\times 100\times \left(\begin{array}{c} n\\ 2 \end{array}\right)$ values of L for each $H_{k}$; and (3) simulate sets of n haplotypes with the derived allele loci located at the center end of the simulated 500 kb region and take the information of the upstream and downstream distances to construct a single statistic L′ that measures pairwise identity by state lengths to obtain $\ell =100\times \left(\begin{array}{c} n\\ 2 \end{array}\right)$ values of L′ for each $H_{k}$ (Supplementary Fig. 20).

We also explored the impact of recombination rate variation in our estimates of selection. First we generated 100 simulation replicates under a certain 4Ns value that contained $A_{1}=150$ variants with a recombination rate r=0 and $A_{2}=150$ variants with a recombination rate $r=1\times 10^{-8}.\;$The L values obtained with a recombination rate r=0 are written as ${L^{A_{1}}}_{j}$, while the L values obtained with a recombination rate $r=1\times 10^{-8}$ are written as ${L^{A_{2}}}_{j}$. Then, following Equation (1), we estimated the likelihood of different 4Ns values using

$$L\left(4Ns,f,D|L\right)=\prod_{j=1}^{\ell_{A1}}L(4Ns,f,D,\rho =0|{L^{A_{1}}}_{j}\in w_{m_{j}})\prod_{j=1}^{\ell_{A2}}L(4Ns,f,D,\rho =1,000|{L^{A_{2}}}_{j}\in w_{m_{j}}),$$

where $\rho =4N\hat{r}l$ are the average recombination rates. N is the population size in the present, $\hat{r}$ is the average per base recombination rate in a region of length l=500 kb with the focal allele in the center of the region. The estimate of selection is found by maximizing this composite likelihood function by using a grid search over a range of candidate 4Ns values going from −200 to 200. $L(4Ns,f,D,\rho =0|{L^{A_{1}}}_{j}\in w_{m_{j}})$ and $L(4Ns,f,D,\rho =1,000|{L^{A_{2}}}_{j}\in w_{m_{j}})$ are estimated using Equation (1) with a recombination rate equal to r=0 and $r=1\times 10^{-8}$, respectively.

The calculation of $L(4Ns,f,D,\rho |{L^{A_{1}}}_{j}\in w_{m_{j}})$ for a particular population-scaled recombination rate value ρ requires approximately 100 h of computation time. Therefore, if we have 300 variants with a different recombination rate, then the computation time scales to 30,000 h. Since this computation time is too large, we developed a strategy to reduce the computation time. Our strategy follows these steps:

1. We took the 21 different percentile values (0^th^, 5^th^, …, 95^th^, 100^th^) from the distribution of 300 average recombination rates $\rho =4N\hat{r}l$, where N is the population size in the present, $\hat{r}$ is the average per base recombination rate in a region of length l taking the upstream and downstream 250 kb regions next to the 300 1% frequency variants.
2. We generated 21 likelihood functions $L(4Ns,f,D,\rho_{j}|L\in w_{i})$ for each selection value explored, each with a different recombination rate $\rho_{j}$ from the 21 different percentile values (0^th^, 5^th^, …, 95^th^, 100^th^) of 300 average recombination rates $\rho_{i}$.
3. We estimated the likelihood function $L(4Ns,f,D,\rho_{k}|L\in w_{i})$ across the 300 regions. To do this we take each of the 6 windows $w_{i}=\{w_{1},w_{2},w_{3},w_{4},w_{5},w_{6}\}$ and:
4. We take the 21 values of $L(4Ns,f,D,\rho_{j}|L\in w_{i})$ from the 21 different percentile values (0^th^, 5^th^, …, 95^th^, 100^th^) for each value of $w_{i}$.
5. We fit a polynomial regression model with 4 regression coefficients, with the independent variable being equal to $\rho_{j}$ and the dependent variable being equal to $L\left(4Ns,f,D,\rho_{j}^{}|L\in w_{i}\right)$.
6. We predicted the value of $L\left(4Ns,f,D,\rho_{k}^{}|L\in w_{i}\right)$ for every one of the 300 average population-scaled recombination rates $\rho_{k}^{}$ in the 300 regions based on the values of the polynomial regression model.

We chose to use 4 regression coefficients based on analysis done with a different number of regression coefficients, and we chose to use the smallest amount of regression coefficients where there was a significant improvement compared with using 1 less regression coefficient based on an error metric ε (Supplementary Table 11). As an example of the good fit of the polynomial regression model across the 6 windows $w_{i}$, we plotted the predicted values of $L\left(4Ns,f,D,\rho_{k}^{}|L\in w_{i}\right)$ based on the polynomial regression model with 4 regression coefficients when 4Ns=0 (Supplementary Fig. 45). We estimated the values of 4Ns in 100 simulation replicates with 300 variants with variable recombination rates. We sampled 300 recombination rates r with replacement from the distribution of 275 average recombination rates per base in the 250-kb upstream and downstream region of a focal loci in the *UK10K* dataset. Those sampled r values were fixed in the 100 simulation replicates. Then, we estimated the likelihood equation using the collection of lengths $L_{k_{j}}=\{L_{k_{j_{1}}},L_{k_{j_{2}}},L_{k_{j_{3}}},\ldots ,L_{k_{j_{2\times (\begin{array}{c} 40\\ 2 \end{array})}}}\}$ of the $2\times \left(\begin{array}{c} 40\\ 2 \end{array}\right)$ values of *L* in each of the 300 regions *k*:

$$L\left(4Ns,D,f|L\right)=\prod_{k=1}^{300}\prod_{i=1}^{\ell_{j}=2\times \left(\begin{array}{c} 40\\ 2 \end{array}\right)}L(4Ns,f,D,\rho_{k}^{}|L_{{k_{j_{i}}}_{}}\in w_{m_{k_{j_{i}}}})$$

where $w_{m_{k_{j_{i}}}}$is an integer between 1 and M=6 indicating the window in which the length $L_{{k_{j_{i}}}_{}}$falls. And used that likelihood equation to obtain an estimate of 4Ns using a grid approach as in the previous analysis. The results are shown in Supplementary Fig. 22.

We used the likelihood functions $L\left(4Ns,f,D,\rho_{k}^{}|L\in w_{i}\right)$ in the 300 regions and Equation (4) to estimate $L\left(\alpha ,\beta ,D,f|L\right)$ using the collection of lengths $L_{k_{j}}=\{L_{k_{j_{1}}},L_{k_{j_{2}}},L_{k_{j_{3}}},\ldots ,L_{k_{j_{2\times (\begin{array}{c} 40\\ 2 \end{array})}}}\}$ of the 300 regions *k* to calculate:

$L\left(\alpha ,\beta ,D,f|L\right)=\prod_{k=1}^{300}\prod_{i=1}^{\ell_{j}=2\times \left(\begin{array}{c} 40\\ 2 \end{array}\right)}L(\alpha ,\beta ,f,D,\rho_{k}^{}|L_{{k_{j_{i}}}_{}}\in w_{m_{k_{j_{i}}}})$

We used this likelihood function to obtain an estimate of the parameters that define the $DFE_{f}$ of the 1% frequency variants in the population expansion scenario using a grid approach. The results from this analysis are shown in Supplementary Fig. 23.

### Assessing the impact of linked selection on our estimates of selection via forward-in-time simulations

We evaluated the impact of linked selection in our estimates of selection. To do this we performed forward-in-time simulations using SLiM (Haller and Messer 2019). These simulations mimic the arrangement of functional elements and recombination rates across the human genome. These simulations cover the human genome in 101 nonoverlapping regions of 20 Mb. We used a scaled population expansion demographic model in this set of analysis based on the population expansion model (Fig. 4). The recombination rate in the simulated regions follows an inferred recombination map in humans (Kong *et al.* 2010). The arrangement of exonic elements is taken from GENCODE v14 (Harrow *et al.* 2012) and the position of the conserved noncoding elements is defined as in (Huber *et al.* 2017). We defined that the DFE of variants in conserved noncoding elements follows a gamma distribution (shape = 0.0415; scale = 640; *N* = 25,636) (Torgerson *et al.* 2009). We assumed that the proportion of synonymous mutations in exonic regions is equal to 1/3.31 (Huber *et al.* 2017), and that synonymous mutations are neutral as assumed in recent papers that infer the DFE. We evaluated the inference of selection when the nonsynonymous mutations had a point population-scaled selection coefficient equal to 0, −50 or −100, and when the DFE of the nonsynonymous mutations followed a gamma distribution of fitness effects inferred in humans (shape = 0.184; scale = 319.8626; *N* = 1,000) (Boyko *et al.* 2008). We scaled the population expansion demographic model (Fig. 4) to avoid a very slow runtime of the SLiM simulations. To do this, we reduced the population size and population expansion time by a factor of 5, and we increased the mutation rate (to get a $u=1.2\;X\;10^{-8}*5$), recombination rate and selection coefficients by the same factor. The number of chromosomes simulated was equal to 4,000.

We used the following procedure to test our inferences of selection for each of the 3 4Ns values evaluated and the Boyko DFE:
0) We performed many simulations of 20 Mb regions across all the human genome in 101 nonoverlapping regions until we obtained at least 600 and 300 regions upstream or downstream of a nonsynomyous or synonymous site, respectively, of a derived allele at a f = 1% (n = 40) frequency where the average per base recombination rate in the region was smaller than $1.2\;X\;10^{-10}*5$. Therefore, the recombination rate in the region was more than 100 times smaller than the mutation rate in the region. Since the recombination rate was more than 2 orders of magnitude smaller than the mutation rate, we assumed a recombination rate r=0 for simplicity in all the analysis from step 1) to 4). 1) We randomly sampled 300 regions with a synonymous site at a frequency f = 1%, and we used those regions to estimate the demographic scenario *D* using our ABC algorithm. We used the collection of $\;L=300\times \left(\begin{array}{c} 40\\ 2 \end{array}\right)$ of values of ***L*** to estimate $D_{w_{i}}=P(L\in w_{i})$ and then we used our *ABC* algorithm starting from step 2. When we ran the *ABC* algorithm, we only calculated ***L*** going downstream to obtain the same number of $L=300\times \left(\begin{array}{c} 40\\ 2 \end{array}\right)$***L*** values as in the data for each draw of parameters from the prior distribution. We used 50,000 draws of parameters before calculating the point estimate for all the parameters as defined in the section *ABC-based inference of the demographic scenario*. Then, we estimated $L\left(4Ns,f,D|L\in w_{m_{j}}\right)$ for inferences of population-scaled selection coefficients and $L(\alpha ,\beta ,D,f|L\in w_{m_{j}})$ for inferences of the DFE_*f*_ based on the inferred demographic scenario D. We used a per base mutation rate of $u=1.2\;X\;10^{-8}*5\;$ and a per base recombination rate equal to 0 to estimate $L\left(4Ns,f,D|L\in w_{m_{j}}\right)\;$ and $L\left(\alpha ,\beta ,D,f|L\in w_{m_{j}}\right).$ 2) We created 100 sets of simulation replicates. We randomly sampled 600 nonsynonymous sites with replacement to create each simulation set. 3) We estimated the value of selection in each simulation replicate using equation (2) in the case of the simulations performed using a point 4Ns value and equation (4) in the case of the simulations where we wanted to estimate the parameters of a DFE. 4) We go back to 1) until we have estimated the value of selection in 10 different inferred demographic scenarios. The results for each of the 10 demographic scenarios are shown as simulation replicates in Supplementary Figs. 24–26.

### *ABC* algorithm used to infer the demographic scenario consistent with haplotypic patterns at 1% frequency synonymous variants in the *UK10K* dataset

Our *ABC* algorithm has the following steps:

Data preparation:

1. We will infer a demographic history using synonymous variants at a frequency f=1%± 0.05%. To do this, we will infer the demographic history that better explains the distribution L of variants at a frequency f=1%± 0.05%.
2. Out of the 3,781 individuals present in the phased *UK10K* haplotype reference panel, we selected the 3,621 individuals that had European ancestry, along with a set of individuals that were not related to other individuals in the panel, as previously defined in the original *UK10K* study (Walter *et al.* 2015).
3. We estimated the frequency of every variant present in the phased haplotype panel.
4. To identify the synonymous variants, we used the functional annotations for each variant from Ensembl 75 Variant Effect Predictor, as reported in the vcf file with allele frequencies available from the *UK10K* website (https://www.uk10k.org/data.html). Since some variants annotated as a synonymous mutation can possess more than one functional annotation, we only considered a variant to be synonymous if it did not possess another annotation that had a higher impact (HIGH or MODERATE) as defined in https://www.ensembl.org/info/genome/variation/prediction/predicted_data.html.. Variants with a high or moderate impact are those annotated to be transcription ablation, splice acceptor variants, splice donor variants, stop gained, frameshift, stop lost, start lost, transcript amplification, inframe insertion, inframe deletion, missense, or protein altering variant.
5. We used the annotations from the 1000 Genomes Project phase 3 (Auton *et al.* 2015) to define the ancestral allele of each variant. This also allows us to define the derived allele of every site.
6. Depending on the frequency f of the variants being investigated, retain all the Y synonymous variants $V=\{V_{1},\;{\;V}_{2},\;\ldots ,V_{Y}\}$ that: 1) Have a derived allele frequency in the interval 1*%*± 0.05% and 2) are more than 5 Mb away from the centromeres or telomeres. In the end we retained 142 synonymous variants.
7. For every variant $V_{i}$, take the number $n_{V_{i}}$ of haplotypes $h_{V_{i}}$ containing the derived allele. Then remove the sites where there is a singleton variant in the sample $h_{V_{i}}$. After removing those sites, calculate the haplotypic length L for every possible pair of haplotypes in $h_{V_{i}}$ going upstream and downstream. The *UK10K* haplotype panel does not contain singleton variants present in the sample of 7,242 chromosomes. Therefore, the calculation of L in this step is done ignoring singleton sites, either because they were not present in the original *UK10K* panel or because we remove them from the sample $h_{V_{i}}$. After performing the L calculations, we will end up with $\ell =\sum_{i=1}^{Y}2\times \left(\begin{array}{c} n_{V_{i}}\\ 2 \end{array}\right)$ values of L.
8. We define 6 nonoverlapping windows $W=\{w_{1},\;w_{2},\;w_{3},\;w_{4},\;w_{5},\;w_{6}\}$*= { (0, 50000], (50000, 100000], (100000, 150000], (150000, 200000], (200000, 250000], (250000*, ∞*)}.* Then, we calculate $P(L\in w_{i})$ using the distribution of L values obtained in the previous step. The distribution $D_{w_{i}}=P(L\in w_{i})$ across the 6 *i* windows represents the summary statistic we will use to infer demography in our *ABC* algorithm.

*ABC* algorithm:

We constructed a demographic model for the synonymous variants at a 1% ± 0.05% frequency in the population. A graphical representation of the model is presented in Supplementary Fig. 28, along with the prior distributions for the demographic parameters of the model. The parameters to infer are the population size in the present $N_{1}$, the population size in the preceding epoch $N_{2}$ and the time $T_{1}$ when the population size changes from $N_{2}$ to $N_{1}$. The uniform prior distributions used for those parameters are $T_{1}\;∼\;\mathrm{uniform}(0,\;\;1,449)$, $N_{1}\;∼\;\mathrm{uniform}(1,000,\;100,000)$ and $N_{2}\;∼\;\mathrm{uniform}(500,\;20,000)$.

Our *ABC* approach follows this procedure:

1. We take the per base average recombination rates of the upstream regions of the 1% ± 0.05% 142 synonymous alleles in the UK10K dataset $\left\{r_{1}^{u},r_{2}^{u},\ldots ,r_{142}^{u}\right\}$ and the per base average recombination rates of the downstream regions $\left\{r_{1}^{d},r_{2}^{d},\ldots ,r_{142}^{d}\right\}$. We also defined the average per base mutation rate $u=\;1.5\;X\;10^{-8}.$
2. Define the frequency f= 1% ± 0.05% of the alleles that will be investigated.
3. Draw a random value from each prior distribution of demographic parameters.
4. Using PReFerSim, simulate 142 random allele frequency trajectories $H_{k}$ where the allele ends at a frequency f=1% ± 0.05% in the present based on the demographic history with the parameter values sampled from the prior distribution. In these simulations we reduced all population sizes and times of population size changes by a factor of five to reduce the computing time. We ended up with a set of allele frequency trajectories $H=\{H_{1},H_{2},\ldots ,H_{142}\}$.
5. Simulate two 250 kb regions with n=72 haplotypes containing the derived allele for each one of the 142 simulated allele frequency trajectories H obtained in the past step using mssel. n is close to the product of 7242*f, where 7242 is the total number of haplotypes in the UK10K dataset. The average per base recombination rates $r_{i}^{u}$ and $r_{i}^{d}$ were used to simulate the two 250 kb regions from each $H_{i}$, where i goes from 1 to 142. The average per base mutation rate $u=\;1.5\;X\;10^{-8}$ was also used. After sampling the u, $r_{i}^{u}$ and $r_{i}^{d}$ values we scaled up their value by a factor of five. After this step, 2*142 datasets with 72 derived haplotypes were simulated. We removed sites where there is a singleton variant in each sample of 72 derived haplotypes, as done in the UK10K data.
6. Calculate $\ell =2\;\times 142\times \left(\begin{array}{c} 72\\ 2 \end{array}\right)$ values of L by taking all possible pairs of haplotypes with the derived allele. Use that distribution of L values to calculate ${D^{'}}_{w_{i}}=P(L\in w_{i})$ for each of the 6 windows $w_{i}$.
7. Calculate $\alpha =\sum_{i=1}^{6}\left|{D^{'}}_{w_{i}}-D_{w_{1}}\right|$
8. Go back to 3) until we have sampled 50,000 values for each demographic parameter from the prior distributions.
9. Retain the 100 simulations where the value of α is smaller.

The values obtained for the parameters in those 100 simulations define the posterior distributions of those parameters. The point estimates of each parameter were defined as the median of their posterior distribution.

The values of α in the 100 retained simulations went from 0.014 to 0.042, indicating that we had a good match between the data and the 100 retained simulations.

### Estimating L taking into account differences in local recombination rates in the *UK10K* dataset and the population expansion scenario

Apart from being dependent on the strength of selection acting on the variants, the distribution of L surrounding each variant on the genome in the *UK10K* data is dependent on the average population scaled recombination rate $\rho =4N\hat{r}l$, where $\hat{r}$ is the average per base recombination rate in a region of length l. We took into account the local recombination rate when inferring the distribution of fitness effects using the 275 non-CpG nonsynonymous 1% frequency variants. To do this, we used our importance sampling method to obtain the distribution of L given the selection coefficient, the inferred demographic scenario, and 21 different recombination rates $\rho^{NS}$. To select the 21 recombination rates, we used the results from a previously inferred recombination map (Kong *et al.* 2010). We took the 21 different percentile values (0^th^, 5^th^, …, 95^th^, 100^th^) from the distribution of 550 average population-scaled recombination rates ρ in the upstream and downstream 250 kb regions next to the 275 nonsynonymous 1% frequency variants. In the end, we generated 21 likelihood functions $L(4Ns,f,D,\rho_{j}^{NS}|L\in w_{i})$ for each selection value explored, each with a different recombination rate $\rho_{j}^{NS}$. Those 21 likelihood functions $L(4Ns,f,D,\rho_{j}^{NS}|L\in w_{i})$ were used to infer selection using the upstream and downstream regions from the nonCpG nonsynonymous 1% frequency variants. They were also used to infer the point estimate of 4Ns in the nonCpG synonymous 1% frequency variants.

First, we evaluated the accuracy of our method to infer selection under the inferred scaled *UK10K* demographic scenario using simulations. We mimicked the amount of information present in the *UK10K* data in each simulation replicate. We followed the protocol detailed in the section *Forward-in-time simulations to assess the impact of selection on the allele frequency trajectories, allele ages, pairwise coalescent times*$T_{2}$, *and*L*values* to generate 100 simulation replicates for 5 different 4Ns values (50, 25, 0, −25, −50) under the 21 different values of recombination $\rho_{j}^{NS}$. In all simulations performed in this section, we used 10,000 trajectories $H_{k}$ where the present-day frequency was equal to f = 1 ±0.05%, and where *n* was equal to f*7,242. The point selection coefficients 4Ns change the distribution of the number of derived alleles that have a frequency in the range f = 1 ±0.05%. As an example of this, 10,161 alleles end with a present-day allele frequency of *n* = 76 derived alleles while 11,617 alleles end with a present-day allele frequency of *n* = 69 derived alleles using 1,700 PReFerSim simulations done using a Poisson mean of 100 new mutations with a 4Ns value equal to −50 in the first epoch. In our simulations of each point selection coefficient 4Ns, we first recorded the number of trajectories $M_{i}$ that ended up with a $n_{i}$ number of derived alleles equal to 69, 70, 71, 72, 73, 74, 75, or 76 in 1,700 PReFerSim simulations done with a Poisson mean number of 100 new mutations in the first epoch of the demographic model. Then, in each simulation replicate we sampled 275 numbers taken from the multinomial distribution with probabilities $p_{i}=M_{i}/\sum_{i=69}^{76}M_{i}$ to obtain 275 numbers reflecting the number of trajectories that have a present-day number of derived alleles $n_{i}$ value equal to 69, 70, 71, 72, 73, 74, 75 or 76 for each 4Ns value. Each simulation replicate contains 275 sets of haplotypes that end with a present-day number of derived alleles $n_{i}$, where each $n_{i}$ value is a sample from the multinomial distribution. In each simulation replicate of 275 independent variants we can define the number of variants $A_{n_{i}}$ where we end up with $n_{i}$ derived alleles in the present and based on that definition we can state that there will be $\ell =2\times \;\sum_{n_{i}=69}^{76}{[A}_{n_{i}}\times \left(\begin{array}{c} n_{i}\\ 2 \end{array}\right)]\approx 2\times \;275\times \left(\begin{array}{c} 72\\ 2 \end{array}\right)$*L* values in each simulation replicate.

We estimated the value of 4Ns in each of those 21 sets of 100 simulation replicates, done with 21 different recombination rates $\rho_{j}^{NS}$ and using 5 different 4Ns values using the likelihood function $L(4Ns,f,D,\rho_{j}^{NS}|L\in w_{i})$ that matches the recombination rate $\rho_{j}^{NS}$ used to do the simulations in each set. Each simulation replicate had $\ell =2\times \;\sum_{n_{i}=69}^{76}{[A}_{n_{i}}\times \left(\begin{array}{c} n_{i}\\ 2 \end{array}\right)]\approx 2\times \;275\times \left(\begin{array}{c} 72\\ 2 \end{array}\right)\;$*L* values (Supplementary Fig. 32). We also performed simulations where the present-day frequency was equal to n=72 (f≈1%; n = 72 chromosomes with the derived allele in a sample of 7,242 chromosomes) for 275 independent variants. We found few differences in terms of the root mean squared errors compared with our simulations where the present-day allele frequency was equal to f = 1 ±0.05% (Supplementary Fig. 32F).

The vast majority of the 550 average recombination rates per base taken from the upstream and downstream 250 kb regions next to the 275 nonsynonymous 1% frequency variants are not equal to the 21 different recombination rates $\rho_{j}^{NS}$. We estimated the likelihood function $L(4Ns,f,D,\rho_{j}^{NS}|L\in w_{i})$ across the 550 regions using the following approach:

For each of the 6 windows $w_{i}=\{w_{1},w_{2},w_{3},w_{4},w_{5},w_{6}\}$:

1. We took the 21 values of $L(4Ns,f,D,\rho_{j}^{NS}|L\in w_{i})$ for each value of $w_{i}$.
2. We fit a polynomial regression model with 5 regression coefficients, with the independent variable being equal to $\rho_{j}^{NS}$ and the dependent variable being equal to $L\left(4Ns,f,D,\rho_{j}^{NS}|L\in w_{i}\right)$.
3. We predicted the value of $L\left(4Ns,f,D,\rho_{j}^{}|L\in w_{i}\right)$ for every one of the 550 average population-scaled recombination rates $\rho_{j}^{}$ in the 550 j regions based on the values of the polynomial regression model.

This approach allows us to estimate the values of $L\left(4Ns,f,D,\rho_{j}^{}|L\in w_{i}\right)$ for the 6 windows $w_{i}$ across the 550 average population-scaled recombination rates $\rho_{j}^{}$. We chose to use 5 regression coefficients based on analysis done with a different number of regression coefficients, and we chose to use the smallest amount of regression coefficients where there was a significant improvement compared with using one less regression coefficient based on an error metric ε (Supplementary Table 12). As an example of the good fit of the polynomial regression model across the 6 windows $w_{i}$, we plotted the predicted values of $L\left(4Ns,f,D,\rho_{j}^{}|L\in w_{i}\right)$ based on the polynomial regression model with 5 regression coefficients when 4Ns=0 (Supplementary Fig. 46). After inspection of the polynomial regression models, we found that the values of $L\left(4Ns,f,D,\rho_{j}^{}|L\in w_{i}\right)$ were predicted to be negative for some windows $w_{i}$ in the recombination rates $\rho^{NS}$ values bigger than the 95% percentile value. This particular effect was not seen in the population expansion scenario. Since $L\left(4Ns,f,D,\rho_{j}^{}|L\in w_{i}\right)$ must be ≤1 and ≥0, we set $L\left(4Ns,f,D,\rho_{j}^{}|L\in w_{i}\right)=1$ for all windows $w_{i}$ in the recombination rates $\rho^{NS}$ values bigger than the 95% percentile value. This is equivalent to stating that in our inferences we will ignore the L values in regions where the recombination rates $\rho^{}$ are bigger than the 95% percentile value from the distribution of 550 average population-scaled recombination rates ρ.

We define the per base average recombination rates of the upstream regions of the 1% ± 0.05% 275 synonymous alleles in the *UK10K* dataset as $\left\{r_{1}^{u},r_{2}^{u},\ldots ,r_{275}^{u}\right\}$ and the per base average recombination rates of the downstream regions $\left\{r_{1}^{d},r_{2}^{d},\ldots ,r_{275}^{d}\right\}$.

We performed 100 simulation replicates with 5 different 4Ns values using 275 variants at a f=1%±0.05% frequency where the average population-scaled recombination rates ρ mimics the values seen in the 550 regions surrounding those variants. The average per base recombination rates $r_{i}^{u}$ and $r_{i}^{d}$ were used to simulate the two 250 kb regions from each of the 275 variants i. We evaluated our approach to estimate selection based on the predicted values of $L\left(4Ns,f,D,\rho_{j}^{}|L\in w_{i}\right)$ for every one of the 550 average population-scaled recombination rates ρ and an analog of Equation (2):

$$L\left(4Ns,D,f|L\right)=\prod_{j=1}^{550}\prod_{i=1}^{\ell_{j}=\left(\begin{array}{c} 72\\ 2 \end{array}\right)}L(4Ns,f,D,\rho_{j}^{}|L_{j_{i}}\in w_{m_{j_{i}}})$$

Our results can be seen on Supplementary Fig. 31. $L_{j_{i}}$ is the pairwise haplotypic identity by state of the haplotype pair i in the recombination rate region *j*.

We also performed 100 simulation replicates with 5 different 4Ns values using 275 variants at a f=1%± 0.05% frequency with each of the 21 population-scaled recombination rates $\rho^{NS}$. We estimated the values of selection on those 100 simulation replicates using the likelihood functions $L\left(4Ns,f,D,\rho_{j}^{NS}|L\in w_{i}\right)$ and Equation (2). The results of this analysis are shown in Supplementary Fig. 32.

We performed 100 simulation replicates under the Boyko distribution of fitness effects and 2 different demographic models (“*UK10K* model” and “scaled *UK10K* model”) using 275 variants at a 1%± 0.05% frequency where the average population-scaled recombination rates ρ mimics the values seen in the 550 regions surrounding those variants. We used the likelihood functions $L\left(4Ns,f,D,\rho_{j}^{}|L\in w_{i}\right)$ in the 550 regions j and an analog of Equation (4) to estimate $L\left(\alpha ,\beta ,D,f|L\right)$ using the collection of lengths $L_{j}=\{L_{j_{1}},L_{j_{2}},L_{j_{3}},\ldots ,L_{j_{\left(\begin{array}{c} 72\\ 2 \end{array}\right)}}\}$ of the 550 regions j:

$$L\left(\alpha ,\beta ,D,f|L\right)=\prod_{j=1}^{550}\prod_{i=1}^{\ell_{j}=\left(\begin{array}{c} 72\\ 2 \end{array}\right)}L\left(\alpha ,\beta ,f,D,\rho_{j}^{}|L_{j_{i}}\in w_{m_{j_{i}}}\right).$$

Finally, we used the likelihood functions $L(\alpha ,\beta ,D,f,\rho_{j}^{}|L\in w_{m_{j_{i}}})$ from the 550 regions to obtain an estimate of the parameters that define the $DFE_{f}$ of the 1%±0.05% frequency variants in the *UK10K* dataset. We also obtained an estimate of those parameters in 100 bootstrap replicates.

### Bootstrap confidence intervals

We used a bootstrap approach to estimate the 95% confidence intervals of our estimate of the selection coefficient *s*. We resampled each of the 275 variants with replacement, with their respective L values, and we estimated the value of selection using this distribution of *L* values. This process was repeated using a sample of 100 bootstrap replicates.

We used the same bootstrap approach to estimate the shape and scale parameters of a compound distribution of fitness effects. The variation across 100 bootstrap replicates is shown in Supplementary Fig. 42.

### Estimation of $P_{\psi }(f|D)$ in the *UK10K* dataset

To estimate $P_{\psi }(f|D)$, we used Equation (A14) where

Numerator: the number of nonsynoymous f=1%±0.05 frequency non-CpG variants that are more than 5 Mb away from centromeres or telomeres. This is 275.

Denominator: we calculated the total number of mutations in the *UK10K* model (see Supplementary Fig. 19), including the alleles that became fixed or extinct from the population. Following Equation (A15), the total number of mutations that appear each generation in our demographic scenario is equal to:$\;2N_{g}ul$. Where $N_{g}$ is the effective population size in that generation. l=35,086,455 is the total number of non-CpG nonsynonymous possible variants that are more than 5 Mb away from centromeres or telomeres (35,086,455). We used the Ensembl 75 Variant Effect Predictor 93.3 to aid in our calculation of l. A variant was annotated as nonsynonymous if it possessed a nonsynonymous annotation in any of the transcripts inspected and it did not possess a variant that gave a “high” impact in any transcript as explained in https://www.ensembl.org/info/genome/variation/prediction/predicted_data.html. Variants with a high impact are those annotated to be transcription ablation, splice acceptor variants, splice donor variants, stop gained, frameshift, stop lost, start lost, or transcript amplification. u is the mutation rate, which we set to 1.5 × 10^−8^ (Ségurel *et al.* 2014). We sum the total number of mutations across all the generations in the *UK10K* model to obtain the denominator of Equation (A14).

We show our estimates of $P_{\psi }\left(s_{j}\right)$ over 3 different $s_{j}$ intervals in Fig. 9 using the estimate of $P_{\psi }(f|D)$ described above. We compare our estimate of $P_{\psi }\left(s_{j}\right)$ with previous estimates (Boyko *et al.* 2008; Kim *et al.* 2017).
