# Supplementary material for: Haplotype-based inference of the distribution of fitness effects
Source: Genetics. 2022 Jan 9;220(4):iyac002. doi: 10.1093/genetics/iyac002 (PMC8982047; doi:10.1093/genetics/iyac002)
Supplement: iyac002_Supplementary_Data [file iyac002_supplementary_data.pdf]

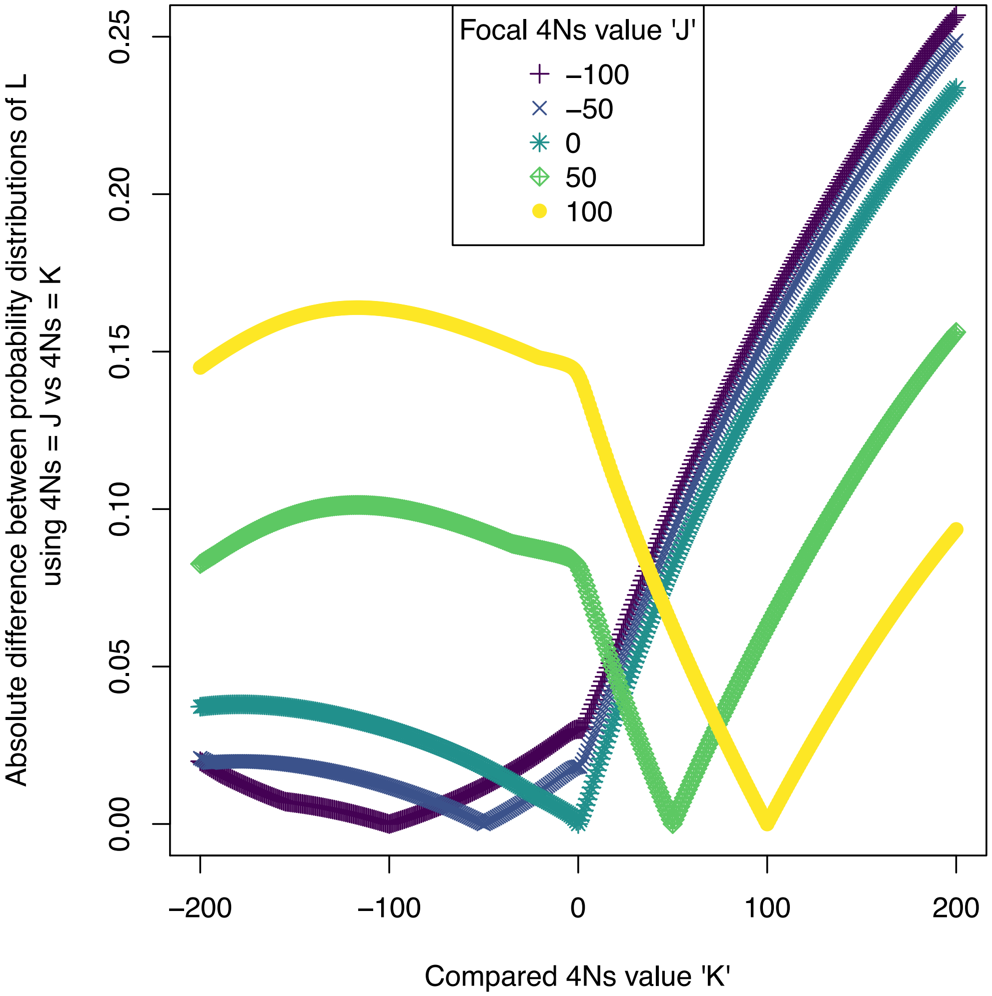

Figure S1

Changes in the probability distribution of $P(L \in w_i|f, D, 4Ns)$ after varying the value of $4Ns$ in a population expansión model. We estimate this distribution using Equation 1.

Y axis label.- Absolute difference between probability distributions of L using $4Ns = J$ vs $4Ns = K$. This is calculated as $Dif = \sum_{i=1}^{6} |P(L \in w_i|f, D, 4Ns = J) - P(L \in w_i|f, D, 4Ns = K)|$.

We see that Dif varies more when doing comparisons to a focal $4Ns$ value J equal to 50 and 100 for $4Ns = K$ positive values than when using focal $4Ns$ values J equal to -50 and -100 and negative $4Ns = K$ values. This observation is pertinent to the resuts seen in Figure 5, where we observe that the inferences of $4Ns$ values in variants with negative $4Ns$ values have a wider distribution over a set of negative $4Ns$ values than the inferences of $4Ns$ in variants with positive $4Ns$ values over a set of positive $4Ns$ values. When we try to estimate the value of $4Ns$ of a deleterious variant we see wider distributions because the distribution $P(L \in w_i|f, D, 4Ns = J)$ varies less over the inspected negative $4Ns$ numbers.

We also observe that the distribution $P(L \in w_i|f, D, 4Ns)$ is more similar for neutral variants with the distribution $P(L \in w_i|f, D, 4Ns)$ of deleterious variants compared to the distribution $P(L \in w_i|f, D, 4Ns)$ of advantageous variants. This also explains why we see some estimates having a wider variation towards deleterious variants in simulations done with neutral variants.

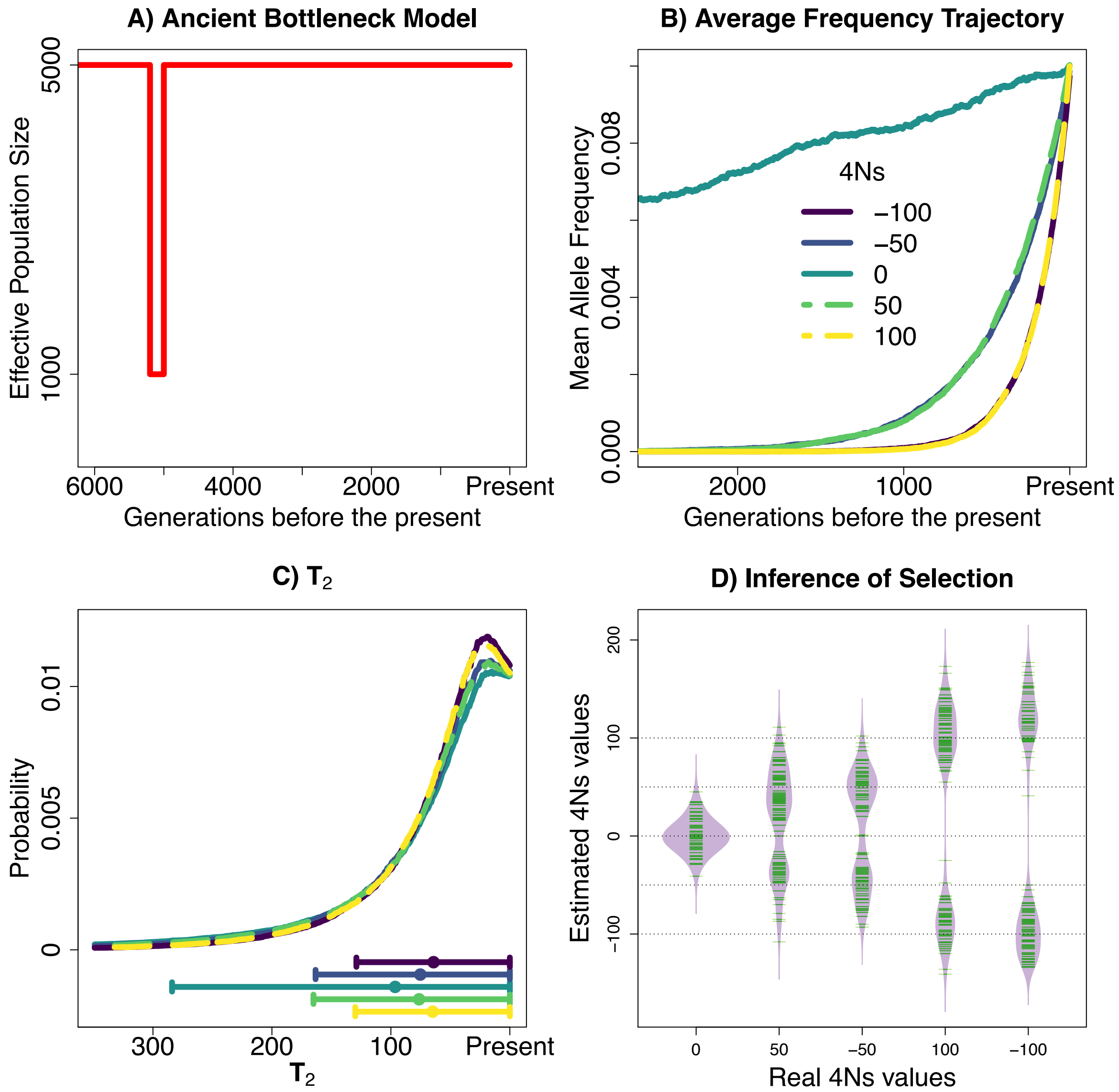

Figure S2

Properties of alleles sampled at a present-day 1% frequency under different strengths of natural selection in a scenario with a bottleneck that took place 5,000 generations ago

A) Demographic model analyzed. B) Mean allele frequency at different times in the past, in units of generations, using 10,000 allele frequency trajectories. C) Probability distribution of pairwise coalescent times T_2. The dot and whiskers represent the mean value of the distribution and the two whiskers extend at both sides of the mean until max(mean +- s.d. ,0).and D) Estimation of the strength of selection using 100 simulation replicates for each $\mathbf{4}Ns$ value analyzed. Each simulation replicate has $\mathbf{2} \times \boldsymbol{A} \times \binom{\boldsymbol{n}}{\mathbf{2}} = \mathbf{2} \times \mathbf{300} \times \binom{\mathbf{40}}{\mathbf{2}} = \mathbf{468,000}\ \boldsymbol{L}$ values. The green lines in D) indicate estimated values of $\mathbf{4}Ns$. We used $\boldsymbol{u} = \mathbf{1.2}\ \boldsymbol{X}\ \mathbf{10^{-8}}$, $\boldsymbol{r} = \mathbf{1.0}\ \boldsymbol{X}\ \mathbf{10^{-8}}$ and $\boldsymbol{l} = \mathbf{1}$ Mb to create the simulated regions. We chose to simulate a region of 1 Mb with the focal allele in the middle of the region because we saw that with this region length we had on average more than 5% of the $\boldsymbol{L}$ values falling in each of the 6 windows $\boldsymbol{W} = \{\boldsymbol{w_1}, \boldsymbol{w_2}, \boldsymbol{w_3}, \boldsymbol{w_4}, \boldsymbol{w_5}, \boldsymbol{w_6}\}$= *{ (0, 100000], (100000, 200000], (200000, 300000], (300000, 400000], (400000, 500000], (500000, ∞)}* for the 5 $\mathbf{4}Ns$ values inspected.

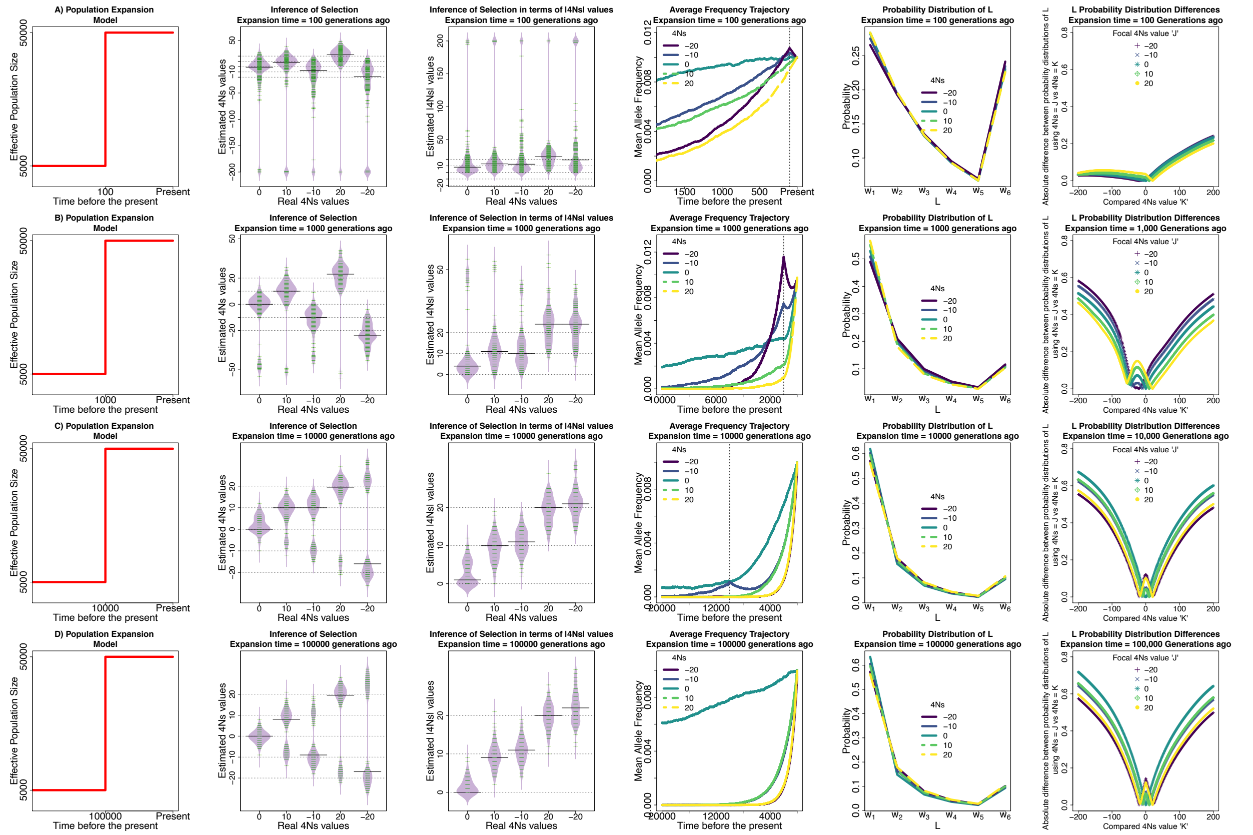

Figure S3

Estimation of the strength of natural selection in 4 population expansion models for 1% frequency alleles

The difference between the four inspected models lies in the population expansion time. We used a population expansion time that took place A) 100, B) 1000, C) 10000 and D) 100000 generations ago. Each simulation replicate contained $\mathbf{2 \times A \times \binom{n}{2} = 2 \times 300 \times \binom{40}{2} = 468{,}000}$ realized values of $\boldsymbol{L}$. The green lines indicate one estimated value of $\boldsymbol{4Ns}$. 'Real $\boldsymbol{4Ns}$ values' indicate the $\boldsymbol{4Ns}$ values used in the simulations and 'Estimated $\boldsymbol{4Ns}$ values' or 'Estimated $|\boldsymbol{4Ns}|$ values' refers to the $\boldsymbol{4Ns}$ or $|\boldsymbol{4Ns}|$ values estimated by our method. The median value of the estimates of $\boldsymbol{4Ns}$ is shown with a solid line. The estimated $\boldsymbol{4Ns}$ and $|\boldsymbol{4Ns}|$ values for each demographic model are shown, respectively, in the two plots at the right side of each defined demographic model. We also obtained $10{,}000$ frequency trajectories for $\boldsymbol{f} = 1\%$ frequency alleles under different strengths of selection using *PReFerSim* forward-in-time simulations under the *PRF* model (Ortega-Del Vecchyo, Marsden, and Lohmueller 2016). We used those frequency trajectories to calculate the mean allele frequency at different times in the past, in units of generations, to obtain an average frequency trajectory for each value of selection in each of the 4 demographic models. We found that when the expansion happened 100 or 1000 generations ago, we can obtain an estimate of $4\mathrm{Ns}$ that appears to be unbiased. When the expansion time took place $10{,}000$ or $100{,}000$ generations ago, we saw that the estimates of $4\mathrm{Ns}$ cluster around values of the same magnitude of the $4\mathrm{Ns}$ values used in simulations (i.e. estimated values of $4\mathrm{Ns}$ cluster close to 10 or -10 when the simulations used a $4\mathrm{Ns}$ value of 10). We saw that the estimates of $|4\mathrm{Ns}|$ appear to be unbiased when the population expansion took place $10{,}000$ or $100{,}000$ generations. The estimates of $4\mathrm{Ns}$ and $|4\mathrm{Ns}|$ when the population expansion happened $10{,}000$ or $100{,}000$ generations ago resemble the results found when the population has a constant size (Figure 3). An inspection of the mean allele frequency trajectories sheds more light into our results. The mean allele frequency trajectory is different for every inspected value of selection in scenario A) and B) which is reflected on our capacity to obtain reasonable estimates of $4\mathrm{Ns}$. On the other hand, the mean allele frequency trajectory for $4\mathrm{Ns}$ values that have the same absolute strength of

selection is remarkably similar in D). In the case of C) the mean allele frequency trajectory is similar for simulations conducted with a $|4\mathrm{Ns}|$ value of 20. That trajectory is more dissimilar for times older than $4{,}000$ generations ago in the case of simulations with a $|4\mathrm{Ns}|$ value of 10 in C). However, this dissimilarity in ancient times for the trajectory is not sufficient to obtain a difference in L values, seen in the fifth column of the Figure on the plots of the probability distribution of L obtained from the simulation of $\mathbf{100} \times \mathbf{2} \times \boldsymbol{A} \times \binom{\boldsymbol{n}}{\mathbf{2}} = \mathbf{2} \times \mathbf{300} \times \binom{\mathbf{40}}{\mathbf{2}} = \mathbf{468,000}$ values of $\boldsymbol{L}$.

We note that there is a bimodal distribution of inferred values under the model presented in A), where the population expansion took place 100 generations ago. In this scenario some inferred estimates are equal to -200 while others are close to the true $4\mathrm{Ns}$ value (see second column). This effect is not present on the other three demographic models. The explanation behind this phenomenon is seen in the probability distribution $P(L \in w_i|f, D, 4Ns)$ estimated using Equation 1 (last column). The difference between the distribution $P(L \in w_i|f, D, 4Ns)$ of deleterious mutations and the distribution $P(L \in w_i|f, D, 4Ns = x)$ for the focal $4Ns = x$ values [-20, -10, 0, 10, 20] is much larger when the population expansion took place 1000, 10000 and 100000 generations ago compared to the demographic model where the expansion happened 100 generations ago. In particular, the similarity between $P(L \in w_i|f, D, 4Ns = -200)$ and $P(L \in w_i|f, D, 4Ns = x)$ where x = [-20, -10, 0, 10, 20] makes it possible to infer that the selection coefficient is equal to $4\mathrm{Ns} = -200$ on some simulations with a slight change on the distribution of L values on each simulation replicate performed with a $4\mathrm{Ns}$ value equal to -20, -10, 0, 10 and 20 in the model presented in A).

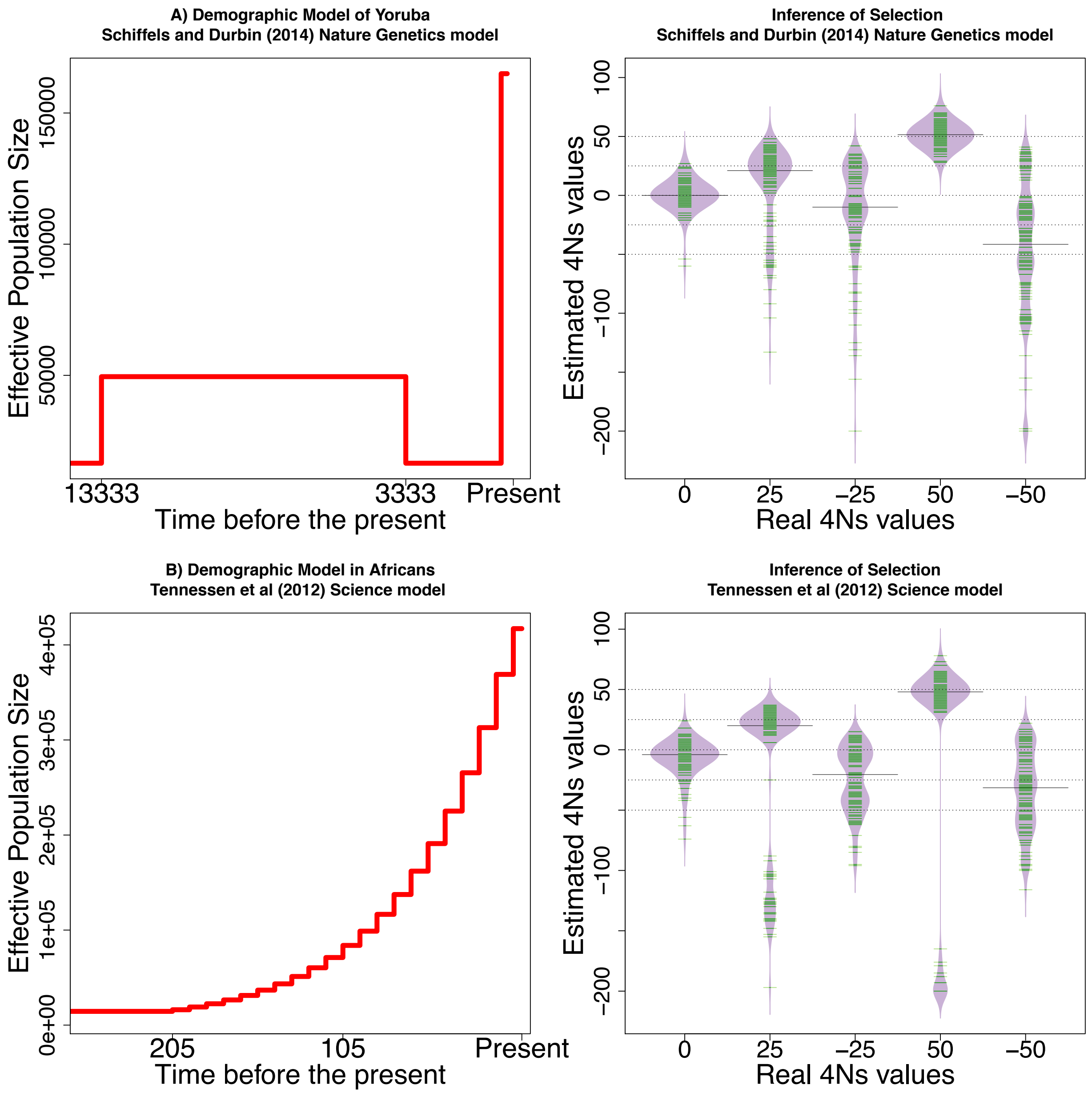

Figure S4.- Inferences of selection under two demographic models: A) The Schiffels and Durbin (2014) Nature Genetics model of the YRI population and B) The Tennessen et al (2012) Science model demographic model of Africans.

The model for the YRI population was presented by Schiffels and Durbin (2014) and mimics the population size changes observed in that population. The Tennessen et al (2012) includes a parameter where the population grows exponentially starting 205 generations ago. We average the population sizes in the original Tennessen et al (2012) model over intervals of 10 generations and use that model (Figure S5B) to perform our inferences. The Tennessen et al (2012) also includes an ancestral expansion from from 7310 to 14474 individuals that took place 5920 generations ago.

We chose to simulate a region of 200 kb and 100 kb with the focal allele in the middle of the region for the Schiffels and Durbin (2014) and Tennessen et al (2012) model, respectively. We saw that with this region length we had on average more than 5% of the $\boldsymbol{L}$ values falling in each of the six windows $\boldsymbol{W} = \{\boldsymbol{w_1}, \boldsymbol{w_2}, \boldsymbol{w_3}, \boldsymbol{w_4}, \boldsymbol{w_5}, \boldsymbol{w_6}\}$= *{ (0, 20000], (20000, 40000], (40000, 60000], (60000, 80000], (80000, 100000], (100000, ∞)}* for the 5 $\boldsymbol{4Ns}$ values inspected in the Schiffels and Durbin (2014) model. We also had on average more than 5% of the $\boldsymbol{L}$ values falling in each of the six windows windows $\boldsymbol{W}$ =

$\{\boldsymbol{w_1}, \boldsymbol{w_2}, \boldsymbol{w_3}, \boldsymbol{w_4}, \boldsymbol{w_5}, \boldsymbol{w_6}\}$= *{ (0, 10000], (10000, 20000], (20000, 30000], (30000, 40000], (40000, 50000], (50000, ∞)}* for the 5 $\boldsymbol{4Ns}$ values inspected in the Tennessen et al (2014) model

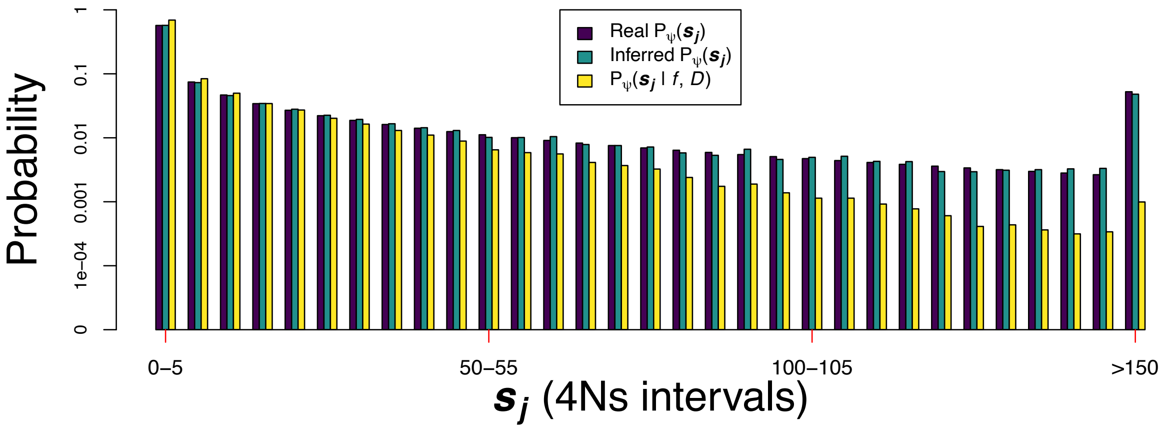

Figure S5

Inference of the distribution of fitness effects of new mutations from the distribution of fitness effects of variants at a certain frequency in deleterious variants.

The DFE follows a gamma distribution with shape and scale parameters equal to 0.184 and 79.966, respectively. The inferences were performed under a population expansion demographic scenario where the population grows from $5{,}000$ to $50{,}000$ individuals in the last 100 generations (see also Figure 4A). 'Real $P_{\psi}(\boldsymbol{s_j})$' refers to the probability of having a $4Ns$ value in a certain interval $\boldsymbol{s_j}$ given the distribution of fitness effects of new mutations with parameters ψ. $P_{\psi}(\boldsymbol{s_j}|f, D)$ is the probability of having a $4Ns$ value in an interval $\boldsymbol{s_j}$ given the distribution of fitness effects DFE with parameters ψ and the demographic scenario D in 1% frequency variants. We calculated $P_{\psi}(\boldsymbol{s_j}|f, D)$ from a set of $41{,}298$ $4Ns$ 1% variants obtained via *PReFerSim* simulations under the DFE and the population expansion scenario. 'Inferred $P_{\psi}(\boldsymbol{s_j})$' is an estimate of the probability of having a $4Ns$ value in a certain interval $\boldsymbol{s_j}$ given the distribution of fitness effects of new mutations. That probability was estimated using $P_{\psi}(\boldsymbol{s_j}|f, D)$ and Equation 6. The selection coefficient s refers exclusively to the action of deleterious variants in this plot.

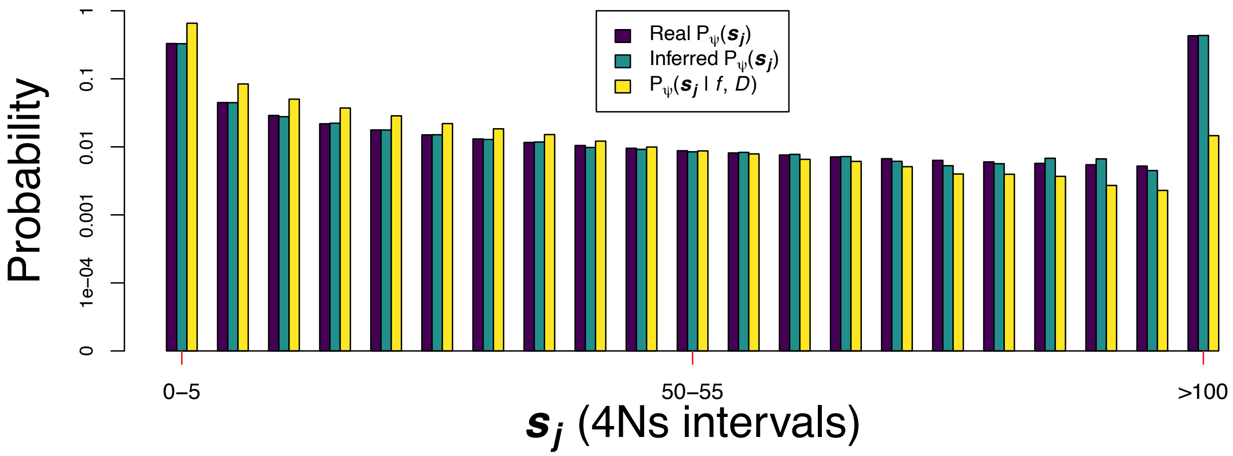
Real $P_\psi(\boldsymbol{s_j})$
Inferred $P_\psi(\boldsymbol{s_j})$
$P_\psi(\boldsymbol{s_j} \mid f, D)$
Probability
1
0.1
0.01
0.001
1e−04
0
0–5
50–55
>100
$\boldsymbol{s_j}$ (4Ns intervals)

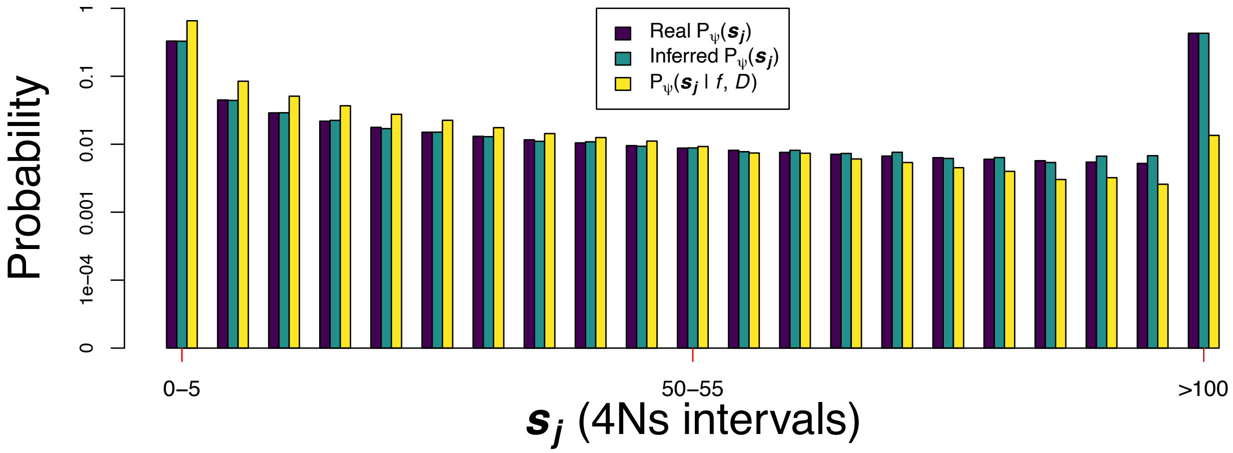
Real $P_\psi(\boldsymbol{s_j})$
Inferred $P_\psi(\boldsymbol{s_j})$
$P_\psi(\boldsymbol{s_j} \mid f, D)$
Probability
1
0.1
0.01
0.001
1e−04
0
0–5
50–55
>100
$\boldsymbol{s_j}$ (4Ns intervals)

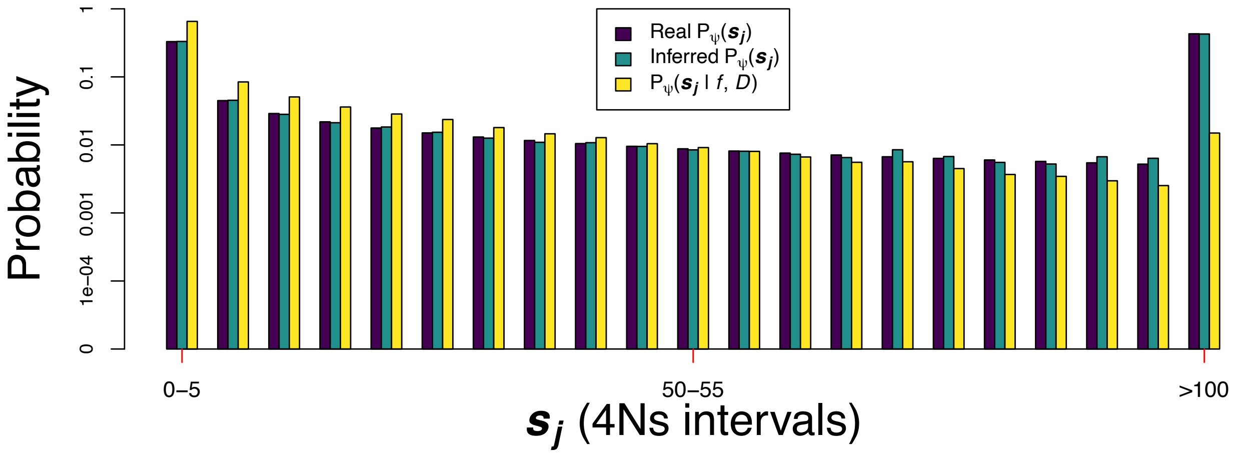
Real $P_\psi(\boldsymbol{s_j})$
Inferred $P_\psi(\boldsymbol{s_j})$
$P_\psi(\boldsymbol{s_j} \mid f, D)$
Probability
1
0.1
0.01
0.001
1e−04
0
0–5
50–55
>100
$\boldsymbol{s_j}$ (4Ns intervals)

Figure S6

Estimated and actual values of $P_\psi(\boldsymbol{s_j})$ on a population expansion model.

The DFE follows a gamma distribution with shape and scale parameters equal to 0.184 and 1599.313, respectively. This is equal to the gamma distribution inferred by Boyko et al. (2008) after adjusting the population sizes to the population expansion demographic model used. The only difference between the three plots shown here is the number of generations simulated in the first epoch, where there were $5{,}000$ individuals. We used $80000,$ 100000 and 120000 generations for the first epoch in the upper, middle and lower panels, respectively. That first epoch was followed by a population expansion to $50{,}000$ individuals that lasted for 100 generations.

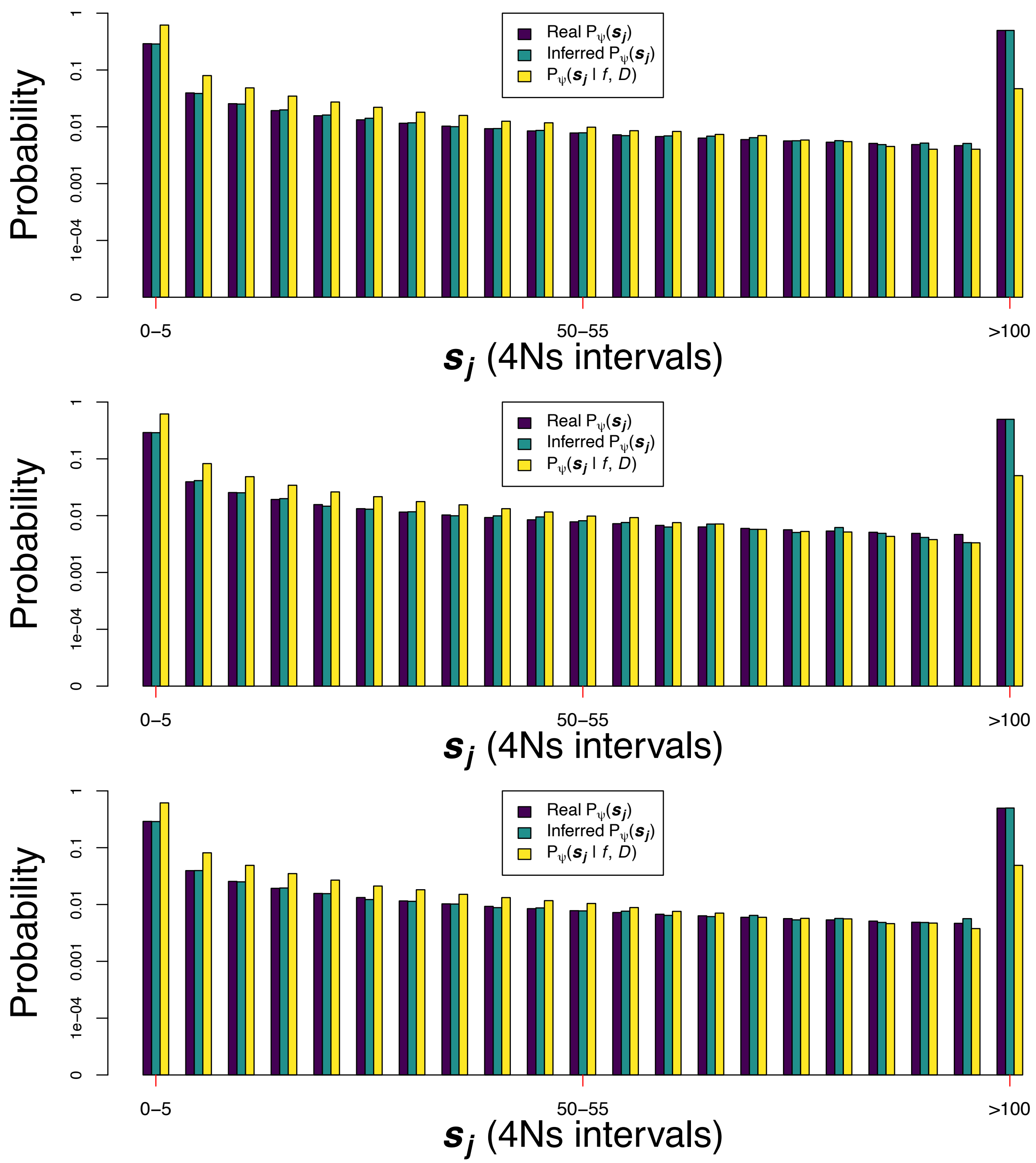

Figure S7

Estimated and actual values of $P_\psi(s_j)$ on a constant population size demographic model.

The DFE follows a gamma distribution with shape and scale parameters equal to 0.184 and 3198.626, respectively. This is equal to the gamma distribution inferred by Boyko et al. (2008) after adjusting the population sizes to the constant population size demographic model used. The only difference between the three plots is the number of generations simulated. We used 160000, 180000 and 200000 generations as the number of generations run in the demographic model in the upper, middle and lower panels, respectively.

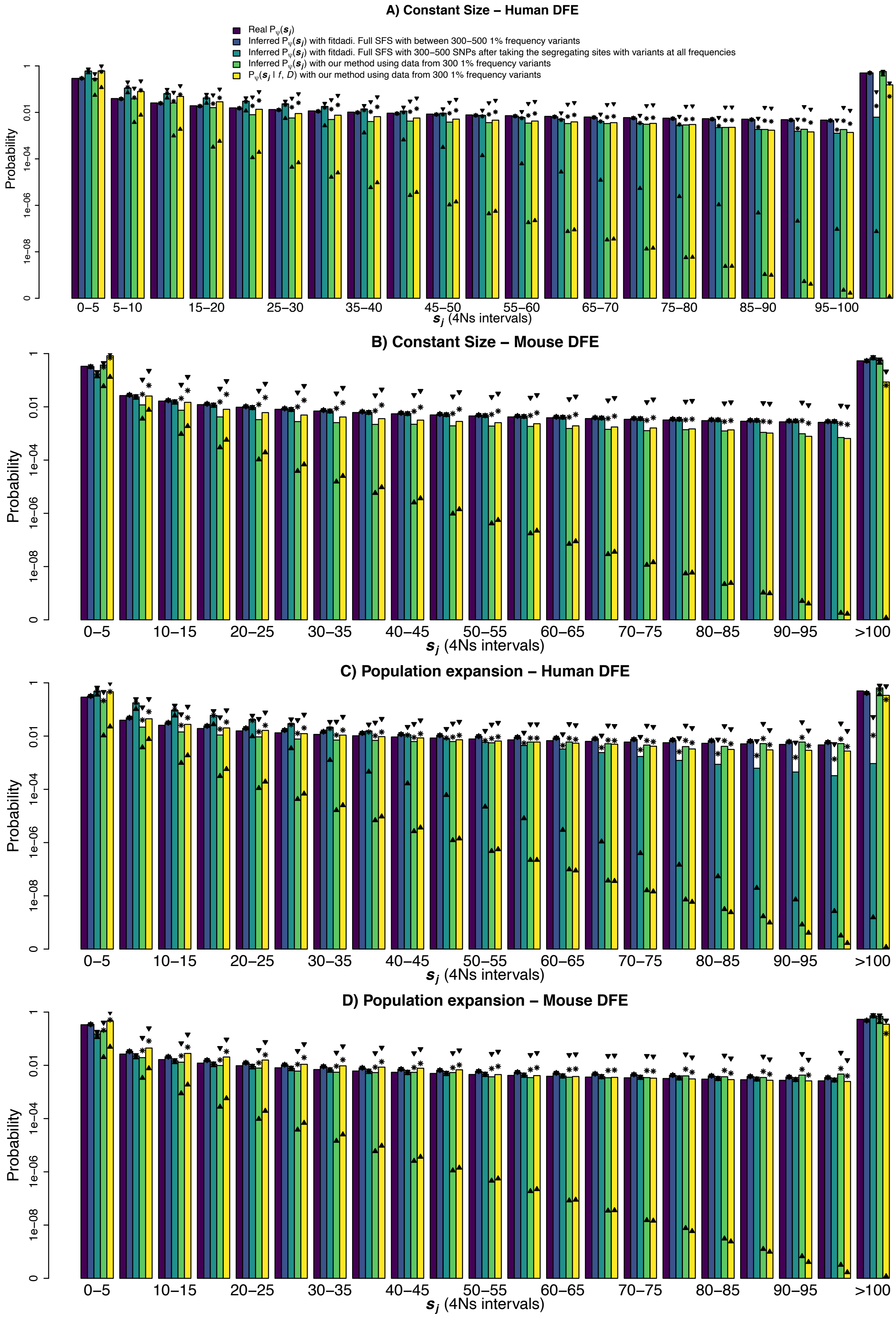

A) Constant Size – Human DFE
Real $P_\psi(s_j)$
Inferred $P_\psi(s_j)$ with fitdadi. Full SFS with between 300–500 1% frequency variants
Inferred $P_\psi(s_j)$ with fitdadi. Full SFS with 300–500 SNPs after taking the segregating sites with variants at all frequencies
Inferred $P_\psi(s_j)$ with our method using data from 300 1% frequency variants
$P_\psi(s_j \mid f, D)$ with our method using data from 300 1% frequency variants
Probability
s_j (4Ns intervals)
B) Constant Size – Mouse DFE
C) Population expansion – Human DFE
D) Population expansion – Mouse DFE

149

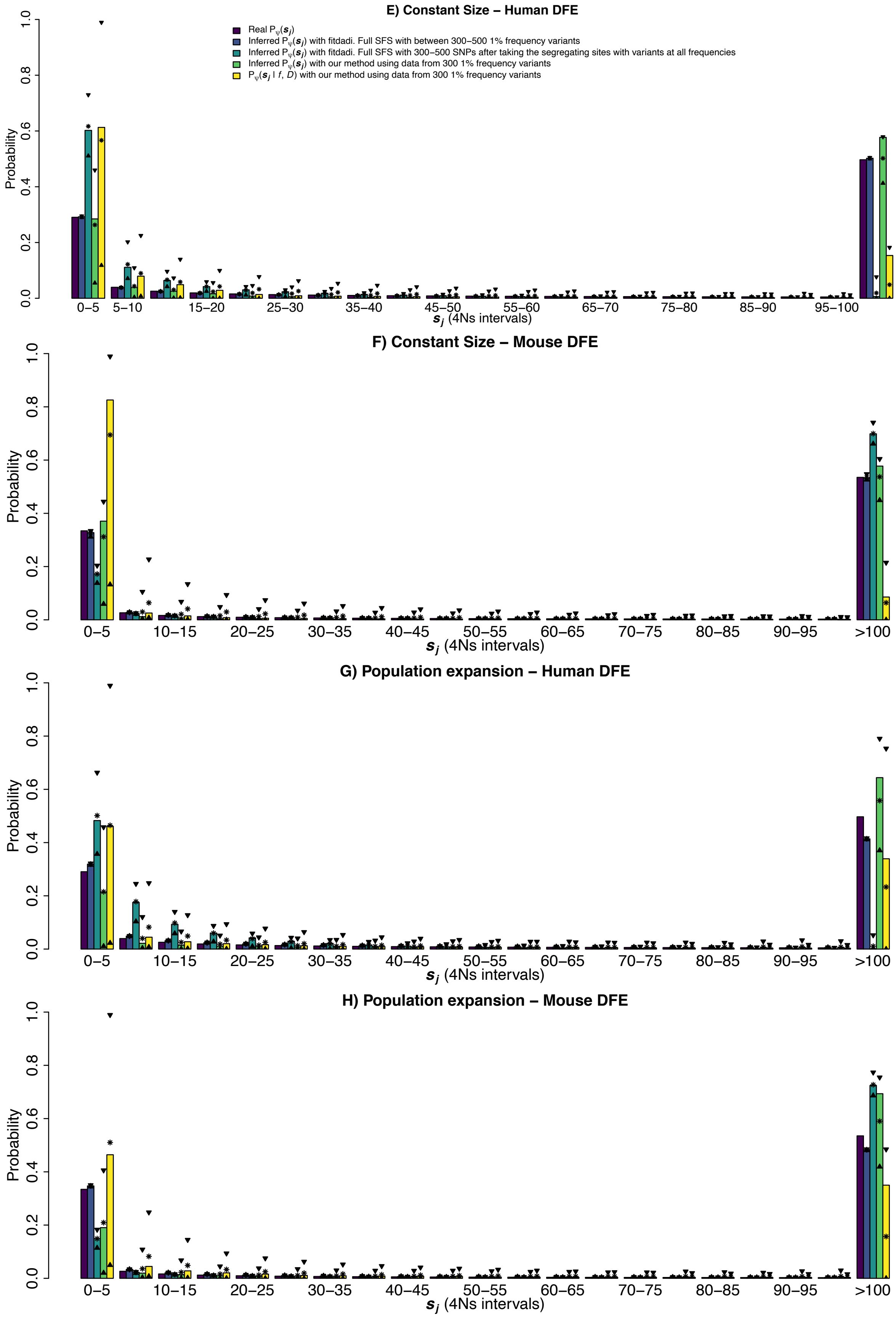
E) Constant Size – Human DFE
Real $P_\psi(s_j)$
Inferred $P_\psi(s_j)$ with fitdadi. Full SFS with between 300–500 1% frequency variants
Inferred $P_\psi(s_j)$ with fitdadi. Full SFS with 300–500 SNPs after taking the segregating sites with variants at all frequencies
Inferred $P_\psi(s_j)$ with our method using data from 300 1% frequency variants
$P_\psi(s_j \mid f, D)$ with our method using data from 300 1% frequency variants
Probability
0.0
0.2
0.4
0.6
0.8
1.0
0–5
5–10
15–20
25–30
35–40
45–50
55–60
65–70
75–80
85–90
95–100
s_j (4Ns intervals)
F) Constant Size – Mouse DFE
Probability
0–5
10–15
20–25
30–35
40–45
50–55
60–65
70–75
80–85
90–95
>100
s_j (4Ns intervals)
G) Population expansion – Human DFE
Probability
0–5
10–15
20–25
30–35
40–45
50–55
60–65
70–75
80–85
90–95
>100
s_j (4Ns intervals)
H) Population expansion – Mouse DFE
Probability
0–5
10–15
20–25
30–35
40–45
50–55
60–65
70–75
80–85
90–95
>100
s_j (4Ns intervals)

Figure S8

Inference of the distribution of fitness effects of new mutations from the distribution of fitness effects of variants in $\mathbf{1\%}$ frequency variants.

'Real $P_\psi(\boldsymbol{s_j})$' is the proportion of variants in a certain $\boldsymbol{s_j}$ interval based on the parameters ψ that define the distribution of fitness effects of new variants DFE.

'Inferred $P_\psi(\boldsymbol{s_j})$ with fitdadi. Full SFS with between 300−500 1% frequency variants' and 'Inferred $P_\psi(\boldsymbol{s_j})$ with fitdadi. Full SFS with 300−500 SNPs after taking the segregating sites with variants at all frequencies' represent inferences performed on 100 simulation replicates with the program fitdadi (Kim et al., 2017). To do this, we fitted a gamma distribution and estimated the shape and scale parameters that better explain the distribution of fitness effects given a known demographic model using data from the site frequency spectrum. Each DFE was defined as an integrable function over a log-spaced range of 600 selection coefficients over intervals from 10^{-8} to 0.5 following Kim et al., 2017. In the case of 'Inferred $P_\psi(\boldsymbol{s_j})$ with fitdadi. Full SFS with between 300−500 1% frequency variants' each simulation replicate contains the SFS computed from simulations performed with *PReFerSim* (Ortega-Del Vecchyo et al., 2016) using an initial $\theta = 1000.0$ until we obtained at least 300 1% frequency variants in a sample size of 4,000 chromosomes under each DFE and demographic scenario. In the case of 'Inferred $P_\psi(\boldsymbol{s_j})$ with fitdadi. Full SFS with 300−500 SNPs after taking the segregating sites with variants at all frequencies' each simulation replicate contains the SFS computed from a simulation performed with *PReFerSim* (Ortega-Del Vecchyo et al., 2016) using an initial θ value chosen to obtain between 300 to 500 SNPs in each simulation in a sample size of 4,000 chromosomes under each DFE and demographic scenario.

The bars on '$P_\psi(\boldsymbol{s_j}|f,D)$ with our method using data from 300 1% frequency variants' and 'Inferred $P_\psi(\boldsymbol{s_j})$ with our method using data from 300 1% frequency variants' represent the median value of those respective estimated probabilities across 100 simulation replicates, where each simulation replicate employs $2 \times A \times \binom{n}{2} = 2 \times 300 \times \binom{40}{2} = 468{,}000\ L$ values. The two triangles shown in each $\boldsymbol{s_j}$ interval denote the 5% and 95% percentile of the '$P_\psi(\boldsymbol{s_j}|f,D)$ with our method using data from 300 1% frequency variants' and 'Inferred $P_\psi(\boldsymbol{s_j})$ with our method using data from 300 1% frequency variants' probabilities estimated across 100 simulation replicates. The mean values of '$P_\psi(\boldsymbol{s_j}|f,D)$ with our method using data from 300 1% frequency variants' and 'Inferred $P_\psi(\boldsymbol{s_j})$ with our method using data from 300 1% frequency variants' from 100 simulations replicates are shown with an asterisk.

We estimated the values of 'Inferred $\boldsymbol{P_\psi}(\boldsymbol{s_j})$ with our method using data from 300 1% frequency variants' using equation (6). The following terms were calculated to use equation (6) (Also see '*Connecting the distribution of fitness effects of variants at a particular frequency ($\boldsymbol{DFE_f}$) with the distribution of fitness effects of new mutations ($\boldsymbol{DFE}$)*'):

$P_\psi(\boldsymbol{s_j}|f,D)$.- Comes from the inferences of $DFE_f(\alpha,\beta)$ taken from Figure 6.

$P_\psi(f|D)$.- Calculated from 2,500 simulations done using *PReFerSim* under each demographic scenario and DFE. The number of new mutations appearing each generation follows a Poisson distribution with a mean equal to $2N_iul = 1{,}000$ in the ancestral epoch in each simulation, where N_i is the population size in generation i.

$P_\psi(f|\boldsymbol{s_j},D)$.- Calculated from 2,500 simulations done using *PReFerSim* under each demographic scenario. The number of new mutations appearing each generation follows a Poisson distribution with a mean equal to $2N_iul = 1{,}000$ in the ancestral epoch in each simulation, where N_i is the population size in generation i. The mean of the Poisson distribution changes between epochs as defined by the N_i values. On the inferences done in 6A) and 6C) of the Human DFE we performed the simulations to estimate $P_\psi(f|\boldsymbol{s_j},D)$ with the Mouse DFE. On the other hand, on the inferences done in 6B) and 6D) of the Mouse DFE we performed the simulations to estimate $P_\psi(f|\boldsymbol{s_j},D)$ with the Human DFE. The DFE used to perform the simulations to calculate $P_\psi(f|\boldsymbol{s_j},D)$ is arbitrary as long as the DFE encompasses all the intervals $\boldsymbol{\sigma} = \{\boldsymbol{s_1},\boldsymbol{s_2},\boldsymbol{s_3},\ldots,\boldsymbol{s_b}\}$ analyzed. Here we decided to use a DFE to calculate $P_\psi(f|\boldsymbol{s_j},D)$ that is different from the DFE we are using to simulate and estimate the values of 'Real $P_\psi(\boldsymbol{s_j})$' in each plot.

The figures A) to D) show the results plotted in a log scale while the figures E) to H) show the results plotted in a normal scale.

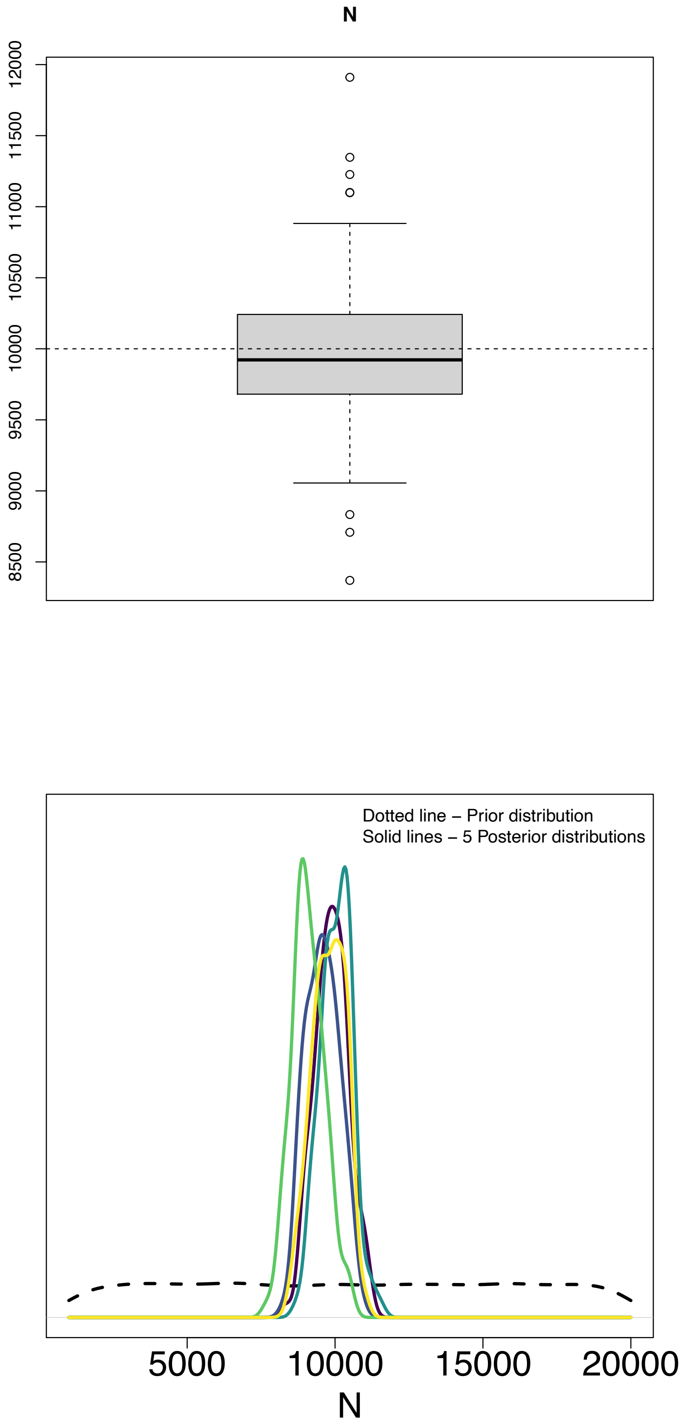

Figure S9

Demographic inference under the constant population size model.

Upper panel – Estimated N values under the constant population size model using the *ABC* approach in 100 simulations. The dotted line denotes the actual value of *N*.

Lower panel – Prior distribution of the N values used in the *ABC* approach along with the posterior distribution in 5 different simulations.

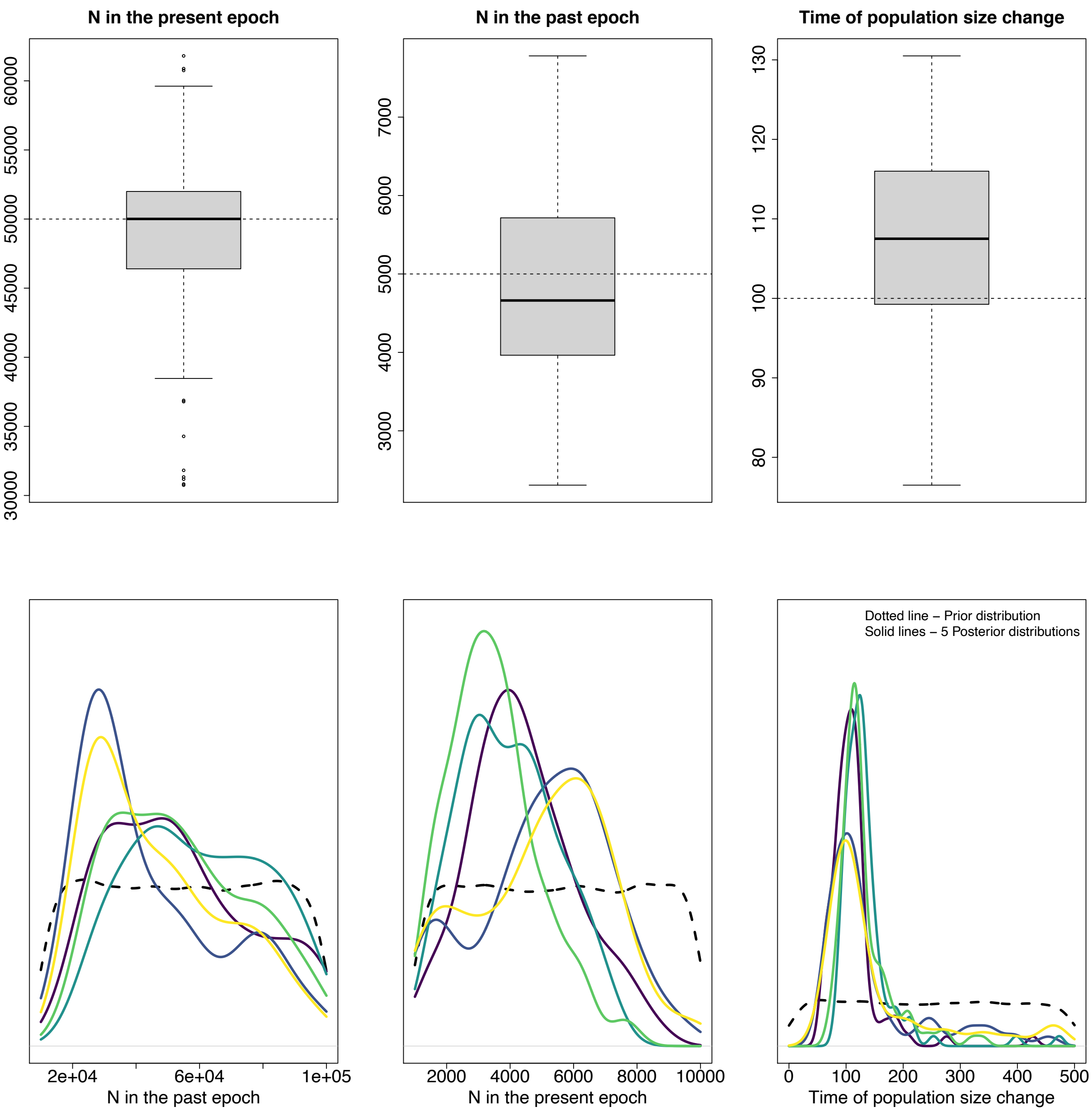

Figure S10

Demographic inference under the population expansion model.

This model has three parameters: The effective population size in the ancient epoch (N in the past epoch), the effective population size in the current-day epoch (N in the present epoch) and the time when the population size changes (Time of population size change).

Upper panel – Estimated values of the parameters in 100 simulations performed using the *ABC* approach. The dotted line denotes the actual value of the parameters.

Lower panel – Prior distribution of the parameter values used in the *ABC* approach along with the posterior distribution in 5 different simulations.

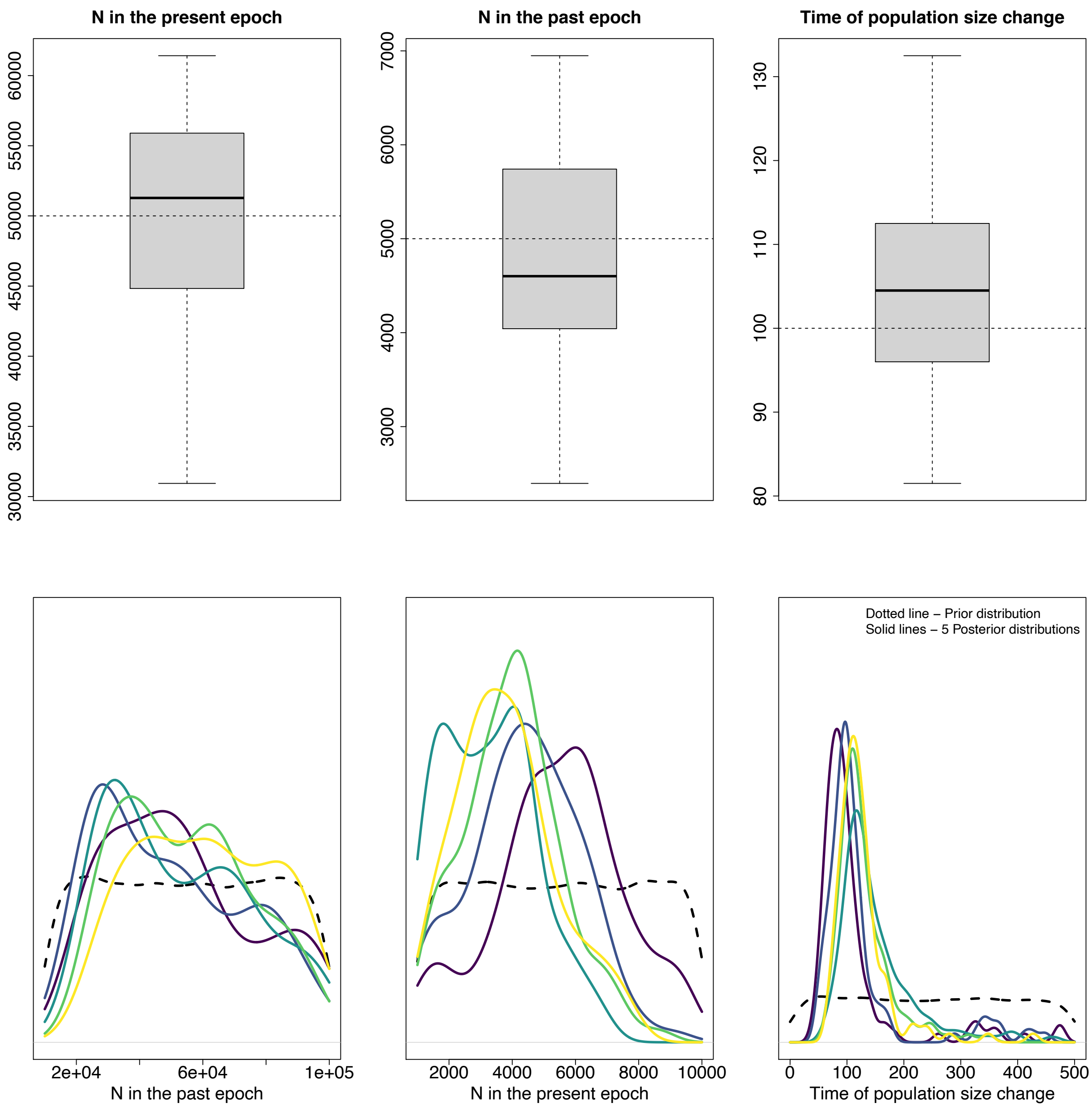

Figure S11

Demographic inference under the population expansion model with 150 variants with a different recombination rate sampled with replacement from the distribution of the 142 average recombination rates per base using the 250 kb upstream and downstream region surrounding each of the 142 *1%* synonymous variants of the *UK10K* dataset.

This model has three parameters: The effective population size in the ancient epoch (N in the past epoch), the effective population size in the current-day epoch (N in the present epoch) and the time when the population size changes (Time of population size change).

Upper panel – Estimated values of the parameters in 100 simulations performed using the *ABC* approach. The dotted line denotes the actual value of the parameters.

Lower panel – Prior distribution of the parameter values used in the *ABC* approach along with the posterior distribution in 5 different simulations.

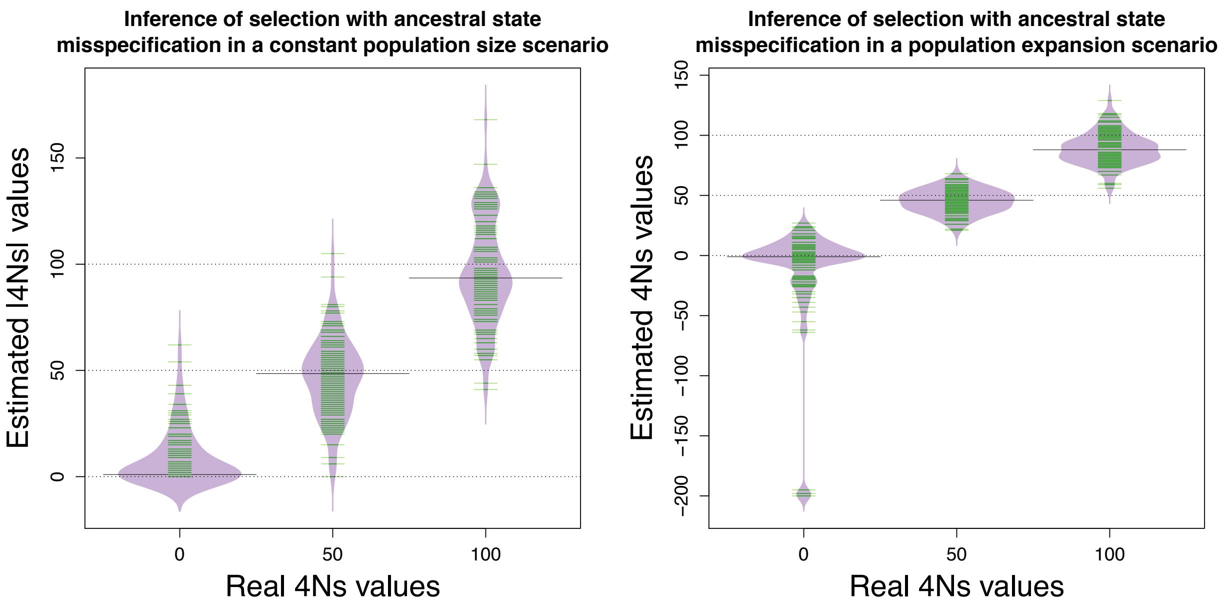

Figure S12

Inference of selection in two demographic scenarios including ancestral state misspecification. Each simulation replicate contained $2 \times A \times \binom{n}{2} = 2 \times 300 \times \binom{40}{2} = 468{,}000$ realized values of $\boldsymbol{L}$. The green lines indicate one estimated value of $4Ns$. 'Real $4Ns$ values' indicate the $4Ns$ values used in the simulations and 'Estimated $4Ns$ values' refers to the values estimated by our method. The median value of the estimates of $4Ns$ is shown with a solid line.

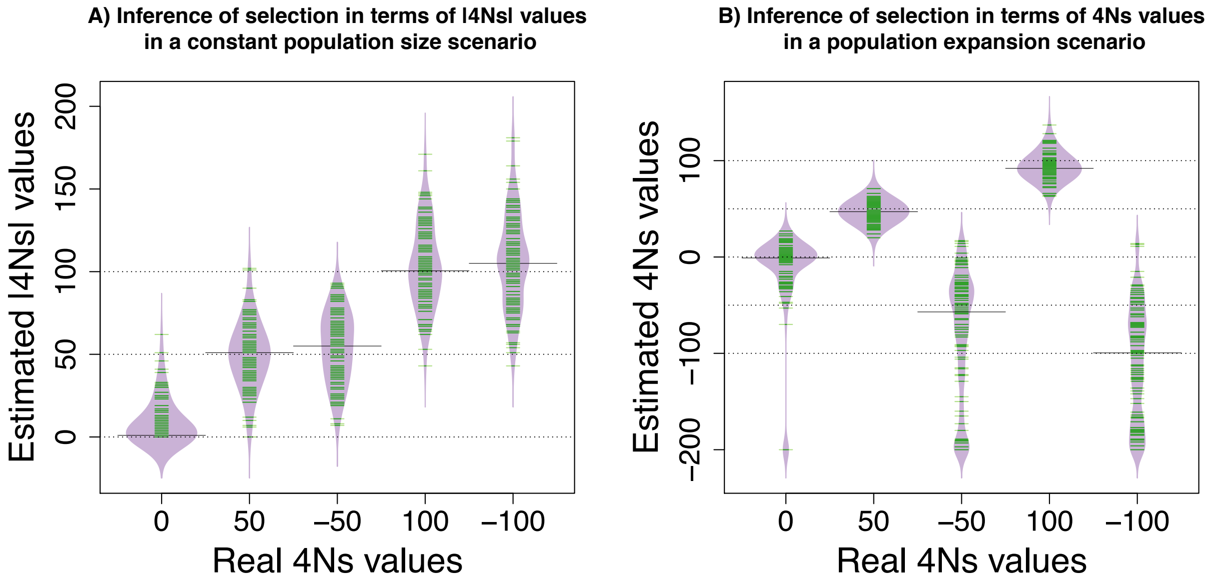

Figure S13

Estimation of the strength of natural selection for 1% frequency alleles in two demographic models after masking variants that only appear once in the sample of haplotypes with the derived allele.

Each simulation replicate contained $\mathbf{2} \times \boldsymbol{A} \times \binom{\boldsymbol{n}}{\mathbf{2}} = \mathbf{2} \times \mathbf{300} \times \binom{\mathbf{40}}{\mathbf{2}} = \mathbf{468{,}000}$ realized values of $\boldsymbol{L}$. The green lines indicate one estimated value of $\mathbf{4}\boldsymbol{Ns}$. ‘Real $\mathbf{4}\boldsymbol{Ns}$ values’ indicate the $\mathbf{4}\boldsymbol{Ns}$ values used in the simulations. ‘Estimated $\mathbf{4}\boldsymbol{Ns}$ values’ or ‘Estimated $|\mathbf{4}\boldsymbol{Ns}|$ values’ refers to the values estimated by our method. The median value of the estimates of $\mathbf{4}\boldsymbol{Ns}$ is shown with a solid line.

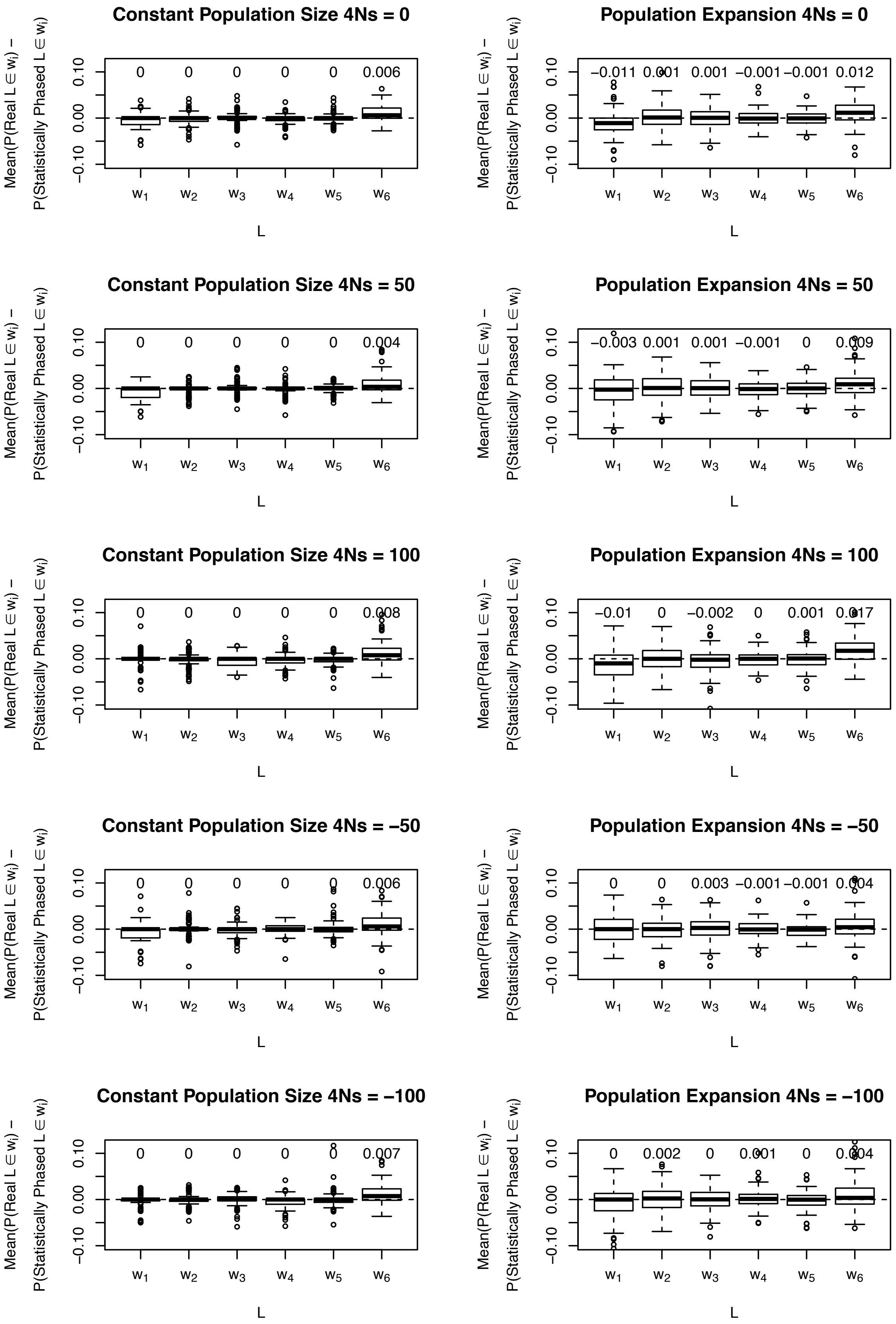

Constant Population Size 4Ns = 0
0 0 0 0 0 0.006
Population Expansion 4Ns = 0
−0.011 0.001 0.001 −0.001 −0.001 0.012
Constant Population Size 4Ns = 50
0 0 0 0 0 0.004
Population Expansion 4Ns = 50
−0.003 0.001 0.001 −0.001 0 0.009
Constant Population Size 4Ns = 100
0 0 0 0 0 0.008
Population Expansion 4Ns = 100
−0.01 0 −0.002 0 0.001 0.017
Constant Population Size 4Ns = −50
0 0 0 0 0 0.006
Population Expansion 4Ns = −50
0 0 0.003 −0.001 −0.001 0.004
Constant Population Size 4Ns = −100
0 0 0 0 0 0.007
Population Expansion 4Ns = −100
0 0.002 0 0.001 0 0.004
Mean(P(Real L ∈ w_i) − P(Statistically Phased L ∈ w_i)
−0.10 0.00 0.10
w1 w2 w3 w4 w5 w6
L

Figure S14

Impact of haplotype phasing errors on the estimates of $\boldsymbol{L}$.

We estimated the impact of haplotype phasing errors in $\boldsymbol{L}$ for two demographic models (constant population size model and a population expansion model) under 5 different values of $4Ns$ (0, 50, 100, -50, -100). We performed 100 simulations for each panel shown above, where there is a combination of one particular demographic model studied and one particular population-scaled value of selection $4Ns$. Each simulation is composed of $2\times\binom{40}{2}=1{,}560$ $\boldsymbol{L}$ values. In each simulation we estimated the proportion $P(L\in w_i)$ of $\boldsymbol{L}$ values that fall in each window w_i in data where the haplotype phase was known ($P(\text{Real } L\in w_i)$) and when the haplotypes were statistically phased ($P(\text{Statistically Phased } L\in w_i)$). The difference between $P(\text{Real } L\in w_i)$ and $P(\text{Statistically Phased } L\in w_i)$ is due to haplotype phasing errors. We report the mean value of $P(\text{Real } L\in w_i)$) - $P(\text{Statistically Phased } L\in w_i)$ for each of the 100 simulations, and plotted the 100 values obtained for each window w_i in each of the demographic models and $4Ns$ values explored.

In each simulation, we created a set of 2000 individuals that randomly contained either a derived or ancestral focal allele. There were 40 haplotypes containing the derived focal allele and 3960 haplotypes with the ancestral focal allele. Then, we performed the statistical phasing using the program ShapeIT2:

```
shapeit.v2.904.3.10.0-693.11.6.el7.x86_64/bin/shapeit --input-vcf VCF1.vcf -M
GeneticMap.txt -O TestShapeIt.phased
```

Where VCF1.vcf is a vcf file containing the unphased genotype information for 2,000 individuals (4,000 chromosomes); GeneticMap.txt is the genetic map of the region analyzed; and TestShapeIt.phased is the file with the statistically phased haplotypic information.

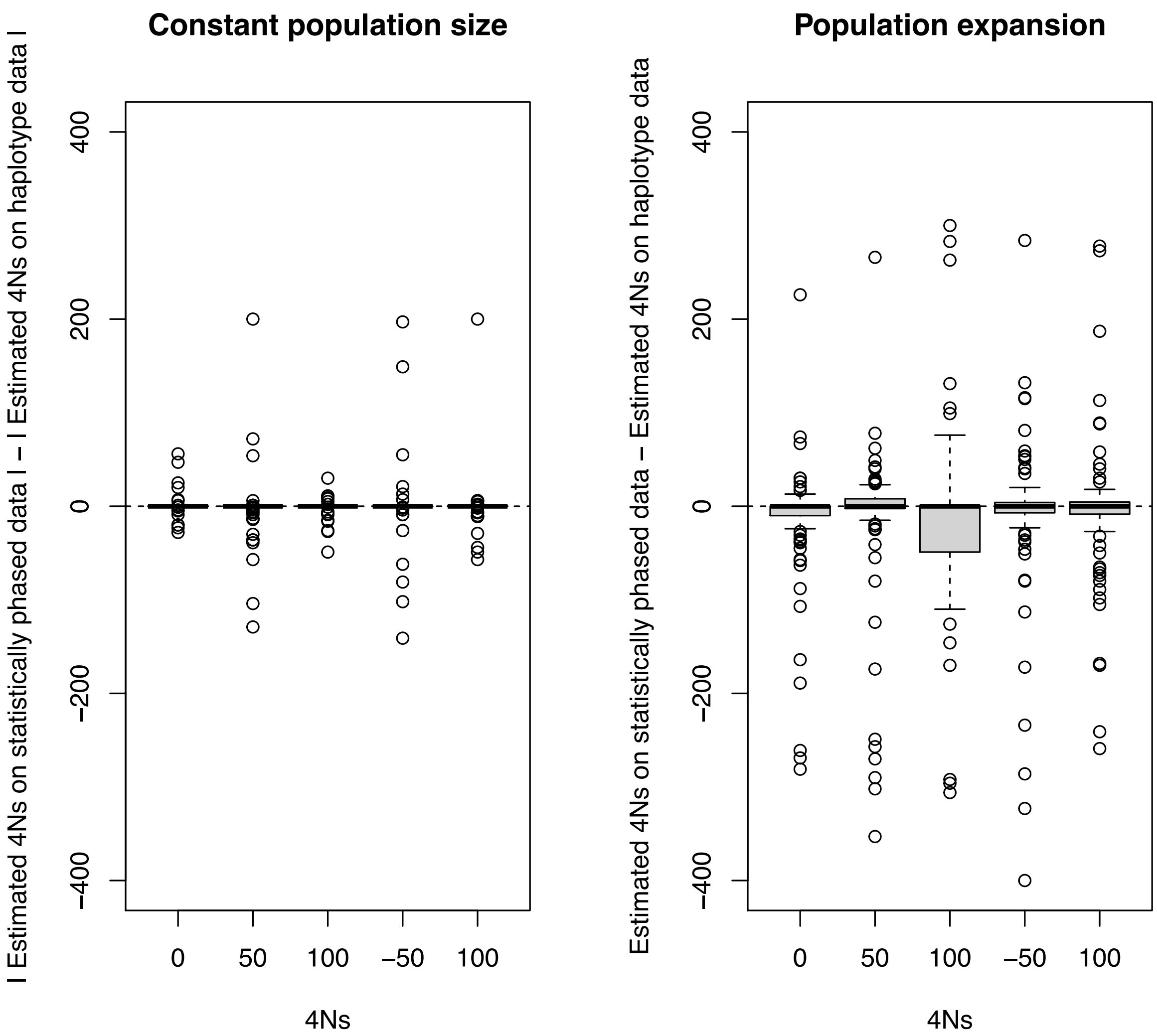

Figure S15

Biases in the estimated values of $4Ns$.

We estimated the value of $4Ns$ for each simulation two times. First, we estimated the value of $4Ns$ on the data when the haplotype phase is known. We refer to those estimates as the 'Estimated $4Ns$ on haplotype data'. Then, we estimated the value of $4Ns$ when we statistically phased the data (command shown in the past figure) and we refer to those $4Ns$ estimates as 'Estimated $4Ns$ on statistically phased data'. We computed the difference in absolute values between 'Estimated $4Ns$ on haplotype data' and 'Estimated $4Ns$ on statistically phased data' on 100 simulations done for simulations done under 5 different $4Ns$ values in the constant population size scenario. Each simulation is composed of $2 \times \binom{40}{2} = 1{,}560$ $\boldsymbol{L}$ values. We also computed the difference between 'Estimated $4Ns$ on haplotype data' and 'Estimated $4Ns$ on statistically phased data' on 100 simulations done for simulations done under 5 different $4Ns$ values in the population expansion size scenario.

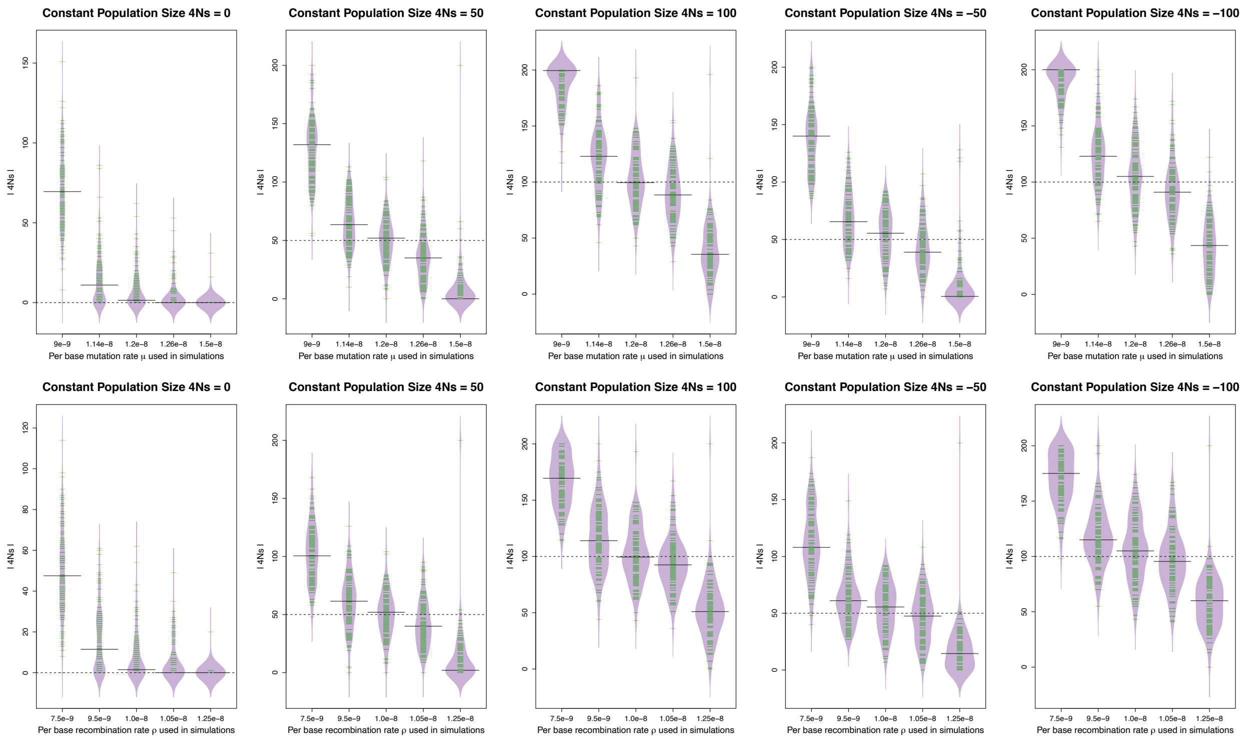

Figure S16

Biases in the estimation of $4Ns$ values due to a mutation rate or recombination rate misspecification in a constant population size model.

We estimated the likelihood function $\mathcal{L}(4Ns, f, D|L \in w_{m_j})$ using a mutation rate per base per generation equal to 1.2×10^{-8} and a recombination rate per base per generation equal to 1.0×10^{-8}. Then, we performed 100 simulation replicates for each combination of five different mutation rates and $4Ns$ values (upper panel); or a combination of five different recombination rates and $4Ns$ values (lower panel). Each simulation replicate contained $2 \times A \times \binom{n}{2} = 2 \times 300 \times \binom{40}{2} = 468{,}000$ realized values of L. We estimated the value of $4Ns$ in each simulation replicate using the likelihood function $\mathcal{L}(4Ns, f, D|L \in w_{m_j})$ generated with a mutation rate per base per generation equal to 1.2×10^{-8} and a recombination rate per base per generation equal to 1.0×10^{-8}. We could assess the effect of mutation rate and recombination rate misspecification because 4 out of the 5 mutation rates and recombination rates employed were different from the parameters used to generate the likelihood function $\mathcal{L}\left(4Ns, f, D\middle|L \in w_{m_j}\right)$.

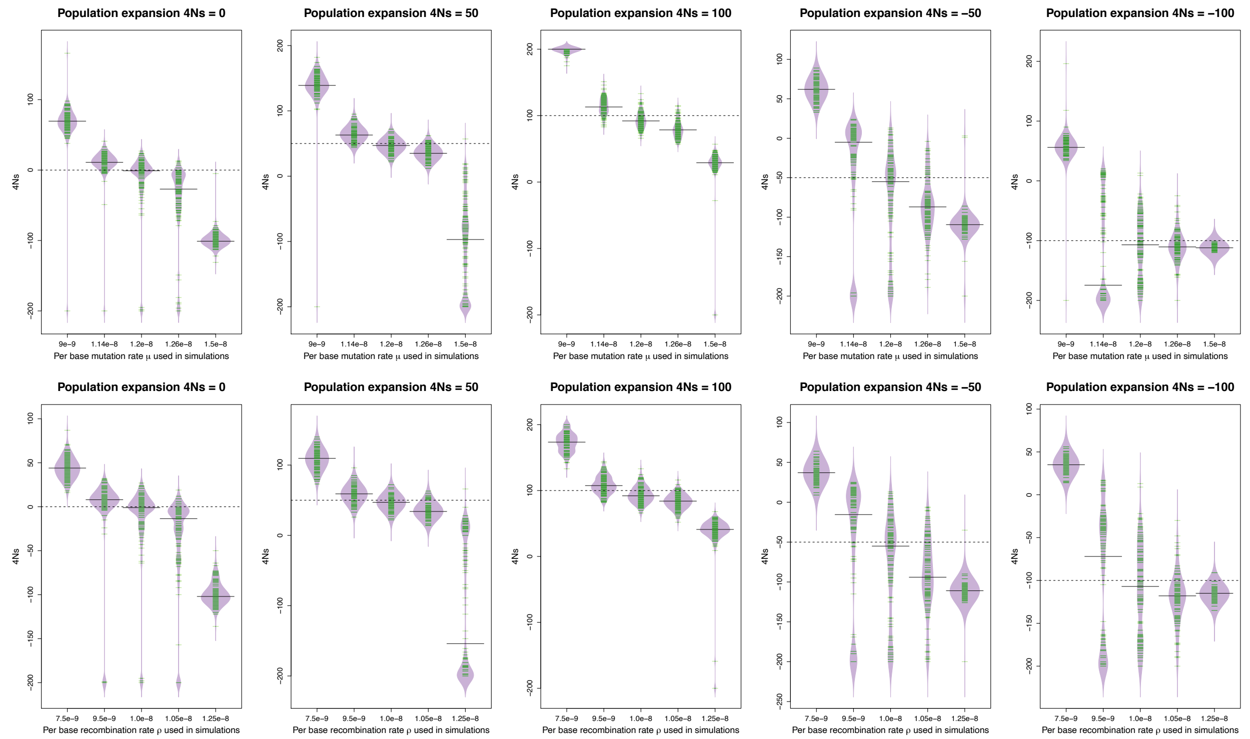

Figure S17

Biases in the estimation of $4Ns$ values due to a mutation rate or recombination rate misspecification in a population expansion model.

We estimated the likelihood function $\mathcal{L}(\mathbf{4}\boldsymbol{Ns}, \boldsymbol{f}, \boldsymbol{D} | \boldsymbol{L} \in \boldsymbol{w}_{\boldsymbol{m}_j})$ using a mutation rate per base per generation equal to 1.2×10^{-8} and a recombination rate per base per generation equal to 1.0×10^{-8}. Then, we performed 100 simulation replicates for each combination of five different mutation rates and $\mathbf{4}\boldsymbol{Ns}$ values (upper panel); or a combination of five different recombination rates and $\mathbf{4}\boldsymbol{Ns}$ values (lower panel). Each simulation replicate contained $\mathbf{2} \times \boldsymbol{A} \times \binom{\boldsymbol{n}}{\mathbf{2}} = \mathbf{2} \times \mathbf{300} \times \binom{\mathbf{40}}{\mathbf{2}} = \mathbf{468{,}000}$ realized values of $\boldsymbol{L}$. We estimated the value of $\mathbf{4}\boldsymbol{Ns}$ in each simulation replicate using the likelihood function $\mathcal{L}(\mathbf{4}\boldsymbol{Ns}, \boldsymbol{f}, \boldsymbol{D} | \boldsymbol{L} \in \boldsymbol{w}_{\boldsymbol{m}_j})$ generated with a mutation rate per base per generation equal to 1.2×10^{-8} and a recombination rate per base per generation equal to 1.0×10^{-8}. We could assess the effect of mutation rate and recombination rate misspecification because 4 out of the 5 mutation rates and recombination rates employed were different from the parameters used to generate the likelihood function $\mathcal{L}\left(\mathbf{4}\boldsymbol{Ns}, \boldsymbol{f}, \boldsymbol{D} \middle| \boldsymbol{L} \in \boldsymbol{w}_{\boldsymbol{m}_j}\right)$.

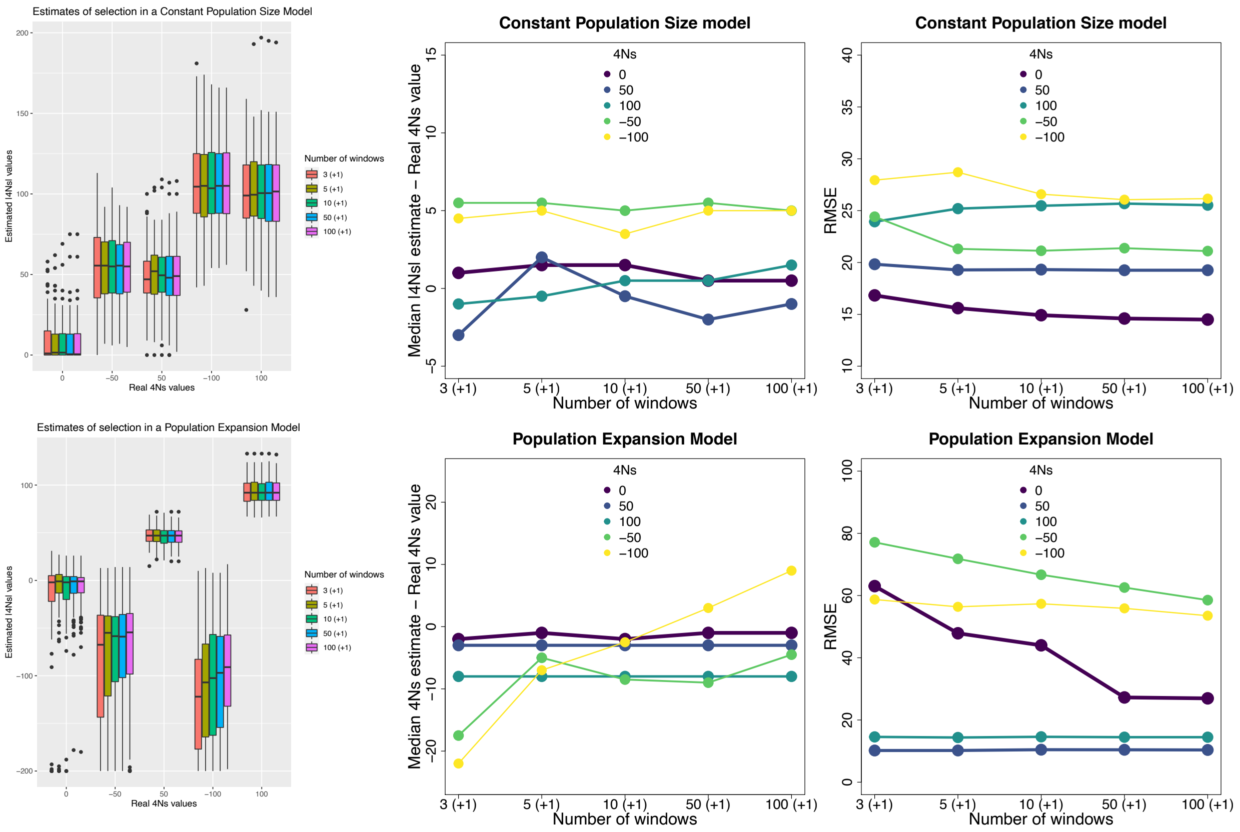

Figure S18

Estimation of selection using a different number of windows under our constant population size model and population expansion model. We divided the vicinity of the haplotype surrounding the derived allele into 3, 5, 10, 50, and 100 windows (plus an extra window denoting no differences in the adjacent 250 kb window)

The differences in the estimated $|4\text{Ns}|$ and 4Ns values in the constant population size and population expansion model are shown in the left plots across 100 simulation replicates including $2 \times A \times \binom{n}{2} = 2 \times 300 \times \binom{40}{2} = 468{,}000$ realized values of L per replicate. The difference in the median $|4\text{Ns}|$ and 4Ns value and the 4Ns value used in the simulations with the constant population size and population expansion model across 100 simulation replicates is shown in the middle plots. The Root Mean Square Error is shown in the right side plots for both models. This is calculated using the $|4\text{Ns}|$ and 4Ns estimates and real values used in the constant population size and population expansion demographic scenarios, respectively.

When we used 11, 51 and 101 windows, we had less than 5% of the L values falling in each window bin. Despite this property we had nearly unbiased estimates of selection when using 11, 51 and 101 windows. We saw that having more than 5% of the L values falling in each window bin gave nearly unbiased results when using 6 window bins for the two models presented here. We did not need to have more than 5% of the L values falling in each window when using 11, 51 and 101 windows in this analysis.

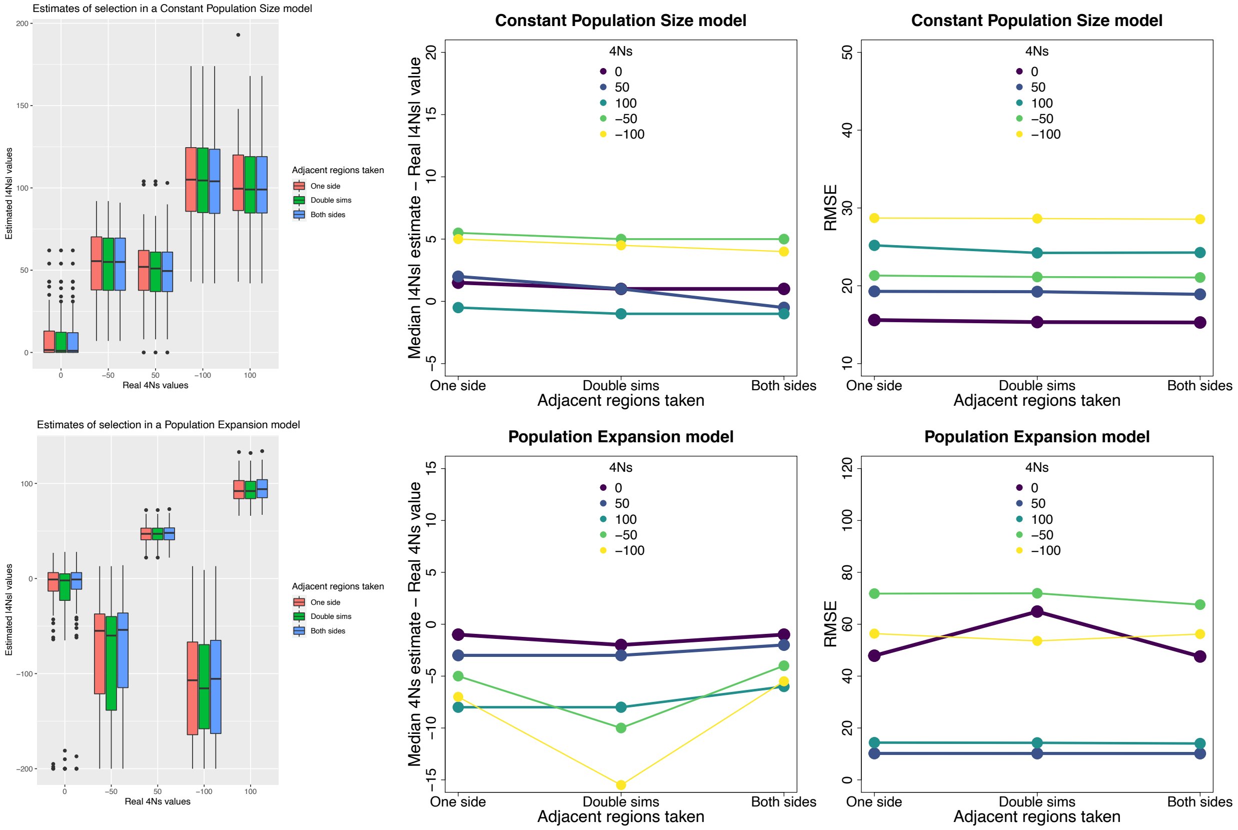

Figure S19

Estimates of 4Ns obtained using equation (1) after calculating $\mathbf{P}\left(\mathbf{L} \in \mathbf{w_j} \middle| \mathbf{D}, \mathbf{H_k}\right)$ with three alternative Monte-Carlo strategies

Strategy 'One Side'.- We simulated the focal allele at the left end of a 250 kb region and calculated $\ell = \binom{n}{2}$ values of $\mathbf{L}$ for each set of n simulated haplotypes. We simulate 100 sets of haplotypes for each H_k to obtain $\ell = 100 \times \binom{n}{2}$ values of $\mathbf{L}$ for each H_k.

Strategy 'Double Sims'.- Compared to 'One side' here we simulate 200 sets of haplotypes for each H_k to obtain $\ell = 200 \times \binom{n}{2}$ values of $\mathbf{L}$ for each H_k.

Strategy 'Both sides'.- We simulated the focal allele at the center of a 500 kb region and calculated $\ell = 2\,X \binom{n}{2}$ values of $\mathbf{L}$ for each set of n simulated haplotypes by going towards the left side and right side of the focal allele. We simulate 100 sets of haplotypes for each H_k to obtain $\ell = 200 \times \binom{n}{2}$ values of $\mathbf{L}$ for each H_k.

With the three strategies we plot:

Left side plots.- The differences in the estimated $|4Ns|$ and $4Ns$ values in the constant population size and population expansion model are shown in the left plots across 100 simulation replicates including $2 \times A \times \binom{n}{2} = 2 \times 300 \times \binom{40}{2} = 468{,}000$ realized values of $\mathbf{L}$ per replicate.

Middle plots.- The difference in the median $|4Ns|$ and $4Ns$ value with the real values used in the simulations under the constant population size and population expansion model, respectively, across 100 simulation replicates is shown in the middle plots.

Right side plots.- The Root Mean Square Error is shown in the right side plots for both models. This is calculated using the $|4Ns|$ and $4Ns$ estimates and real values used in the constant population size and population expansion demographic scenarios.

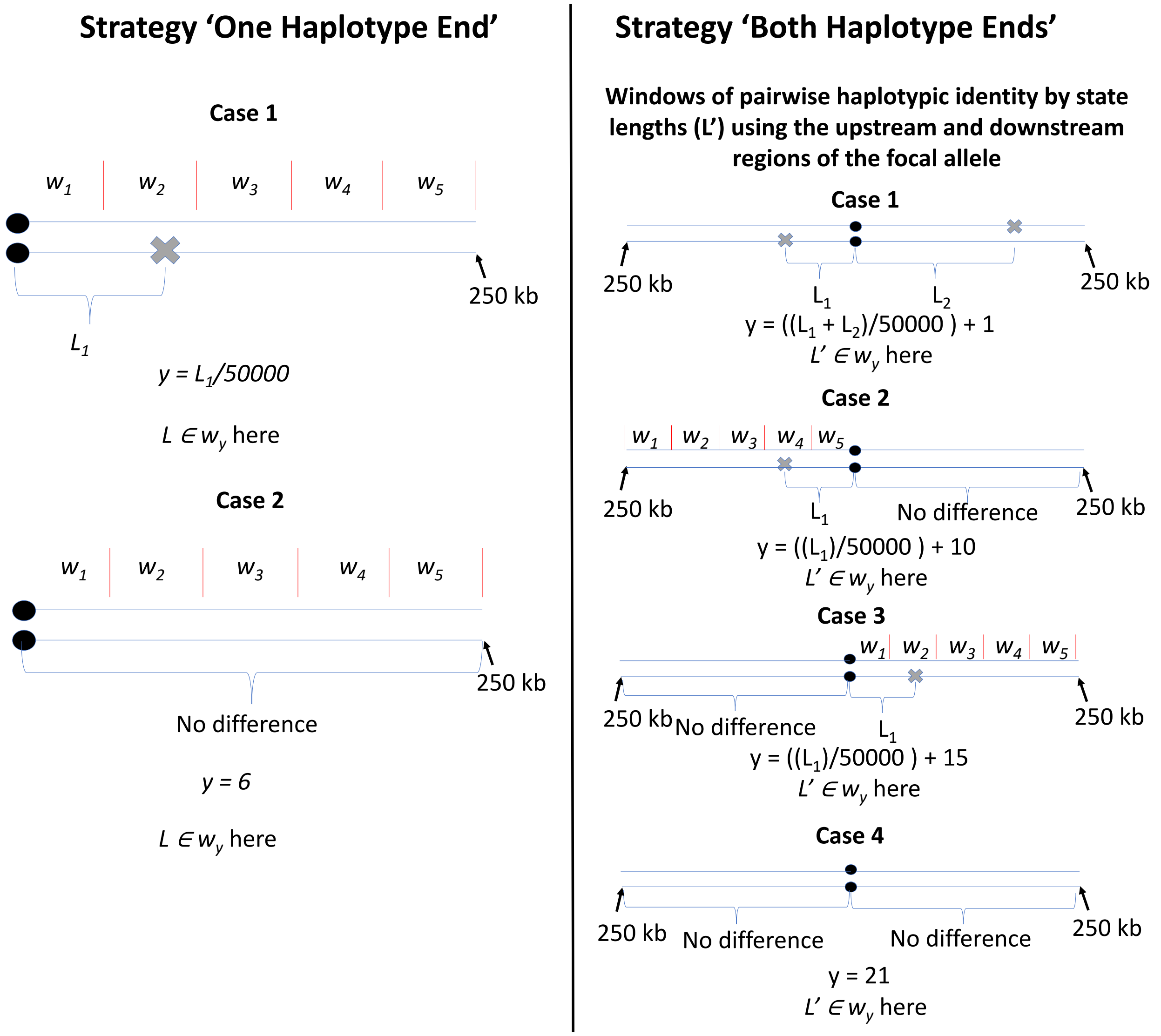
Strategy 'One Haplotype End'
Case 1
w_1 w_2 w_3 w_4 w_5
250 kb
L_1
$y = L_1/50000$
$L \in w_y$ here
Case 2
w_1 w_2 w_3 w_4 w_5
250 kb
No difference
$y = 6$
$L \in w_y$ here
Strategy 'Both Haplotype Ends'
Windows of pairwise haplotypic identity by state lengths (L') using the upstream and downstream regions of the focal allele
Case 1
250 kb
L_1
L_2
250 kb
$y = ((L_1 + L_2)/50000) + 1$
$L' \in w_y$ here
Case 2
w_1 w_2 w_3 w_4 w_5
250 kb
L_1
No difference
250 kb
$y = ((L_1)/50000) + 10$
$L' \in w_y$ here
Case 3
w_1 w_2 w_3 w_4 w_5
250 kb
No difference
L_1
250 kb
$y = ((L_1)/50000) + 15$
$L' \in w_y$ here
Case 4
250 kb
No difference
No difference
250 kb
$y = 21$
$L' \in w_y$ here

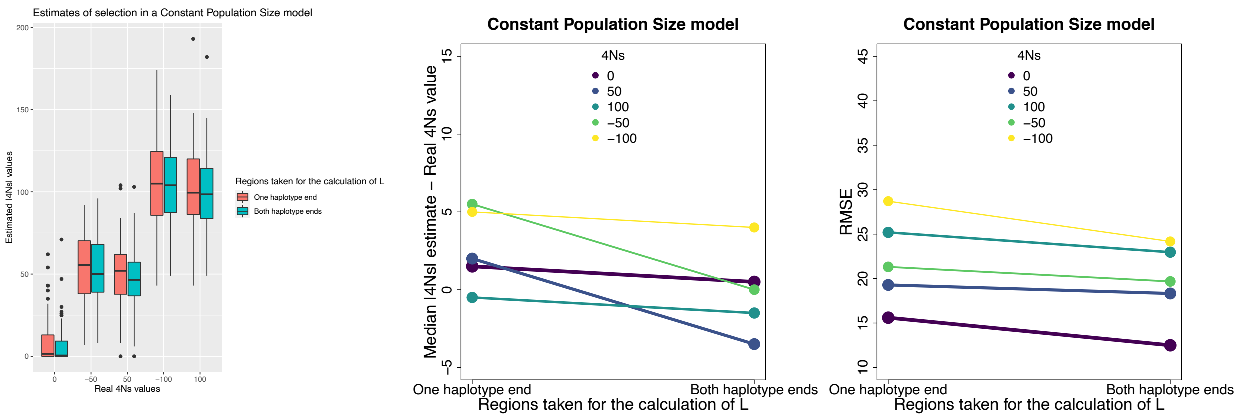
Estimates of selection in a Constant Population Size model
Estimated l4NsI values
Real 4Ns values
Regions taken for the calculation of L
One haplotype end
Both haplotype ends
Constant Population Size model
4Ns
0
50
100
−50
−100
Median l4NsI estimate − Real 4Ns value
One haplotype end
Both haplotype ends
Regions taken for the calculation of L
Constant Population Size model
RMSE
One haplotype end
Both haplotype ends
Regions taken for the calculation of L

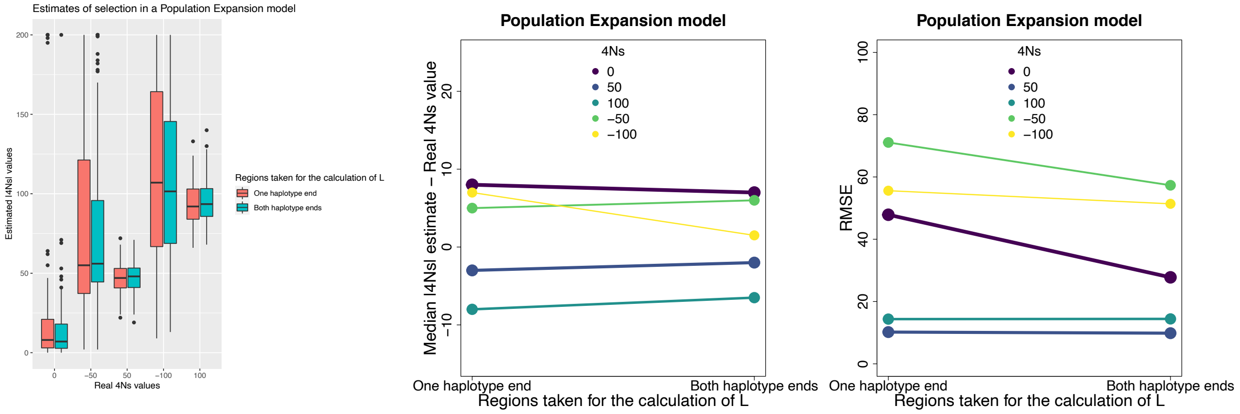

Figure S20.- Estimates of 4Ns obtained using equation (1) after calculating $\mathbf{P}\left(\mathbf{L} \in \mathbf{w_j} \middle| \mathbf{D}, \mathbf{H_k}\right)$ with 100 sets of $\mathbf{n}$ haplotypes for each realization of $\mathbf{H_k}$ using two strategies

Strategy ‘One Haplotype End’.- We simulated the focal allele at the left end of a 250 kb region and calculated $\ell = \binom{n}{2}$ values of $\mathbf{L}$ for each set of n simulated haplotypes. We simulate 100 sets of haplotypes for each $\mathrm{H_k}$ to obtain $\ell = 100 \times \binom{n}{2}$ values of $\mathbf{L}$ for each $\mathrm{H_k}$.

Strategy ‘Both haplotype ends’.- We simulated the focal allele at the center of a 500 kb region and calculated $\ell = \binom{n}{2}$ values of $\mathbf{L}$ for each set of n simulated haplotypes by using information from both the left and right region adjacent to the focal allele. The procedure to compute $\mathrm{L} \in \mathrm{w_j}$ using both strategies is depicted in the upper plot. We simulate 100 sets of haplotypes for each $\mathrm{H_k}$ to obtain $\ell = 100 \times \binom{n}{2}$ values of $\mathbf{L}$ for each $\mathrm{H_k}$. For both strategies we plot:

Left side plots.- The differences in the estimated $|4\mathrm{Ns}|$ and $4\mathrm{Ns}$ values in the constant population size and population expansion model are shown in the left plots across 100 simulation replicates including $2 \times \mathrm{A} \times \binom{n}{2} = 2 \times 300 \times \binom{40}{2} = 468{,}000$ realized values of L per replicate.

Middle plots.- The difference in the median $|4\mathrm{Ns}|$ and $4\mathrm{Ns}$ value with the real values used in the simulations under the constant population size and population expansion model, respectively, across 100 simulation replicates is shown in the middle plots.

Right side plots.- The Root Mean Square Error is shown in the right side plots for both models. This is calculated using the $|4\mathrm{Ns}|$ and $4\mathrm{Ns}$ estimates and real values used in the constant population size and population expansion demographic scenarios.

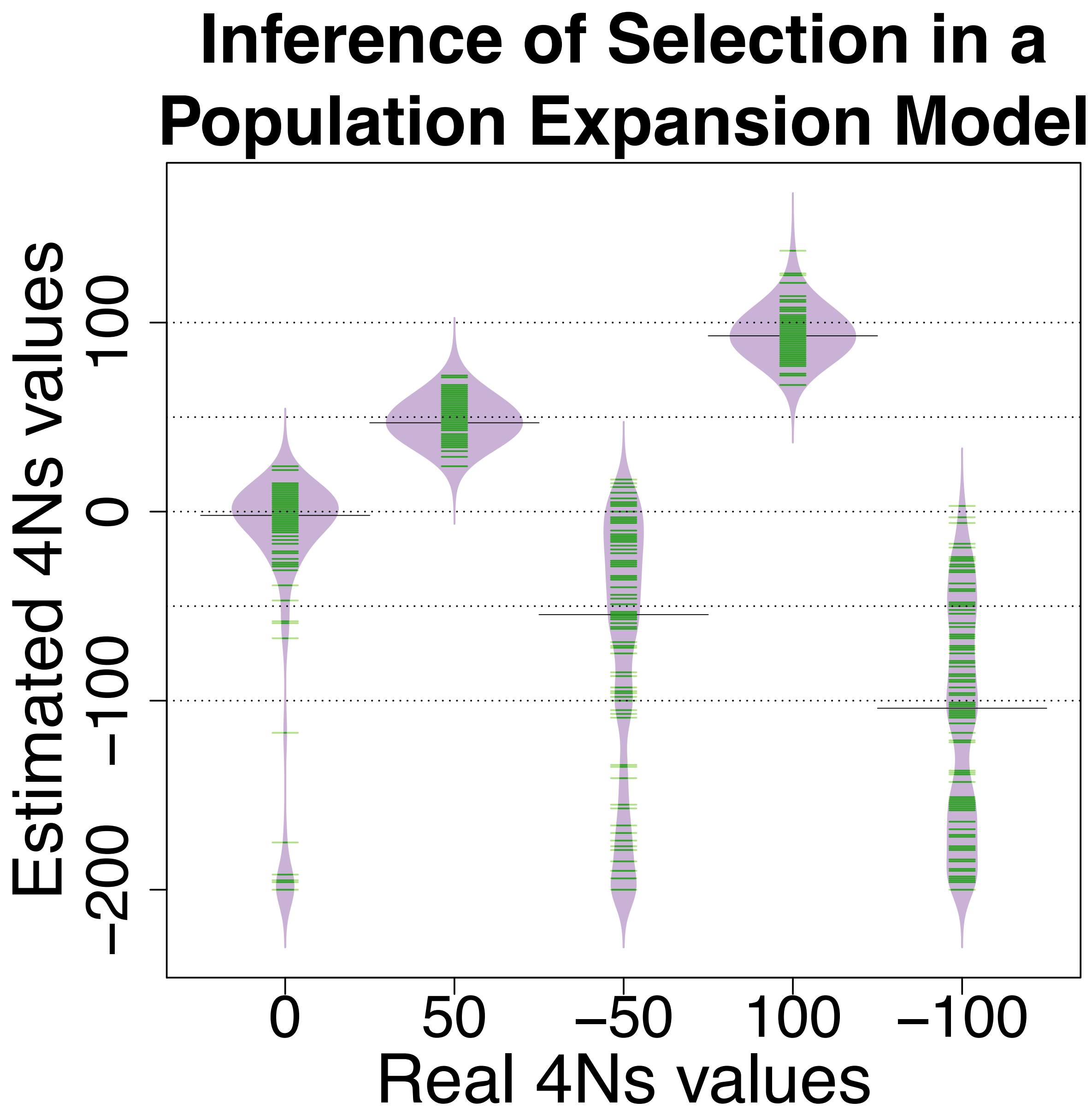

Figure S21

Estimation of the strength of natural selection in a population expansion model for 1% frequency alleles. Each simulation replicate contains A=150 variants with a recombination rate in the surrounding region equal to $r = 0$ and A=150 variants with a recombination rate in the surrounding region $r = 1 \text{ X } 10^{-8}$

Each simulation replicate contained $2 \times A \times \binom{n}{2} = 2 \times 300 \times \binom{40}{2} = 468,000$ realized values of L. The green lines indicate one estimated value of $4Ns$. 'Real $4Ns$ values' indicate the $4Ns$ values used in the simulations and 'Estimated $4Ns$ values' refers to the values estimated by our method. The median value of the estimates of $4Ns$ is shown with a solid line.

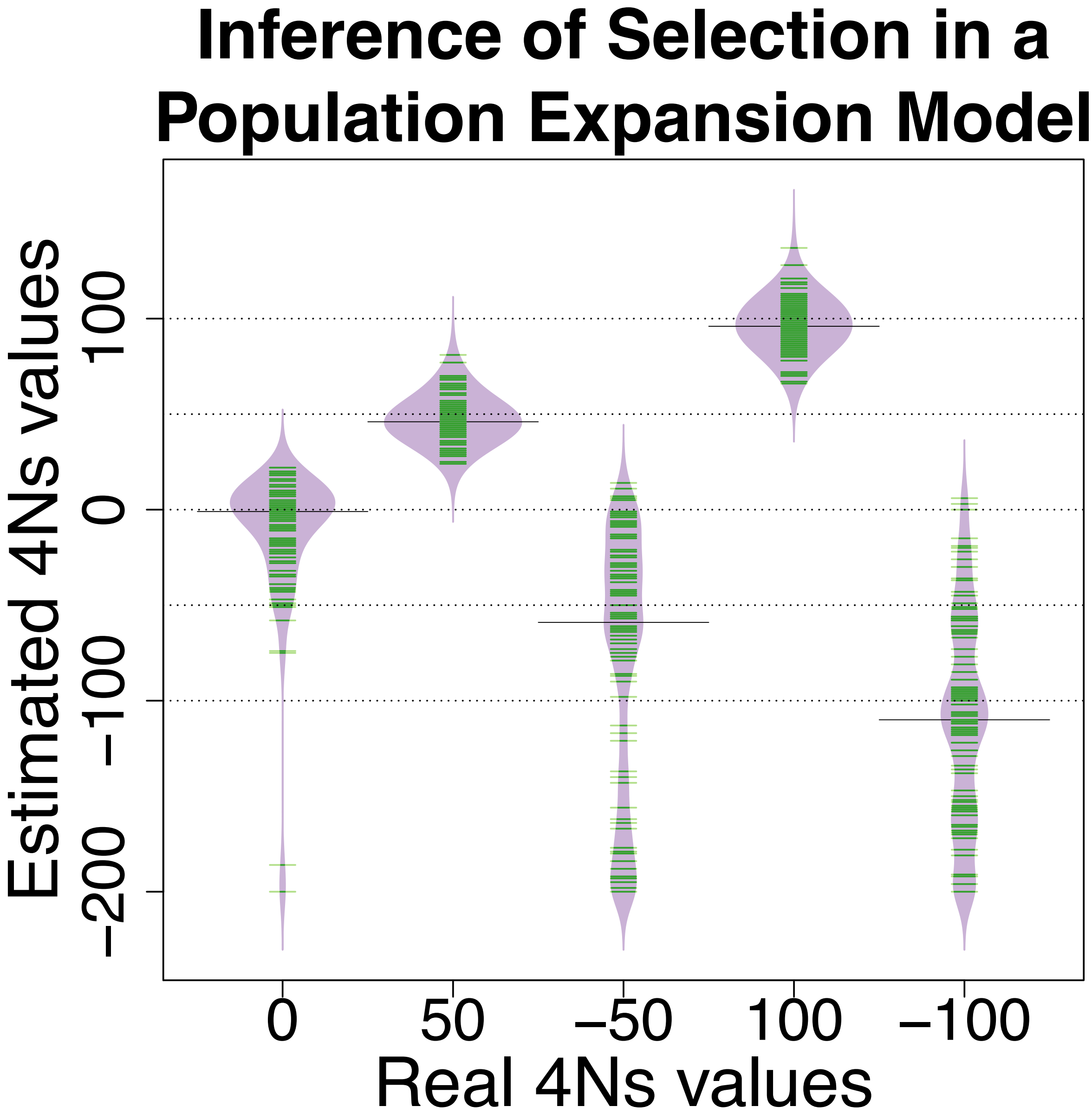

Figure S22

Estimation of the strength of natural selection in a population expansion model for 1% frequency alleles with a different recombination rate for each of the 300 variants.

Each simulation replicate contained $\mathbf{2} \times \boldsymbol{A} \times \binom{n}{2} = \mathbf{2} \times \mathbf{300} \times \binom{\mathbf{40}}{\mathbf{2}} = \mathbf{468,000}$ realized values of $\boldsymbol{L}$. The green lines indicate one estimated value of $\mathbf{4}\boldsymbol{Ns}$. ‘Real $\mathbf{4}\boldsymbol{Ns}$ values’ indicate the $\mathbf{4}\boldsymbol{Ns}$ values used in the simulations and ‘Estimated $\mathbf{4}\boldsymbol{Ns}$ values’ refers to the values estimated by our method. The median value of the estimates of $\mathbf{4}\boldsymbol{Ns}$ is shown with a solid line.

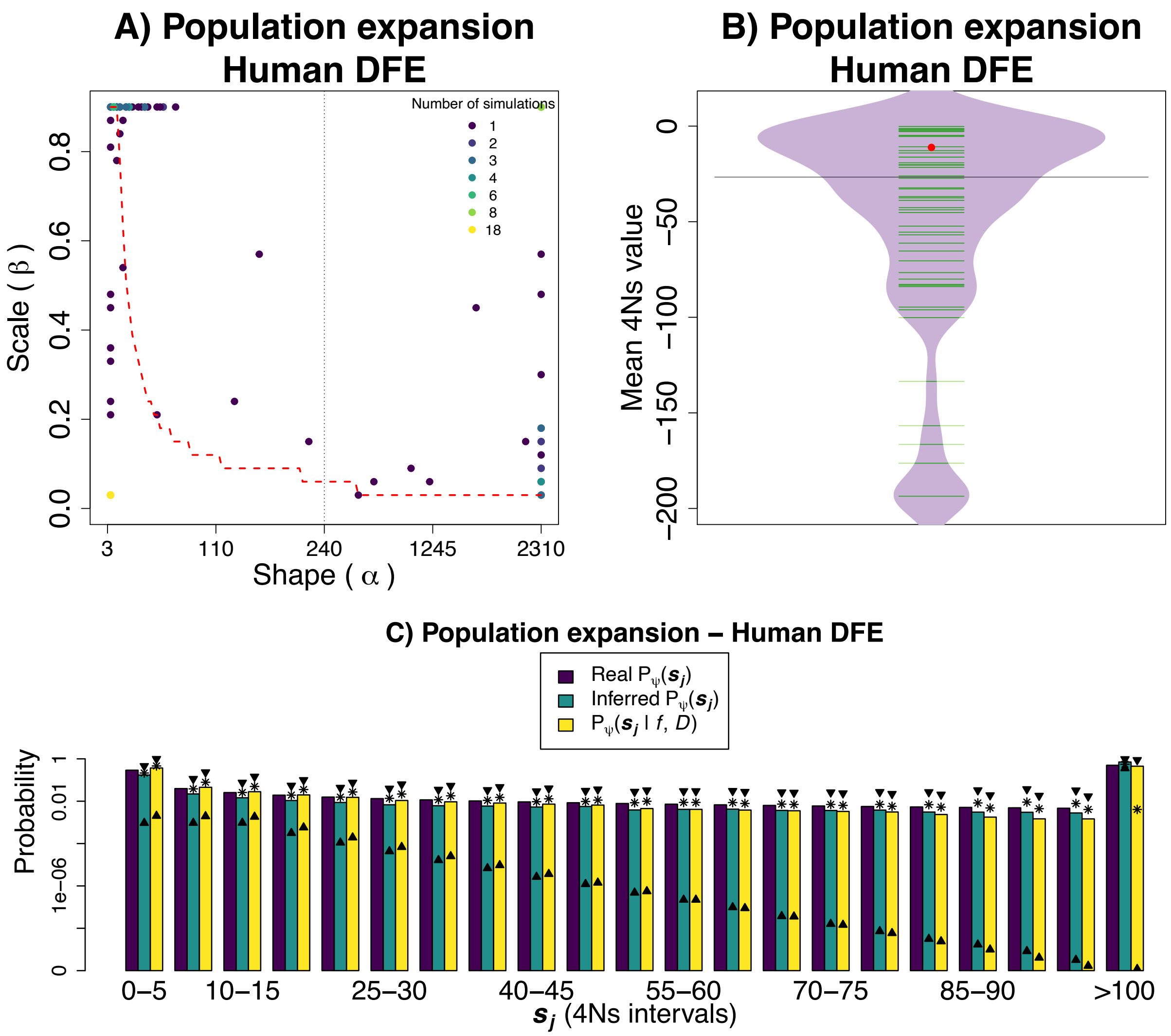

Figure S23

Estimation of the strength of natural selection in a population expansion model for 1% frequency alleles with a different recombination rate for each of the 300 variants when the distribution of fitness effects uses the human distribution of fitness effects.

A) We tested if our method was capable of estimating the parameters of the $\boldsymbol{DFE_f}$ of variants at a frequency of 1%. The shape ($\boldsymbol{\alpha}$) and scale ($\boldsymbol{\beta}$) parameters define the compound $\boldsymbol{DFE_f}$ distribution. Each simulation replicate contained $\mathbf{2} \times \boldsymbol{A} \times \binom{\boldsymbol{n}}{\mathbf{2}} = \mathbf{2} \times \mathbf{300} \times \binom{\mathbf{40}}{\mathbf{2}} = \mathbf{468,000}$ realized values of $\boldsymbol{L}$. The number of simulation replicates estimated to have a particular combination of $\boldsymbol{\alpha}$ and $\boldsymbol{\beta}$ parameters is shown with a different color in each plot. The dotted red line represents a combination of shape and scale parameters from the partially collapsed gamma distribution that gives a similar mean $\boldsymbol{4Ns}$ value to the mean $\boldsymbol{4Ns}$ value of the underlying $\boldsymbol{DFE_f}$. The grid of scale parameters explored goes from (**0.03, 0.06, …, 0.9**) and the grid of shape parameters explored goes from (**3, 6, … , 210**) and then there is a change in the grid of shape parameters explored, specified by the dotted line, and the grid takes values from (**240, 270, …, 2310**) .

B) The beanplots show the distribution of the estimated mean $\boldsymbol{4Ns}$ values based on the $\boldsymbol{DFE_f}$ estimated on the 100 simulation replicates. The red dots show the actual mean $\boldsymbol{4Ns}$ value in 50,000 1% frequency variants simulated using each particular $\boldsymbol{DFE}$ and demographic model $\boldsymbol{D}$. The green lines indicate estimated values of $\boldsymbol{4Ns}$ across simulation replicates based on the $\boldsymbol{DFE_f}$ estimates. The median value of the estimates of $\boldsymbol{4Ns}$ is shown with a solid line.

C) 'Real $P_\psi(\boldsymbol{s_j})$' is the proportion of variants in a certain $\boldsymbol{s_j}$ interval based on the parameters ψ that define the distribution of fitness effects of new variants DFE.

'Real $P_\psi(\boldsymbol{s_j})$' is the proportion of variants in a certain $\boldsymbol{s_j}$ interval based on the parameters ψ that define the distribution of fitness effects of new variants DFE. The bars on '$P_\psi(\boldsymbol{s_j}|f,D)$' and 'Inferred $P_\psi(\boldsymbol{s_j})$' represent the median value of those respective estimated probabilities across 100 simulation replicates, where each simulation replicate employs $2 \times A \times \binom{n}{2} = 2 \times 300 \times \binom{40}{2} = 468{,}000$ L values. The two triangles shown in each $\boldsymbol{s_j}$ interval denote the 5% and 95% percentile of the 'Inferred $P_\psi(\boldsymbol{s_j})$' or '$P_\psi(\boldsymbol{s_j}|f,D)$' probabilities estimated across 100 simulation replicates. The mean values of '$P_\psi(s_j|f,D)$' and 'Inferred $P_\psi(s_j)$' from 100 simulations replicates are shown with an asterisk.

We estimated the values of 'Inferred $\boldsymbol{P_\psi(s_j)}$' using equation (6). The following terms were calculated to use equation (6) (Also see '*Connecting the distribution of fitness effects of variants at a particular frequency ($\boldsymbol{DFE_f}$) with the distribution of fitness effects of new mutations ($\boldsymbol{DFE}$)*'):

$P_\psi(\boldsymbol{s_j}|f,D)$.- Comes from the inferences of $DFE_f(\alpha,\beta)$ taken from Panel A).

$P_\psi(f|D)$.- Calculated from $2{,}500$ simulations done using *PReFerSim* under the population expansion scenario and the Human DFE. The number of new mutations appearing each generation follows a Poisson distribution with a mean equal to $2N_iul = 1{,}000$ in the ancestral epoch in each simulation, where N_i is the population size in generation i.

$P_\psi(f|\boldsymbol{s_j},D)$.- Calculated from $2{,}500$ simulations done using *PReFerSim*. The number of new mutations appearing each generation follows a Poisson distribution with a mean equal to $2N_iul = 1{,}000$ in the ancestral epoch in each simulation, where N_i is the population size in generation i. The mean of the Poisson distribution changes between epochs as defined by the N_i values. We performed the simulations to estimate $P_\psi(f|\boldsymbol{s_j},D)$ with the Mouse DFE. The DFE used to perform the simulations to calculate $P_\psi(f|\boldsymbol{s_j},D)$ is arbitrary as long as the DFE encompasses all the intervals $\boldsymbol{\sigma} = \{\boldsymbol{s_1},\boldsymbol{s_2},\boldsymbol{s_3},\ldots,\boldsymbol{s_b}\}$ analyzed. Here we decided to use a DFE to calculate $P_\psi(f|\boldsymbol{s_j},D)$ that is different from the DFE we are using to simulate and estimate the values of 'Real $P_\psi(\boldsymbol{s_j})$' in each plot.

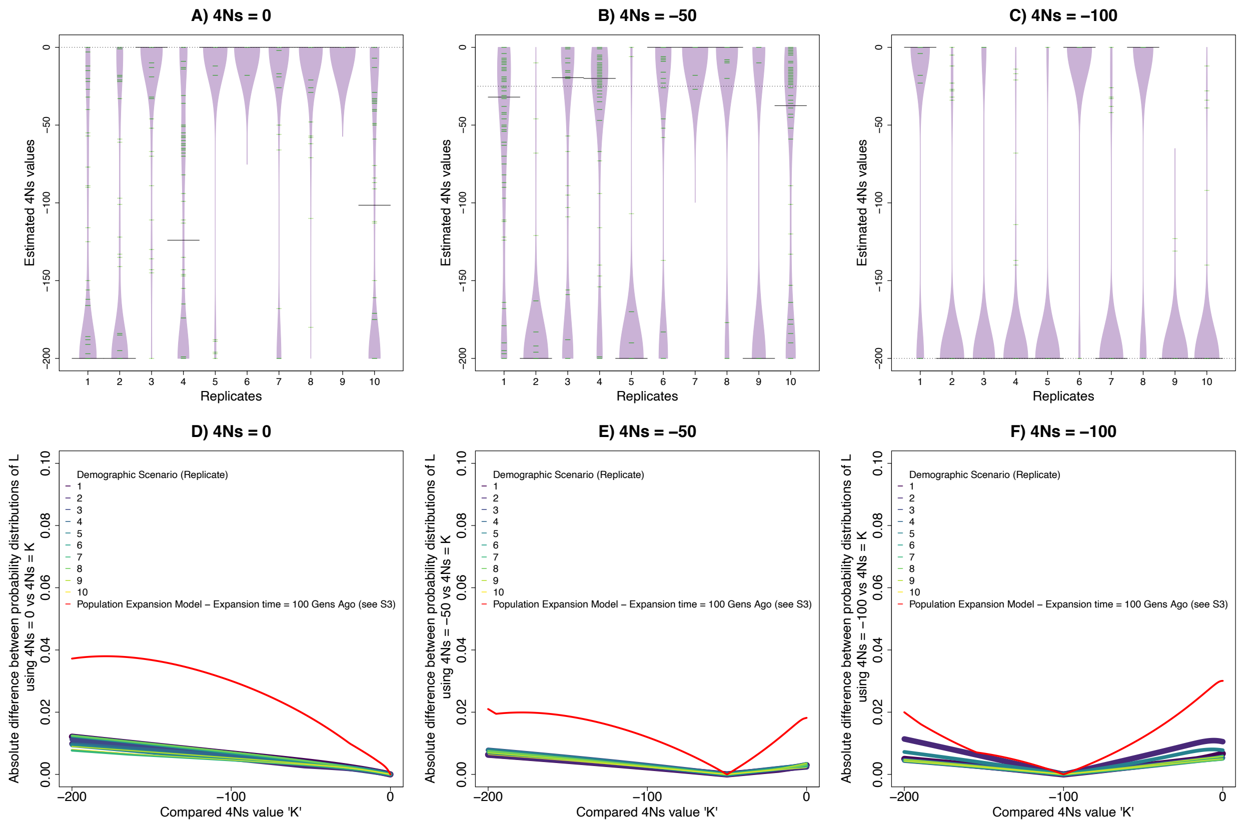

Figure S24

Estimation of the strength of natural selection using SLiM simulations.

A-C) We estimated the demographic parameters to perform the inferences of $\mathbf{4Ns}$ using 10 different sets of synonymous mutations for each value of $\mathbf{4Ns}$ (see Supplementary Methods). We performed 100 inferences of $\mathbf{4Ns}$ in each of the 10 demographic models inferred. The x-axis in each plot shows 10 replicates. Each replicate shows one of the 10 demographic models inferred. The $\mathbf{4Ns}$ value used for all the simulated nonsynonymous mutations is shown at the top of each plot.

We analyzed 100 sets of simulations with $\boldsymbol{\ell} = \mathbf{600} \times \binom{\mathbf{40}}{\mathbf{2}} = \mathbf{468,000}$ realized values of $\boldsymbol{L}$ used for each simulation replicate. Each set of simulations contained $\mathbf{600}$ independent 1% frequency variants, where each variant had 40 haplotypes with the derived allele. 'Estimated $\mathbf{4Ns}$ values' refers to the values estimated by our method. The large dashed line shows the median $\mathbf{4Ns}$ value estimated across the 1,000 estimated $\mathbf{4Ns}$ values. The median value of the estimates of $\mathbf{4Ns}$ is shown with a solid line in each simulation replicate. The green lines are estimated values of $\mathbf{4Ns}$, where there are 100 estimated values of $\mathbf{4Ns}$ in each replicate.

The grid of explored $4Ns$ values goes from -200 to 0.

The 25th and 75th percentile of the estimated $4Ns$ values was equal to [0,-157.25], [0, -200] and [0, -200] for the simulations done with a $4Ns$ value equal to 0, -50 and -100 for the nonsynonymous mutations.

D-F) Y axis label.- Absolute difference between probability distributions of L using $4Ns = J$, vs $4Ns = K$. J takes values equal to 0 (*D*), -50 (*E*) or -100 (*F*). This is calculated as $Dif = \sum_{i=1}^{6} |P(L \in w_i | f, D, 4Ns = J) - P(L \in w_i | f, D, 4Ns = K)|$.

We plotted the value of Dif for the 10 demographic models inferred for each value of selection and for a population expansion model where the population expansion time took place 100 generations ago (100G model). We see that the value of Dif is broadly lower in the 10 demographic models inferred for each value of selection than in the population expansion model where the population expansion time took place 100 generations ago. As seen in the caption of Figure S3A, the value of Dif is sufficiently small in the 100G model across a broad range of values, particularly from 4Ns = 0 to 4Ns = -200, such that a small deviation on the distribution of L values can push the 4Ns estimates to one edge of the grid of explored values. The even smaller differences seen in Figure S24D, S24E and S24F signal that we should also

expect to see estimates going to one or both edges of the distribution of 4Ns values. We see that this is the case based on the 4Ns estimates presented on Figure S24A, S24B and S24C. The small values of *Dif* are responsible for the broad bimodal distribution of estimated 4Ns values, clustering close to 0 or -200, observed when taking together the results from Figure S24A, S24B and S24C.

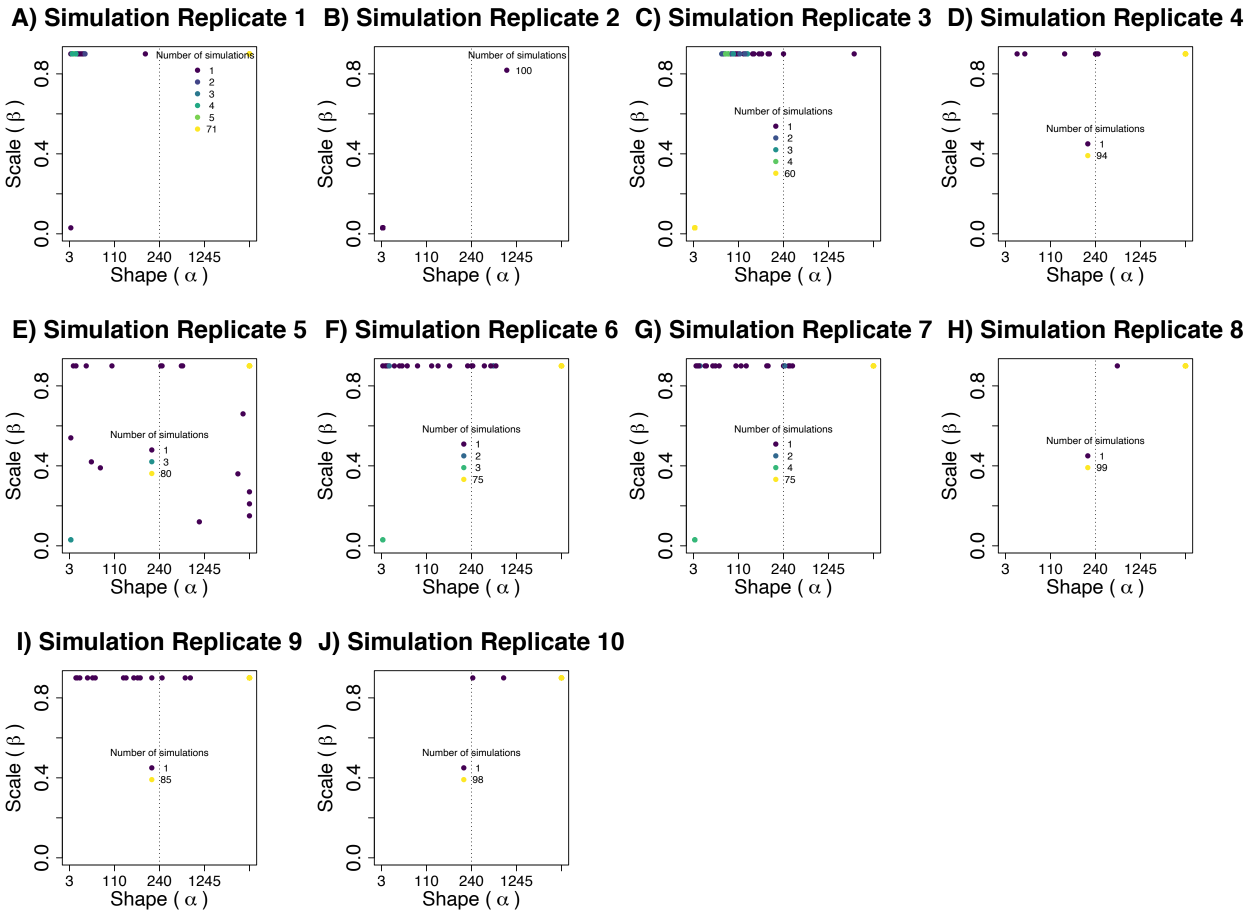

Figure S25

Estimation of the strength of natural selection using SLiM simulations.

We estimated the demographic parameters to perform the inferences of $\boldsymbol{4Ns}$ using 10 different sets of synonymous mutations for each value of $\boldsymbol{4Ns}$ (see Supplementary Methods). We performed 100 inferences of $\boldsymbol{4Ns}$ in each of the 10 demographic models inferred. Each simulation replicate shows inferences done using one of the 10 demographic models inferred.

We analyzed 100 sets of simulations with $\ell = 600 \times \binom{40}{2} = 468{,}000$ realized values of L for each simulation replicate. Each set of simulations contained 600 independent 1% frequency variants, where each variant contained 40 haplotypes with the derived allele.

The number of sets of simulations estimated to have a particular combination of $\boldsymbol{\alpha}$ and $\boldsymbol{\beta}$ parameters is shown with a different color in each plot. The grid of scale parameters explored goes from ($0.03,\ 0.06,\ \ldots,\ 0.6$) and the grid of shape parameters explored goes from ($3,\ 6,\ \ldots\ ,\ 210$) and then there is a change in the grid of shape parameters explored, specified by the dotted line, and the grid then takes values from ($240,\ 270, \ldots, 2100$) .

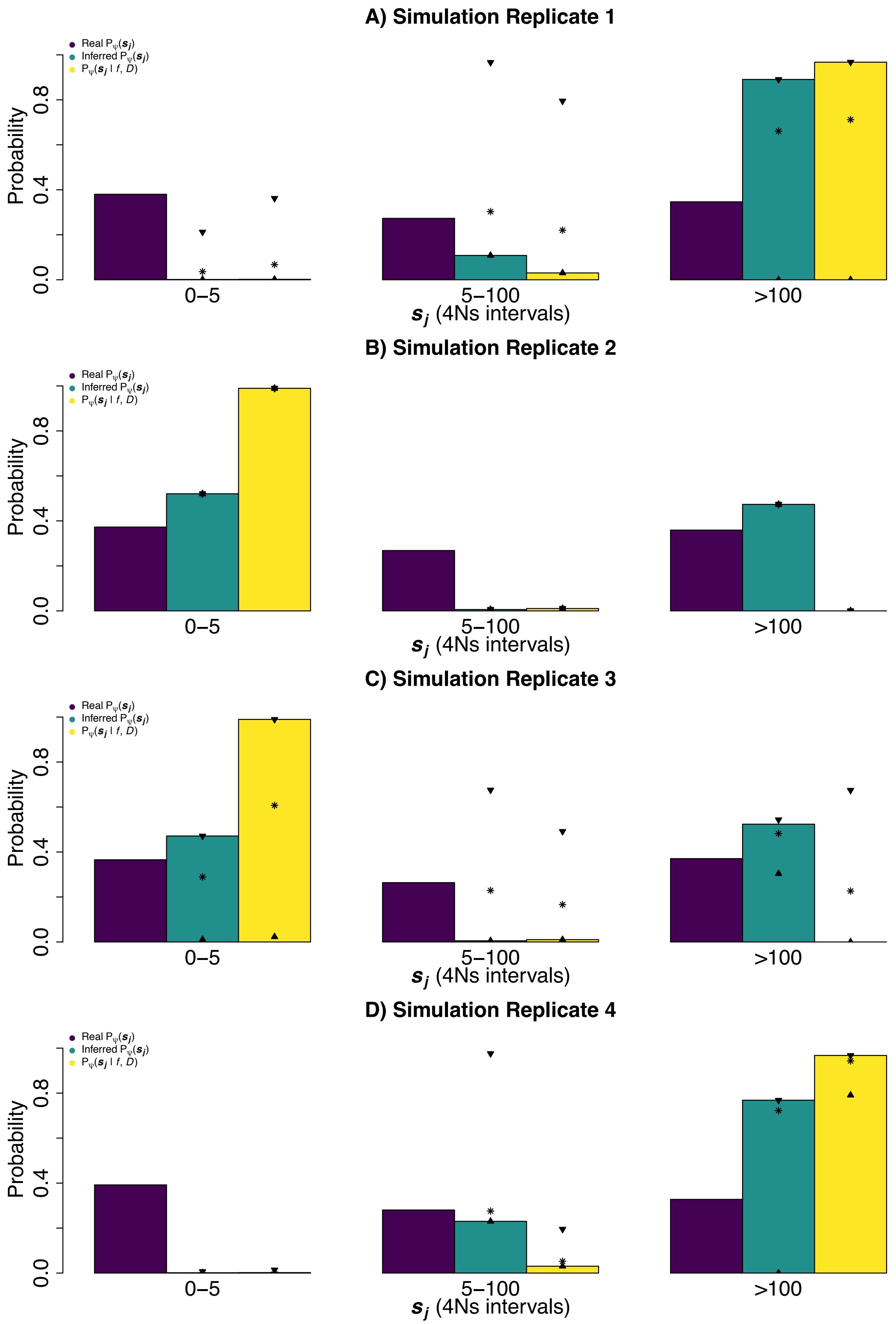
A) Simulation Replicate 1
Real Pψ(sj)
Inferred Pψ(sj)
Pψ(sj | f, D)
Probability
0.0
0.4
0.8
0–5
5–100
>100
sj (4Ns intervals)
B) Simulation Replicate 2
Real Pψ(sj)
Inferred Pψ(sj)
Pψ(sj | f, D)
Probability
0.0
0.4
0.8
0–5
5–100
>100
sj (4Ns intervals)
C) Simulation Replicate 3
Real Pψ(sj)
Inferred Pψ(sj)
Pψ(sj | f, D)
Probability
0.0
0.4
0.8
0–5
5–100
>100
sj (4Ns intervals)
D) Simulation Replicate 4
Real Pψ(sj)
Inferred Pψ(sj)
Pψ(sj | f, D)
Probability
0.0
0.4
0.8
0–5
5–100
>100
sj (4Ns intervals)

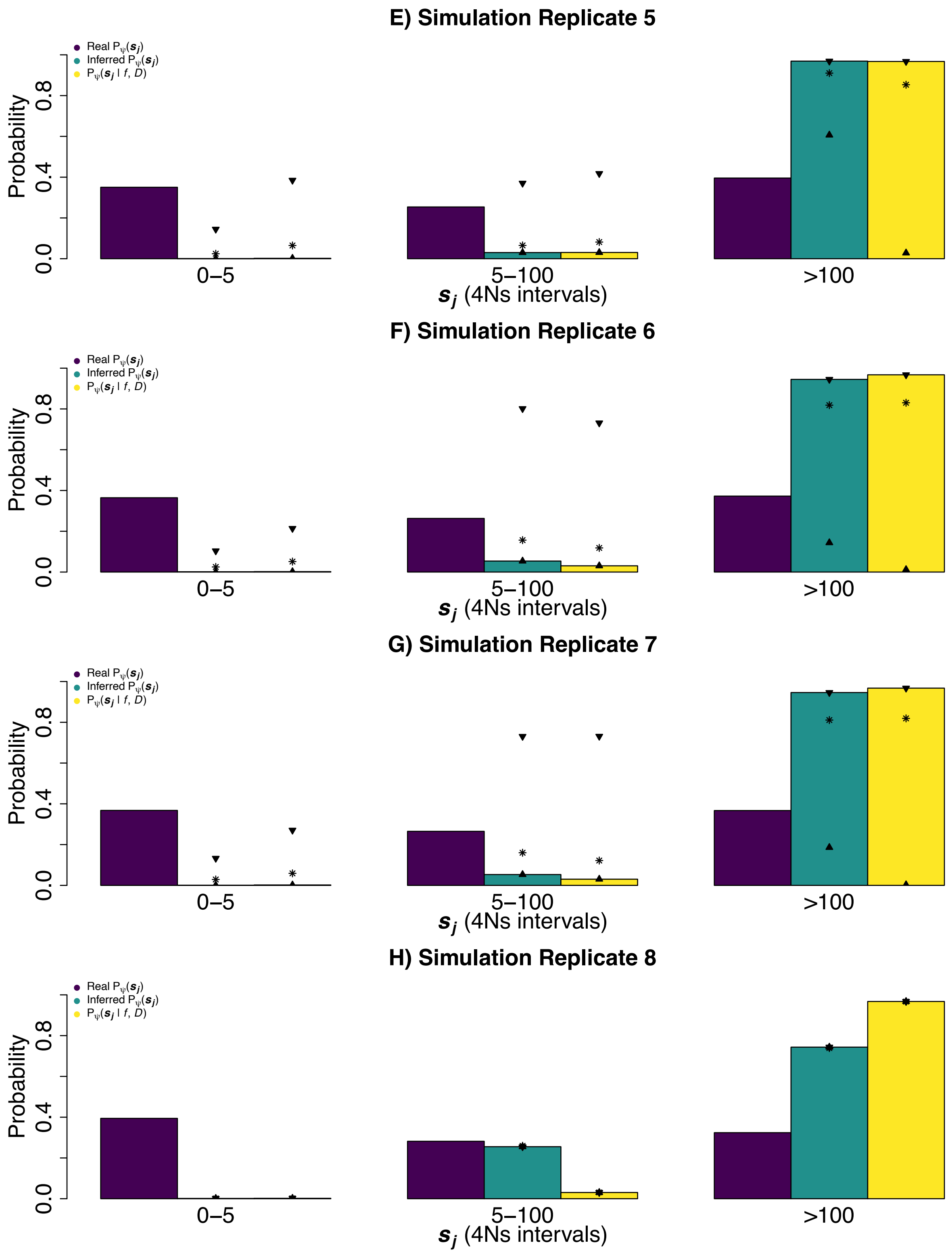
E) Simulation Replicate 5
F) Simulation Replicate 6
G) Simulation Replicate 7
H) Simulation Replicate 8
Real Pψ(sj)
Inferred Pψ(sj)
Pψ(sj | f, D)
Probability
0.0
0.4
0.8
0–5
5–100
>100
sj (4Ns intervals)

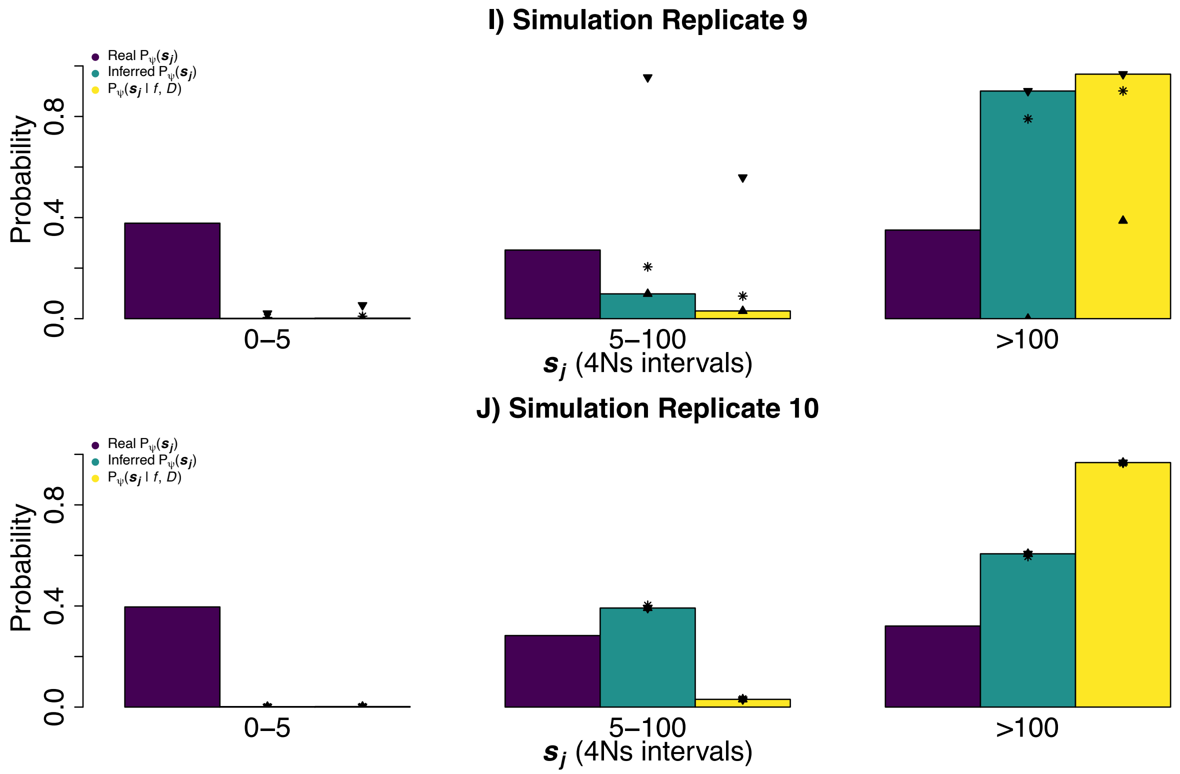
I) Simulation Replicate 9
Real $P_\psi(s_j)$
Inferred $P_\psi(s_j)$
$P_\psi(s_j \mid f, D)$
Probability
0.0
0.4
0.8
0–5
5–100
>100
s_j (4Ns intervals)
J) Simulation Replicate 10
Real $P_\psi(s_j)$
Inferred $P_\psi(s_j)$
$P_\psi(s_j \mid f, D)$
Probability
0.0
0.4
0.8
0–5
5–100
>100
s_j (4Ns intervals)

Figure S26

Estimation of the strength of natural selection using SLiM simulations.

We estimated the demographic parameters to perform the inferences of $\boldsymbol{4Ns}$ using 10 different sets of synonymous mutations for each value of $\boldsymbol{4Ns}$ (see Supplementary Methods). We performed 100 inferences of $\boldsymbol{4Ns}$ in each of the 10 demographic models inferred. Each simulation replicate shows inferences done using one of the 10 demographic models inferred.

We analyzed 100 sets of simulations with $\ell = 600 \times \binom{40}{2} = 468{,}000$ realized values of L for each simulation replicate. Each set of simulations contained 600 independent 1% frequency variants, where each variant contained 40 haplotypes with the derived allele.

The value of N in each simulation replicate depends on the demographic scenario D estimated in each simulation replicate. It is equal to the effective population size estimated in the first epoch in each simulation replicate.

'Real $P_\psi(s_j)$' refers to the actual probability of having a $4Ns$ value in a particular interval s_j given the distribution of fitness effects of new mutations DFE.

'Inferred $P_\psi(s_j)$' refers to the inferred probability of having a $4Ns$ value in a particular interval s_j given the distribution of fitness effects of new mutations DFE.

'$P_\psi(s_j|f, D)$' is the inferred probability of having a $4Ns$ value in an interval s_j given the distribution of fitness effects DFE with parameters ψ and the demographic scenario D in $f = 1\%$ frequency variants.

The probabilities 'Inferred $P_\psi(s_j)$' and '$P_\psi(s_j|f, D)$' in the s_j interval = $[5,\ 50)$ were obtained by summing up the probabilities of 'Inferred $P_\psi(s_j)$' and '$P_\psi(s_j|f, D)$' over the intervals $[5,\ 10)$, $[10,\ 15)$, $[15,\ 20)$, $[20,\ 25)$, $[25,\ 30)$, $[30,\ 35)$, $[35,\ 40)$, $[40,\ 45)$ and $[45,\ 50)$. The selection coefficient s refers exclusively to the action of deleterious variants in this plot. The two triangles shown in each s_j interval denote the 5% and 95% percentile of the probabilities 'Inferred $P_\psi(s_j)$' and '$P_\psi(s_j|f, D)$' over 100 sets of simulations in each simulation replicate. The asterisk signs from 'Inferred $P_\psi(s_j)$' and '$P_\psi(s_j|f, D)$' are the mean values calculated from 100 sets of simulations.

We estimated the values of 'Inferred $\boldsymbol{P_\psi(s_j)}$' using equation (6). The following terms were calculated to use equation (6) (Also see '*Connecting the distribution of fitness effects of variants at a particular frequency ($\boldsymbol{DFE_f}$) with the distribution of fitness effects of new mutations ($\boldsymbol{DFE}$)*'):

$P_\psi(s_j|f, D)$.- Comes from the inferences of $DFE_f(\alpha, \beta)$.

$P_\psi(f|D)$.- We estimated this probability using SLiM simulations. In our simulations, we divided the genome into 101 non-overlapping regions of 20 Mb that covered the whole genome. We simulated each region of the genome 160 times. Then, we estimated $P_\psi(f|D)$ as the number of variants that had a frequency $f = 1\%$ over the expected number of mutations in the estimated demographic scenario in each simulation replicate using Equation S15. The mutation rate per base u was set equal to $4.19\,X\,10^{-7}$, which is equal to the ratio of nonsynonymous over synonymous mutations in exons, which we set as $2.31/3.31$ following (Huber *et al.* 2017), times the scaling factor used in the simulations (5), times $1.2\,X\,10^{-8}$. The value of l was equal to the number of bases in the exons $(29{,}277{,}495)$ times across the 101 non-overlapping regions multiplied by the number of times each region was simulated (160), making l equal to $4{,}684{,}399{,}200$. The value of N_g depends on the population size estimated in each generation in the demographic model estimated in each simulation replicate.

$P_\psi(f|s_j, D)$.- Calculated from 400 simulations done using *PReFerSim* under the demographic scenario inferred in each simulation replicate. We used the Mouse DFE in this simulations after scaling the selection coefficient by 5. The number of new mutations appearing each generation follows a Poisson distribution with a mean equal to $2N_i ul = 10{,}000$ in the ancestral epoch in each simulation, where N_i is the population size in generation i. The mean of the Poisson distribution changes between epochs as defined by the N_i values.

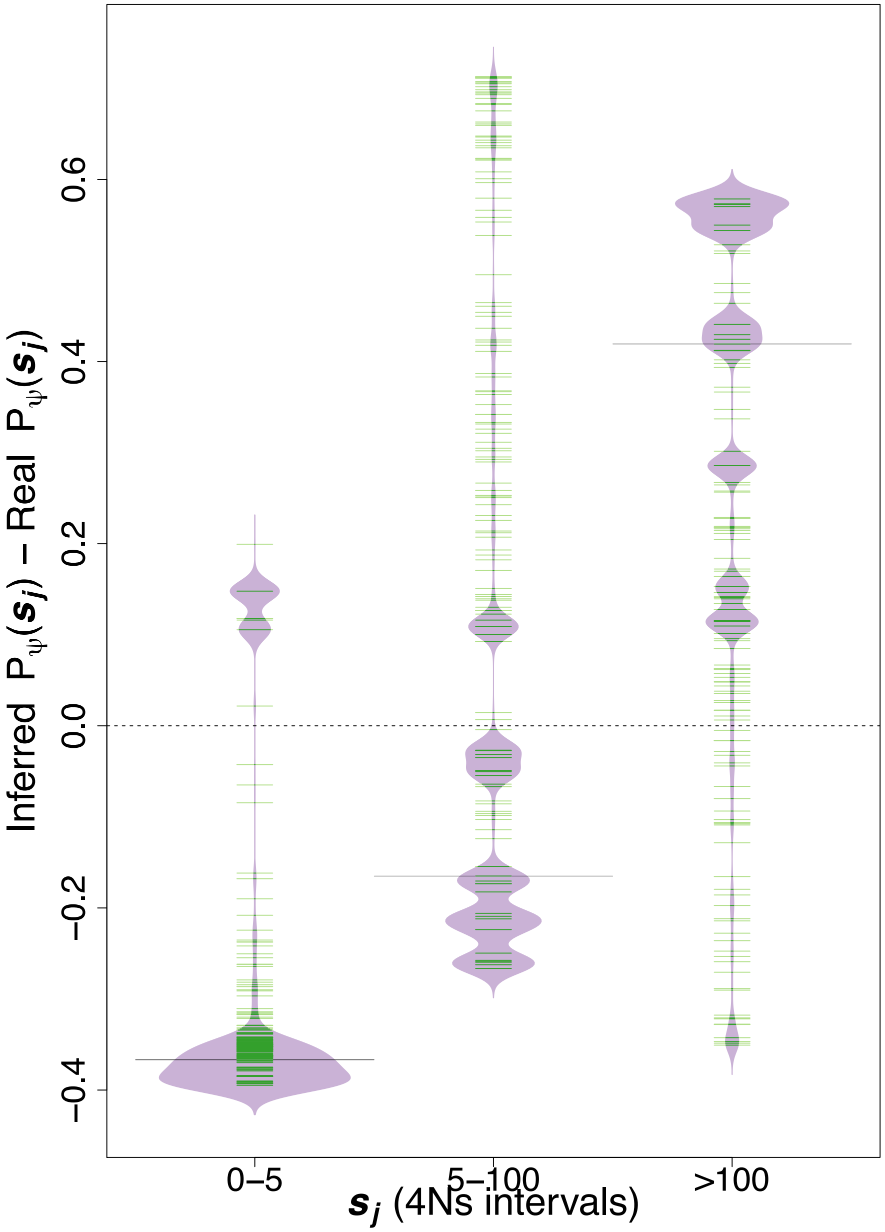

Figure S27

Difference between the 'Inferred $P_\psi(s_j)$' and the 'Real $P_\psi(s_j)$' probabilities over the 1,000 sets of simulations performed (100 sets of simulations per simulation replicate) in 3 different s_j bins.

The 5th and 95th percentile of the difference between the 'Inferred $P_\psi(s_j)$' and the 'Real $P_\psi(s_j)$' probabilities were equal to [-0.394, 0.148], [-0.262, 0.461], [-0.197, 0.5787].

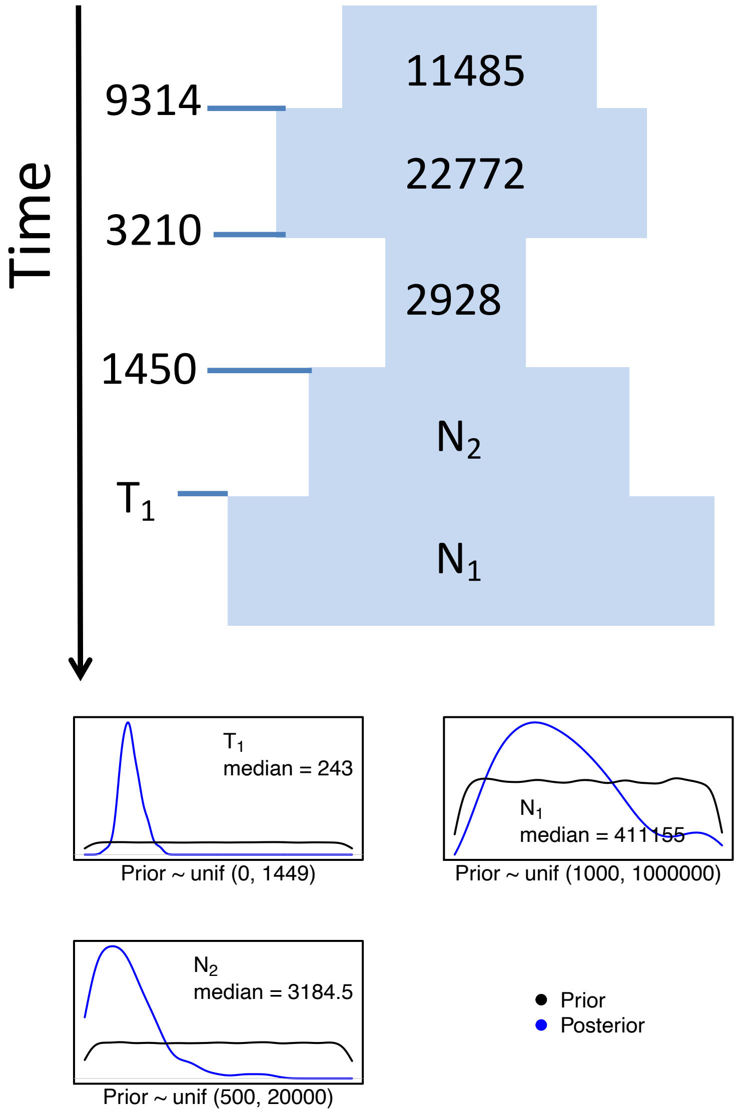

Figure S28

Inference of demographic models that fit the L distribution at 1% frequency synonymous variants in the *UK10K* dataset.

The plots below show the prior and posterior distribution for the demographic parameters in the demographic model.

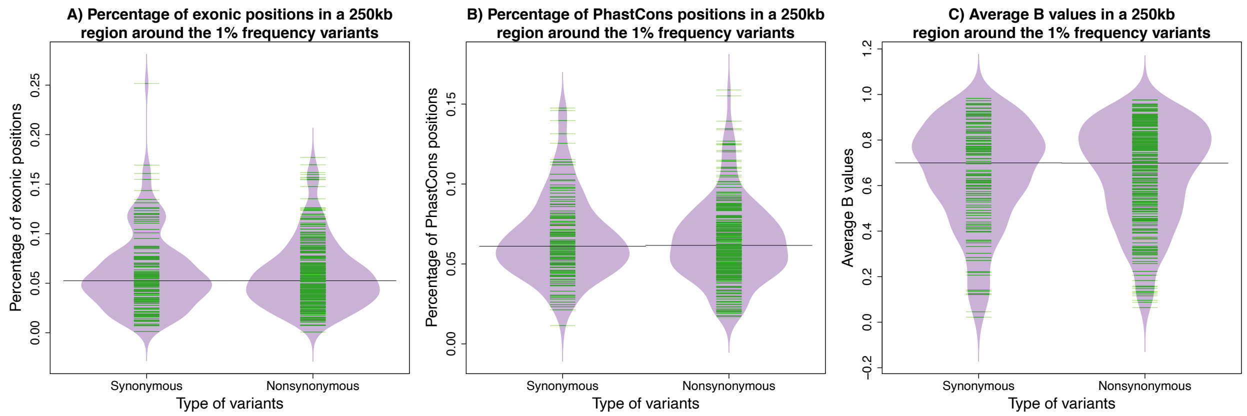

Figure S29

Percentage of exonic positions, percentage of *PhastCons* element positions and average *B* values in the 250 kb upstream and downstream region of the inspected 1% frequency variants.

A) The proportion of exonic positions for 1% frequency synonymous and nonsynonymous variants has a non-significant difference in mean values (Syn = 6.07%; Nonsyn = 5.84%; Mann-Whitney U test for mean differences p –value = 0.7667), similar medians (Syn = 5.25%; Nonsyn = 5.26%) and a non-significant difference in their variances (Syn = 0.151%; Nonsyn = 0.116%; Brown-Forsythe test for the difference in variances p-value = 0.3305), respectively.

B) The proportion of positions in a *PhastCons* element for 1% frequency synonymous and nonsynonymous variants have a very similar mean (Syn = 6.56%; Nonsyn = 6.37%; Mann-Whitney U test for mean differences p –value = 0.601), median (Syn = 6.11%; Nonsyn = 6.16%) and a non-significant difference in their variances (Syn = 0.064%; Nonsyn = 0.065%; Brown-Forsythe test for the difference in variances p-value = 0.7782).

C) The average *B* values for 1% frequency synonymous and nonsynonymous variants display a very similar mean (Syn = 65.29%; Nonsyn = 65.19%; Mann-Whitney U test for mean differences p –value = 0.9116), similar medians (Syn = 69.96%; Nonsyn = 69.88%), and a non-significant difference in their variances (Syn = 5.03%; Nonsyn = 4.78%; Brown-Forsythe test for the difference in variances p-value = 0.9161).

The exonic positions were taken from the UCSC table browser (Karolchik *et al.* 2003) using the following Table Browser controls: Clade = Mammal; Genome = Human; Assembly = hg19; Group = Genes and Gene Predictions; Track = UCSC Genes; Table = known gene; Region = Genome.

The positions of the PhastCons elements were obtained with these Table Browser controls: Clade = Mammal; Genome = Human; Assembly = hg19; Group = Comparative Genomics; Track = Conservation; Table = 100 Vert. El (phastConsElements100way); Region = Genome.

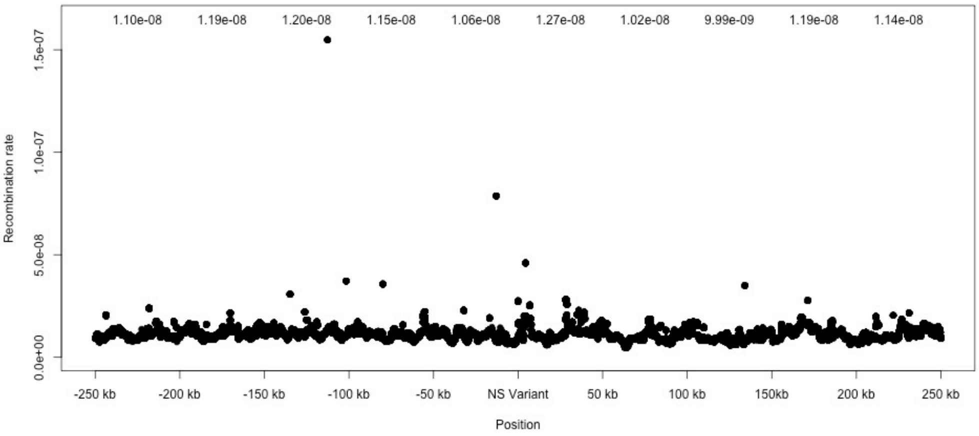
1.10e-08
1.19e-08
1.20e-08
1.15e-08
1.06e-08
1.27e-08
1.02e-08
9.99e-09
1.19e-08
1.14e-08
Recombination rate
0.0e+00
5.0e-08
1.0e-07
1.5e-07
-250 kb
-200 kb
-150 kb
-100 kb
-50 kb
NS Variant
50 kb
100 kb
150kb
200 kb
250 kb
Position

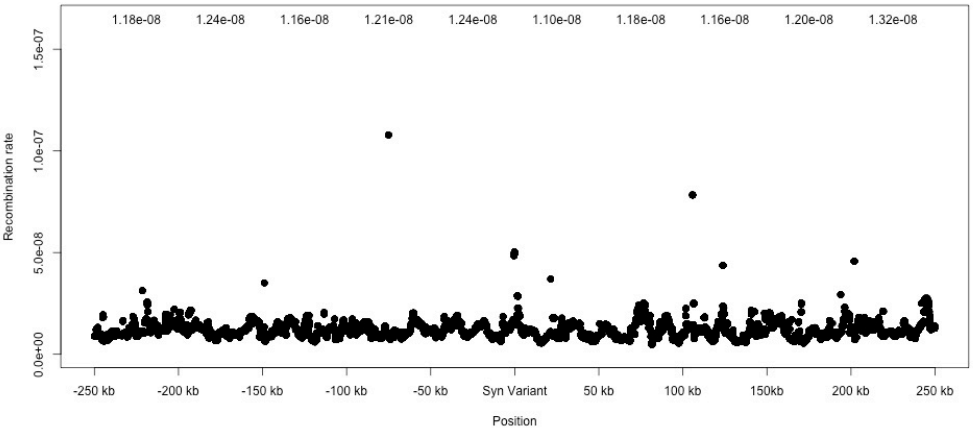
1.18e-08
1.24e-08
1.16e-08
1.21e-08
1.24e-08
1.10e-08
1.18e-08
1.16e-08
1.20e-08
1.32e-08
Recombination rate
0.0e+00
5.0e-08
1.0e-07
1.5e-07
-250 kb
-200 kb
-150 kb
-100 kb
-50 kb
Syn Variant
50 kb
100 kb
150kb
200 kb
250 kb
Position

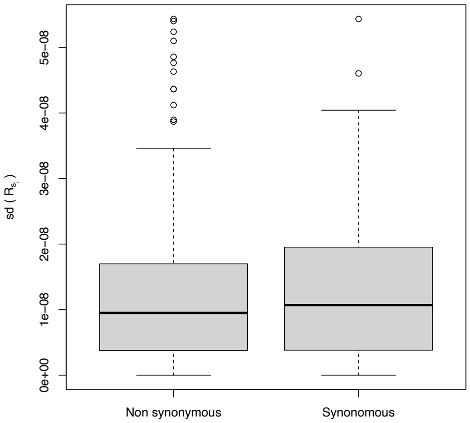
sd (Rsi)
0e+00
1e−08
2e−08
3e−08
4e−08
5e−08
Non synonymous
Synonomous

Figure S30

Distribution of recombination rate values in the vicinity of the synonymous and nonsynonymous sites.

Upper panel.- Mean recombination rate values 250 kb downstream and 250 kb upstream of the 275 1% nonsynonymous variants analyzed in the *UK10K* dataset. The black dots indicate the mean recombination values at each position upstream or downstream of the 275 nonsynonymous variants analyzed. The numbers shown at the top indicate the mean recombination values in each of the 10 $\boldsymbol{s_j}$ windows = {(-250kb, -200 kb), (-200kb, -150 kb), (-150kb, -100 kb), (-100kb, -50 kb), (-50kb, 0 kb), (0 kb, 50 kb), (50 kb, 100 kb), (100 kb, 150 kb), (150 kb, 200 kb), (200 kb, 250 kb)}.

Middle panel.- Mean recombination rate values 250 kb downstream and 250 kb upstream of the 152 1% synonymous variants analyzed in the *UK10K* dataset. The black dots indicate the mean recombination values at each position upstream or downstream of the 152 synonymous variants analyzed. The numbers shown at the top indicate the mean recombination values in each of the 10 $\boldsymbol{s_j}$ windows = {(-250 kb, -200 kb), (-200kb, -150 kb), (-150kb, -100 kb), (-100kb, -50 kb), (-50kb, 0 kb), (0 kb, 50 kb), (50 kb, 100 kb), (100 kb, 150 kb), (150 kb, 200 kb), (200 kb, 250 kb)}.

Bottom panel.- In the left boxplot we plot the standard deviation in the 5 mean recombination rates $\boldsymbol{R_{sj}}$ of the 5 windows downstream {(-250kb, -200 kb), (-200kb, -150 kb), (-150kb, -100 kb), (-100kb, -50 kb), (-50kb, 0 kb)} for each one of the 275 1% nonsynonymous variants analyzed. We also plot the standard deviation in the 5 mean recombination rates $\boldsymbol{R_{sj}}$ of the 5 windows upstream (0 kb, 50 kb), (50 kb, 100 kb), (100 kb, 150 kb), (150 kb, 200 kb), (200 kb, 250 kb)}.

The right boxplot shows the same analysis for the 152 1% synonymous variants.

We conclude that in the 250 kb region upstream and downstream of the inspected synonymous and nonsynonymous variants there is variation in the recombination rates, however the variation is not very large since the standard deviation is similar to the mean recombination rate across the whole region. Further, there is no clear positional effect such that recombination rates increase or decrease markedly with distance from the focal SNP. Finally, we observe the recombination rates are not different between non-synonymous and synonymous sites (mean recombination rate within 250 kb for nonsynonymous = $1.13\ X\ 10^{-8}$; mean recombination rate within 250 kb for S = $1{,}27\ X\ 10^{-8}$; p-value Mann-Whitney U Test = 0.1587).

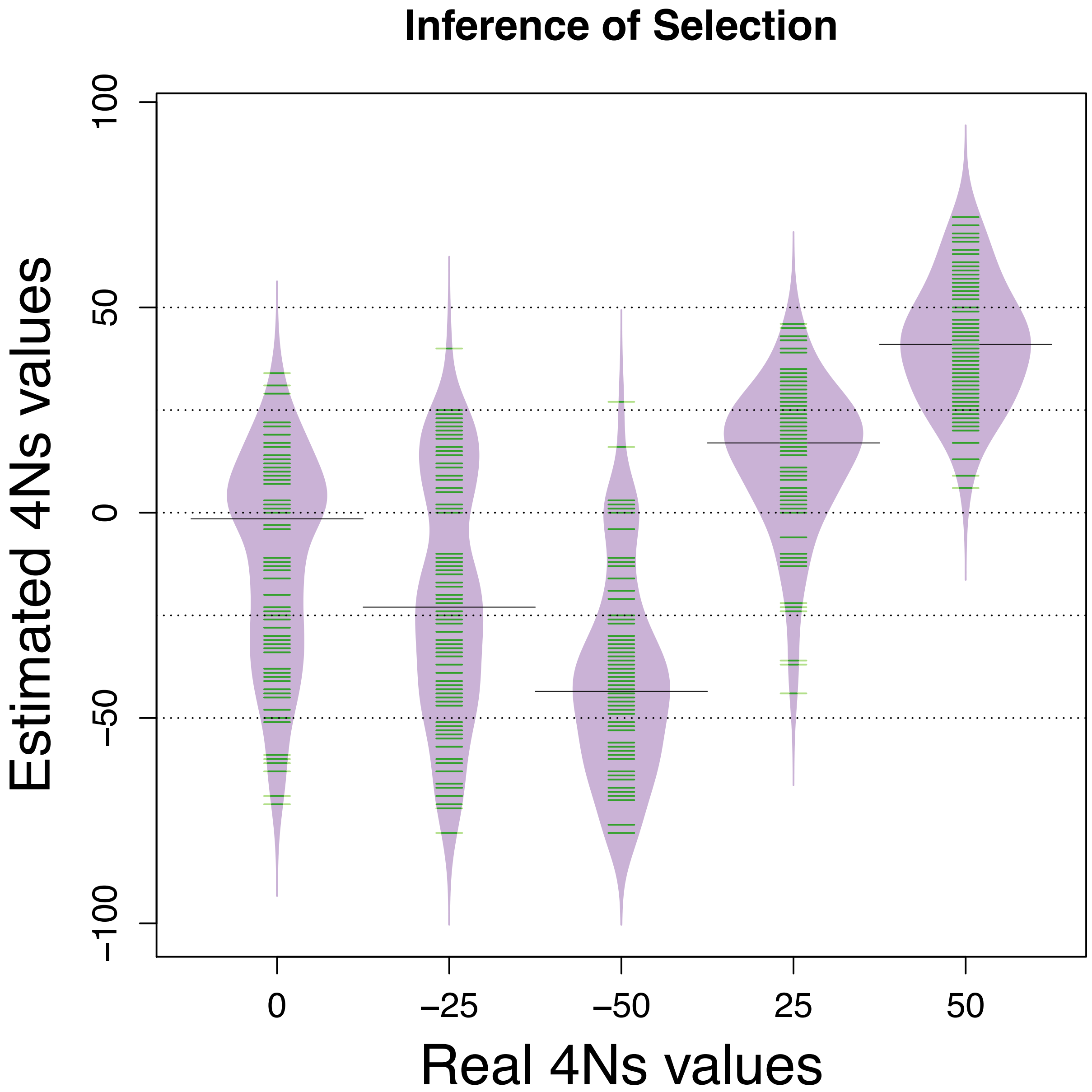

Figure S31

Estimation of the strength of natural selection under the demographic model inferred from the scaled *UK10K* dataset on simulations.

Each simulation replicate contains 275 independent loci with 69-76 haplotypes containing the derived allele ($\boldsymbol{f} = \mathbf{1}\% \pm \mathbf{0.05}\%$). We calculated $\boldsymbol{L}$ going upstream and downstream of the focal loci, obtaining $\boldsymbol{\ell} \approx \binom{\mathbf{72}}{\mathbf{2}} \times \mathbf{2} \times \mathbf{275}\,\boldsymbol{L}$ values for each simulation replicate.

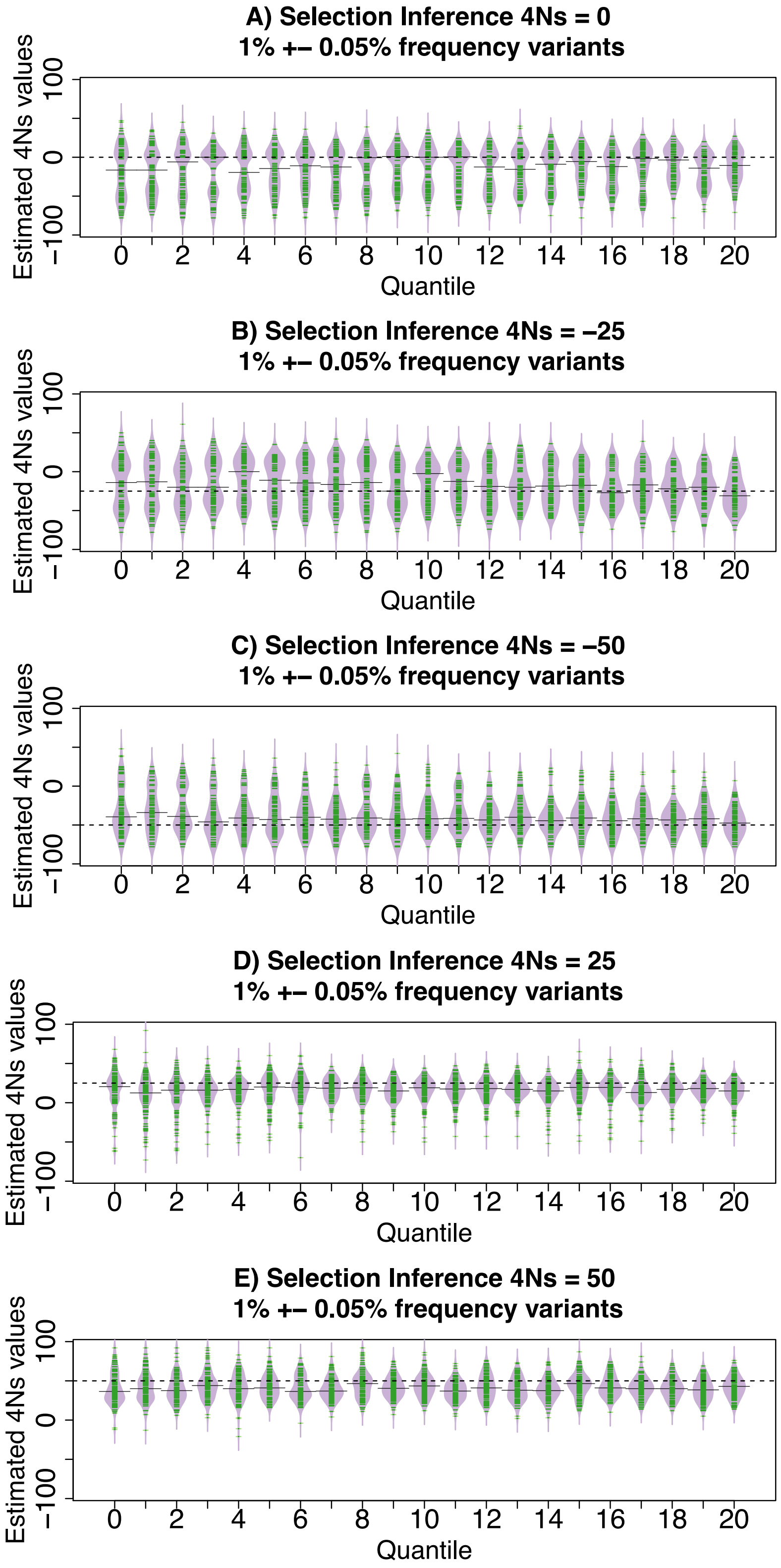
A) Selection Inference 4Ns = 0
1% +– 0.05% frequency variants
B) Selection Inference 4Ns = –25
1% +– 0.05% frequency variants
C) Selection Inference 4Ns = –50
1% +– 0.05% frequency variants
D) Selection Inference 4Ns = 25
1% +– 0.05% frequency variants
E) Selection Inference 4Ns = 50
1% +– 0.05% frequency variants
Estimated 4Ns values
Quantile
–100
0
100
0
2
4
6
8
10
12
14
16
18
20

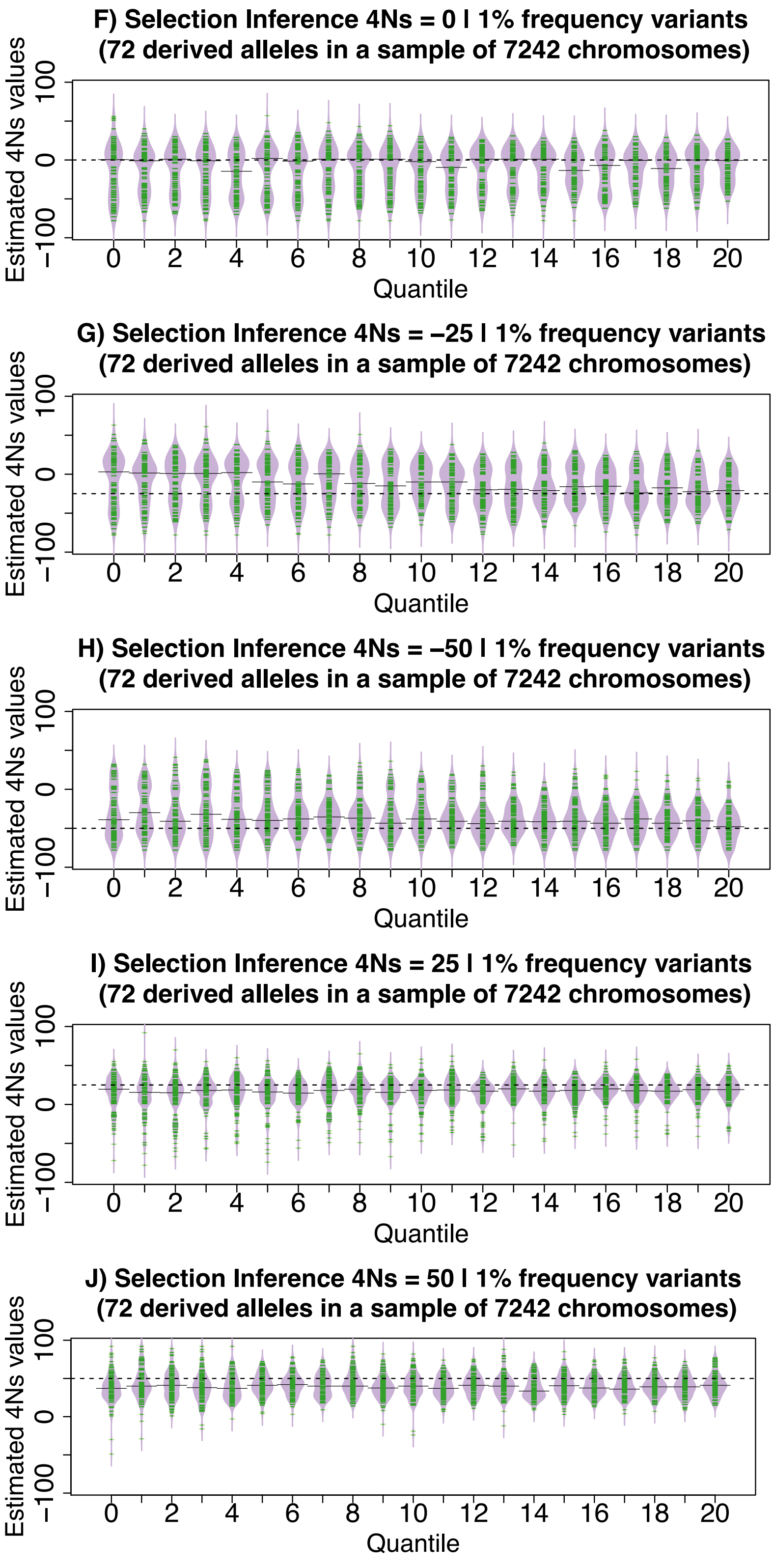
F) Selection Inference 4Ns = 0 l 1% frequency variants
(72 derived alleles in a sample of 7242 chromosomes)
Estimated 4Ns values
100
0
−100
0
2
4
6
8
10
12
14
16
18
20
Quantile
G) Selection Inference 4Ns = −25 l 1% frequency variants
(72 derived alleles in a sample of 7242 chromosomes)
Estimated 4Ns values
100
0
−100
0
2
4
6
8
10
12
14
16
18
20
Quantile
H) Selection Inference 4Ns = −50 l 1% frequency variants
(72 derived alleles in a sample of 7242 chromosomes)
Estimated 4Ns values
100
0
−100
0
2
4
6
8
10
12
14
16
18
20
Quantile
I) Selection Inference 4Ns = 25 l 1% frequency variants
(72 derived alleles in a sample of 7242 chromosomes)
Estimated 4Ns values
100
0
−100
0
2
4
6
8
10
12
14
16
18
20
Quantile
J) Selection Inference 4Ns = 50 l 1% frequency variants
(72 derived alleles in a sample of 7242 chromosomes)
Estimated 4Ns values
100
0
−100
0
2
4
6
8
10
12
14
16
18
20
Quantile

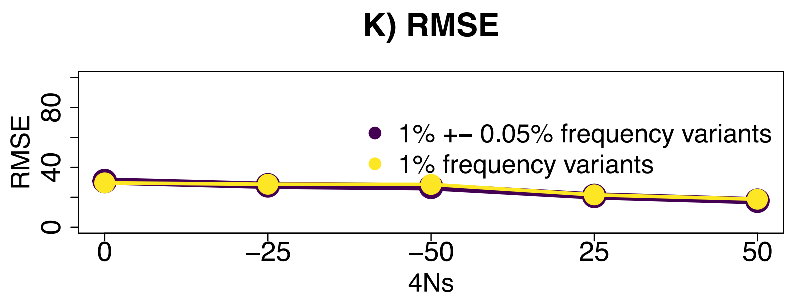

Figure S32

Inference of selection under the scaled *UK10K* demographic scenario for $\mathbf{5}$ different values of selection and $\mathbf{21}$ different recombination rates.

We inferred selection across 100 simulation replicates for each of the 21 recombination rates explored. The 21 different recombination rates used are the 21 different percentile values (0th, 5th, …, 95th, 100th) from the distribution of 550 average recombination rates per base taken from the upstream and downstream 250 kb regions next to the 275 nonsynonymous 1% frequency variants.

A-E) Each simulation replicate contains 275 independent loci with 69-76 haplotypes containing the derived allele ($\boldsymbol{f} = \mathbf{1}\% \pm \mathbf{0.05}\%$) and a fixed recombination rate for all the loci. We calculated $\boldsymbol{L}$ going upstream and downstream of the focal loci, obtaining $\boldsymbol{\ell} \approx \binom{\mathbf{72}}{\mathbf{2}} \times \mathbf{2} \times \mathbf{275}\,\boldsymbol{L}$ values for each simulation replicate.

F-J) Each simulation replicate contains 275 independent loci with 72 haplotypes containing the derived allele and a fixed recombination rate for all the loci. We calculated $\boldsymbol{L}$ going upstream and downstream of the focal loci, obtaining $\boldsymbol{\ell} = \binom{\mathbf{72}}{\mathbf{2}} \times \mathbf{2} \times \mathbf{275}\,\boldsymbol{L}$ values for each simulation replicate.

K) Root Mean Square Error (RMSE) for simulations done with 1% frequency variants (72 haplotypes with the derived allele in a sample of 7242 chromosomes) or with variants that have a $f = 1\% \pm 0.05\%$ frequency. The points shown for each 4Ns value show the RMSE taking into account the simulations done under the 21 different recombination rates.

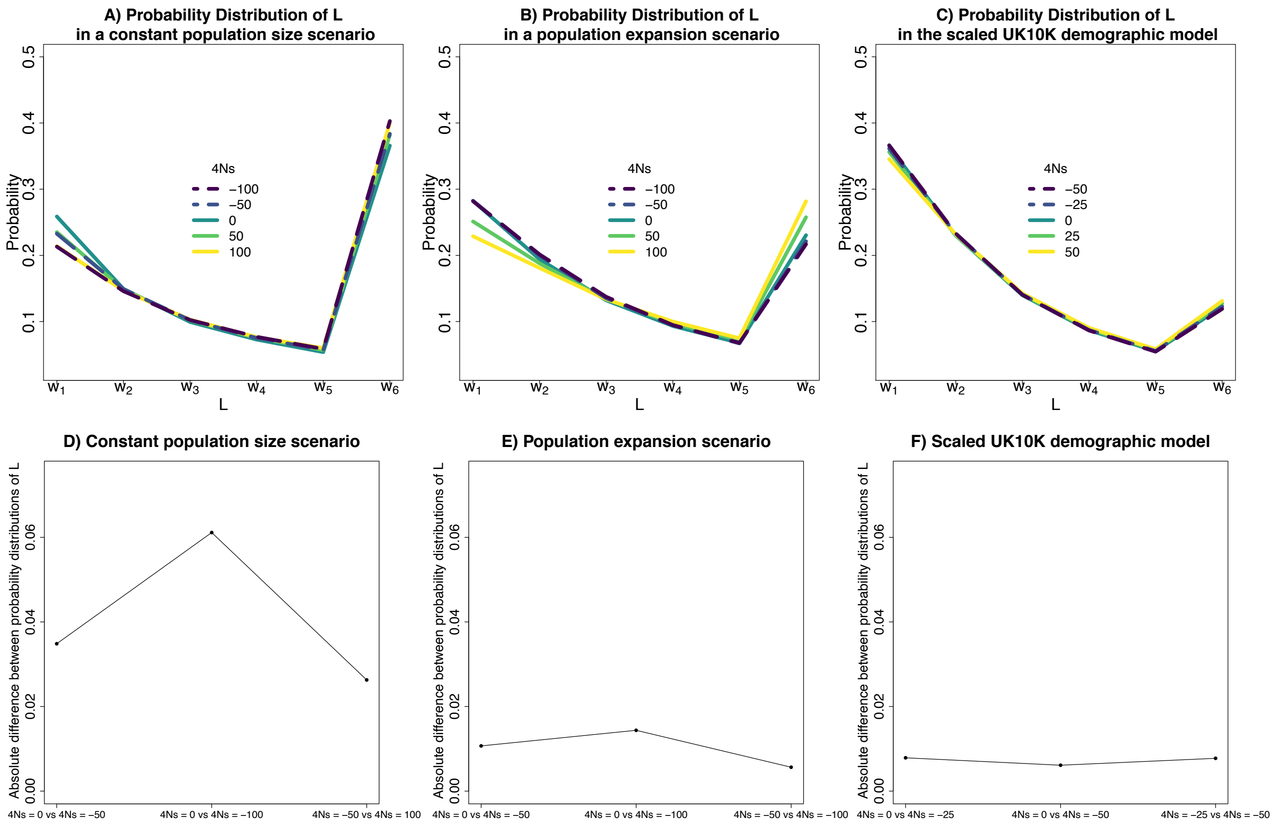

Figure S33

Changes in the probability distributions of $P(L \in w_i|f, D, 4Ns)$ on different demographic models using A = 30,000 simulated independent $f = 1\%$ variants with n = 40 for panels A), B), D) and E), and A=27,500 simulated independent $f = 1\%$ variants with n = 72 for panels C) and F). A per base mutation rate of 1.2e-8 and a per base recombination rate of 1e-8 was used for panels A), B), D) and E) while a mutation rate of 1.5e-8 * 5 and a per base recombination rate of 8.25e-9 * 5 (the median per base recombination rate from the 275 upstream and downstream regions of the 1%+-0.05% 275 nonsynonymous variants analyzed) for panels C) and F). The 5 factor that is multiplied in the scaled *UK10K* demographic model is used to make the values of θ and the ρ the same as in the *UK10K* demographic model (not-scaled). The *UK10K* haplotype panel does not contain singleton variants. Therefore, the calculation of L in this step is done ignoring singleton variants in the sample of haplotypes with the derived allele.

Upper panels) Probability distributions of $P(L \in w_i|f, D, 4Ns)$ under different values of $4Ns$ in three different demographic scenarios. The label 'Probability' refers to $P(L \in w_i|f, D, 4Ns)$ in the upper panel.
Lower panels) Absolute difference between probability distributions with different $4Ns$ values x and y. This is calculated as $\sum_{i=1}^{6} |P(L \in w_i|f, D, 4Ns = x) - P(L \in w_i|f, D, 4Ns = y)|$.

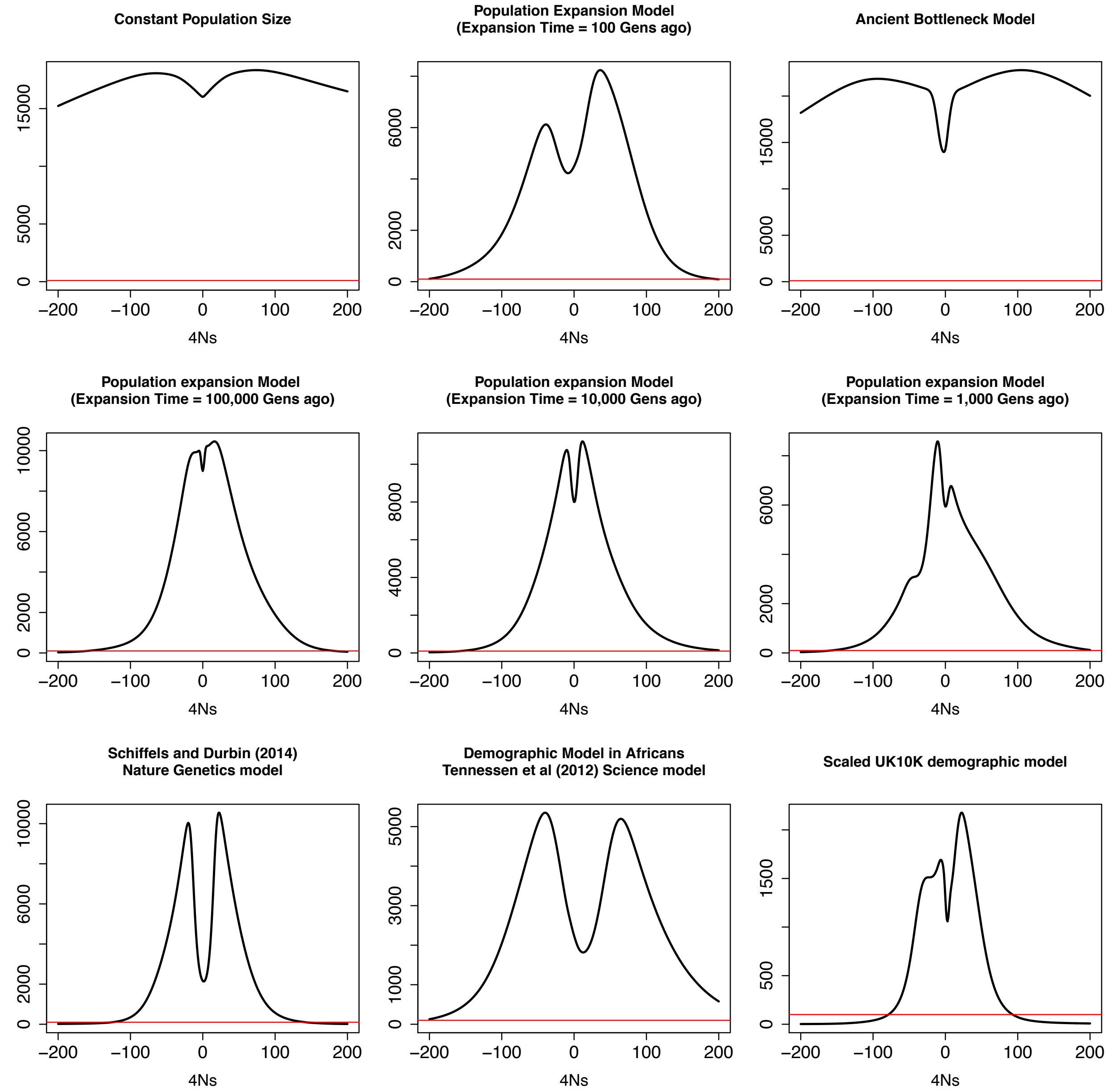

Figure S34

Effective Sample Sizes (*ESS*) under nine different demographic models using $100,000$ simulated allele frequency trajectories from our proposal distribution.

Demographic models:

Constant population size model (Inference results shown in Figure 3); Population expansion model (Expansion Time = 100 Gens Ago) (Inference results shown in Figure 5 and S3); Ancient Bottleneck Model (Inference results shown in Figure S2); Population expansion model (Expansion Time = $100{,}000$ or $10{,}000$ or $1{,}000$ Gens Ago) (Inference results shown in S2); Schiffels and Durbin (2014) Nature Genetics Model (Inference results shown in S4); Demographic Model in Africans Tennessen et al (2012) Science model (Inference results shown in S4); Scaled UK10K demographic model (Inference results shown in Figure S31-S32).

The red line shown in each plot denotes the value of ESS = 100.

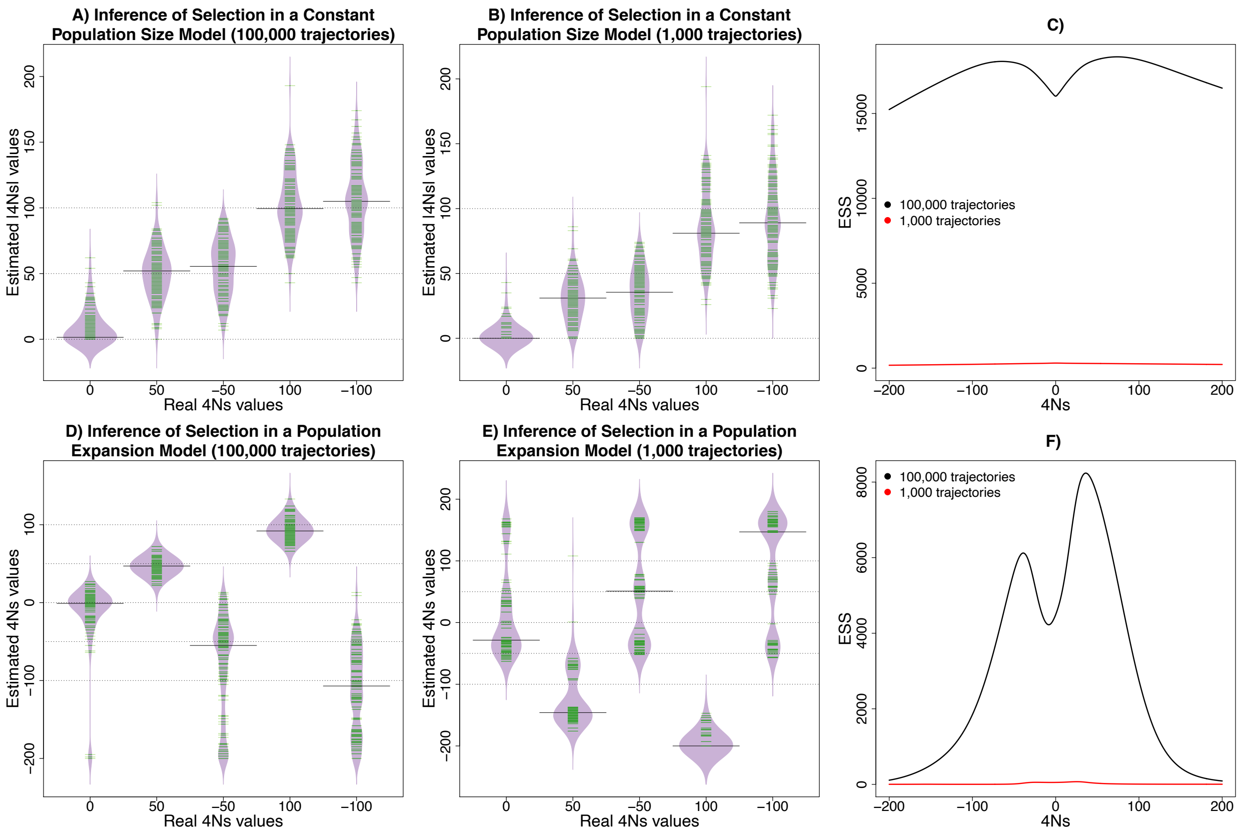

Figure S35

The impact of the *ESS* on the estimates of $4Ns$.

We evaluated our estimates of the strength of natural selection ($4Ns$) under two different demographic scenarios, a constant population size model and a population expansion model.

A) and B) Estimates of $4Ns$ under a constant population size model using 100,000 and 1,000 trajectories, respectively, to perform the integration over the space of allele frequency trajectories using our importance sampling approach. The estimates of $4Ns$ show a small bias in B), where the value of *ESS* is smaller as seen in C).

D) and E) Estimates of $4Ns$ under a constant population size model using 100,000 and 1,000 trajectories, respectively, to perform the integration over the space of allele frequency trajectories using our importance sampling approach. The estimates of $4Ns$ are biased in E), where the value of *ESS* is smaller as seen in F).

Using a small number of trajectories to perform the integration over the space of allele frequency trajectories leads to small *ESS* values, as shown here. Here we show that using a small number of trajectories leads to small *ESS* values which, in turn, lead to inaccurate estimates of $4Ns$.

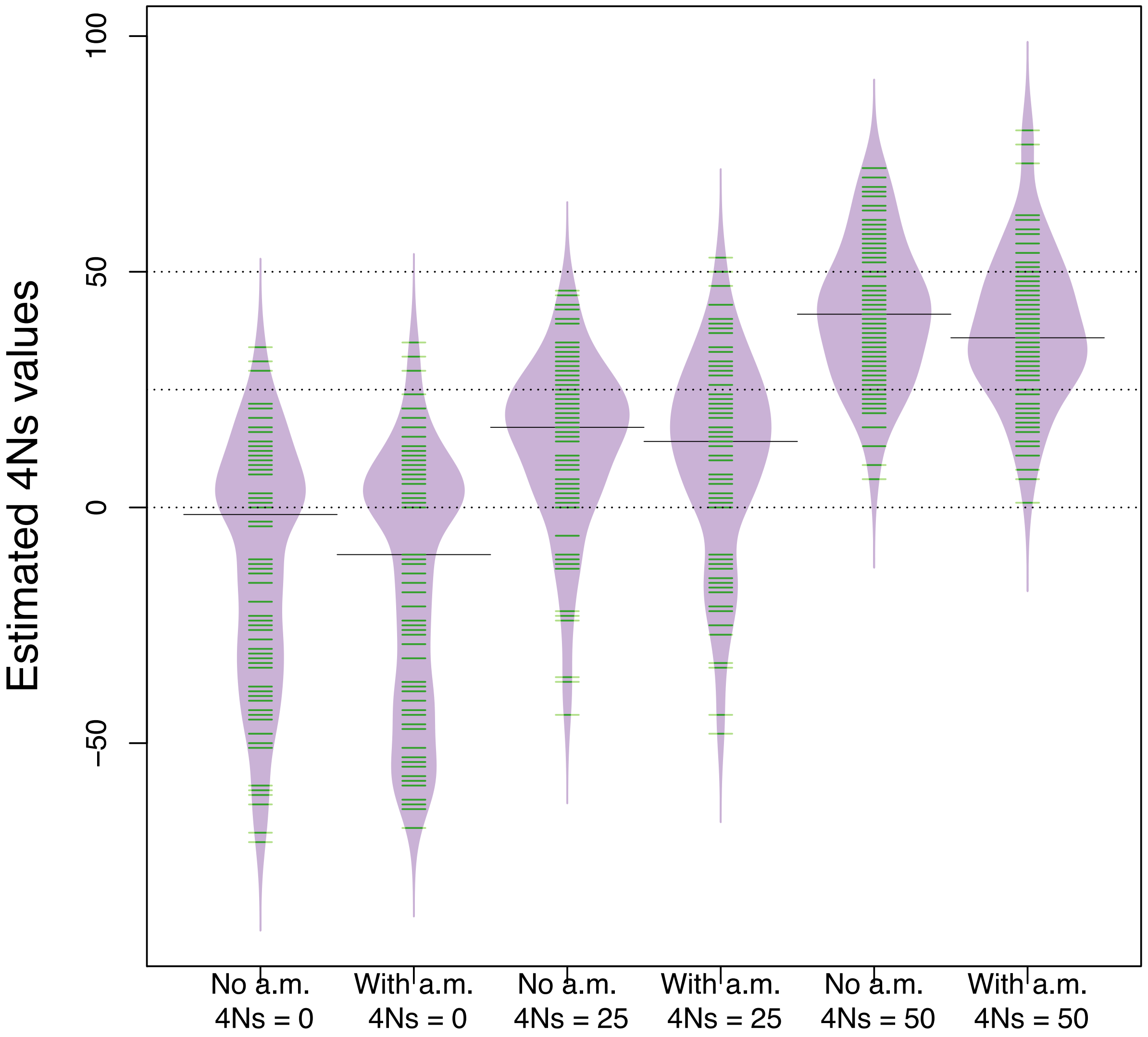

Figure S36

Inference of selection in the scaled *UK10K* demographic scenario including ancestral state misspecification in simulations.

Each simulation replicate contains 275 independent loci with 69-76 haplotypes containing the derived allele **(**$f = 1\% \pm 0.05\%$**)**. We calculated L going upstream and downstream of the focal loci, obtaining $\ell \approx \binom{72}{2} \times 2 \times 275$ L values for each simulation replicate.

The green lines indicate one estimated value of $4Ns$. 'Estimated $4Ns$ values' refers to the values estimated by our method. The median value of the estimates of $4Ns$ is shown with a solid line. The dashed line matches the $4Ns$ value used in the simulations.

We used a multinomial distribution to define the number of variants with 69 up to 76 derived alleles in each simulation replicate, along with the number of variants where a 1% frequency ancestral allele was missclassifed as a 1% frequency derived allele. To do this, we used a multinomial distribution where we sampled 275 numbers. We first define the number of trajectories M_i that ended up with a n_i value equal to 69, 70, 71, 72, 73, 74, 75 or 76 in $1{,}700$ PReFerSim simulations done with a Poisson mean number of

100 new mutations in the first epoch of the demographic model. The expected number of misspecified 1% frequency ancestral alleles M_{A_i} depends on *P_{incorrect}(f)* from Table S6 and is equal to $M_{A_i} = \frac{M_i}{1-P_{incorrect}(f)} - M_i$. The probability of sampling a variant with n_i derived alleles is equal to $M_i/(\sum_{i=69}^{76} M_i + M_{A_i})$ and the probability of sampling a variant with n_i ancestral alleles is equal to $M_{A_i}/(\sum_{i=69}^{76} M_i + M_{A_i})$. We sampled 275 variants per simulation replicate.

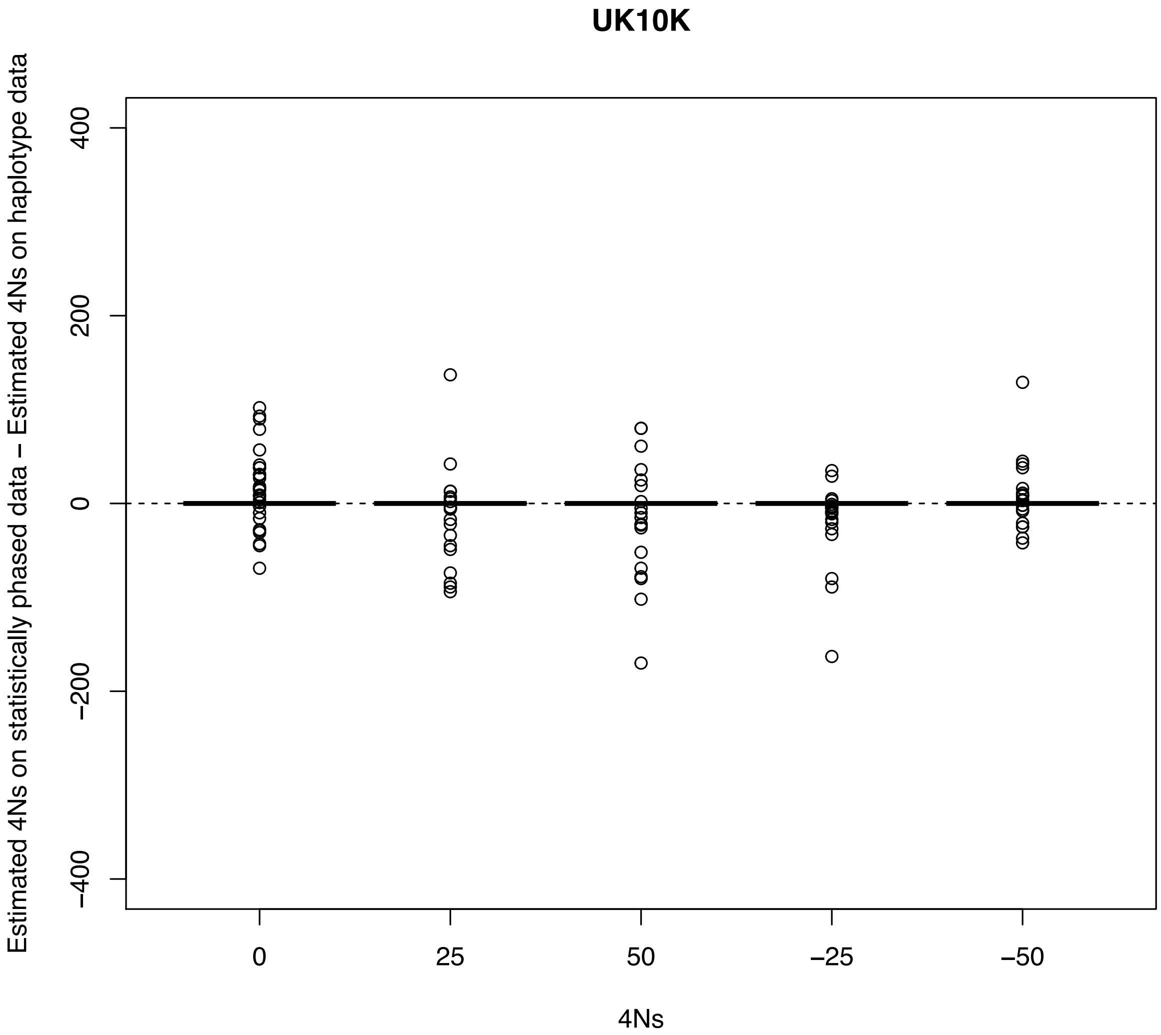

Figure S37

Biases in the estimated values of $4Ns$ in the scaled *UK10K* demographic model.

We estimated the value of $4Ns$ for each simulation two times. First, we estimated the value of $4Ns$ on the data when the haplotype phase is known. We refer to those estimates as the 'Estimated $4Ns$ on haplotype data'. Then, we estimated the value of $4Ns$ when we statistically phased the data (command shown in the past figure) and we refer to those $4Ns$ estimates as 'Estimated $4Ns$ on statistically phased data'. We computed the difference between 'Estimated $4Ns$ on haplotype data' and 'Estimated $4Ns$ on haplotype data' on 100 simulations done for simulations done under 5 different $4Ns$ values with 69-76 haplotypes containing the derived allele **(** $f = 1\% \pm 0.05\%$ **)**. Each simulation is composed of approximately $2 \times \binom{72}{2} = 2{,}556$ $\boldsymbol{L}$ values. In each simulation, we created a set of $3{,}621$ individuals that randomly contained either a derived or ancestral focal allele. There were 69-76 haplotypes containing the derived focal allele and 7166-7173 haplotypes with the ancestral focal allele. Then, we performed the statistical phasing using the program ShapeIT2:

```
shapeit.v2.904.3.10.0-693.11.6.el7.x86_64/bin/shapeit --input-vcf VCF1.vcf -M
GeneticMap.txt -O TestShapeIt.phased
```

Where VCF1.vcf is a vcf file containing the unphased genotype information for $3{,}621$ individuals ($7{,}242$ chromosomes); GeneticMap.txt is the genetic map of the region analyzed. The recombination rate was

sampled randomly from the 550 recombination rates upstream or downstream of the 275 1% frequency variants in the UK10K dataset; and TestShapeIt.phased is the file with the statistically phased haplotypic information.

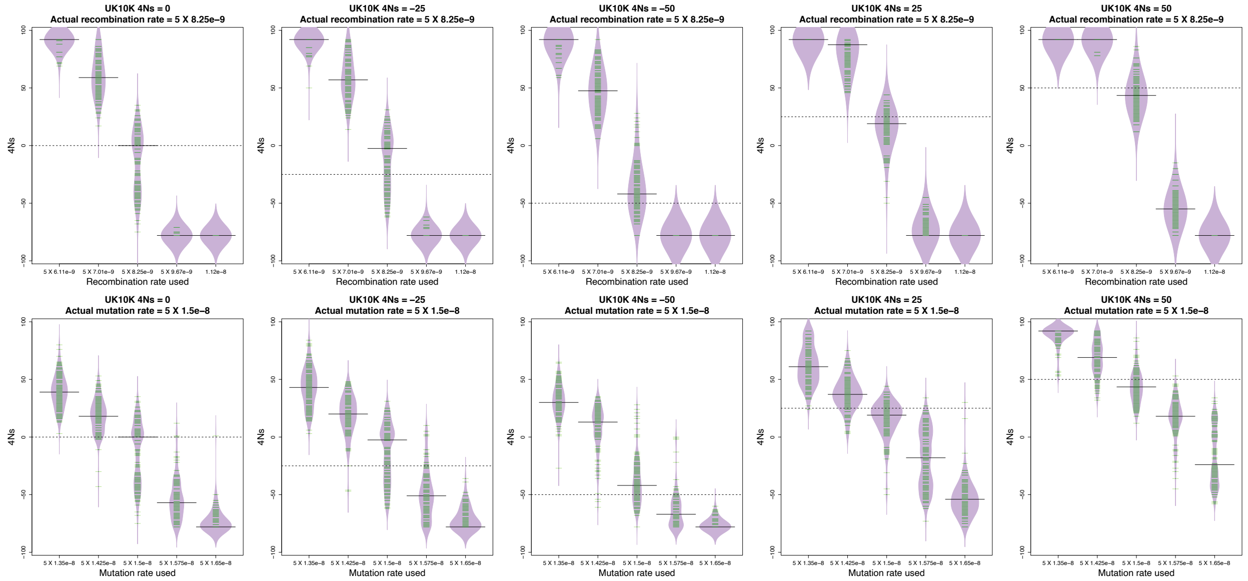

Figure S38

Biases in the estimation of $4Ns$ values due to a mutation rate or recombination rate misspecification in the scaled *UK10K* model.

We estimated the likelihood function $\mathcal{L}(4Ns, f, D|L \in w_{m_j})$ using a mutation rate per base per generation equal to $5 \times 1.5 \times 10^{-8}$ and a recombination rate per base per generation equal to $5 \times 8.25 \times 10^{-9}$. Then, we performed 100 simulation replicates for each combination of five different mutation rates and $4Ns$ values (upper panel); or a combination of five different recombination rates and $4Ns$ values (lower panel). Each simulation replicate contained $\ell \approx 2 \times A \times \binom{n}{2} \approx 2 \times 275 \times \binom{72}{2}$ realized values of $\boldsymbol{L}$. We estimated the value of $4Ns$ in each simulation replicate using the likelihood function $\mathcal{L}(4Ns, f, D|L \in w_{m_j})$ generated with a mutation rate per base per generation equal to $5 \times 1.5 \times 10^{-8}$ and a recombination rate per base per generation equal to $5 \times 8.25 \times 10^{-9}$. We could assess the effect of mutation rate and recombination rate misspecification because 4 out of the 5 mutation rates and recombination rates employed were different from the parameters used to generate the likelihood function $\mathcal{L}\left(4Ns, f, D\middle|L \in w_{m_j}\right)$.

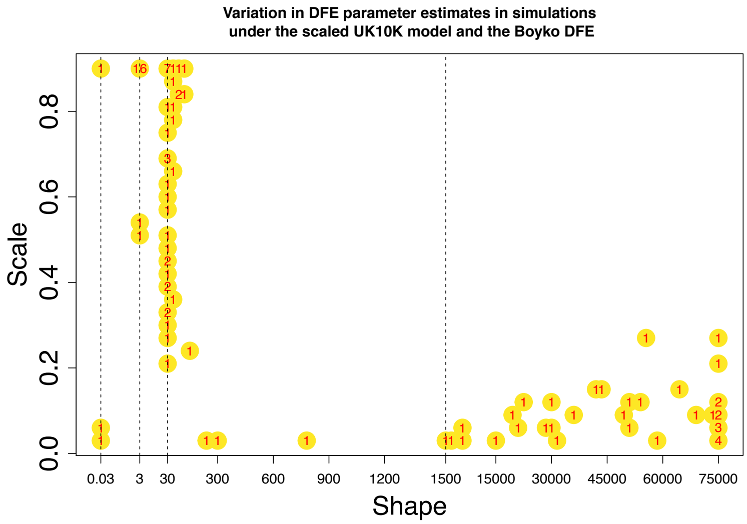
Variation in DFE parameter estimates in simulations
under the scaled UK10K model and the Boyko DFE
Scale
0.0
0.2
0.4
0.6
0.8
Shape
0.03
3
30
300
600
900
1200
1500
15000
30000
45000
60000
75000

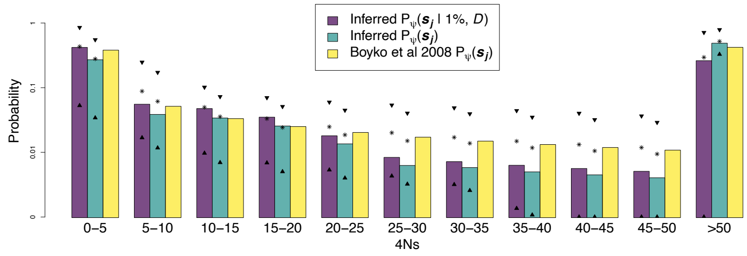
Inferred $P_\psi(s_j \mid 1\%, D)$
Inferred $P_\psi(s_j)$
Boyko et al 2008 $P_\psi(s_j)$
Probability
0–5
5–10
10–15
15–20
20–25
25–30
30–35
35–40
40–45
45–50
>50
4Ns

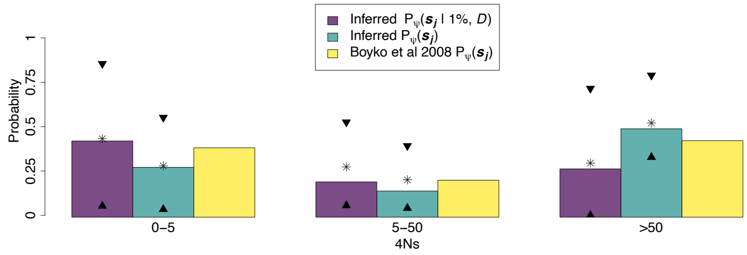
Inferred $P_\psi(s_j \mid 1\%, D)$
Inferred $P_\psi(s_j)$
Boyko et al 2008 $P_\psi(s_j)$
Probability
0
0.25
0.5
0.75
1
0–5
5–50
>50
4Ns

Figure S39

Variation in estimates of the distribution of fitness effects in $\mathbf{100}$ sets of simulations of $\mathbf{275}\ \boldsymbol{f} = \mathbf{1\%} \pm \mathbf{0.05\%}$ frequency variants. We simulated these variants under the scaled *UK10K* demographic model and the Boyko et al. 2008 distribution of fitness effects. The upstream and downstream recombination rates were assigned based on the average per base recombination rate in the $\mathbf{250}$ kb region surrounding each variant in the $\mathbf{275} \pm \mathbf{0.05\%}$ frequency variants in the *UK10K* dataset.

On the top we show the estimated scale and shape parameters of the compound estimated DFE in 100 sets of simulations replicates. The red numbers indicate the number of bootstrap replicates found in each combination of scale and shape parameters.

On the middle panel we compare the estimated values of 'Inferred $P_\psi(\boldsymbol{s_j}|1\%, D)$', 'Inferred $P_\psi(\boldsymbol{s_j})$' against the value of 'Boyko et al 2008 $P_\psi(\boldsymbol{s_j})$'. To calculate 'Inferred $P_\psi(\boldsymbol{s_j})$' from the 'Inferred $P_\psi(\boldsymbol{s_j}|f, D)$' we used equation (6), calculating $P_\psi(f|D)$ from a set of approximately $12.32\ \mathrm{X}\ 10^{10}$ variants simulated with *PReFerSim* under the scaled *UK10K* demographic model and the Boyko distribution of fitness effects; and $P_\psi(f|\boldsymbol{s_j}, D)$ from a set of approximately $12.32\ \mathrm{X}\ 10^{10}$ variants simulated with *PReFerSim* under the scaled *UK10K* demographic model and the mice distribution of fitness effects. The median values of 'Inferred $P_\psi(\boldsymbol{s_j}|f, D)$' and 'Inferred $P_\psi(\boldsymbol{s_j})$' across 100 simulations are shown in the bars across each $\boldsymbol{s_j}$ interval. The bar from 'Boyko et al 2008 $P_\psi(\boldsymbol{s_j})$' is the value inferred for each $\boldsymbol{s_j}$ interval based on the DFE inferred in the Boyko et al 2008 paper. The two triangles shown in each $\boldsymbol{s_j}$ interval denote the 5% and 95% percentile of the 'Inferred $P_\psi(\boldsymbol{s_j})$' or '$P_\psi(\boldsymbol{s_j}|f, D)$' probabilities estimated across the 100 simulation replicates. The asterisk signs from 'Inferred $P_\psi(\boldsymbol{s_j})$' and '$P_\psi(\boldsymbol{s_j}|f, D)$' probabilities are the mean values calculated from 100 simulation replicates. The actual estimate of 'Boyko et al 2008 $P_\psi(\boldsymbol{s_j})$' is inside the 5% and 95% percentile of the 'Inferred $P_\psi(\boldsymbol{s_j})$' values across all $\boldsymbol{s_j}$ intervals. Note that the mean value of 'Inferred $P_\psi(\boldsymbol{s_j})$' is closer to the true value of 'Boyko et al 2008 $P_\psi(\boldsymbol{s_j})$' than the median value of 'Inferred $P_\psi(\boldsymbol{s_j})$' across all $\boldsymbol{s_j}$ intervals .

In the bottom panel, we also compare the estimated values of 'Inferred $P_\psi(\boldsymbol{s_j}|f, D)$', 'Inferred $P_\psi(\boldsymbol{s_j})$' against the value of 'Boyko et al 2008 $P_\psi(\boldsymbol{s_j})$'. To calculate 'Inferred $P_\psi(\boldsymbol{s_j}|f, D)$', 'Inferred $P_\psi(\boldsymbol{s_j})$' and 'Boyko et al 2008 $P_\psi(\boldsymbol{s_j})$' for the middle bin $\boldsymbol{s_j} \in [5,50)$ we summed the values of those three respective probabilities across eight $\boldsymbol{s_j}$intervals: $\boldsymbol{s_j} \in [5, 10)$, $\boldsymbol{s_j} \in [10, 15)$, $\boldsymbol{s_j} \in [15,20)$, $\boldsymbol{s_j} \in [20,25)$, $\boldsymbol{s_j} \in [25,30)$, $\boldsymbol{s_j} \in [30,35)$, $\boldsymbol{s_j} \in [35,40)$, $\boldsymbol{s_j} \in [40,45)$ and $\boldsymbol{s_j} \in [45,50)$. Note that here the y-axis is plotted on a linear scale while in the middle panel we used a logarithmic scale. The two triangles shown in each $\boldsymbol{s_j}$ interval denote the 5% and 95% percentile of the 'Inferred $P_\psi(\boldsymbol{s_j})$' or '$P_\psi(\boldsymbol{s_j}|f, D)$' probabilities estimated across 100 simulation replicates. The asterisk signs from 'Inferred $P_\psi(\boldsymbol{s_j})$' and '$P_\psi(\boldsymbol{s_j}|f, D)$' probabilities are the mean values calculated from 100 simulation replicates.

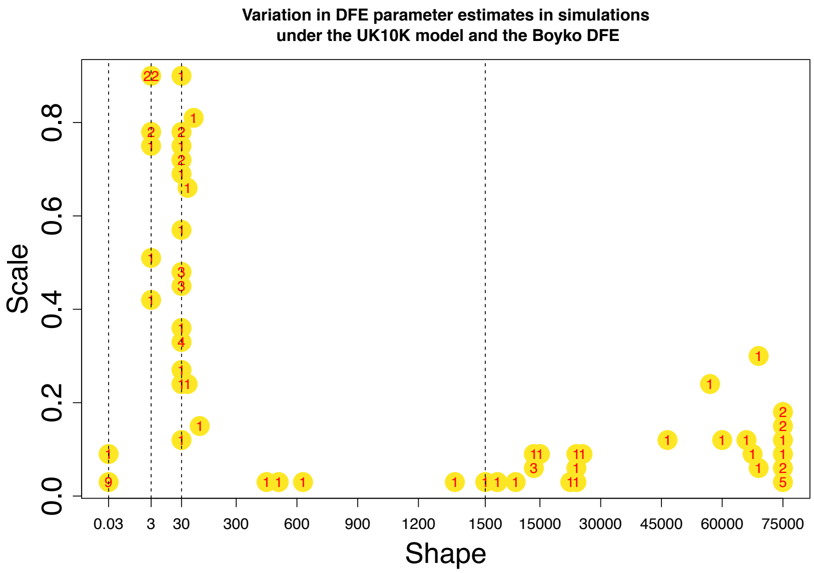

Variation in DFE parameter estimates in simulations
under the UK10K model and the Boyko DFE
Scale
Shape
0.03
3
30
300
600
900
1200
1500
15000
30000
45000
60000
75000
0.0
0.2
0.4
0.6
0.8

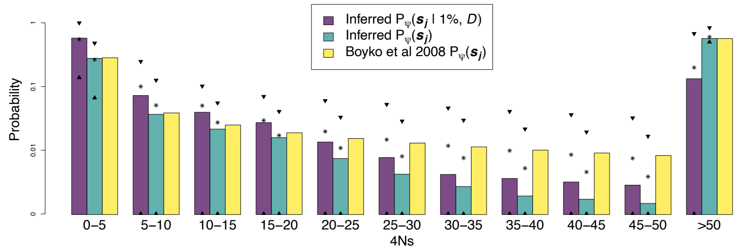

Inferred $P_\psi(\boldsymbol{s_j} \mid 1\%, D)$
Inferred $P_\psi(\boldsymbol{s_j})$
Boyko et al 2008 $P_\psi(\boldsymbol{s_j})$
Probability
0–5
5–10
10–15
15–20
20–25
25–30
30–35
35–40
40–45
45–50
>50
4Ns

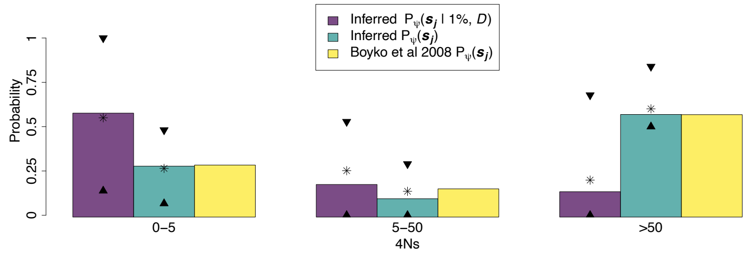

Inferred $P_\psi(\boldsymbol{s_j} \mid 1\%, D)$
Inferred $P_\psi(\boldsymbol{s_j})$
Boyko et al 2008 $P_\psi(\boldsymbol{s_j})$
Probability
0
0.25
0.5
0.75
1
0–5
5–50
>50
4Ns

Figure S40

Variation in estimates of the distribution of fitness effects in $\mathbf{100}$ sets of simulations of $\mathbf{275}\ \boldsymbol{f} = \mathbf{1\%} \pm \mathbf{0.05\%}$ frequency variants with $\mathbf{72}$ haplotypes containing the derived allele in each variant.

We simulated these variants under the *UK10K* demographic model and the Boyko et al. 2008 distribution of fitness effects. Here we used the *UK10K* model instead of the scaled *UK10K* model employed in Supplementary Figure S30. The inference of $P_\psi(s_j|f,D)$ was performed using Equation 4 and the scaled *UK10K* demographic model. This mimics the analysis performed in the *UK10K* dataset, where the inferences of DFE_f, and therefore $P_\psi(s_j|f,D)$, were performed using the scaled *UK10K* demographic model while the estimation of $P_\psi(f|D)$ and $P_\psi(f|s_j,D)$ was done using the *UK10K* model. The upstream and downstream recombination rates were assigned based on the average per base recombination rate in the 250 kb region surrounding each variant in the 275 1% frequency variants.

On the top we show the estimated scale and shape parameters of the compound estimated DFE in 100 sets of simulations replicates. The red numbers indicate the number of bootstrap replicates found in each combination of scale and shape parameters. Note that here we are fitting the parameters of the DFE_f to a gamma distribution. However, although the DFE is simulated from a gamma distribution there is no guarantee that the DFE_f follows a gamma distribution.

On the middle panel we compare the estimated values of 'Inferred $P_\psi(\boldsymbol{s_j}|f,D)$', 'Inferred $P_\psi(\boldsymbol{s_j})$' against the value of 'Boyko et al 2008 $P_\psi(\boldsymbol{s_j})$'. To calculate 'Inferred $P_\psi(\boldsymbol{s_j})$' from the 'Inferred $P_\psi(\boldsymbol{s_j}|f,D)$' we used equation (4), calculating $P_\psi(f|D)$ from a set of approximately $53..39\ \mathrm{X}\ 10^{10}$ variants simulated with *PReFerSim* under the *UK10K* demographic model and the Boyko distribution of fitness effects; and $P_\psi(f|\boldsymbol{s_j},D)$ from a set of approximately $61.60\ \mathrm{X}\ 10^{9}$ variants simulated with *PReFerSim* under the scaled *UK10K* demographic model and the Mouse distribution of fitness effects. The median values of 'Inferred $P_\psi(\boldsymbol{s_j}|f,D)$' and 'Inferred $P_\psi(\boldsymbol{s_j})$' across 100 simulations are shown in the bars across each $\boldsymbol{s_j}$ interval. The bar from 'Boyko et al 2008 $P_\psi(\boldsymbol{s_j})$' is the value inferred for each $\boldsymbol{s_j}$ interval based on the DFE inferred in the Boyko et al 2008 paper. The two triangles shown in each $\boldsymbol{s_j}$ interval denote the 5% and 95% percentile of the 'Inferred $P_\psi(\boldsymbol{s_j})$' or '$P_\psi(\boldsymbol{s_j}|f,D)$' probabilities estimated across the 100 simulation replicates. The asterisk signs from 'Inferred $P_\psi(\boldsymbol{s_j})$' and '$P_\psi(\boldsymbol{s_j}|f,D)$' probabilities are the mean values calculated from 100 simulation replicates. The actual estimate of 'Boyko et al 2008 $P_\psi(\boldsymbol{s_j})$' is inside the 5% and 95% percentile of the 'Inferred $P_\psi(\boldsymbol{s_j})$' values across all $\boldsymbol{s_j}$ intervals. Note that the mean value of 'Inferred $P_\psi(\boldsymbol{s_j})$' is closer to the true value of 'Boyko et al 2008 $P_\psi(\boldsymbol{s_j})$' than the median value of 'Inferred $P_\psi(\boldsymbol{s_j})$' across all $\boldsymbol{s_j}$ intervals .

In the bottom panel, we also compare the estimated values of 'Inferred $P_\psi(\boldsymbol{s_j}|f,D)$', 'Inferred $P_\psi(\boldsymbol{s_j})$' against the value of 'Boyko et al 2008 $P_\psi(\boldsymbol{s_j})$'. To calculate 'Inferred $P_\psi(\boldsymbol{s_j}|f,D)$', 'Inferred $P_\psi(\boldsymbol{s_j})$' and 'Boyko et al 2008 $P_\psi(\boldsymbol{s_j})$' for the middle bin $\boldsymbol{s_j} \in [5,50)$ we summed the values of those three respective probabilities across eight $\boldsymbol{s_j}$intervals: $\boldsymbol{s_j} \in [5,10)$, $\boldsymbol{s_j} \in [10,15)$, $\boldsymbol{s_j} \in [15,20)$, $\boldsymbol{s_j} \in [20,25)$, $\boldsymbol{s_j} \in [25,30)$, $\boldsymbol{s_j} \in [30,35)$, $\boldsymbol{s_j} \in [35,40)$, $\boldsymbol{s_j} \in [40,45)$ and $\boldsymbol{s_j} \in [45,50)$. Note that here the y-axis is plotted on a linear scale while in the middle panel we used a logarithmic scale. The two triangles shown in each $\boldsymbol{s_j}$ interval denote the 5% and 95% percentile of the 'Inferred $P_\psi(\boldsymbol{s_j})$' or '$P_\psi(\boldsymbol{s_j}|f,D)$' probabilities estimated across 100 simulation replicates. The asterisk signs from 'Inferred $P_\psi(\boldsymbol{s_j})$' and '$P_\psi(\boldsymbol{s_j}|f,D)$' probabilities are the mean values calculated from 100 simulation replicates.

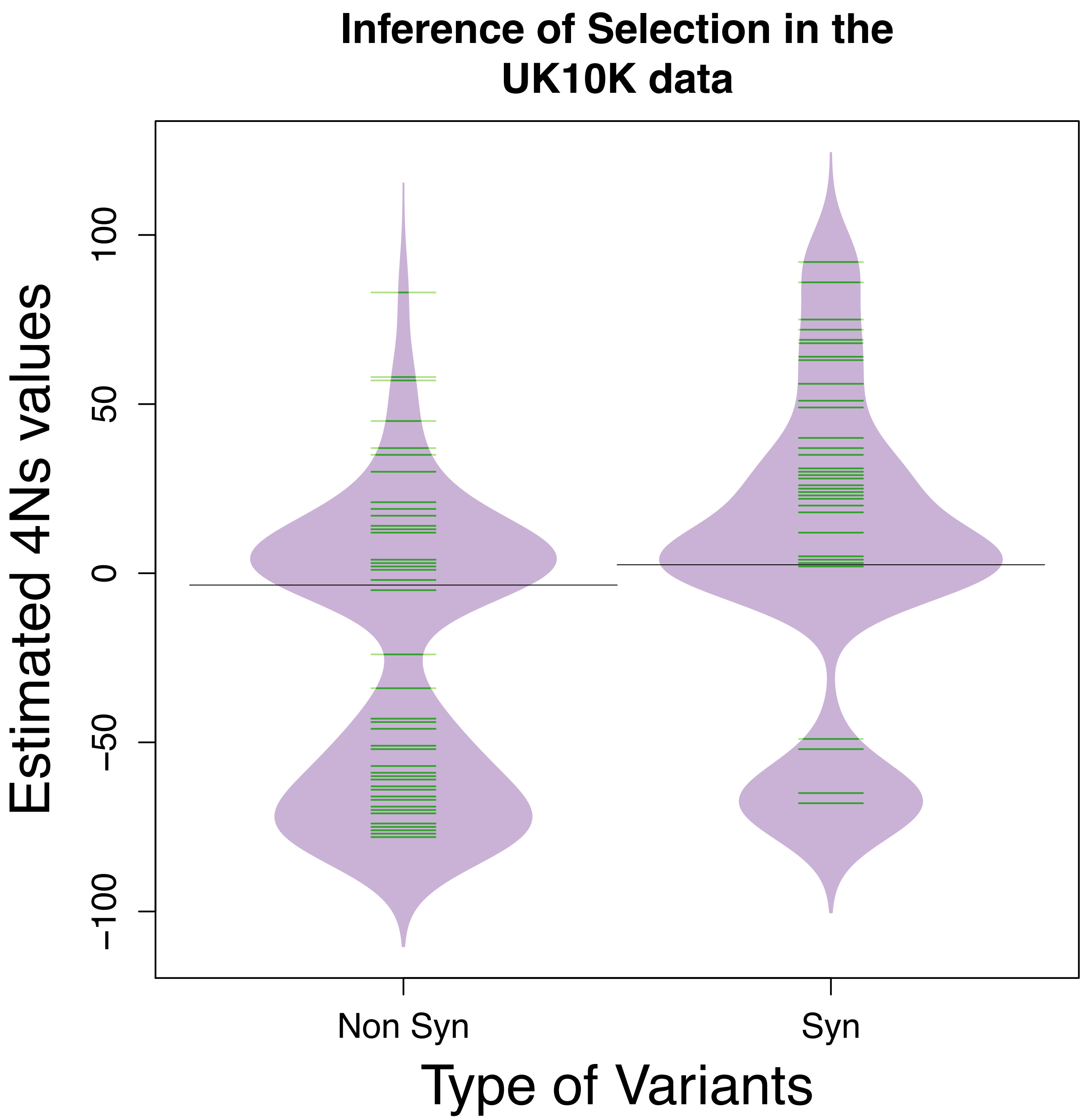

Figure S41

Variation around the estimated $4Ns$ values using bootstrap replicates.

We performed 100 bootstrap replicates on the 1% frequency nonsynonymous and synonymous variants. To create the bootstrap replicates, we sampled variants with replacement from our set of synonymous and nonsynonymous variants until we obtained 142 and 275 variants, respectively. Then, we estimated the $\mathbf{4Ns}$ value for each bootstrap replicate. The point estimates of $\mathbf{4Ns}$ in the 275 1% frequency nonsynonymous variants of the *UK10K* dataset is equal to $\mathbf{4Ns} = -50$ and $\mathbf{4Ns} = 3$ for the 142 1% frequency synonymous variants.

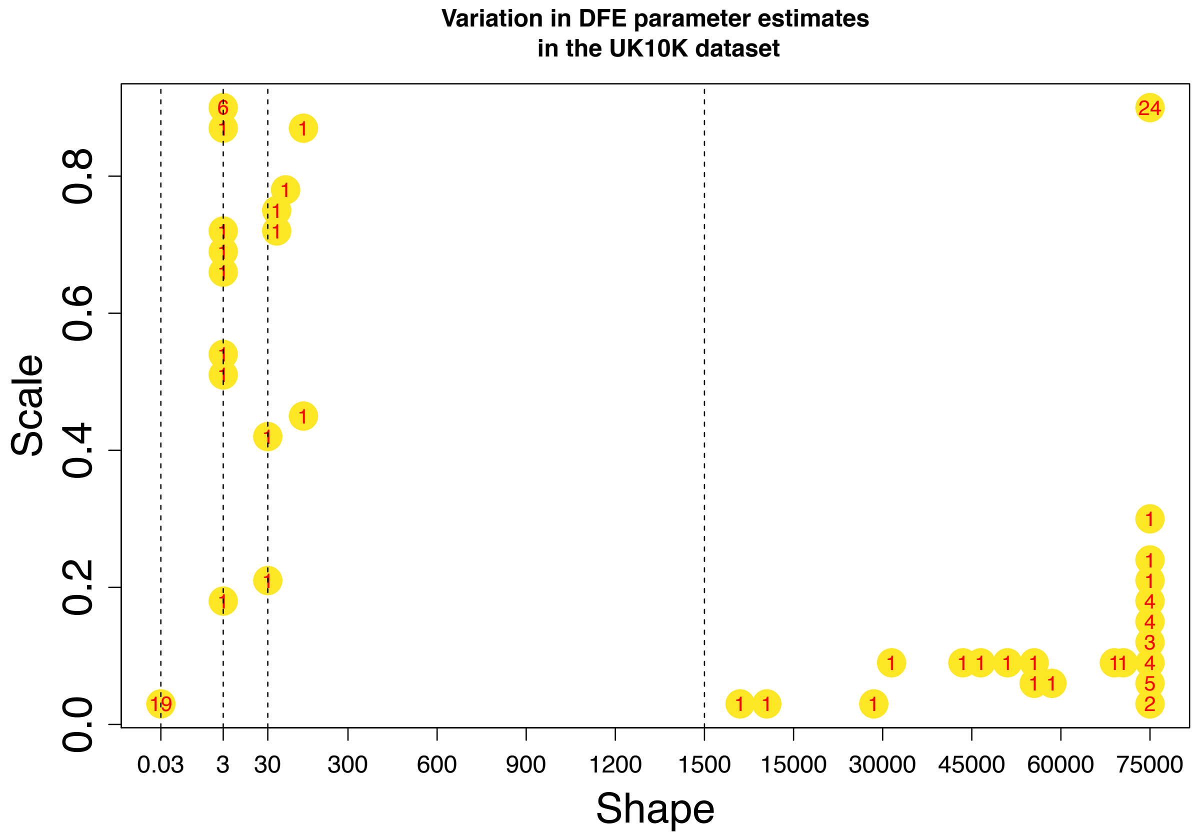

Figure S42

Variation in estimates of the distribution of fitness effects of 1% frequency nonsynonymous variants in the *UK10K* dataset assessed using a bootstrap approach.

We created each bootstrap replicate by sampling variants with replacement from our set of nonsynonymous variants until we obtained 275 variants. A) Estimated scale and shape parameters of the compound distribution in 100 bootstrap replicates. The red numbers indicate the number of bootstrap replicates found in each combination of scale and shape parameters. We found that estimates of the shape parameter tended to cluster on the edges of the searched parameter values. We did not explore higher values of the shape parameter because we found that our method showed low *ESS*, smaller than 100, when $4Ns$ was smaller than -78 (Figure S30) and the shape values were large enough that more than 99% of the probability was clustered at the highest $4Ns$ value explored ($\tau = 75$) when defining the probability distributions as seen in equation 3.

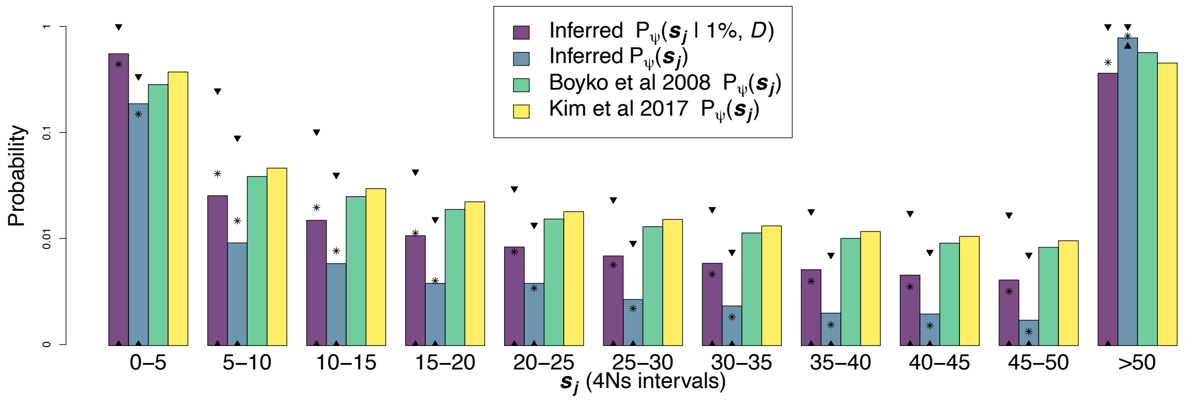

Figure S43

Inferred distribution of fitness effects of new mutations and 1% frequency deleterious variants in the *UK10K* dataset.

'Inferred $P_\psi(s_j)$' refers to the probability of having a $4Ns$ value in a particular interval s_j given the distribution of fitness effects of new mutations DFE. 'Inferred $P_\psi(s_j \mid 1\%, D)$' is the probability of having a $4Ns$ value in a particular interval s_j given that the variant has a 1% frequency, and the demographic scenario D. The selection coefficient s refers exclusively to the action of deleterious variants in this plot. We compared our inferences with those of Boyko et al. (2008) and Kim et al. (2017). The two triangles shown in each s_j interval denote the upper and lower limit of the 90% bootstrap percentile interval across 100 bootstrap replicates. The asterisk signs are the mean values for the inferred probabilities $P_\psi(s_j \mid 1\%, D)$ and $P_\psi(s_j)$ calculated from 100 bootstrap replicates.

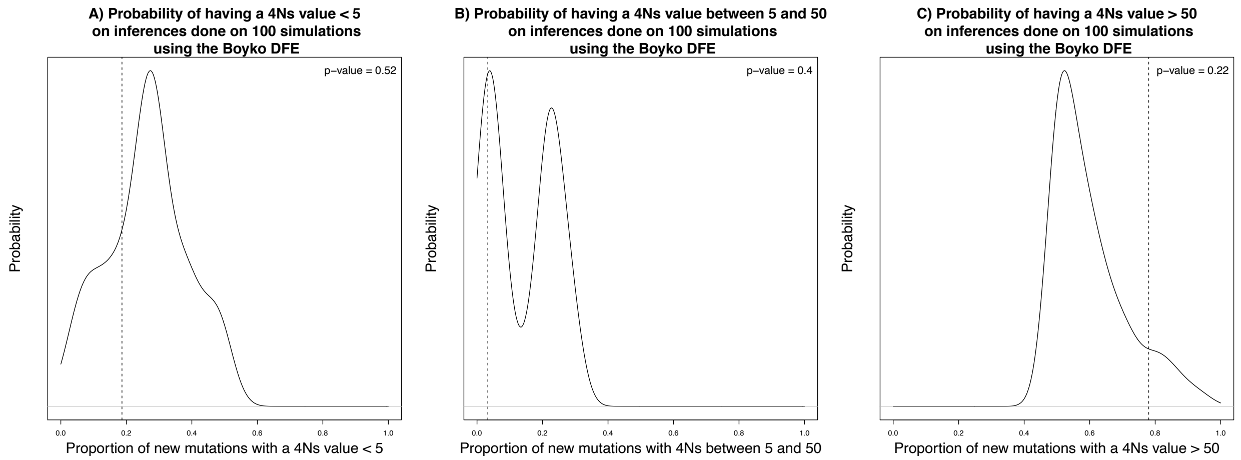

Figure S44

Probability distributions of the inferred proportion of variants having a A) $4Ns$ value < 5, B) $4Ns$ value between 5 and 50, and c) $4Ns$ value > 50 on 100 simulations done using the Boyko et al. (2008) distribution of fitness effects.

We took the results of the inferred $P_{\psi}(\boldsymbol{s_j})$ shown in the bottom plot from Supplementary Figure S31 and created probability density plots for the 3 $\boldsymbol{s_j}$ intervals shown there. We used those results to create null distributions of inferred $P_{\psi}(\boldsymbol{s_j})$ values under the assumption that we used the Boyko et al. (2008) DFE.

The vertical dashed lines shown in every plot represent the values of $P_{\psi}(\boldsymbol{s_j})$ in each $\boldsymbol{s_j}$ bin as calculated from the *UK10K* dataset. We calculated a p-value based on the results from the *UK10K* dataset and the null distributions of $P_{\psi}(\boldsymbol{s_j})$ values. Based on the p-values, we can not reject the null hypothesis that the proportion of variants that have a *4Ns* value in a particular $\boldsymbol{s_j}$ interval in the *UK10K* dataset is different from what is expected under the Boyko distribution of fitness effects.

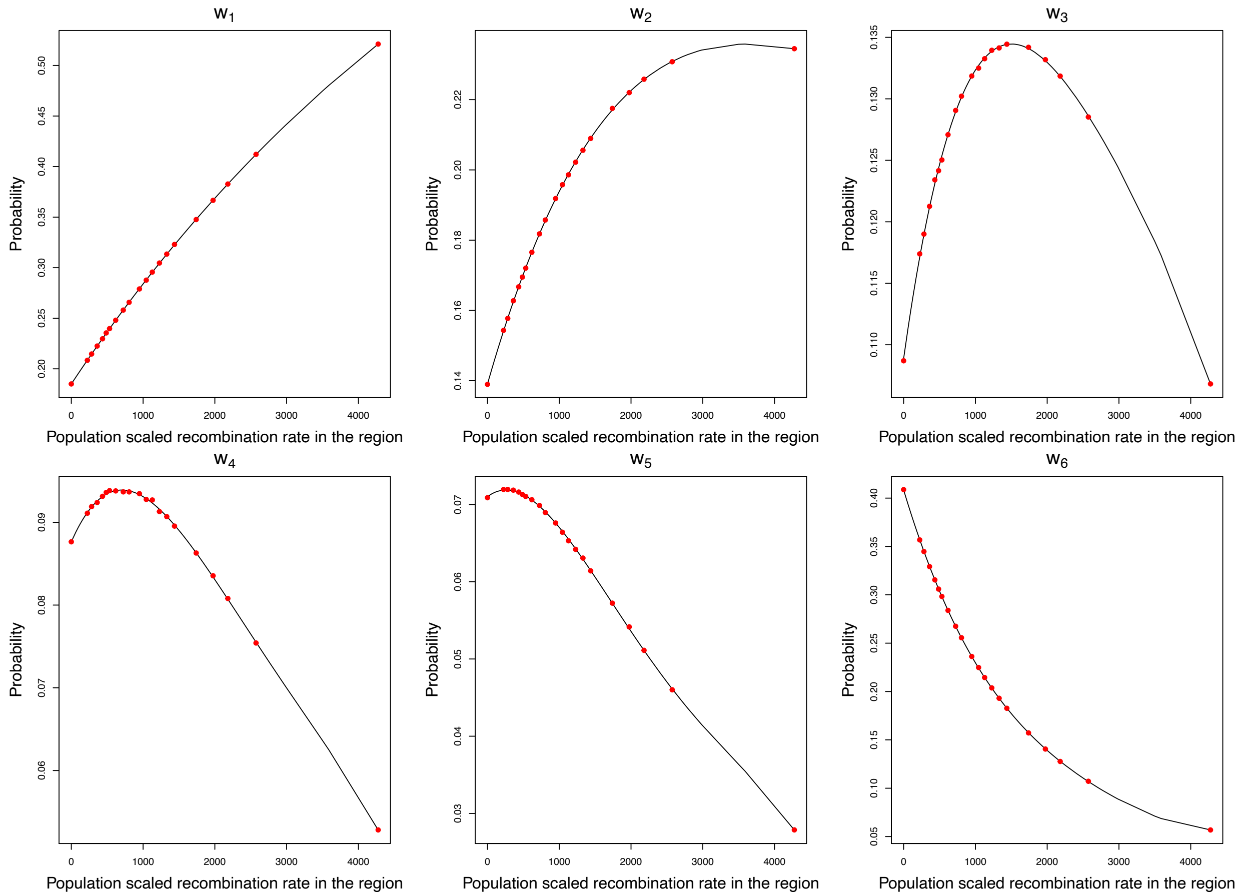

Figure S45

Fit of the polynomial regression model to the probabilities $P(L \in w_i)$ under different population scaled recombination rates when $4Ns = 0$ under the population expansion model

The red points show estimated values of $P(L \in w_i)$ under 21 different percentile values (0th, 5th, …, 95th, 100th) from the distribution of 300 average population-scaled recombination rates taking into account the 250 kb upstream and downstream regions next to the 300 nonsynonymous 1% frequency variants. The line represents the polynomial regression model fit to the data.

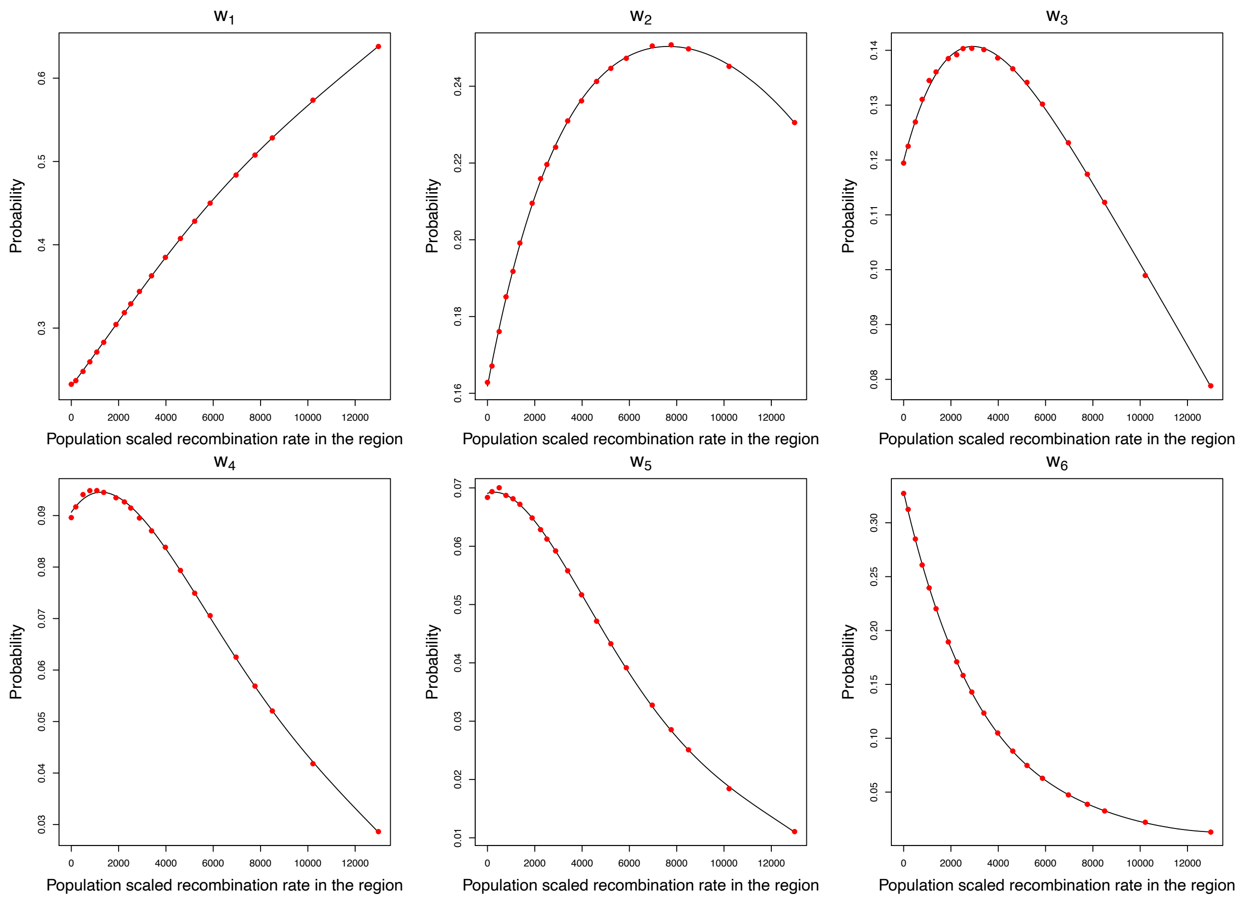

Figure S46

Fit of the polynomial regression model to the probabilities $P(L \in w_i)$ under different population scaled recombination rates when $4Ns = 0$

The red points show estimated values of $P(L \in w_i)$ under 20 different percentile values (0th, 5th, ..., 90th, 95th) from the distribution of 546 average population-scaled recombination rates in the upstream and downstream 250 kb regions next to the 275 nonsynonymous 1% frequency variants. The line represents the polynomial regression model fit to the data. We discarded the data from regions where the population-scaled recombination rate was bigger than the 95th percentile.

Table S1
Notation used in the manuscript

DFE	Distribution of fitness effects for new mutations
s	Selection coefficient
SFS	Site frequency spectrum
f	Sample allele frequency
DFE_f	Distribution of fitness effects of variants at a frequency *f*
L_j and L	Haplotypic identity-by-state length between a pair of haplotypes
$\boldsymbol{W} = \{w_1, w_2, \ldots, w_M\}$	Set of discrete non-overlapping windows that extend to the side of the derived allele (see Figure 1)
M	Number of non-overlapping windows at a side of the derived allele.
A	Number of loci that contain a derived allele at a frequency *f*
A_{n_i}	Number of loci that contain n_i copies of the derived allele
n	Number of haplotypes with a derived allele
N	Effective population size in the most ancient epoch of the demographic scenario *D*
D	Demographic scenario
N_R	Effective population size in the most recent epoch
$4Ns$	Population-scaled selection coefficient
$H_k = \{i_T, i_{T-1}, i_{T-2}, \ldots, i_2, i_1, i_0\}$	A particular allele frequency trajectory. i_T is equal to the number of alleles in generation T. The number of alleles is listed going forwards in time. Generation *T* is the most ancient generation.
$\boldsymbol{L}$	Collection of observed pairwise identity-by-state lengths
T_2	Coalescent time between a pair of haplotypes
l	Length of the simulated region
u	Mutation rate per base per generation
r	Recombination rate per base per generation
$\theta = 4Nul$	Population-scaled mutation rate
$\rho = 4Nrl$	Population-scaled recombination rate
α	Shape parameter of a gamma distribution
β	Scale parameter of a gamma distribution
τ	Upper threshold of the *4Ns* values explored in the partially collapsed gamma distribution (see Equation 3)
$DFE_f(\alpha, \beta)$	A partially collapsed gamma distribution that defines the DFE_f
$\boldsymbol{s_j}$	Interval of $4Ns$ values $[4Ns_0, 4Ns_1)$, where s_0 and s_1 define two different selection

	coefficients.
$\boldsymbol{s}$	Set of non-overlapping intervals $\boldsymbol{s}$ = {[$4Ns_0$, $4Ns_1$), [$4Ns_1$, $4Ns_2$), [$4Ns_2$, $4Ns_3$)… , [$4Ns_{b-1}$, $4Ns_b$)} = { $\boldsymbol{s_1}, \boldsymbol{s_2}, \boldsymbol{s_3}, \ldots, \boldsymbol{s_b}$ }.
ψ	Vector of the parameters $\psi = \{\psi_1, \psi_2, \psi_3, \ldots, \psi_\kappa\}$ that define the *DFE*.
$\boldsymbol{N} = \{N_T, N_{T-1}, N_{T-2}, \ldots, N_2, N_1, N_0\}$	Effective population sizes from generation T to generation *0*, where the population sizes are listed going forwards in time. Generation *T* is the most ancient generation.
ℓ	Number of haplotype pairs evaluated

Table S2

Comparison of estimates of the mean allele age of a 1% frequency variant in a demographic scenario of a constant population size ($N = 10,000$).

The theoretical estimates were obtained by Maruyama (1974) and assume that the populations are in the diffusion limit, where N tends to infinity and the value of $4Ns$ tends to a fixed constant. We report an estimate of allele age from forward-in-time simulations using the mean allele age across $10,000$ forward-in-time allele frequency trajectories. Our estimate is based on alleles that were sampled at a 1% frequency based on a sample of $4,000$ chromosomes. We also report the standard deviation of the allele ages, shown inside parenthesis, for the forward-in-time simulations.

$4Ns$	0	-50	50	-100	100
Maruyama's theoretical estimates	1861	654	654	474	474
Forward-in-time simulation estimates based on the sample allele frequency (standard deviation)	1872.69 (5295.08)	641.49 (636.20)	649.00 (635.53)	462.68 (371.32)	468.42 (377.05)

Table S3
Estimated values of $P_{\psi}(f|D)$ and $P_{\psi}(f|s_j, D)$ using 2,500 simulations under three demographic models that include a population expansion.
The only difference between the three models is the number of generations that the model was run for the first epoch: 80000, 100000 or 120000 generations. After that first epoch, the population grows to 50000 individuals for 100 generations in the three models. Note that the ratio of $P_{\psi}(f|D)$ / $P_{\psi}(f|\boldsymbol{s_j}, D)$ is very similar for the three models under all the $\boldsymbol{s_j}$ intervals inspected. This is important because if we use the same probability value of $P_{\psi}(\boldsymbol{s_j}|f, D)$, we should obtain the same value of $P_{\psi}(\boldsymbol{s_j})$ for the three demographic models since $P_{\psi}(\boldsymbol{s_j}) = \frac{P_{\psi}(\boldsymbol{s_j}|f,D)\, P_{\psi}(f|D)}{P_{\psi}(f|\boldsymbol{s_j},D)}$. The values of $P_{\psi}(f|D)$ were equal to 3.08 X 10^{-7}, 2.47 X 10^{-7} and to 2.07 X 10^{-7} for the demographic models ran for 80000, 100000 or 120000 generations during the first epoch, respectively.
The number of new mutations appearing each generation follows a Poisson distribution with a mean equal to $2N_i ul = 1{,}000$ in the ancestral epoch in each simulation, where N_i is the population size in generation i. The mean of the Poisson distribution changes between epochs as defined by the N_i values.

$\boldsymbol{s_j}$	$P_{\psi}(f\|\boldsymbol{s_j}, D)$ First epoch ran for 80000 generations	$P_{\psi}(f\|\boldsymbol{s_j}, D)$ First epoch ran for 100000 generations	$P_{\psi}(f\|\boldsymbol{s_j}, D)$ First epoch ran for 120000 generations	$\frac{P_{\psi}(f\|D)}{P_{\psi}(f\|\boldsymbol{s_j}, D)}$ First epoch ran for 80000 generations	$\frac{P_{\psi}(f\|D)}{P_{\psi}(f\|\boldsymbol{s_j}, D)}$ First epoch ran for 100000 generations	$\frac{P_{\psi}(f\|D)}{P_{\psi}(f\|\boldsymbol{s_j}, D)}$ First epoch ran for 120000 generations
0-5	6.17E-07	4.95E-07	4.08E-07	0.50	0.50	0.51
5-10	5.81E-07	4.74E-07	3.85E-07	0.53	0.52	0.54
10-15	5.58E-07	4.34E-07	3.73E-07	0.55	0.57	0.56
15-20	5.15E-07	4.06E-07	3.52E-07	0.60	0.61	0.59
20-25	4.98E-07	4.01E-07	3.21E-07	0.62	0.62	0.65
25-30	4.49E-07	3.70E-07	3.18E-07	0.69	0.67	0.65
30-35	4.42E-07	3.36E-07	2.96E-07	0.70	0.74	0.70
35-40	3.97E-07	3.22E-07	2.76E-07	0.78	0.77	0.75
40-45	3.83E-07	2.86E-07	2.45E-07	0.81	0.87	0.84
45-50	3.33E-07	2.95E-07	2.27E-07	0.93	0.84	0.91
50-55	3.18E-07	2.60E-07	2.23E-07	0.97	0.95	0.93
55-60	2.92E-07	2.37E-07	2.06E-07	1.06	1.04	1.01
60-65	2.60E-07	2.24E-07	1.88E-07	1.18	1.11	1.10
65-70	2.62E-07	2.05E-07	1.77E-07	1.17	1.20	1.17
70-75	2.57E-07	1.75E-07	1.38E-07	1.20	1.42	1.50
75-80	2.33E-07	1.81E-07	1.38E-07	1.33	1.37	1.50
80-85	2.16E-07	1.55E-07	1.37E-07	1.43	1.60	1.51
85-90	1.67E-07	1.39E-07	1.36E-07	1.85	1.78	1.52
90-95	1.25E-07	1.19E-07	9.17E-08	2.46	2.07	2.26
95-100	1.58E-07	9.41E-08	8.21E-08	1.96	2.63	2.52
> 100	4.73E-09	3.35E-09	3.23E-09	65.20	72.04	64.05

Table S4

Estimated values of $P_\psi(f|D)$ and $P_\psi(f|s_j, D)$ using 2500 simulations under three demographic models of a constant population size.

The only difference between the three models is the number of generations that the model was run: 160000, 180000 or 200000 generations. Note that the ratio of $P_\psi(f|D)$ / $P_\psi(f|\boldsymbol{s_j}, D)$ is very similar for the three models under all the $\boldsymbol{s_j}$ intervals inspected. This is important because if we use the same probability value of $P_\psi(\boldsymbol{s_j}|f, D)$, we should obtain the same value of $P_\psi(\boldsymbol{s_j})$ for the three demographic models since $P_\psi(\boldsymbol{s_j}) = \frac{P_\psi(\boldsymbol{s_j}|f,D)\, P_\psi(f|D)}{P_\psi(f|\boldsymbol{s_j},D)}$. The values of $P_\psi(f|D)$ were equal to 1.45 X 10^{-7}, 1.30 X 10^{-7} and to 1.17 X 10^{-7} for the demographic models ran for 160000, 180000 or 200000 generations during the first epoch, respectively.

The number of new mutations appearing each generation follows a Poisson distribution with a mean equal to $2N_i ul = 1{,}000$ in the ancestral epoch in each simulation, where N_i is the population size in generation i. The mean of the Poisson distribution changes between epochs as defined by the N_i values.

$\boldsymbol{s_j}$	$P_\psi(f\|\boldsymbol{s_j}, D)$ First epoch ran for 80000 generations	$P_\psi(f\|\boldsymbol{s_j}, D)$ First epoch ran for 100000 generations	$P_\psi(f\|\boldsymbol{s_j}, D)$ First epoch ran for 120000 generations	$\frac{P_\psi(f\|D)}{P_\psi(f\|\boldsymbol{s_j}, D)}$ First epoch ran for 160000 generations	$\frac{P_\psi(f\|D)}{P_\psi(f\|\boldsymbol{s_j}, D)}$ First epoch ran for 180000 generations	$\frac{P_\psi(f\|D)}{P_\psi(f\|\boldsymbol{s_j}, D)}$ First epoch ran for 200000 generations
0-5	3.12E-07	2.78E-07	2.49E-07	0.46	0.47	0.47
5-10	3.00E-07	2.59E-07	2.40E-07	0.48	0.50	0.49
10-15	2.80E-07	2.50E-07	2.28E-07	0.52	0.52	0.51
15-20	2.54E-07	2.24E-07	2.09E-07	0.57	0.58	0.56
20-25	2.46E-07	2.32E-07	2.02E-07	0.59	0.56	0.58
25-30	2.27E-07	2.16E-07	2.02E-07	0.64	0.60	0.58
30-35	2.23E-07	1.97E-07	1.87E-07	0.65	0.66	0.63
35-40	2.29E-07	2.03E-07	1.73E-07	0.63	0.64	0.67
40-45	1.95E-07	1.74E-07	1.76E-07	0.74	0.75	0.66
45-50	1.97E-07	1.59E-07	1.57E-07	0.74	0.82	0.74
50-55	1.82E-07	1.57E-07	1.58E-07	0.80	0.83	0.74
55-60	1.77E-07	1.59E-07	1.35E-07	0.82	0.82	0.86
60-65	1.74E-07	1.56E-07	1.37E-07	0.84	0.84	0.85
65-70	1.56E-07	1.30E-07	1.35E-07	0.93	1.00	0.87
70-75	1.58E-07	1.30E-07	1.08E-07	0.92	1.00	1.08
75-80	1.50E-07	1.36E-07	1.24E-07	0.97	0.96	0.94
80-85	1.39E-07	1.08E-07	1.15E-07	1.04	1.20	1.02
85-90	1.35E-07	1.15E-07	1.11E-07	1.08	1.13	1.05
90-95	1.13E-07	1.19E-07	1.14E-07	1.28	1.09	1.02
95-100	1.15E-07	1.29E-07	7.80E-08	1.27	1.01	1.50
> 100	7.24E-09	6.51E-09	6.05E-09	20.05	20.00	19.28

Table S5

Proportion $P_{incorrect}(f)$ of ancestral alleles at a frequency $f = 1\%$ incorrectly called as derived alleles.

We performed 500 forward-in-time simulations of independent variants using *PReFerSim* under two alternative demographic models, the constant population size model and the population expansion model. We recorded the number of sites $F_{True}(f)$ and $F_{True}(1-f)$ that contain a derived allele and an ancestral allele at a frequency f, respectively. We calculated $P_{incorrect}(f)$ using the equation (S19) with a p_{misid} value equal to 0.015.

The number of new mutations appearing each generation follows a Poisson distribution with a mean equal to $2N_i ul = 1{,}000$ in the ancestral epoch in each simulation, where N_i is the population size in generation i. The mean of the Poisson distribution changes between epochs as defined by the N_i values.

Constant population size model

4Ns	$F_{True}(f)$	$F_{True}(1-f)$	$P_{incorrect}(f)$
0	25046	246	0.00015
-50	15423	0	0.0
-100	9331	0	0.0
50	25248	9821	0.00589
100	25272	15735	0.00939

Population expansion model

4Ns	$F_{True}(f)$	$F_{True}(1-f)$	$P_{incorrect}(f)$
0	24961	234	0.00014
-50	13515	0	0.0
-100	5504	0	0.0
50	25249	11615	0.00696
100	25162	19752	0.01181

Table S6

Proportion $P_{incorrect}(f)$ of ancestral alleles at a frequency $f = 1\% \pm 0.05\%$ incorrectly called as derived alleles.

We performed 500 forward-in-time simulations of independent variants using *PReFerSim* under the scaled *UK10K* demographic model. We recorded the number of sites $F_{True}(f)$ and $F_{True}(1 - f)$ that contain a derived allele and an ancestral allele at a frequency f, respectively. We calculated $P_{incorrect}(f)$ using the equation (S19) with a p_{misid} value equal to 0.015. The number of new mutations appearing each generation follows a Poisson distribution with a mean equal to $2N_i ul = 1{,}000$ in the ancestral epoch in each simulation, where N_i is the population size in generation i. The mean of the Poisson distribution changes between epochs as defined by the N_i values.

4Ns	f (number of derived alleles/number of chromosomes)	$F_{True}(f)$	$F_{True}(1-f)$	$P_{incorrect}(f)$
0	69/7242	4519	142	0.00048
0	70/7242	4372	148	0.00052
0	71/7242	4268	143	0.00051
0	72/7242	4286	143	0.00051
0	73/7242	4404	138	0.00048
0	74/7242	4167	133	0.00049
0	75/7242	4082	140	0.00052
0	76/7242	4047	138	0.00052

4Ns	f (number of derived alleles/number of chromosomes)	$F_{True}(f)$	$F_{True}(1-f)$	$P_{incorrect}(f)$
25	69/7242	4427	1429	0.00489
25	70/7242	4255	1467	0.00522
25	71/7242	4280	1421	0.00503
25	72/7242	4220	1455	0.00522
25	73/7242	4253	1433	0.00510
25	74/7242	4263	1454	0.00517
25	75/7242	3984	1460	0.00555
25	76/7242	3892	1460	0.00568

4Ns	f (number of derived alleles/number of	$F_{True}(f)$	$F_{True}(1-f)$	$P_{incorrect}(f)$

	chromosomes)			
50	69/7242	4123	3911	0.01424
50	70/7242	4039	3947	0.01466
50	71/7242	3985	4022	0.01514
50	72/7242	4078	4079	0.01500
50	73/7242	3878	4012	0.01551
50	74/7242	3956	3936	0.01493
50	75/7242	3901	4037	0.01551
50	76/7242	3873	4079	0.01579

4Ns	*f* (number of derived alleles/number of chromosomes)	$F_{True}(f)$	$F_{True}(1-f)$	$P_{incorrect}(f)$
-25	69/7242	3780	0	0.00000
-25	70/7242	3612	0	
-25	71/7242	3684	0	0.00000
-25	72/7242	3588	0	0.00000
-25	73/7242	3617	0	0.00000
-25	74/7242	3511	0	0.00000
-25	75/7242	3457	0	0.00000
-25	76/7242	3373	0	0.00000

4Ns	*f* (number of derived alleles/number of chromosomes)	$F_{True}(f)$	$F_{True}(1-f)$	$P_{incorrect}(f)$
-50	69/7242	3494	0	0.00000
-50	70/7242	3396	0	0.00000
-50	71/7242	3386	0	0.00000
-50	72/7242	3246	0	0.00000
-50	73/7242	3217	0	0.00000
-50	74/7242	3176	0	0.00000
-50	75/7242	3071	0	0.00000
50	76/7242	2941	0	0.00000

Table S7

Percentiles of the Inferred '$P_{\psi}(s_j)$' or '$P_{\psi}(s_j|f, D)$' probabilities estimated across 100 simulation replicates for different s_j intervals based on Figure S39.

$P_{\psi}(s_j)$

s_j	25% and 75% percentile	10% and 90% percentile	5% and 95% percentile	2.5% and 97.5% percentile	Probability from Boyko et al. (2008) scaled by the ancestral population size in the scaled *UK10K* model
0-5	(0.131, 0.372)	(0.052, 0.500)	(0.034, 0.552)	(0.027, 0.610)	0.381
5-10	(0.019, 0.085)	(0.012, 0.172)	(0.012, 0.172)	(0.005, 0.172)	0.052
10-15	(0.011, 0.061)	(0.007, 0.073)	(0.007, 0.073)	(0.003, 0.073)	0.033
15-20	(0.008, 0.038)	(0.005, 0.049)	(0.005, 0.051)	(0.002, 0.052)	0.025
20-25	(0.007, 0.031)	(0.004, 0.045)	(0.004, 0.045)	(0.002, 0.045)	0.020
25-30	(0.005, 0.028)	(0.003, 0.039)	(0.003, 0.040)	(0.001, 0.040)	0.017
30-35	(0.003, 0.026)	(0.003, 0.037)	(0.003, 0.039)	(0.0, 0.039)	0.015
35-40	(0.003, 0.021)	(0.001, 0.031)	(0.001, 0.035)	(0.0, 0.035)	0.013
40-45	(0.002, 0.020)	(0.000, 0.028)	(0.0, 0.032)	(0.0, 0.032)	0.012
45-50	(0.002, 0.018)	(0.000, 0.025)	(0.0, 0.029)	(0.0, 0.029)	0.011
>50	(0.390, 0.652)	(0.328, 0.763)	(0.328, 0.789)	(0.328, 0.847)	0.421

$$P_{\psi}(\boldsymbol{s_j}|f, D)$$

s_j	25% and 75% percentile	10% and 90% percentile	5% and 95% percentile	2.5% and 97.5% percentile
0-5	(0.204, 0.576)	(0.081, 0.775)	(0.053, 0.854)	(0.042, 0.945)
5-10	(0.027, 0.122)	(0.017, 0.248)	(0.017, 0.248)	(0.007, 0.248)
10-15	(0.016, 0.085)	(0.010, 0.102)	(0.010, 0.102)	(0.005, 0.102)
15-20	(0.011, 0.051)	(0.007, 0.067)	(0.007, 0.070)	(0.003, 0.071)
20-25	(0.009, 0.042)	(0.006, 0.060)	(0.005, 0.060)	(0.003, 0.060)
25-30	(0.007, 0.037)	(0.004, 0.052)	(0.004, 0.054)	(0.001, 0.054)
30-35	(0.004, 0.032)	(0.003, 0.045)	(0.003, 0.049)	(0.001, 0.049)
35-40	(0.003, 0.027)	(0.001, 0.040)	(0.001, 0.044)	(0.0, 0.044)
40-45	(0.003, 0.025)	(0.001, 0.035)	(0.001, 0.040)	(0.0, 0.040)
45-50	(0.003, 0.022)	(0.0, 0.031)	(0.0, 0.037)	(0.0, 0.037)
>50	(0.094, 0.476)	(0.0, 0.649)	(0.0, 0.715)	(0.0, 0.785)

Table S8

Percentiles of the Inferred '$P_{\psi}(s_j)$' or '$P_{\psi}(s_j|f,D)$' probabilities estimated across 100 simulation replicates for different s_j intervals based on Figure S40.

$P_{\psi}(s_j)$

s_j	25% and 75% percentile	10% and 90% percentile	5% and 95% percentile	2.5% and 97.5% percentile	Probability from Boyko et al. (2008) scaled by the ancestral population size in the *UK10K* model
0-5	(0.165, 0.358)	(0.080, 0.423)	(0.066, 0.481)	(0.047, 0.481)	0.283
5-10	(0.010, 0.084)	(0.006, 0.126)	(0.0, 0.126)	(0.0, 0.126)	0.038
10-15	(0.007, 0.047)	(0.004, 0.055)	(0.0, 0.055)	(0.0, 0.055)	0.025
15-20	(0.005, 0.025)	(0.003, 0.038)	(0.0, 0.041)	(0.0, 0.041)	0.019
20-25	(0.003, 0.011)	(0.002, 0.029)	(0.0, 0.033)	(0.0, 0.033)	0.015
25-30	(0.003, 0.008)	(0.001, 0.024)	(0.0, 0.029)	(0.0, 0.029)	0.013
30-35	(0.002, 0.008)	(0.0, 0.024)	(0.0, 0.030)	(0.0, 0.030)	0.011
35-40	(0.001, 0.005)	(0.0, 0.017)	(0.0, 0.022)	(0.0, 0.022)	0.010
40-45	(0.000, 0.005)	(0.0, 0.014)	(0.0, 0.019)	(0.0, 0.020)	0.009
45-50	(0.000, 0.004)	(0.0, 0.012)	(0.0, 0.017)	(0.0, 0.017)	0.008
>50	(0.508, 0.654)	(0.5, 0.788)	(0.5, 0.839)	(0.5, 0.871)	0.568

$$P_{\psi}(\boldsymbol{s_j}|f, D)$$

s_j	25% and 75% percentile	10% and 90% percentile	5% and 95% percentile	2.5% and 97.5% percentile
0-5	(0.344, 0.744)	(0.166, 0.880)	(0.138, 1.0)	(0.097, 1.0)
5-10	(0.020, 0.165)	(0.011, 0.248)	(0.0, 0.248)	(0.0, 0.248)
10-15	(0.012, 0.086)	(0.008, 0.102)	(0.0, 0.102)	(0.0, 0.102)
15-20	(0.009, 0.043)	(0.006, 0.066)	(0.0, 0.070)	(0.0, 0.071)
20-25	(0.006, 0.019)	(0.004, 0.053)	(0.0, 0.060)	(0.0, 0.061)
25-30	(0.005, 0.015)	(0.002, 0.044)	(0.0, 0.052)	(0.0, 0.053)
30-35	(0.003, 0.012)	(0.001, 0.037)	(0.0, 0.046)	(0.0, 0.047)
35-40	(0.001, 0.010)	(0.0, 0.031)	(0.0, 0.041)	(0.0, 0.042)
40-45	(0.001, 0.009)	(0.0, 0.027)	(0.0, 0.036)	(0.0, 0.037)
45-50	(0.000, 0.007)	(0.0, 0.024)	(0.0, 0.032)	(0.0, 0.033)
>50	(0.0, 0.317)	(0.0, 0.572)	(0.0, 0.678)	(0.0, 0.744)

Table S9
Upper and lower limit of different bootstrap percentile intervals of the Inferred '$P_{\psi}(s_j)$' and '$P_{\psi}(s_j|f, D)$' probabilities estimated across 100 bootstrap replicates for different s_j intervals.

$P_{\psi}(s_j)$

s_j	50% bootstrap percentile interval	80% bootstrap percentile interval	90% bootstrap percentile interval	95% bootstrap percentile interval
0-5	(0.015, 0.253)	(0.0, 0.338)	(0.0, 0.338)	(0.0, 0.338)
5-10	(0.0, 0.010)	(0.0, 0.058)	(0.0, 0.089)	(0.0, 0.089)
10-15	(0.0, 0.007)	(0.0, 0.027)	(0.0, 0.040)	(0.0, 0.040)
15-20	(0.0, 0.004)	(0.0, 0.014)	(0.0, 0.015)	(0.0, 0.016)
20-25	(0.0, 0.004)	(0.0, 0.008)	(0.0, 0.013)	(0.0, 0.017)
25-30	(0.0, 0.003)	(0.0, 0.003)	(0.0, 0.009)	(0.0, 0.014)
30-35	(0.0, 0.003)	(0.0, 0.003)	(0.0, 0.007)	(0.0, 0.012)
35-40	(0.0, 0.002)	(0.0, 0.002)	(0.0, 0.007)	(0.0, 0.011)
40-45	(0.0, 0.002)	(0.0, 0.002)	(0.0, 0.007)	(0.0, 0.011)
45-50	(0.0, 0.002)	(0.0, 0.002)	(0.0, 0.007)	(0.0, 0.009)
>50	(0.662, 0.947)	(0.654, 1.000)	(0.648, 1.000)	(0.648, 1.000)

$$P_\psi(\boldsymbol{s_j}|f, D)$$

$\boldsymbol{s_j}$	50% bootstrap percentile interval	80% bootstrap percentile interval	90% bootstrap percentile interval	95% bootstrap percentile interval
0-5	(0.043, 0.748)	(0.0, 1.0)	(0.0, 1.0)	(0.0, 1.0)
5-10	(0.0, 0.029)	(0.0, 0.161)	(0.0, 0.248)	(0.0, 0.248)
10-15	(0.0, 0.017)	(0.0, 0.068)	(0.0, 0.102)	(0.0, 0.102)
15-20	(0.0, 0.013)	(0.0, 0.039)	(0.0, 0.043)	(0.0, 0.046)
20-25	(0.0, 0.010)	(0.0, 0.018)	(0.0, 0.030)	(0.0, 0.038)
25-30	(0.0, 0.008)	(0.0, 0.008)	(0.0, 0.023)	(0.0, 0.035)
30-35	(0.0, 0.007)	(0.0, 0.007)	(0.0, 0.019)	(0.0, 0.031)
35-40	(0.0, 0.006)	(0.0, 0.006)	(0.0, 0.018)	(0.0, 0.028)
40-45	(0.0, 0.005)	(0.0, 0.006)	(0.0, 0.017)	(0.0, 0.025)
45-50	(0.0, 0.005)	(0.0, 0.005)	(0.0, 0.017)	(0.0, 0.023)
>50	(0.0, 0.854)	(0.0, 0.999)	(0.0, 0.999)	(0.0, 0.999)

Table S10

Comparison of selection estimates of new mutations using our method and the estimates obtained by Kim et al. (2017) and Boyko et al. (2008).

The estimates we obtained for the *UK10K* dataset were computed using the median and the 90% bootstrap percentile interval for each of the 4 selection intervals shown below. To do this, we generated 100 bootstrap replicates, where we created each bootstrap replicate by sampling variants with replacement from our set of nonsynonymous variants until we get 275 variants. Then, we estimated the parameters of the compound distribution for each bootstrap replicate. To obtain the proportion of s values in each of the 4 s intervals shown below, we applied equation 6 using our inferred value of $P_{\psi}(\boldsymbol{s_j}|f, D)$ given our estimates of the compound distribution. In the case of $0 \leq s < 2.18 \times 10^{-5}$ we used $\boldsymbol{s_j} \in [0,1)$. We summed the intervals $\boldsymbol{s_j} \in [1,5)$ and $\boldsymbol{s_j} \in [5,10)$ in the case of $2.18 \times 10^{-5} \leq s < 2.18 \times 10^{-4}$. For the bin $2.18 \times 10^{-4} \leq s < 1.09 \times 10^{-3}$, we summed the values of $P_{\psi}(\boldsymbol{s_j})$ across eight bins $\boldsymbol{s_j} \in [10,15)$, $\boldsymbol{s_j} \in [15,20)$, $\boldsymbol{s_j} \in [20,25)$, $\boldsymbol{s_j} \in [25,30)$, $\boldsymbol{s_j} \in [30,35)$, $\boldsymbol{s_j} \in [35,40)$, $\boldsymbol{s_j} \in [40,45)$ and $\boldsymbol{s_j} \in [45,50)$. The probability that $s > 1.09 \times 10^{-3}$ is simply 1 – P($s < 1.09 \times 10^{-3}$).

We compared the estimates of the 90% bootstrap percentile interval with previously obtained estimates. We assume an N value equal to 11,485 for our estimates to obtain the s values in each $\boldsymbol{s_j}$ interval. That N value is equal to the most ancestral population size in the *UK10K* model (see Figure S28). We report the median across 100 bootstrap replicates for the *UK10K* model and the numbers in parenthesis correspond to the 90% bootstrap percentile interval.

	$0 \leq s < 2.18 \times 10^{-5}$	$2.18 \times 10^{-5} \leq s < 2.18 \times 10^{-4}$	$2.18 \times 10^{-4} \leq s < 1.09 \times 10^{-3}$	$s > 1.09 \times 10^{-3}$
Boyko et al. (2008) (African Americans)	0.211	0.111	0.111	0.568
Kim et al. (2017) (ESP, u = 1.5e-8)	0.284	0.135	0.130	0.452
UK10K	0.163 (4.11e-6, 0.336)	0.032 (2.89e-7, 0.249)	0.024 (0.0, 0.082)	0.781 (0.647, 1.0)

Table S11
Fit of a polynomial regression model with a different number of regression coefficients in the population expansion model.

We define the Average Error ε as:

$$\varepsilon = \frac{\sum_{i=-200}^{200} \sum_{j=1}^{6} A_{ij}}{6 * 401}$$

Where:

$$A_{ij} = \sum_{k=1}^{21} |\mathcal{L}(4Ns = i, f, D, \rho_k \,|L \in w_j) - PRL[\mathcal{L}(4Ns = i, f, D, \rho_k \,|L \in w_j)]|$$
$$= \sum_{k=1}^{21} |P(L \in w_j|4Ns = i, f, D, \rho_k\,) - PRL[P(L \in w_j|4Ns = i, f, D, \rho_k\,)|$$

We define $PRL[P(L \in w_j|4Ns = i, f, D, \rho_k\,)]$ and $PRL[\mathcal{L}(4Ns = i, f, D, \rho_k\,|L \in w_j)]$ as the value predicted for $P(L \in w_j|4Ns = i, f, D, \rho_k\,)$ based on a polynomial regression model with the number of regression coefficients defined in the left cell of the table. The model fit with the polynomial regression line follows this equation:

$$P(L \in w_j|4Ns = i, f, D, \rho_k\,) = \beta_0 + \sum_{i=1}^{c} \beta_i * \rho_k^c$$

Where *c* is the number of regression coefficients fit. In our analysis we chose the smallest number of regression coefficients *c* where the error metric improved by more than 25% compared to the previous number of regression coefficients c – 1, which is 4.

Number of Regression Coefficients *c*	$\boldsymbol{\varepsilon}$	Fit improvement	Average absolute difference between the median inferred 4Ns value and real 4Ns value in inferences over 5 values of 4Ns (100 simulations per 4Ns value)	Mean RMSE on inferences based on 5 values of 4Ns (100 simulations per 4Ns value)
1	0.1959	-	N.A.	N.A.
2	0.0447	0.7720	2	36.6317
3	0.0104	0.7665	5.8	39.2885
4	0.0061	0.4179	5.6	38.7922
5	0.0055	0.0879	4.9	39.6076
6	0.0052	0.0657	4.9	37.2513
7	0.0051	0.0232	N.A.	N.A.
8	0.0046	0.0821	N.A.	N.A.
9	0.0044	0.0565	N.A.	N.A.

Table S12

Fit of a polynomial regression model with a different number of regression coefficients in the UK10K nonsynonymous variants

We define the Average Error ε as:

$$\varepsilon = \frac{\sum_{i=-78}^{92} \sum_{j=1}^{6} A_{ij}}{6 * 171}$$

Where:

$$A_{ij} = \sum_{k=1}^{20} |\mathcal{L}(4Ns = i, f, D, \rho_k \,|L \in w_j) - PRL[\mathcal{L}(4Ns = i, f, D, \rho_k \,|L \in w_j)]|$$

$$= \sum_{k=1}^{21} |P(L \in w_j|4Ns = i, f, D, \rho_k\,) - PRL[P(L \in w_j|4Ns = i, f, D, \rho_k\,)|$$

We define $PRL[P(L \in w_j|4Ns = i, f, D, \rho_k\,)]$ and $PRL[\mathcal{L}(4Ns = i, f, D, \rho_k\,|L \in w_j)]$ as the value predicted for $P(L \in w_j|4Ns = i, f, D, \rho_k\,)$ based on a polynomial regression model with the number of regression coefficients defined in the left cell of the table. The model fit with the polynomial regression line follows this equation:

$$P(L \in w_j|4Ns = i, f, D, \rho_k\,) = \beta_0 + \sum_{i=1}^{c} \beta_i * \rho_k^c$$

Where *c* is the number of regression coefficients fit. In our analysis we chose the smallest number of regression coefficients *c* where the error metric improved by more than 25% compared to the previous number of regression coefficients c – 1, which is 5.

Number of Regression Coefficients *c*	$\boldsymbol{\varepsilon}$	Fit improvement	Average absolute difference between the median inferred 4Ns value and real 4Ns value in inferences over 5 values of 4Ns (100 simulations per 4Ns value)	Mean RMSE on inferences based on 5 values of 4Ns (100 simulations per 4Ns value)
1	0.3813	-	29	36.14218
2	0.1554	0.5925	N.A.	N.A.
3	0.0542	0.6514	7.5	23.05287
4	0.0177	0.6739	5.2	23.96184
5	0.0089	0.4964	5.4	24.00124
6	0.0073	0.1764	5.7	23.99609
7	0.0064	0.1201	5.9	24.00507
8	0.0061	0.0559	5.5	23.83199
9	0.0055	0.1026	5.1	24.59131
